# Alkyltriphenylphosphonium turns naphthoquinoneimidazoles into potent membrane depolarizers against *mycobacteria*†

**DOI:** 10.1039/d2md00251e

**Published:** 2022-10-13

**Authors:** Kevin Timothy Fridianto, Gregory Adrian Gunawan, Kiel Hards, Jickky Palmae Sarathy, Gregory M. Cook, Thomas Dick, Mei-Lin Go, Yulin Lam

**Affiliations:** Department of Chemistry, National University of Singapore 117543 Singapore chmlamyl@nus.edu.sg; Department of Pharmacy, National University of Singapore 117543 Singapore meilin.go@nus.edu.sg; Department of Microbiology and Immunology, University of Otago Dunedin 9054 New Zealand; Center for Discovery and Innovation, Hackensack Meridian Health & Department of Medical Sciences, Hackensack Meridian School of Medicine Nutley NJ 071110 USA thomas.dick.cdi@gmail.com; Department of Microbiology and Immunology, Georgetown University Washington DC USA

## Abstract

Due to its central role in energy generation and bacterial viability, mycobacterial bioenergetics is an attractive therapeutic target for anti-tuberculosis drug discovery. Building upon our work on antimycobacterial dioxonaphthoimidazoliums that were activated by a proximal positive charge and generated reactive oxygen species upon reduction by Type II NADH dehydrogenase, we herein studied the effect of a distal positive charge on the antimycobacterial activity of naphthoquinoneimidazoles by incorporating a trialkylphosphonium cation. The potency-enhancing properties of the linker length were affirmed by structure–activity relationship studies. The most active compound against *M. tb* H37Rv displayed good selectivity index (SI = 34) and strong bactericidal activity in the low micromolar range, which occurred through rapid bacterial membrane depolarization that resulted in depletion of intracellular ATP. Through this work, we demonstrated a switch of the scaffold's mode-of-action *via* relocation of positive charge while retaining its excellent antibacterial activity and selectivity.

## Abstract 2

Herein, we establish the antimycobacterial activity of trialkylphosphonium-containing naphthoquinoneimidazoles, which exhibited limited redox activity but extensive membrane depolarization that resulted in depletion of ATP.

## Graphical abstract

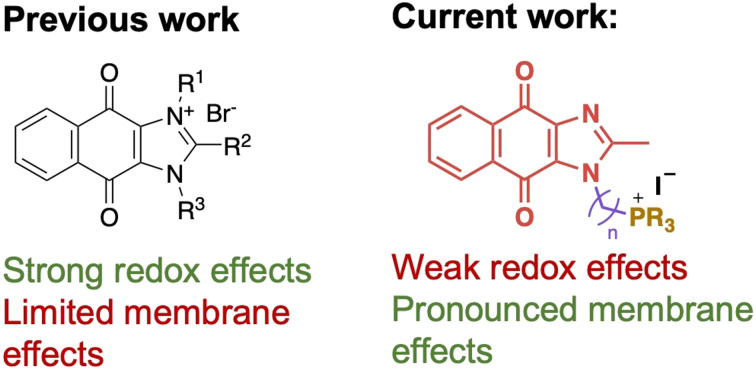

## Introduction

Tuberculosis (TB) is a contagious, airborne disease that remains a major health problem worldwide. In spite of advances in our understanding of the pathogenic organism *Mycobacterium tuberculosis* (*M. tb*), TB remains difficult to treat. Until the outbreak of SARS-CoV-2, more deaths were attributed to *M. tb* than any other infectious agents.^1^ Current TB treatment involves a multidrug regimen lasting for 6–24 months. This characteristically long treatment time is attributed to quiescent *M. tb* which are recalcitrant towards first-line TB drugs^2^ and drug-resistant *M. tb*, which necessitates longer treatments involving second- and third-line TB drugs.^3^ Moreover, most TB drugs used today were discovered decades ago and the TB drug pipeline, notwithstanding recent additions like delamanid,^4^ remains thin. Hence there is a pressing need to develop new drugs that kill both replicating and phenotypically drug-tolerant nonreplicating mycobacteria.

In recent years, there is a growing interest in targeting mycobacterial energetics due to its pivotal role in energy generation for various cellular processes.^5–7^ In line with this idea, targeting bacterial membrane function is a compelling strategy for several reasons. Firstly, the bacterial membrane provides selective permeability for cellular homeostasis and energy transduction, irrespective of the metabolic status of the cell. Secondly, the membrane contains nearly a third of the proteins in a cell and is the site for processes such as active transport of nutrients and wastes, bacterial respiration and the establishment of the proton motive force (PMF) in association with respiratory enzymes, which are critical for bacterial survival and reproduction. Thirdly, membrane-targeting agents may interact with multiple targets in the membrane, thus greatly diminishing the likelihood of bacteria acquiring resistance to these agents.^8^ The feasibility of this approach has been corroborated by various membrane-active compounds that have antimycobacterial activities.^9–13^

**Fig. 1:**
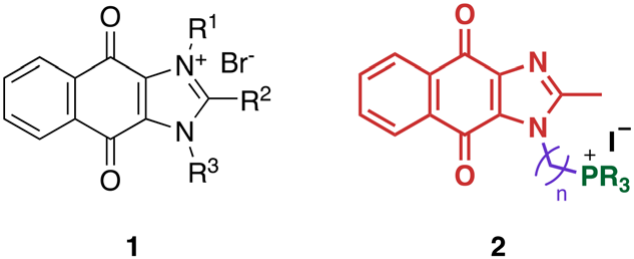
General structure of dioxonaphthoimidazoliums 1, which generates ROS upon reduction by NDH-2. The general structure of trialkylphosphonium naphthoquinoneimidazole 2 is shown with the possible sites of modification highlighted in different colors: green, trialkylphosphonium; purple, aliphatic linker; red, naphthoquinoneimidazole scaffold.

We recently reported the development of redox-cycling dioxonaphthoimidazoliums as potent antimycobacterial agents (1, Fig. 1) whose prominent features include optimal lipophilicity introduced by alkyl substituents (clog *P* = 2.0–2.5) to maximize interaction with the bacterial membrane and a dioxonaphthoimidazolium core activated by a positive charge on the imidazolium nitrogen, which enables sustained generation of reactive oxygen species (ROS) inside bacteria upon reduction by type II NADH dehydrogenase (NDH2).^14^ The most active compounds exhibited submicromolar inhibitory and bactericidal activity against both *M. bovis* BCG and *M. tb*, particularly against nutrient-deprived *M. tb*, which is an advantage over most first-line TB drugs.^15^ The proximal positive charge was posited to reduce the redox potential of the quinone moiety due to its electron-withdrawing nature, which is consistent with our observation that the neutral naphthoquinoneimidazoles were significantly less active.^14^

However, the effects of a distal positive charge on antimycobacterial activity have yet to be established.

Since lipophilicity was an important factor for activities of 1 and its analogs, we hypothesized that a lipophilic trialkylphosphonium cation could be used to furnish a distal positive charge on the scaffold. Earlier studies have shown that the triphenylphosphonium (TPP) cation is a mitochondria-targeting chemotype^16–19^ and grafting TPP to antimycobacterial agents like phenothiazines have resulted in significant improvements in activity, likely due to enhanced localization in the mycobacterial membrane.^12^ Hence in this study, we asked whether the distal positive charge would cause activation of the quinone group as observed in 1, and if it would exhibit additional membrane-targeting properties similar to previous examples. We sought to answer these by attaching a trialkylphosphonium moiety to the naphthoquinoneimidazole scaffold *via* an alkyl linker (2, Fig. 1).

## Results and discussion

### Chemistry

**Fig. 2:**
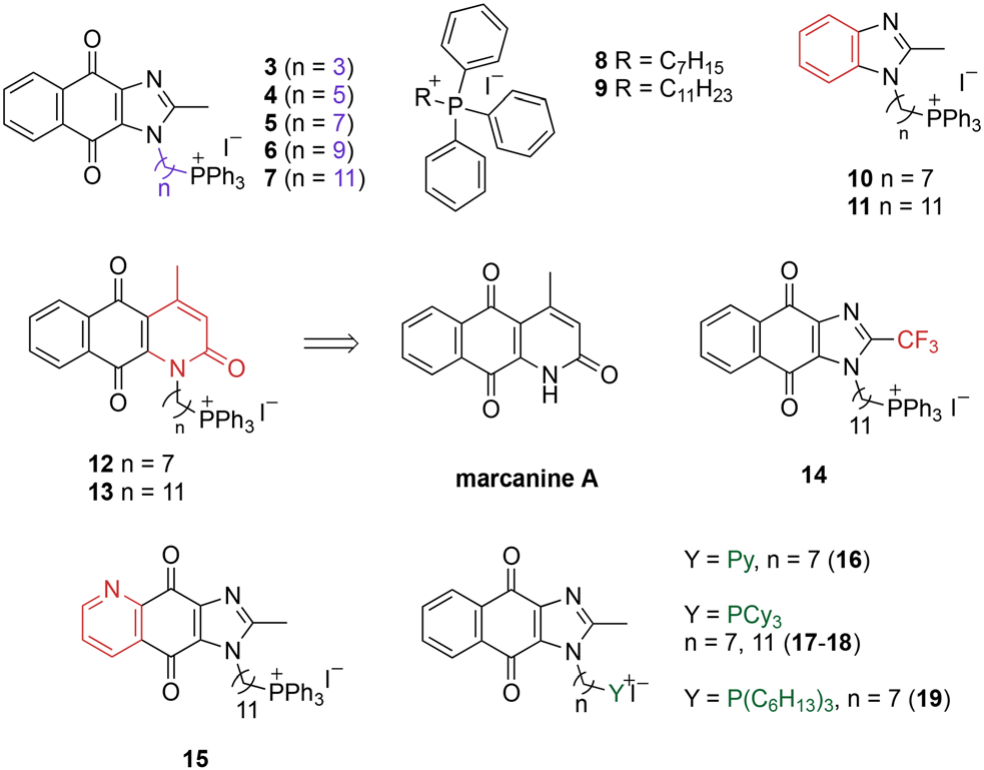
Compounds synthesized for the structure–activity relationship study.

A structure–activity relationship study was carried out to assess the contribution of each part of the scaffold to activity (Fig. 2), starting with linker length. Building on previous reports on TPP-containing analogs with short alkyl linkers (*n* = 3 or 5), a series of analogs with varying linker lengths were synthesized to include longer chains (3–7, *n* = 3–11).^12,13^ Henceforth, the medium and long alkyl linkers (*n* = 7 and *n* = 11) were chosen as representative lengths for modifications on various parts as follows: the naphthoquinoneimidazole scaffold was removed (8–9), replaced with benzimidazole to investigate the effects of the quinone moiety (10–11) and with marcanine A, an antiplasmodial natural product^20,21^ that is structurally similar to naphthoquinoneimidazole (12–13). The methyl substituent on the imidazole was replaced with a trifluoromethyl group (14) and a nitrogen was incorporated into the phenyl ring to afford a quinolinedione (15). Lastly, the triphenylphosphonium substituent was changed to pyridinium and other trialkylphosphonium groups (16–19).

**Scheme 1:**
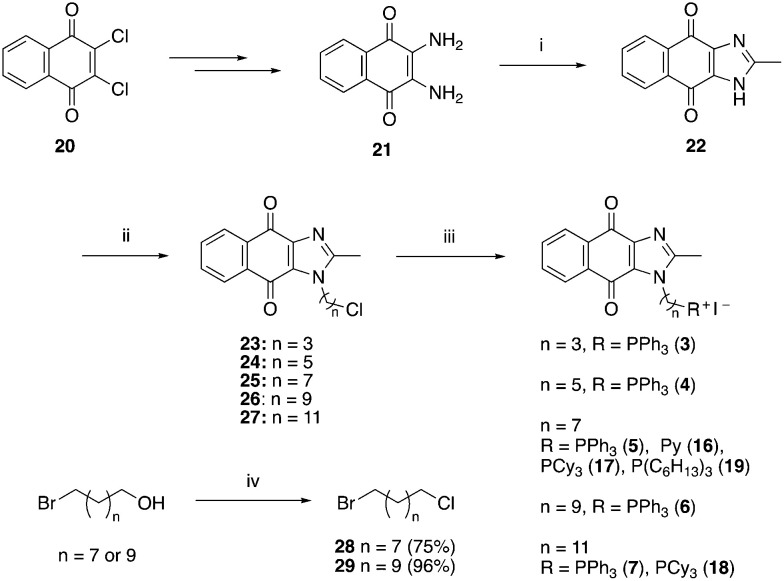
Reagents and conditions: (i) CH_3_COOH, reflux, o/n, 39%; (ii) NaH, Br(CH_2_)_*n*_Cl, DMF, 0 °C – RT, o/n, 43–64%; (iii) refer to Table 1, (iv) SOCl_2_, reflux, 1 h.

**Scheme 2:**
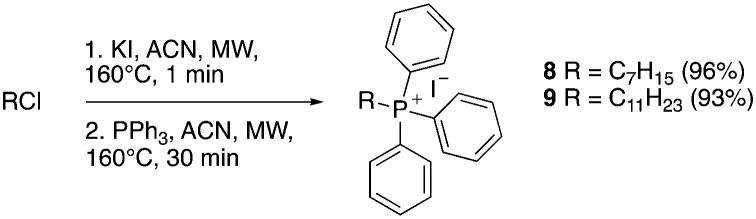


**Scheme 3:**
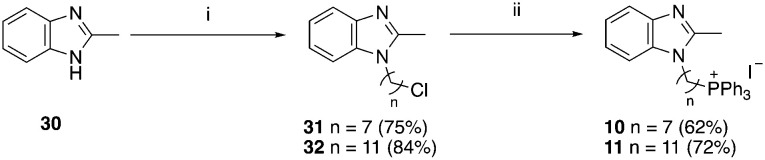
Reagents and conditions: (i) Br(CH_2_)_*n*_Cl, NaH, DMF, 0 °C to RT, o/n, (ii) KI, ACN, reflux 30 min, then PPh_3_, ACN, reflux, 3 d.

**Scheme 4:**
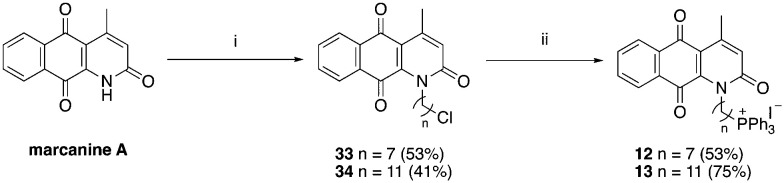
Reagents and conditions: (i) Br(CH_2_)_*n*_Cl, NaH, DMF, 0 °C to RT, o/n, (ii) KI, ACN, reflux 30 min, then PPh_3_, ACN, reflux, 3 d.

**Scheme 5:**
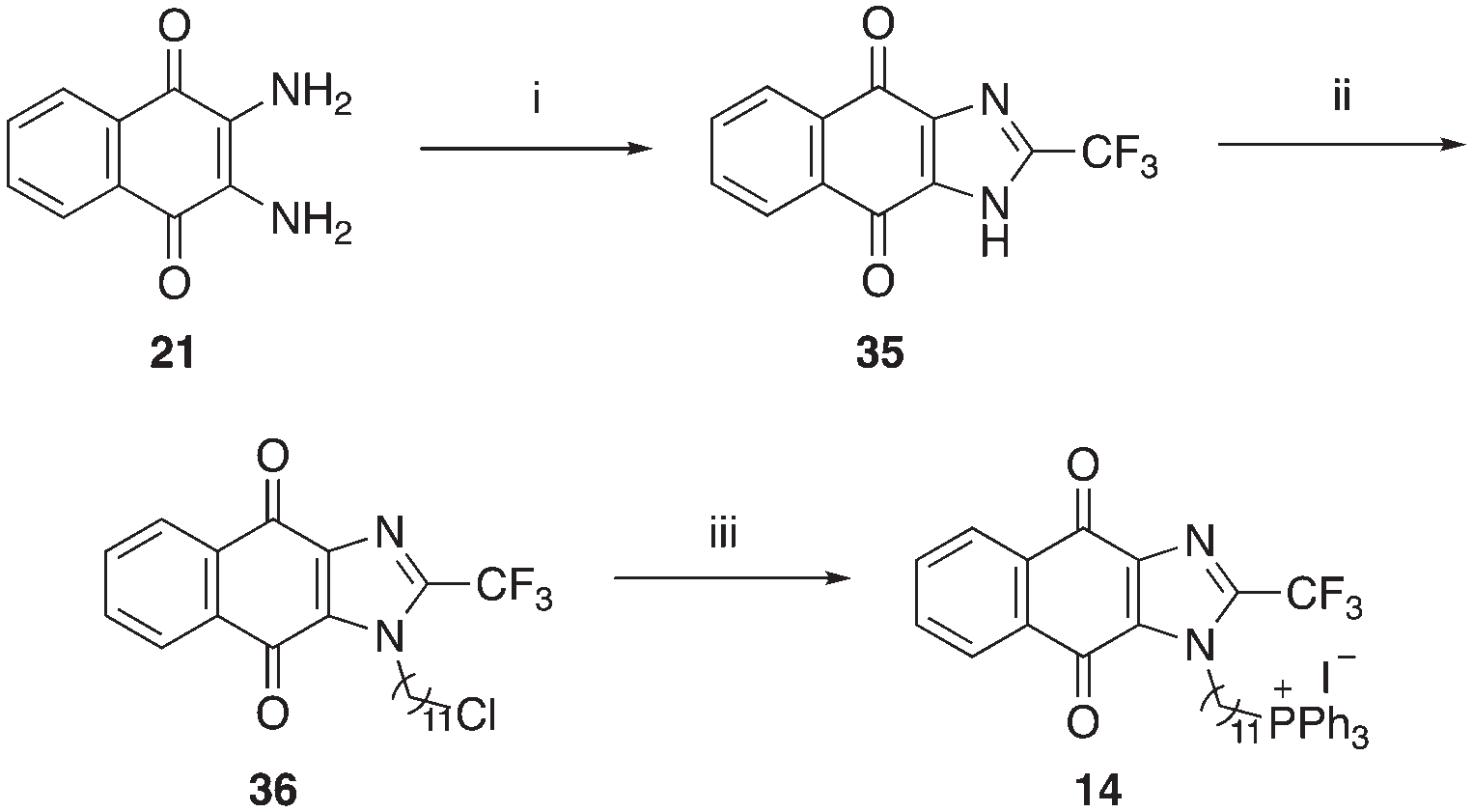
Reagents and conditions: (i) TFA, reflux, o/n, 88%, (ii) Br(CH_2_)_11_Cl, NaH, DMF, 0 °C to RT, 49%, (iii) KI, PPh_3_, ACN, reflux, 5 d, 60%.

**Scheme 6:**
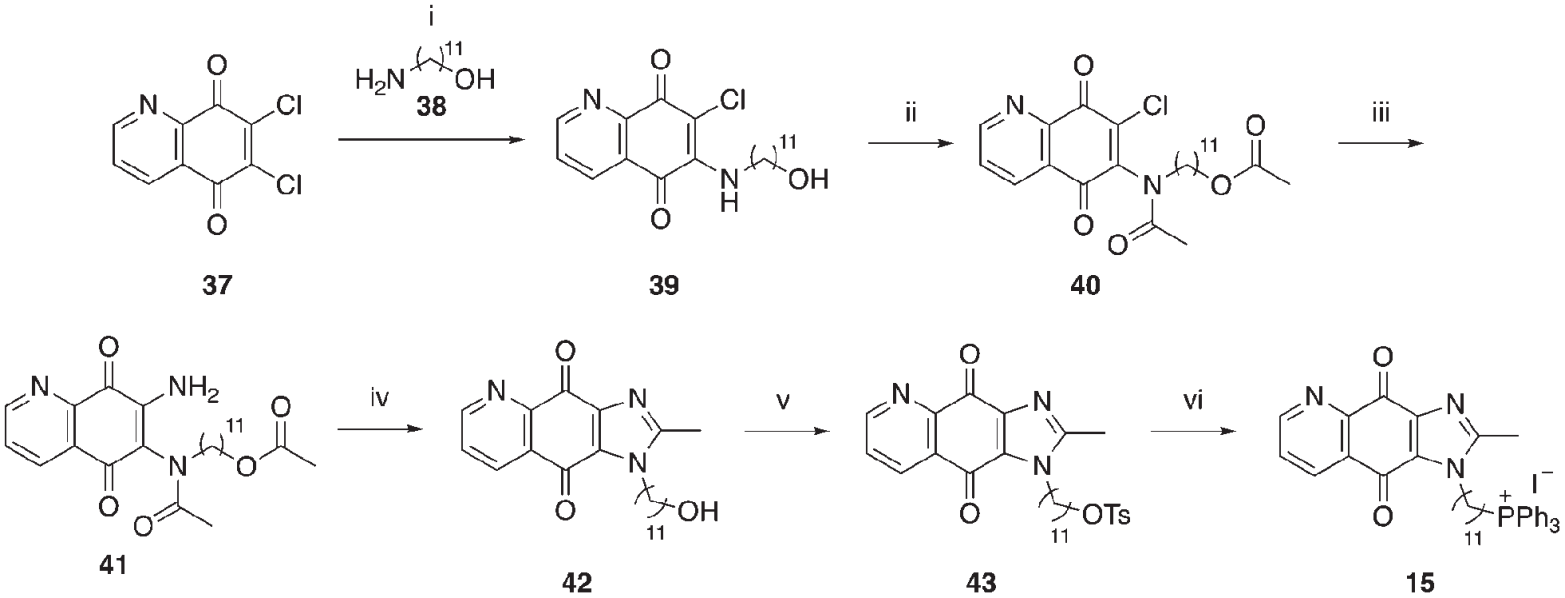
Reagents and conditions: (i) 38, EtOH, RT, 2 h, 49%, (ii) Ac_2_O, conc. H_2_SO_4_ (cat.), RT, 2 h, 84%, (iii) 7 N NH_3_ in MeOH, ACN, 45 °C, 2 h, 54%, (iv) 48% HBr, 1 : 1 EtOH/EtOAc, reflux, o/n, 60%, (v) TsCl, TEA, DCM, RT, 24 h, 60%, (vi) KI, PPh_3_, ACN, MW, 160 °C, 2 h, 70%.

The syntheses of 3–19 are shown in Schemes 1–6. Attachment of the trialkylphosphine group to the haloalkyl linker under reflux condition took 3 days to complete and provided 3 in good yield (Table 1, entry 1). Optimization of the reaction by sequential addition of the reagents improved neither the product yield nor the reaction time (Table 1, entry 2); hence, we attempted microwave-assisted synthesis, which significantly shortened the reaction time to one hour and improved the yield to 98% (Table 1, entry 3).

**Table 1:** Summary of reaction conditions attempted during optimization of the final step to synthesize 3

No.	Reaction conditions	Yield
1	KI (3 eq.), PR_3_ (3 eq.) and precursor (1 eq.), reflux (3 days)	87%
2	a. Reflux KI (3 eq.) and precursor (1 eq.) for 1 h	84%
	b. Add PR_3_ (3 eq.), reflux (4 days)	
3	a. KI (3 eq.) and precursor (1 eq.), MW at 160 °C for 1 min	98%
	b. Add PR_3_ (3 eq.), MW at 160 °C (1 h)	

### Structure–activity relationship

The purities of compounds 3–19 were determined on reversed phase HPLC using two different wavelengths and mobile phases and found to have purities of ≥95% (Table S1†). The compounds were then tested against the *M. tb* surrogate organism *M. bovis* BCG to determine the minimum concentration that inhibits 50% and 90% of bacterial growth (MIC_50_ and MIC_90_). Due to the method used (broth dilution with twofold serial dilution), only differences in MIC that exceeded twofold were considered significant.

Variation of linker length (3–7, Table 2) showed a positive correlation between linker length and MIC_90_ as a measure of activity. Overall, linker extension from three to eleven carbons resulted in a sevenfold increase in activity. While the variation in activity was limited, analogs with shorter linkers (*n* = 3 and 5) exhibited weaker activity than the longer analogs. Subsequent modifications on the scaffold thus focused on longer linkers (*n* = 7, 9, 11).

**Table 2:** Summary of biological activities of test compounds

Compound	Structure	Clog *P*^a^	*M. bovis* BCG	
			MIC_50_^b^	MIC_90_^b^
3	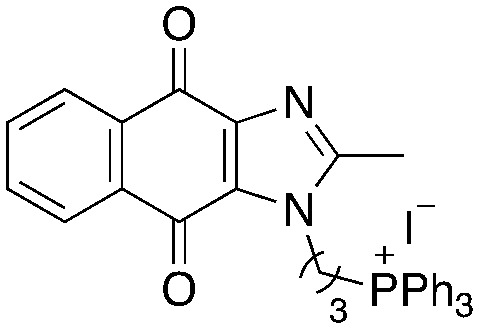	7.17	4.0 (4.0, 4.0)	5.6 (5.8, 5.5)
4	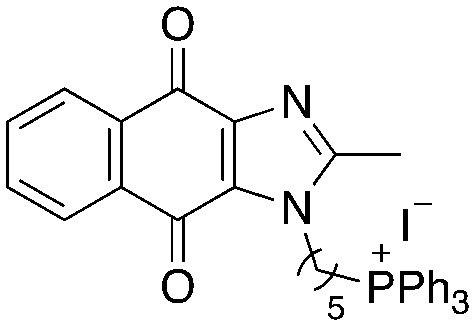	8.05	2.4 (2.6, 2.2)	4.6 (4.6, 4.6)
5	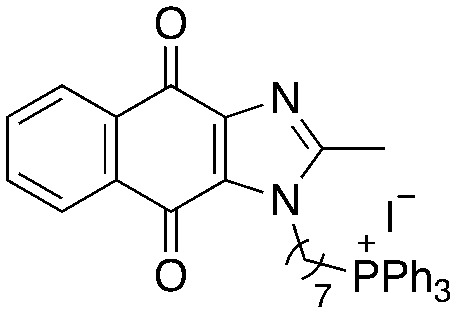	9.10	1.2 (1.2, 1.2)	2.3 (2.4, 2.2)
6	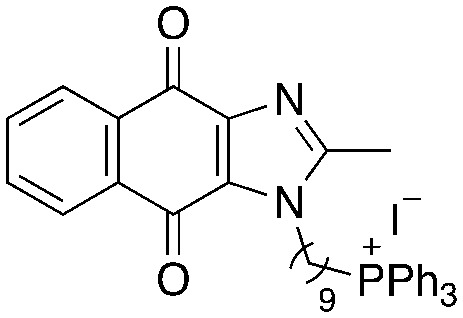	10.16	0.59 (0.6, 0.58)	1 (1, 1)
7	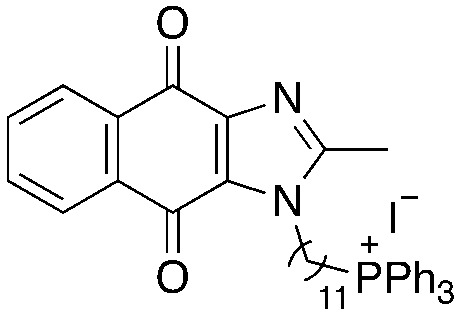	11.22	0.5 (0.6, 0.5)	0.8 (0.9, 0.7)
8	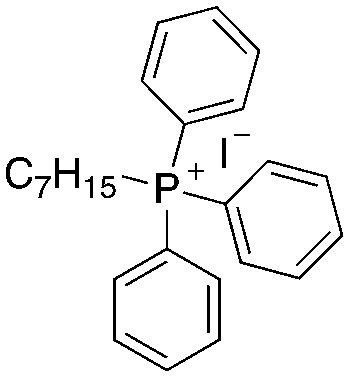	9.31	5.5 (6.7, 4.4)	10.5 (10.2, 10.8)
9	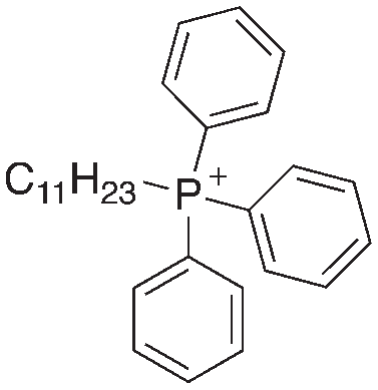	11.42	2.1 (2, 2.2)	2.8 (2.7, 2.8)
10	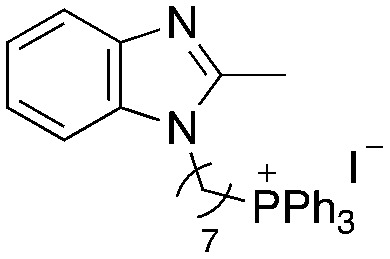	9.66	3.8 (3.8, 3.8)	5.6 (5.7, 5.6)
11	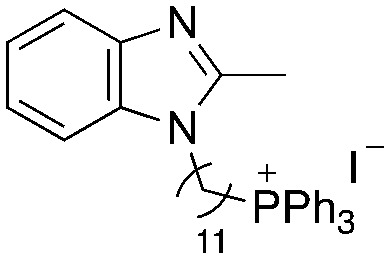	11.78	0.9 (0.9, 1.0)	1.4 (1.4, 1.4)
12	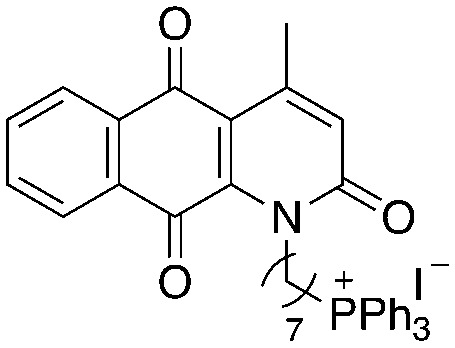	9.53	1 (1.05, 1)	1.4 (1.4, 1.4)
13	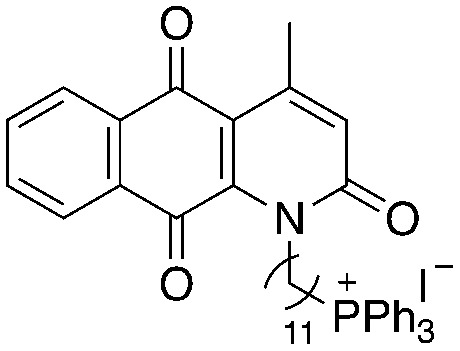	11.65	3.8 (3.6, 4.05)	5.7 (5.5, 5.9)
14	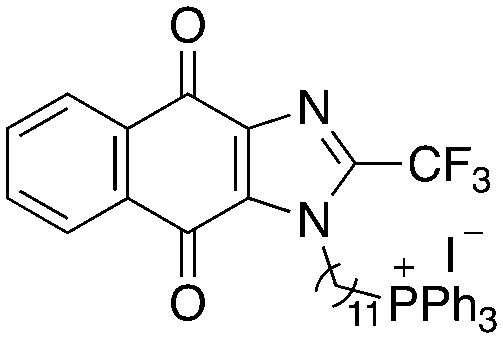	11.83	1.8 (1.7, 1.9)	2.8 (2.7, 2.8)
15	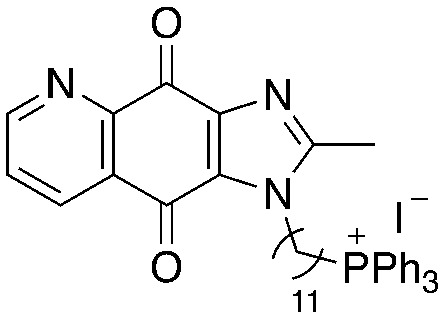	9.72	0.65 (0.6, 0.7)	1.2 (1.2, 1.3)
16	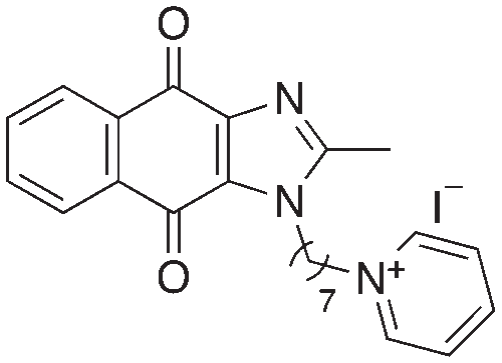	−1.23	28.7 (32.5, 25.0)	48.5 (48.0, 49.0)
17	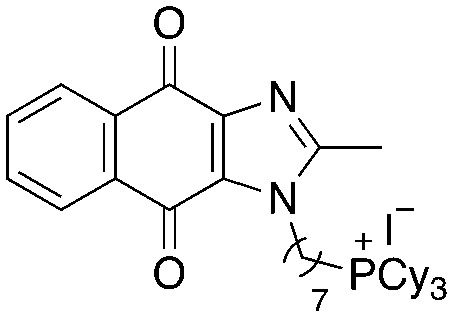	4.91	1.3 (1.3, 1.3)	2.5 (2.6, 2.5)
18	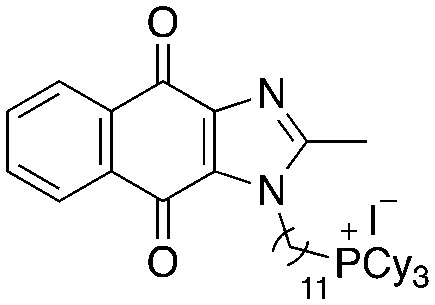	7.02	0.6 (0.6, 0.6)	0.9 (1.0, 0.8)
19	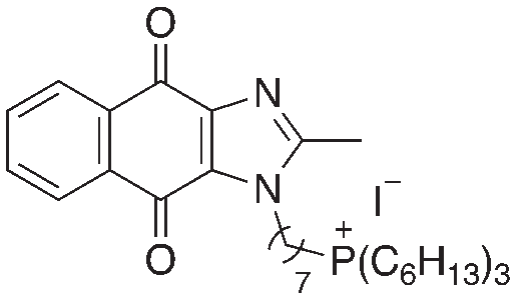	11.81	0.7 (0.7, 0.7)	1.4 (1.4, 1.4)

a Clog *P* values were estimated using ChemDraw Professional 16.0.

b Minimum inhibitory concentration (in μM) required to reduce growth by 50% (MIC_50_) or 90% (MIC_90_) compared to untreated controls.

While keeping the linker length and phosphonium substituent constant, we observed that removing the naphthoquinoneimidazole scaffold (8–9) caused more than twofold decrease in activity. The similar clog *P* values of 5 and 8 (9.10 and 9.31) as well as 6 and 9 (11.22 and 11.42) signify that the activity was not caused by nonspecific effects arising from high lipophilicity. Meanwhile, removal of the quinone moiety (10–11) only resulted in a modest twofold decrease in activity, while changing the scaffold to marcanine A (12–13), replacing the methyl substituent with a trifluoromethyl group (14) and introducing a nitrogen (15) did not improve activity.

Next, we attempted to improve the inhibitory activity against *M. bovis* BCG by replacing TPP with pyridinium (16, Table 2), which is also a delocalized cation. However, this resulted in a significant loss of activity which may be attributed to its less bulky and less lipophilic character. Lastly, varying the phosphonium substituents to non-aromatic cyclic (17–18) or acyclic (19) groups also did not give an improvement in the inhibitory activity.

In view of the trend in inhibitory activity, we focused our subsequent studies on analogs with submicromolar potency (MIC_90_ ≤ 1 μM), namely compounds 6, 7, and 18. Evaluation of their cytotoxicity against Vero E6 cells as a representative mammalian cell line (Tables 3 and S2†) showed that compounds 6 and 7 exhibited adequate selectivity, whereas compound 18 was not sufficiently selective (the selectivity index SI, ratio of IC_50_ to MIC_50_, should be ≥10 to be considered sufficiently selective).^22^ The growth inhibitory activities of 6 and 7 were then confirmed on the pathogenic organism *M. tb* H37Rv. Encouragingly, both actives also exhibited potent inhibitory activities against *M. tb* H37Rv and good aqueous solubility (Table 4).

**Table 3:** Cytotoxicity evaluation of 6, 7, and 18. IC_50_, concentration required to reduce growth of Vero E6 (African green monkey kidney epithelial cells) by 50% compared to untreated controls, are averages of three separate determinations

	6	7	18
Vero E6 IC_50_ (μM)	12.4 (± 0.1)	5.6 (± 0.2)	4.1 (± 0.3)
SI (IC_50 Vero_/MIC_50 *M. bovis* BCG_)	21	11	7

**Table 4:** Summarized biological evaluation of active compounds 6 and 7

		6	7
*M. bovis* BCG	MIC_50_^a^	0.58 (0.6, 0.59)	0.55 (0.6, 0.5)
	MIC_90_^a^	1 (1, 1)	0.8 (0.9, 0.7)
	MBC_90_^b^	1.6 (1.6, 1.6)	1.6 (1.6, 1.6)
	MBC_99_^b^	3.1 (3.1, 3.1)	3.1 (3.1, 3.1)
	MBC_99.9_^b^	6.2 (6.2, 6.2)	6.2 (6.2, 6.2)
*M. tb* H37Rv	MIC_50_^a^	0.37 (0.4, 0.34)	1.15 (1.2, 1.1)
	MIC_90_^a^	0.8 (0.75, 0.85)	1.8 (1.5, 2.1)
Vero E6	IC_50_^c^	12.4 (± 0.1)	5.6 (± 0.2)
SI (IC_50 Vero_/MIC_50 *M.tb*_)		34	5
Aqueous solubility	μM^d^	105.8	27.0

a Minimum inhibitory concentration (in μM) required to reduce growth by 50% (MIC_50_) or 90% (MIC_90_) compared to untreated controls.

b Minimum bactericidal concentration (in μM) required to kill 90%, 99%, and 99.9% of bacteria.

c Concentration (in μM) required to reduce growth of Vero E6 (African Green Monkey kidney epithelial cells) by 50% compared to untreated controls.

d Determined at 25 °C, pH 7.4, 24 h agitation. All values are averages of two or more separate determinations.

These promising preliminary results warranted further characterization of bactericidal activity and elucidation of the mode of action. Specifically, there is a need to ascertain the effects of the naphthoquinoneimidazole and TPP groups on the compound's activity. To this end, studies on the mode of action focused on experiments to establish the redox cycling capability of naphthoquinoneimidazole as well as the membrane effects of the TPP moiety.

### Bactericidal activity against *M. bovis* BCG

**Fig. 3:**
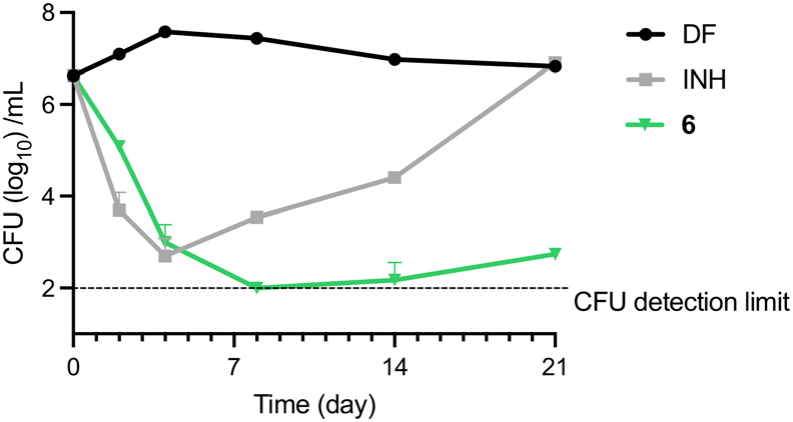
Time-kill kinetics of 6. Mid-log phase *M. bovis* BCG diluted to OD_600_ = 0.1 was treated with test compounds at 4× MIC_90_ (6 = 5 μM, INH = 15 μM) and incubated at 37 °C with shaking. Diluted cultures were plated onto 7H10 agar plates for CFU enumeration at indicated time points. Experiment was repeated once yielding the same results. Result shown was from one representative experiment.

*M. bovis* BCG cultures were treated with various concentrations of 6 for 5 days, after which aliquots from representative concentrations were diluted and plated onto 7H10 agar plates for enumeration of colony forming units (CFU). All the bactericidal concentrations up to MBC_99.9_ fell in the low micromolar range (Table 3). To determine if the bactericidal effect was time-dependent, *M. bovis* BCG was treated with 6 and isoniazid (INH) at 4× MIC_90_ and assessed for 21 days to quantify the number of remaining CFU. Treatment with 6 resulted in a steady decrease of CFU up to day 8, after which the CFU levels remained near the detection limit (Fig. 3).

Meanwhile, consistent with previous observations, the control group treated with INH showed a rapid decrease in CFU in the first two time points, after which the number of cultures dramatically increased due to emergence of resistant mutants.^23^

### Effect of actives on reactive oxygen species (ROS) generation and activation of NADH oxidation

**Fig. 4:**
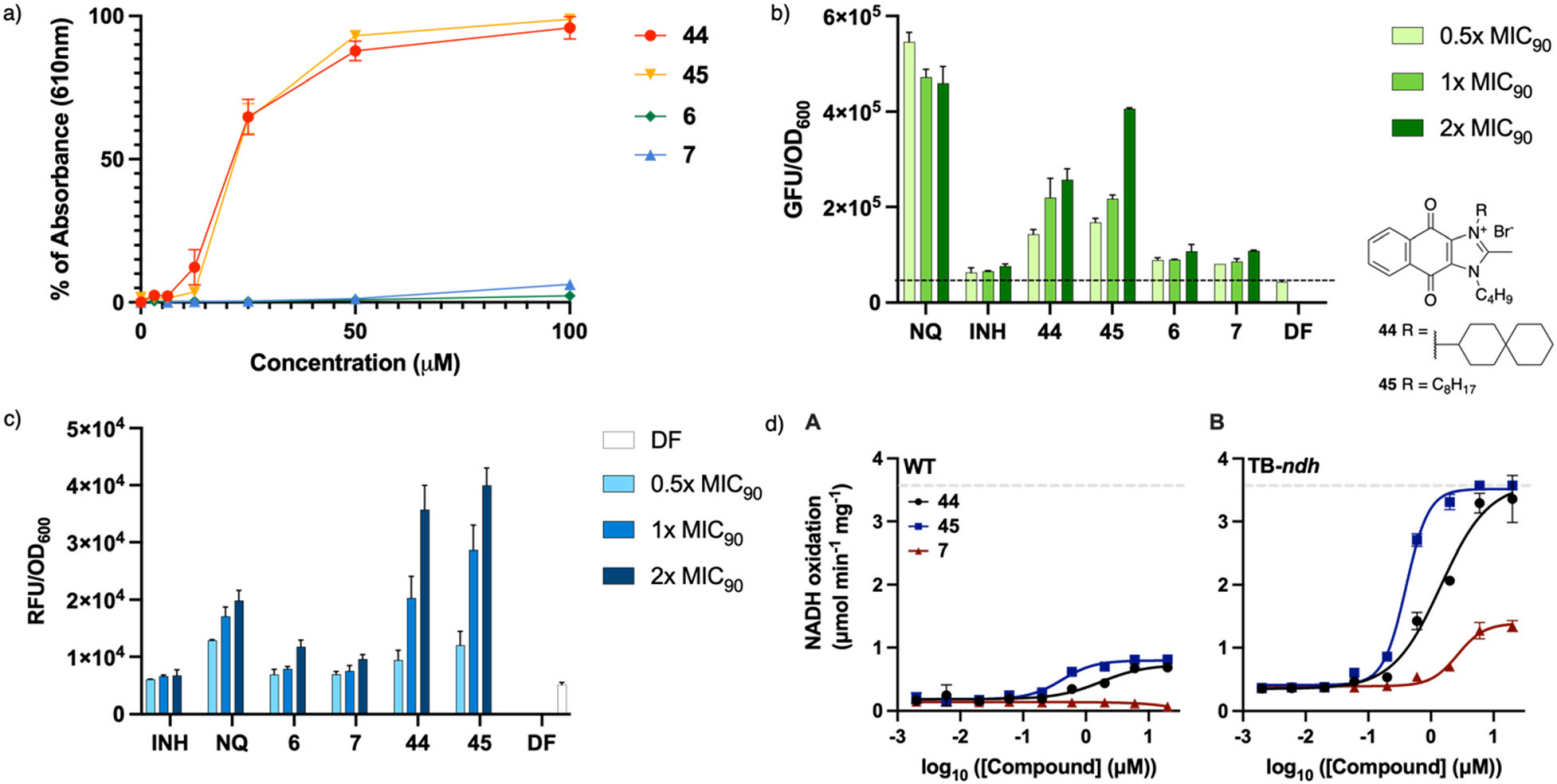
Redox effects of 6 and 7. 1,4-Naphthoquinone (NQ, MIC_90_ = 100 μM) and compounds 44–45 (MIC_90_ = 0.32 μM) were used as positive controls, while INH (MIC_90_ = 3.7 μM) was used as negative control. a) Dose response curves for the HRP assay. Drug-free samples were taken as 0% absorbance. Measurement was carried out in two independent experiments with three technical replicates for each compound. Results shown are from representative experiments. (b) Measured relative green fluorescence unit (GFU) of CellROX green normalized to OD_600_ upon generation of ROS. Mid-log phase *M. bovis* BCG was diluted to OD_600_ = 0.3, after which the culture was treated with test compounds for 1.5 hours and incubated at 37 °C, followed by treatment with dye for 30 min. (c) Absence of induction of the p*furA* gene promoter by 6 and 7. The modified *M. bovis* BCG strain was grown to mid-log phase and diluted to OD_600_ = 0.4 prior to treatment with four different concentrations of test compounds for 24 hours at 37 °C. Experiments were performed at least twice independently. Results shown are from representative experiments. (d) Absence of activation of NADH oxidation by 7. Inverted membrane vesicles of *M. smegmatis mc*^2^4517 A) WT or B) TB-*ndh*, which harbors an overexpression plasmid of the *M. tb ndh* gene, were treated with the indicated compounds. Dashed line represents the upper detection limit. Error bars represent standard deviation from triplicate measurements.

To establish the effect of the distal positive charge on the redox cycling activity of actives, measurement of ROS generation *in vitro* was carried out using the horseradish peroxidase-phenol red assay designed to detect hydrogen peroxide (Fig. 4a).^14^ Earlier studies on dioxonaphthoimidazoliums showed that the compound's activity was dependent on the activation of the redox cycling quinone by the positively charged scaffold (demonstrated by 44–45), while the neutral scaffold exhibited no ROS generation.^14^ Unlike 44 and 45, 6 and 7 showed negligible H_2_O_2_ generation in the presence of horseradish peroxidase (Fig. 4a). To confirm that this phenomenon also occurs within the mycobacteria, we monitored ROS production in treated cultures over a range of concentrations (0.5× to 2× MIC_90_) using a commercial kit (CellROX™ Green Reagent, Invitrogen). Likewise, we found dose-dependent ROS formation in cultures treated with 44 and 45 but negligible levels of ROS in cultures treated with 6 and 7 (Fig. 4b). The absence of ROS generation indicates that the distal positive charge did not increase the propensity of the naphthoquinoneimidazole to redox cycle, which might suggest that it had no effect on the reduction potential of the quinone group.

To establish whether the actives cause oxidative stress, we monitored the fluorescence emitted by a strain of *M. bovis* BCG in which the red fluorescence protein (RFP) expression is controlled by the oxidative stress-inducible gene promoter p*furA*.^14^ An increase in the fluorescent signal indicates an actively transcribing p*furA,* and by extrapolation, the presence of oxidative stress. As shown in Fig. 4c, compounds 44 and 45 elicited a discernible dose-dependent increase in the fluorescence signal, signaling activation of p*furA*, but this profile was not observed in cultures exposed to the non-redox cycling actives 6 and 7. This suggests that perturbation of mycobacterial redox equilibrium is markedly diminished when the positive charge is moved from the scaffold to a distal location, such that there is negligible response from the oxidative stress regulon in the organism.

To ascertain if the intracellular redox cycling pathway was involved in the reduction of the naphthoquinoneimidazole, *i.e.* the quinone would be reduced by an electron donor derived from carbon catabolism (*e.g.* NADH) in a reaction catalyzed by membrane-bound primary dehydrogenases such as NDH2, we determined the ability of 6 and 7 to stimulate NADH oxidation in inverted membrane vesicles (IMV) prepared from wild-type (WT) *M. smegmatis mc*^*2*^4517 or a mutant strain of *M. smegmatis mc*^*2*^4517 that overexpresses NDH2 (TB-*ndh*).^21^ As shown in Fig. 4d, 44 activated NADH oxidation in both WT *M. smegmatis* and TB-*ndh* IMV with EC_50_ of 760 and 3740 nM, respectively. The same was observed with 45 (EC_50_ = 200 nM for both WT and TB-*ndh*); however, in contrast, 7 showed negligible activation of NADH oxidation in WT *M. smegmatis* membranes but observable activation in TB-*ndh* (EC_50_ = 1400 nM). It thus appears that the presence of a distal positive charge did not provide an increase of NADH oxidation, thus confirming that the bactericidal activities of the actives are not linked to ROS generation.

### Effect of actives on membrane potential and depletion of ATP

**Fig. 5:**
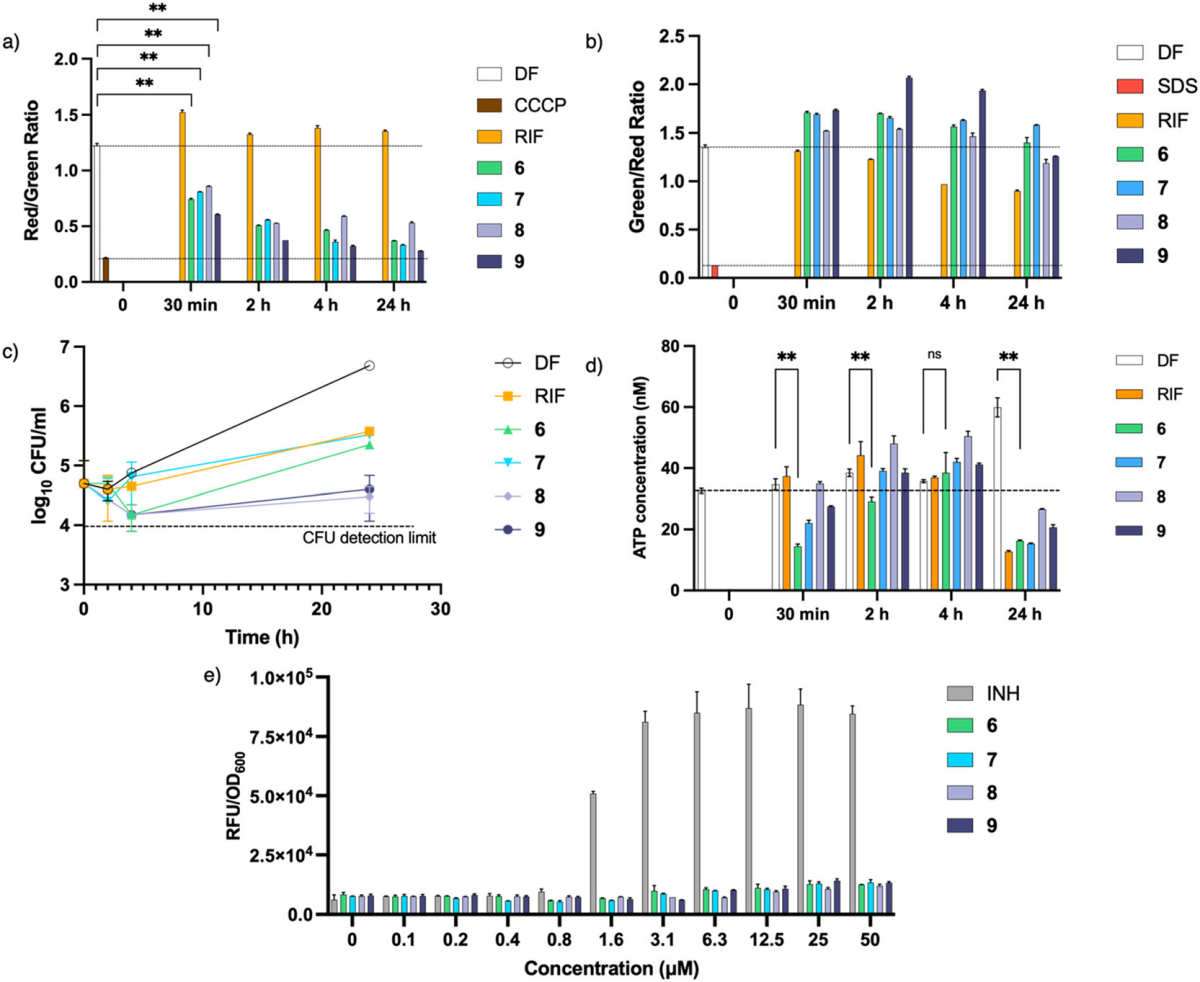
Membrane effects of 6 and 7. Mid-log phase *M. bovis* BCG was diluted to OD_600_ = 0.1 and treated with each compound at 4× MIC_90_. Compounds 8 and 9 were included as controls. (a) Changes in red/green ratio of DiOC_2_(3), which correlates to membrane potential, across different timepoints. (b) Changes in green/red ratio of SYTO®9/propidium iodide, which correlates to membrane integrity, across different timepoints. Green and red fluorescence were recorded at *λ*_ex_ = 488 nm/*λ*_em_ = 530 nm and *λ*_ex_ = 488 nm/*λ*_em_ = 630 nm respectively, and the ratios of the fluorogenic dye was calculated. Drug-free (DF) and RIF (4× MIC_90_ = 0.08 μM) samples were taken as negative controls while CCCP (5 μM) and 5% SDS were used as positive controls. Dashed line indicates ratios of DF and positive controls. (c) CFU of each treated culture over time. (d) Amount of ATP remaining upon treatment based on luminescence measurement. Dashed line reflects the ATP concentration of DF (untreated) sample at the start of the experiment. Means were significantly different than DF control, *P* < 0.01 (**), Student's *t*-test, GraphPad Prism, ver. 9. (e) Absence of induction of the p*iniBAC* gene promoter after treatment with representative test compounds. The modified *M. bovis* BCG strain was grown to mid-log phase and diluted to OD_600_ = 0.4 prior to treatment with test compounds for 24 hours. Error bars depict the standard deviation based on triplicate measurements. Experiments were carried out at least twice independently. Results from representative experiments are shown.

Cationic amphiphilic antimycobacterial agents have been shown to disrupt the structural and functional integrity of the mycobacterial cell membrane.^8–10,24,25^ Such disruptions affect the cell membrane resulting in impaired permeability, loss of membrane potential, induction of cell membrane stress reporter genes and aberration in membrane protein function. To determine if the actives are membrane disruptors, we monitored the time-dependent uptake of DiOC_2_(3) (to detect membrane depolarization) and SYTO®9/propidium iodide (to detect membrane permeabilization) by *M. bovis* BCG cultures treated with 6 and 7 at 4× MIC_90_. To establish the effect of the TPP moiety, compounds 8 and 9 were tested together at the same concentration. Results obtained showed a rapid and sustained loss of membrane potential upon treatment with the actives (Fig. 5a). However, this loss of membrane potential was not accompanied by membrane permeabilization (Fig. 5b). A plot of CFU at representative time points (Fig. 5c) revealed that the loss of membrane potential was not caused by cell death, as CFU count of cultures treated with 6 and 7 remained relatively constant after 24 hours.

Since maintenance of the membrane potential is obligatory for ATP synthesis, one possible consequence of membrane depolarization is depletion of intracellular ATP.^26^ Hence the effect of the actives on ATP synthesis was assessed by measuring the ATP concentration at the same time points. Fig. 5d shows that upon treatment with 6 and 7, there was an initial drop in ATP levels, consistent with the loss in membrane potential observed (Fig. 5a). However, the ATP levels subsequently recovered before showing further decline after 24 hours. Since membrane depolarization was maintained during this period, the rebound in ATP levels may be attributed to metabolic remodelling which leads to enhancement of ATP-generating pathways and reduction of ATP consumption to minimize ATP loss.^27^ Further depletion of ATP was only observed after 24 hours which is consistent with other membrane-depolarizing compounds reported earlier.^11,26,28^

Lastly, we probed the mycobacterial p*iniBAC* promoter which is transcriptionally upregulated when the cell envelope is subjected to stress stimuli elicited by cell wall inhibitors and membrane targeting agents.^29^ To verify induction of the promoter, we monitored the fluorescent signal emitted by *M. bovis* BCG cultures expressing red fluorescent protein (RFP) under control of p*iniBAC* promoter that had been treated with various concentrations of 6, 7 and the positive control INH. As seen from Fig. 5e, there was no increase in fluorescence from cultures treated with 6 and 7. This result together with the absence of membrane permeabilization signifies that 6 and 7 did not induce cell envelope stress. On the other hand, INH, which disrupts mycobacterial cell wall biosynthesis, increased p*iniBAC* promoter activity as seen from the elevated fluorescent signals.

Inclusion of compounds 8 and 9 in all experiments revealed that both compounds behaved similarly to 6 and 7, suggesting that the TPP moiety was responsible for the membrane effects observed. We demonstrated that the inhibitory activities of 8 and 9 are significantly lower than that of 6 and 7; hence, we surmise that the presence of the dioxonaphthoimidazole scaffold might improve the activity of the latter due to the presence of hydrogen bond acceptors that facilitate interactions with the more polar components of the mycobacterial membrane, thereby increasing the affinity of the molecules toward the membrane.

While the high lipophilicity of the actives may raise doubts on their drug-like properties, we showed that compound 6 (solubility at pH 7.4 = 106 μM) has comparable aqueous solubility profile to 44 and 45 (solubility at pH 7.4 = 118 μM). If further characterization of the pharmacokinetic properties turns out to be unfavorable, a drug delivery vehicle may need to be utilized to overcome the lipophilic character. Additional characterization of the consequences of membrane depolarization beyond ATP depletion would also be beneficial. We previously observed that treatment of *M. bovis* BCG with TPP-containing indoles resulted in elongation of bacilli, which was attributed to a defective cell division caused by membrane depolarization; by extrapolation, we expect 6 and 7 to elicit a similar effect.^13^

## Conclusions

This study was envisioned to establish the effect of distal positive charge on the naphthoquinoneimidazole scaffold, which consequently translates to its antimycobacterial activity. While the naphthoquinoneimidazole *per se* is not a redox cycler, a positive charge is posited to reduce the reduction potential and increase its redox-cycling propensity. This was true for the dioxonaphthoimidazoliums 1 where the positive charge located on the imidazolium N resulted in activation of redox cycling and ROS production responsible for its antibacterial activity. However, in this study, we established that when the positive charge is moved to a distal location using a triphenylphosphonium cation, the resulting compounds caused rapid loss of membrane potential and depletion of ATP with diminished redox cycling and ROS-generating properties—essentially exhibiting a switch in their mode of action while maintaining good antibacterial properties, selectivity against mammalian cells, and solubility. Further characterization of physicochemical and pharmacokinetic properties as well as the downstream effects of membrane depolarization will help to ascertain the extent of perturbation of mycobacterial energetics caused by the active compounds.

## Author contributions

M. L. G., K. H., and K. T. F. conceptualized this work. Investigation was carried out by M. L. G., K. T. F., G. A. G., J. P. S., and K. H. K. T. F., M. L. G., Y. L., T.D. contributed to the writing of the manuscript. Review & editing was performed by all authors. M. L. G. and T. D. contributed to funding acquisition. Supervision was performed by M. L. G., Y. L., G. M. C., and T. D. Corresponding authors M. L. G., Y. L., and T. D. contributed equally to this work.

## Conflicts of interest

There are no conflicts to declare.

## Supplementary Material

MD-013-D2MD00251E-s001
