# Supplementary material for: Alkyltriphenylphosphonium turns naphthoquinoneimidazoles into potent membrane depolarizers against mycobacteria
Source: RSC Med Chem. 2022 Oct 13;13(12):1605–13. doi: 10.1039/d2md00251e (PMC9749938; doi:10.1039/d2md00251e)
Supplement: MD-013-D2MD00251E-s001 [file MD-013-D2MD00251E-s001.pdf]

Electronic Supplementary Material (ESI) for RSC Medicinal Chemistry.
This journal is © The Royal Society of Chemistry 2022

Alkyltriphenylphosphonium Turns Naphthoquinoneimidazoles Into Potent Membrane Depolarizers Against *Mycobacteria*

Kevin Timothy Fridianto, Gregory Adrian Gunawan, Kiel Hards, Jickky Palmae Sarathy, Gregory M. Cook, Thomas Dick,* Mei-Lin Go,* Yulin Lam*

Supporting Information

Table of Contents

1. General Chemistry (pg. 3)

2. Synthesis and characterization of intermediates (pg. 3)

3. Synthesis and characterization of final compounds (pg. 8)

4. Synthesis of marcanine A (pg. 12)

5. Purity determination of final compounds (pg. 14)

6. NMR spectra of final compounds and intermediates (pg. 15)

7. HRMS spectra of **6** and **7** (pg. 49)

8. HPLC chromatograms of **6** and **7** (pg. 51)

9. General Biology (pg. 55)

10. Cytotoxicity determination on Vero E6 cells (pg. 57)

11. Plasmid and primers used in the construction of *M. bovis* BCG-p*iniBAC*-RFP reporter system (pg. 58)

12. Plasmid and primers used in the construction of *M. bovis* BCG-p*furA*-RFP reporter system (pg. 58)

13. References (pg. 59)

1. General Chemistry

Commercially available reagents of synthetic grade (or better) were used without further purification. Air-sensitive experiments were carried out under N_2 atmosphere in oven-dried glassware fitted with rubber septa. Microwave-assisted reactions were conducted on a Monowave 400 microwave reactor (Anton Paar, Austria). Reactions were monitored by thin- layer chromatography (TLC) on aluminum-backed sheets coated with silica gel (Merck 60 F254, 250 µm thickness). Compounds were purified by column chromatography on silica gel 60 (230−400 mesh, Merck), and compounds were detected on TLC sheets by ultraviolet light or with appropriate stains (phosphomolybdic acid (PMA), potassium permanganate). ^{1}H and ^{13}C NMR spectra were recorded in $CDCl_3$, CD_3OD, DMSO-d$_6$, or acetone-d$_6$ on a Bruker Avance 300, 400, or 500 MHz spectrometer (Bruker Corp., MS). Chemical shifts were reported in ppm on the δ scale using residual protiosolvent signals (^{1}H NMR: $CDCl_3$ δ 7.26, DMSO-d$_6$ δ 2.50, CD_3OD-d$_4$ δ 3.31, acetone-d$_6$ δ 2.05; ^{13}C NMR: $CDCl_3$ δ 77.0, DMSO-d$_6$ δ 39.5, CD3OD-d$_4$ δ 49.0, acetone-d$_6$ δ 206.7) as internal references. Coupling constants (J) were reported in Hz, and splitting patterns as singlet (s), broad singlet (br s), doublet (d), doublet of doublets (dd), triplet (t), triplet of doublets (td), quartet (q), quintet (quint), sextet (sext), or multiplet (m). Nominal mass spectra were captured on an LCQ Fleet Ion Trap LC-MS system (Thermo Scientific) by ESI or EI, run in either positive or negative ionization mode. High-resolution mass spectra were recorded on a Bruker MicroTOF-QII mass spectrometer (Bruker Corp., MA) by ESI (positive or negative ionization mode). Purities of final compounds were analyzed by reverse-phase high-performance liquid chromatography (HPLC) (Shimadzu Nexera SR, Japan) and found to be >95% pure.

2. Synthesis and characterization of intermediates

The synthetic route and procedures by Li *et al.* was modified and employed.[S1]

General procedure for alkylation of the naphthoimidazolediones **22** and **35** (Procedure A)

Compound **22** or **35** was reacted with NaH (60% dispersion in mineral oil, 2 eq.) in anhydrous DMF (3 mL) at 0°C for 30 minutes under N_2. The corresponding bromochloroalkane (1.3 eq.) was added to the reaction mixture and allowed to warm to room temperature and stirred overnight under N_2. Upon completion, the mixture was quenched with cold water at 0°C. The crude product was extracted with DCM (4 × 20 mL), washed with water (4 × 20 mL) and brine (1 × 20 mL). The organic layers were combined and dried over anhydrous $MgSO_4$, filtered and concentrated under reduced pressure to obtain crude residues, which were purified by column chromatography using DCM:EtOAc (16:1 – 1:1) to obtain the desired products as a yellow solid. This procedure is referred to as Procedure A.

General procedure for nucleophilic substitution to attach trialkylphosphine (Procedure B)

KI (3 eq.) and the desired linker starting material were dissolved in ACN (5 mL) and subjected to microwave (MW) irradiation to a temperature of 160°C for 1 min. The corresponding nucleophile (3 eq.) was then added to the reaction mixture and subjected to microwave (MW) irradiation to a temperature of 160°C for a further 0.5 – 2 h. Upon completion of the reaction, the mixture was filtered. The filtrate was then loaded onto silica and purified by column chromatography using DCM:MeOH (49:1 – 9:1) to yield a yellow solid/oil as the product. This procedure is referred to as Procedure B.

2,2'-(1,4-dioxo-1,4-dihydronaphthalene-2,3-diyl)bis(isoindoline-1,3-dione)

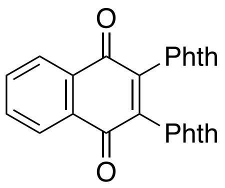

The synthesis procedure by Chesneau *et al.* was employed to give the desired product in 85% yield.[S2] ^{1}H NMR (500 MHz, $CDCl_3$): δ 8.25-8.22 (m, 2H), 7.90-7.83 (m, 6H), 7.78-7.74 (m, 4H). ^{13}C NMR (125 MHz, $CDCl_3$): δ 178.3, 164.7, 139.2, 136.6, 134.7, 131.8, 131.3, 127.5, 124.4. +ESI-MS calcd. for $C_{26}H_{13}N_2O_6$: 449.08; found: 449.03. Characterization data in agreement with literature.

2,3-Diaminoonaphthoquinone (**21**)

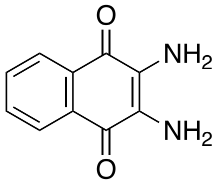

The synthesis procedure by Chesneau *et al.* was employed to give the desired product in 89% yield.[S2] ^{1}H NMR (500 MHz, Acetone-*d*$_6$): δ 7.89-7.85 (m, 2H), 7.64-7.60 (m, 2H), 5.02 (br s, 4H). ^{13}C NMR (125 MHz, Acetone-*d*$_6$): δ 179.8, 133.5, 132.4, 128.7, 125.7. +ESI-MS calcd. for $C_{10}H_9N_2O_2$: 189.07; found: 188.97. Characterization data in agreement with literature.

2-Methyl-1*H*-naphtho[2,3-*d*]imidazole-4,9-dione (**22**) and its 2-trifluoromethyl analog (**35**)

The synthesis procedure by Pan *et al.* was modified and employed with either glacial acetic acid or trifluoroacetic acid. [S3] The mixture was loaded onto silica gel and purified through column chromatography using DCM:MeOH (49:1 – 9:1) before flushing out the product using MeOH:EtOAc (19:1) to yield a yellowish-grey solid as **22** (39%)**.** For **35**, the mixture was dried *in vacuo* and recrystallised using hot MeOH to yield orange crystals (88%).

2-Methyl-1*H*-naphtho[2,3-*d*]imidazole-4,9-dione (**22**)

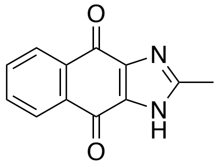

^{1}H NMR (500 MHz, DMSO-*d*$_6$): δ 8.04 (dd, *J* = 5.1, 3.45 Hz, 2H), 7.64-7.61 (dd, *J* = 5.25, 3.5 Hz, 2H), 2.44 (s, 3H). ^{13}C NMR (125 MHz, DMSO-*d*$_6$): δ 180.2, 152.7, 135.0, 133.8, 127.2, 126.2, 14.1. +ESI-MS calcd. for $C_{12}H_9N_2O_2$: 213.07; found: 213.14. Characterization data in agreement with literature.

2-Trifluoromethyl-1*H*-naphtho[2,3-*d*]imidazole-4,9-dione (**35**)

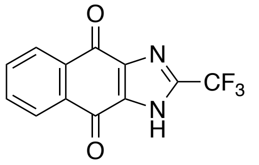

^{1}H NMR (500 MHz, Acetone-*d*$_6$): δ 8.21-8.18 (m, 2H), 7.92-7.88 (m, 2H). ^{13}C NMR (125 MHz, Acetone-*d*$_6$): δ 177.7, 142.1 (q, ^{2}J(C,F) = 36.6 Hz, 1C), 139.9, 135.0, 134.1, 127.5, 119.4 (q, ^{1}J(C,F) = 266.6 Hz, 1C). HRMS (+ESI) calcd. for $C_{12}H_4N_2O_2F_3$: 265.0230; found: 265.0223. Characterization data in agreement with literature.[S4]

N-(3-Chloropropyl)-2-methylnaphtho[2,3-*d*]imidazole-4,9-dione (**23**, $C_{15}H_{13}N_2O_2Cl$)

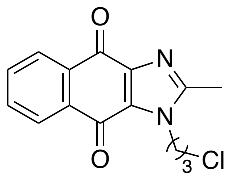

Procedure A was employed with **22** (50 mg, 0.236 mmol) and 1-bromo-3-chloropropane (30 μL, 0.303 mmol) to afford the desired product as yellow solid (29.3 mg, 43%). ^{1}H NMR (500 MHz, $CDCl_3$): δ 8.21-8.17 (m, 1H), 8.09-8.06 (m, 1H), 7.72-7.67 (m, 1H), 4.52 (t, *J* = 7.2 Hz, 2H), 3.62 (t, *J* = 5.98 Hz, 2H), 2.61 (s, 3H), 2.31 (quint, *J* = 6.63 Hz, 2H). ^{13}C NMR (125 MHz, $CDCl_3$): δ 178.8, 176.2, 153.2, 143.4, 133.8, 133.4, 132.9, 132.7, 132.0, 127.0, 126.4, 43.2, 41.4, 32.7, 13.3. HRMS (+ESI) calcd. for $C_{15}H_{14}N_2O_2Cl$: 289.0738; found: 289.0748.

N-(5-Chloropentyl)-2-methylnaphtho[2,3-*d*]imidazole-4,9-dione (**24**, $C_{17}H_{17}N_2O_2Cl$)

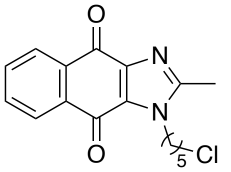

Procedure A was employed with **22** (50 mg, 0.236 mmol) and 1-bromo-5-chloropentane (40 μL, 0.303 mmol) to afford the desired product as yellow solid (40.9 mg, 55%). ^{1}H NMR (500 MHz, $CDCl_3$): δ 8.19-8.17 (m, 1H), 8.08-8.07 (m, 1H), 7.70-7.66 (m, 1H), 4.36 (t, *J* = 7.58 Hz, 2H), 3.54 (t, *J* = 6.43 Hz, 2H), 2.55 (s, 3H), 2.31 (m, 4H), 2.31 (quint, *J* = 7.68 Hz, 2H). ^{13}C NMR (125 MHz, $CDCl_3$): δ 178.9, 176.1, 152.8, 143.2, 133.6, 133.3, 133.0, 132.7, 132.0, 126.9, 126.3, 45.5, 44.5, 31.8, 29.5, 23.8, 13.3. HRMS (+ESI) calcd. for $C_{17}H_{18}N_2O_2Cl$: 317.1051; found: 317.1060.

N-(7-Chloroheptyl)-2-methylnaphtho[2,3-*d*]imidazole-4,9-dione (**25**, $C_{19}H_{21}N_2O_2Cl$)

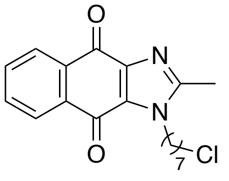

Procedure A was employed with **22** (50 mg, 0.236 mmol) and 1-bromo-7-chloroheptane (50 μL, 0.303mmol) to afford the desired product as yellow solid (52.3 mg, 64%). ^{1}H NMR (400 MHz, $CDCl_3$): δ 8.25-8.21 (m, 1H), 8.14-8.10 (m, 1H), 7.74-7.69 (m, 1H), 4.37 (t, *J* = 7.6 Hz, 2H), 3.52 (t, *J* = 6.62 Hz, 2H), 2.57 (s, 3H), 1.87-1.72 (m, 4H), 1.49-1.36 (m, 6H). ^{13}C NMR (125 MHz, $CDCl_3$): δ 178.9, 176.2, 152.9, 143.0, 133.7, 133.4, 133.1, 132.8, 132.0, 127.0, 126.4, 45.7, 44.9, 32.4, 30.2, 28.4, 26.6, 26.4, 13.3. HRMS (+ESI) calcd. for $C_{19}H_{22}N_2O_2Cl$: 345.1364; found: 345.1369.

1-(9-Chlorononyl)-2-methyl-1*H*-naphtho[2,3-*d*]imidazole-4,9-dione (**26**, $C_{21}H_{25}ClN_2O_2$)

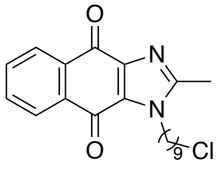

Procedure A was employed with **22** (50 mg, 0.24 mmol) and **28** (70 μL, 0.31 mmol) to afford the desired product as yellow solid (31.1 mg, 35%). ^{1}H NMR (500 MHz, $CDCl_3$): δ 8.21-8.19 (m, 1H), 8.11-8.09 (m, 1H), 7.71-7.67 (m, 2H), 4.37-4.34 (t, *J* = 7.35 Hz, 2H), 3.51-3.49 (t, *J* = 6.7 Hz, 2H), 2.55 (s, 3H), 1.79-1.77 (m, 2H), 1.75-1.71 (quint, *J* = 6.8 Hz, 2H), 1.39-1.29 (m, 2H), 1.57-1.51 (quint, *J* = 6.95 Hz, 2H), 1.47-1.41 (quint, J = 6.8 Hz, 2H), 1.35-1.27 (m, 10H). ^{13}C NMR (125 MHz, $CDCl_3$): δ 178.9, 176.1, 152.9, 143.1, 133.6, 133.3, 133.1, 132.7,

132.0, 126.9, 126.4, 45.8, 45.0, 32.5, 30.3, 29.2, 28.9, 28.7, 26.7, 26.5, 13.4. HRMS (+APCI) calculated for $C_{21}H_{25}ClN_2O_2$: 372.1610; found: 372.1610.

N-(11-Chloroundecyl)-2-methylnaphtho[2,3-*d*]imidazole-4,9-dione (**27**, $C_{23}H_{29}N_2O_2Cl$)

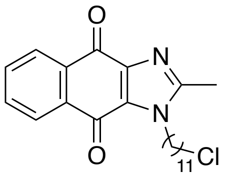

Procedure A was employed with **22** (30 mg, 0.141 mmol) and **29** (60 μL, 0.241 mmol) to afford the desired product as yellow solid (33.3 mg, 59%). ^1H NMR (400 MHz, $CDCl_3$): δ 8.22-8.17 (m, 1H), 8.12-8.07 (m, 1H), 7.71-7.66 (m, 1H), 4.35 (t, *J* = 7.58 Hz, 2H), 3.50 (t, *J* = 6.76 Hz, 2H), 2.55 (s, 3H), 1.82-1.70 (m, 4H), 1.39-1.24 (m, 14H). ^{13}C NMR (100 MHz, $CDCl_3$): δ 178.9, 176.1, 152.9, 143.2, 133.6, 133.3, 133.1, 132.7, 132.0, 126.9, 126.4, 45.8, 45.1, 32.5, 30.3, 29.3, 29.1, 28.7, 26.8, 26.5, 13.3. HRMS (+ESI) calcd. for $C_{23}H_{30}N_2O_2Cl$: 401.1990; found: 401.1980.

General procedure for synthesis of bromochloroalkanes

To a mixture of the corresponding bromoalcohol (2 mmol) and pyridine (5 μL), $SOCl_2$ (2.2 mmol) was added slowly over 30 mins at 0°C. The mixture was then heated under reflux at 90°C for 5 h. The mixture was quenched with water (10 mL) and extracted with DCM (3 × 20 mL). The combined organic layer was washed with $NaHCO_3$ (10 mL), brine (10 mL) and dried with $MgSO_4$. The solution was concentrated and purified through column chromatography using hexane. The product was then dried *in vacuo* to yield a colourless oil.

1-Bromo-9-chlorononane (**28**, $C_9H_{18}BrCl$). The procedure was employed with 9-bromononan-1-ol (0.442 g) to afford the product as a colourless liquid (0.46 g, 96%). ^1H NMR (400 MHz, $CDCl_3$): δ 3.54-3.51 (t, *J* = 6.76 Hz, 2H), 3.42-3.38 (quint, *J* = 6.84 Hz, 2H), 1.88-1.81 (quint, *J* = 6.96 Hz, 2H), 1.79-1.72 (quint, *J* = 6.88 Hz, 2H), 1.45-1.39 (quint, J = 7.8 Hz, 4H), 1.32-1.31 (m, 6H). ^{13}C NMR (100 MHz, $CDCl_3$): δ 45.1, 33.9, 32.7, 32.6, 29.2, 28.7, 28.6, 28.1, 26.8. +ESI-MS calcd. for $C_9H_{18}BrCl$: 241.60; found: 240.03. Characterization data in agreement with literature.[S5]

1-Bromo-11-chloroundecane (**29**, $C_{11}H_{22}BrCl$). The procedure was employed with 11-bromoundecan-1-ol (0.5 g, 2 mmol) to afford the product as a colourless liquid (0.405 g, 75%) that was a mixture with 1,11-dichloroundecane in a 2:1 ratio from the NMR. ^1H NMR (500 MHz, $CDCl_3$): δ 3.53 (t, *J* = 6.7 Hz, 2.67H), 3.40 (quint, *J* = 6.83 Hz, 1.33H), 1.85 (quint, *J* = 7.19 Hz, 1.33H), 1.76 (quint, *J* = 7.13 Hz, 2.67H), 1.43-1.39 (m, 4H), 1.28 (s, 10H). ^{13}C NMR (125 MHz, $CDCl_3$): δ 45.3, 34.2, 34.1, 33.0, 32.8, 29.5, 29.5, 29.0, 28.9, 28.3, 27.0. +ESI-MS calcd. for $C_{11}H_{22}BrCl$: 268.07; found: 268.20. Characterization data in agreement with literature.[S6]

1-(Chloroheptyl)-2-methylbenzimidazole (**31**, $C_{15}H_{21}ClN_2$)

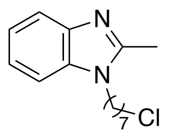

Procedure A was employed with **30** (50 mg, 0.38 mmol) and 1-bromo-7-chloroheptane (80 μL, 0.49 mmol) to afford the desired product as colorless oil (75.5 mg, 75%). ^1H NMR (500 MHz, $CDCl_3$): δ 7.72-7.71 (m, 1H), 7.32-7.29 (m, 1H), 7.27-7.23 (m, 2H), 4.12-4.09 (t, *J* = 7.35 Hz, 2H), 3.55-3.52 (t, *J* = 6.55 Hz, 2H), 2.62 (s, 3H), 1.85-1.81 (quint, *J* = 7.3 Hz, 2H), 1.79-1.74 (quint, *J* = 6.65 Hz, 2H), 1.48-1.36 (m, 6H). ^{13}C NMR (125 MHz, $CDCl_3$): δ 151.3, 142.5, 134.9, 121.9, 121.7, 118.9, 109.1, 44.9, 43.7, 32.3, 29.6, 28.5, 26.7, 26.6, 13.9. HRMS (+ESI) calcd. for $C_{15}H_{22}ClN_2$: 265.1466; found: 265.1469.

1-(Chloroundecyl)-2-methylbenzimidazole (**32**, $C_{19}H_{29}ClN_2$)

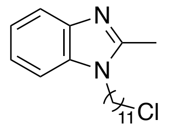

Procedure A was employed with **30** (0.047 g, 0.15 mmol) and **29** (110 μL, 0.44 mmol) to afford the desired product as colorless oil (0.102 mg, 84%). ^1H NMR (500 MHz, $CDCl_3$): δ 7.70-7.66 (m, 1H), 7.30-7.27 (m, 1H), 7.24-7.20 (m, 2H), 4.09-4.07 (t, *J* = 7.35 Hz, 2H), 3.53-3.51 (t, *J* = 6.7 Hz, 2H), 2.60 (s, 3H), 1.82-1.72 (m, 4H), 1.42-1.26 (m, 14H). ^{13}C NMR (125 MHz, $CDCl_3$): δ 151.4, 142.6, 135.1, 121.8, 121.7, 119.0, 109.1, 45.1, 43.9, 32.6, 29.7, 29.4, 29.3, 29.2, 28.8, 26.9, 26.8, 13.9. HRMS (+ESI) calcd. for $C_{19}H_{30}ClN_2$: 321.2092; found: 321.2091.

1-(7-Chloroheptyl)-4-methylbenzo[*g*]quinoline-2,5,10(1*H*)-trione (**33**, $C_{21}H_{22}ClNO_3$)

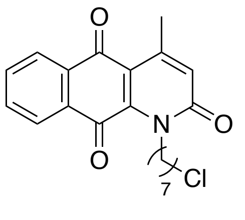

Procedure A was employed on marcanine A (30 mg, 0.13 mmol) and 1-bromo-7-chloroheptane (30 μL, 0.16 mmol) to afford the desired product as yellow liquid (24.9 mg, 53%). ^1H NMR (500 MHz, $CDCl_3$): δ 8.26-8.24 (d, *J* = 7.6 Hz, 1H), 8.21-8.19 (d, *J* = 7.35 Hz, 1H), 7.79-7.73 (m, 2H), 4.56-4.53 (t, *J* = 6.55 Hz, 2H), 3.54-3.52 (t, *J* = 6.75 Hz, 2H), 2.79 (s, 3H), 1.86-1.75 (m, 4H), 1.51-1.24 (m, 7H). ^{13}C NMR (125 MHz, $CDCl_3$): δ 184.0, 182.1, 165.9, 153.4, 149.7, 134.2, 133.7, 133.5, 132.4, 126.9, 126.9, 124.6, 118.5, 66.9,

45.0, 32.5, 28.7, 28.5, 26.7, 25.8, 22.9. HRMS (+APCI) calcd. for $C_{21}H_{23}ClNO_3$: 372.1361; found: 372.1366.

1-(11-Chloroundecyl)-4-methylbenzo[*g*]quinoline-2,5,10(1*H*)-trione (**34**, $C_{25}H_{30}ClNO_3$)

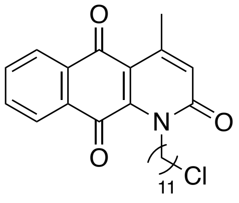

Procedure A was employed on marcanine A (27.4 mg, 0.11 mmol) and **29** (40 µL, 0.13 mmol) to afford the desired product as yellow liquid (20.1 mg, 41%). ^{1}H NMR (500 MHz, $CDCl_3$): δ 8.27-8.25 (dd, *J* = 7.35, 0.8 Hz, 2H), 8.22-8.21 (dd, *J* = 7.8, 1.2 Hz, 2H), 7.80-7.74 (m, 2H), 6.86 (s, 1H), 4.56-4.53 (t, *J* = 6.55 Hz, 2H), 3.53-3.51 (t, *J* = 6.7 Hz, 2H), 2.81 (s, 3H), 1.86-1.73 (m, 4H), 1.49-1.24 (m, 15H). ^{13}C NMR (125 MHz, $CDCl_3$): δ 184.1, 182.2, 165.9, 153.4, 149.7, 134.2, 133.8, 133.6, 132.5, 126.9, 126.9, 124.6, 118.5, 67.2, 45.1, 32.6, 29.5, 29.4, 29.4, 29.3, 28.9, 28.8, 26.8, 25.9, 22.9. HRMS (+ESI) calcd. for $C_{25}H_{31}ClNO_3$: 428.1987; found: 428.1981.

N-(11-Chloroundecyl)-2-trifluoromethylnaphtho[2,3-*d*]imidazole-4,9-dione (**36**, $C_{23}H_{26}N_2O_2ClF_3$)

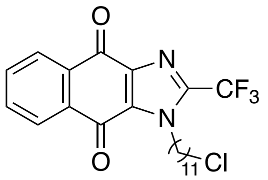

Procedure A was employed with **35** (40 mg, 0.150 mmol) and **29** (60 µL, 0.241 mmol) to afford the desired product as yellow solid (33.4 mg, 49%). ^{1}H NMR (500 MHz, $CDCl_3$): δ 8.28-8.26 (m, 1H), 8.18-8.16 (m, 1H), 7.80-7.75 (m, 1H), 4.55 (t, *J* = 8.05 Hz, 2H), 3.51 (t, *J* = 6.73 Hz, 2H), 1.90-1.82 (m, 2H), 1.78-1.71 (m, 2H), 1.46-1.33 (m, 6H), 1.28 (s, 8H). ^{13}C NMR (125 MHz, $CDCl_3$): δ 178.0, 176.6, 142.2, 142.7 (q, ^2J(C,F) = 39.9 Hz, 1C) , 134.5, 133.9, 133.3, 133.0, 132.8, 127.5, 126.9, 118.3 (q, ^1J(C,F) = 270.6 Hz, 1C), 47.7, 45.2, 32.6, 32.6, 31.0, 29.4, 29.3, 29.3, 28.9, 28.8, 26.8, 26.5. HRMS (+ESI) calcd. for $C_{23}H_{26}N_2O_2ClF_3Na$: 477.1527; found: 177.1529.

Synthesis of 11-aminoundecan-1-ol

2-(11-Hydroxy-undecyl)-isoindole-1,3-dione
11-bromoundecan-1-ol (0.502 g, 2 mmol) and potassium phthalimide (0.481 mg, 2.6 mmol) were dissolved in DMF (10 mL) and stirred at 70°C for 5 h. The mixture was then allowed to reflux at 90°C for 5 h. The mixture was then filtered and washed with EtOAc (20 mL). The combined organic layer was washed with water (4 × 20 mL), brine (10 mL) and dried with Na_2SO_4. The solution was evaporated, redissolved in DCM and purified through a column chromatography using DCM:EtOAc (20:1 – 8:1). The product was then dried *in vacuo* to yield a white solid (0.485 g, 76%). ^{1}H NMR (500 MHz, $CDCl_3$): δ 7.73-7.69 (m, 2H), 7.61-7.58 (m, 2H), 3.55 (t, *J* = 7.38 Hz, 3H), 3.51 (t, *J* = 6.75 Hz, 3H), 2.67 (s, 1H), 1.56-1.52 (m, 2H), 1.47-1.42 (quint, *J* = 7.01 Hz, 2H), 1.20-1.14 (m, 14H). ^{13}C NMR (125 MHz, $CDCl_3$): δ 168.2, 133.6, 131.8, 122.8, 77.2, 62.4, 37.7, 32.5, 29.2, 29.1, 29.1, 28.8, 28.3, 26.5, 25.5. HRMS (+ESI) calcd. for $C_{19}H_{27}NO_3Na$: 340.1883; found: 340.1884. Characterization data in agreement with literature.[S7]

11-Amino-1-undecanol (**38**)
To a solution of 11-phthalimidoundecanol (0.485 g, 1.53 mmol) in THF (9 mL), hydrazine monohydrate (2 mL, 40.1 mmol) was added and the reaction mixture was refluxed for 5 h. Upon completion, the mixture was then filtered and the filtrate evaporated to dryness. The white solid was redissolved in DCM and washed with 3 M NaOH (1 × 20 mL), water (3 × 20 mL) and brine (20 mL). The organic layer was then dried with $MgSO_4$. The solution was dried *in vacuo* to yield the product as a white solid (0.246 g, 86%). ^{1}H NMR (500 MHz, $CDCl_3$): δ 3.49 (t, *J* = 6.73 Hz, 2H), 2.58 (t, *J* = 7.03 Hz, 2H), 2.24 (br s, 3H), 1.45 (quint, *J* = 6.85 Hz, 2H), 1.37-1.34 (m, 2H), 1,91 (br s, 14H). ^{13}C NMR (125 MHz, $CDCl_3$): δ 62.0, 41.9, 33.4, 32.7, 29.4, 29.4, 29.3, 29.3, 29.3, 26.7, 25.7. +ESI-MS calcd. for $C_{11}H_{26}NO$: 188.20; found: 188.36. Characterization data in agreement with literature. [S8]

6-(11-Hydroxyundecyl)amino-7-dichloroquinoline-5,8-dione (**39**, $C_{20}H_{27}N_2O_3Cl$)

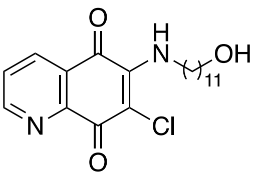

Compound **37** was synthesized as previously reported.[S9] Compounds **37** (45.6 mg, 0.2 mmol) and **38** (75 mg, 0.4 mmol) were dissolved in 5 mL EtOH and stirred for 30 minutes at RT. Upon completion, the reaction mixture was diluted with 10 mL DCM and loaded onto silica gel for purification by column chromatography (DCM/EtOAc 20:1) to afford the desired product as red solid (37.2 mg, 49%) together with the corresponding isomer 7-(11-hydroxyundecyl)amino-6-dichloroquinoline-5,8-dione (29.3 mg, 0.077 mmol, 39%). ^{1}H NMR (500 MHz, $CDCl_3$): δ 8.97 (d, *J* = 3.2 Hz, 1H), 8.32 (d, *J* = 7.35 Hz, 1H), 7.56 (dd, *J* = 7.55, 4.65 Hz, 1H), 6.06 (br s, 1H), 3.84 (t, *J* = 6.75 Hz, 1H), 3.82 (t, *J* = 6.85 Hz, 1H), 3.61 (t, *J* = 6.65 Hz, 2H), 1.78 (br s, 1H), 1.67 (quint, *J* = 7.25 Hz, 2H), 1.53 (quint, *J* = 7 Hz, 2H), 1.38-1.25 (m, 14H). ^{13}C NMR (125 MHz, $CDCl_3$): δ 180.0, 175.1, 155.1, 148.4, 146.0, 143.7, 134.6, 126.6, 126.4, 62.9, 45.1, 32.7, 30.9, 29.4, 29.3, 29.3, 29.1, 26.5, 25.6. HRMS (+ESI) calcd. for $C_{20}H_{28}N_2O_3Cl$: 379.1783; found: 379.1777.

11-[*N*-(7-Chloro-5,8-dioxoquinolin-6-yl)acetamido]undecyl acetate (**40**, $C_{24}H_{31}N_2O_5$)

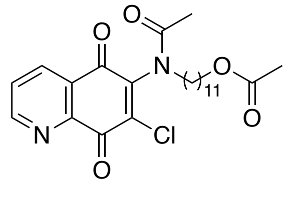

Compound **39** (37.2 mg, 0.098 mmol) was dissolved in 1 mL acetic anhydride containing two drops of concentrated H_2SO_4. The mixture was stirred for 1 h at RT. Upon completion, distilled water was slowly added to quench excess anhydride, and the mixture was extracted with 3 × 10 mL DCM. The combined organic layer was washed with water, followed by 20 mL brine, dried over Na_2SO_4, concentrated under reduced pressure, and purified by column chromatography (16:1 – 4:1 DCM/EtOAc) to give the product as yellow liquid (37.9 mg, 83%). ^{1}H NMR (500 MHz, $CDCl_3$): δ 9.09 (s, 1H), 8.48 (s, 1H), 7.76 (s, 1H), 4.00 (t, *J* = 6.7 Hz, 2H), 3.61 (t, *J* = 7.65 Hz, 2H), 2.29-1.91 (m, 6H), 1.60-1.49 (m, 4H), 1.25-1.21 (m, 14H). ^{13}C NMR (125 MHz, $CDCl_3$): δ 176.1, 171.2, 166.9, 155.2, 150.8, 148.3, 147.0, 145.3, 141.1, 135.5, 128.3, 77.2, 64.5, 43.8, 29.4, 29.3, 29.2, 29.1, 29.1, 28.5, 26.9, 25.8, 22.0, 20.9. HRMS (+ESI) calcd. for $C_{24}H_{31}N_2O_5Na$: 485.1814; found: 485.1818.

11-[*N*-(7-Amino-5,8-dioxoquinolin-6-yl)acetamido]undecyl acetate (**41**, $C_{24}H_{31}N_3O_5$)

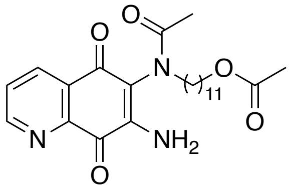

Compound **40** (37.9 mg, 0.082 mmol) was dissolved in 5 mL ACN. 7N ammonia solution in MeOH (20 µL, 0.900 mmol) was added to the solution, and the resulting mixture was heated under reflux overnight. Upon completion, the reaction mixture was adsorbed onto silica gel and purified by column chromatography (8:1 – 1:1 DCM/EtOAc) to give the product as red-orange liquid (17.7 mg, 49%). ^{1}H NMR (500 MHz, $CDCl_3$): δ 8.97 (dd, *J* = 4.35, 1.25 Hz, 1H), 8.48 (dd, *J* = 7.75, 1.25 Hz, 1H), 7.70 (dd, *J* = 7.83, 4.63 Hz, 1H), 6.56 (br s, 1H), 6.27 (br s, 1H), 4.01 (t, *J* = 6.75 Hz, 2H), 3.72-3.66 (m, 1H), 3.41-3.35 (m, 1H), 2.02 (s, 3H), 1.92 (s, 3H), 1.60-1.55 (m, 3H), 1.47-1.44 (m, 1H), 1.27-1.21 (m, 14H). ^{13}C NMR (125 MHz, $CDCl_3$): δ 180.0, 178.0, 171.6, 171.2, 153.5, 146.5, 145.9, 134.8, 129.7, 128.6, 117.9, 64.6, 46.9, 29.5, 29.4, 29.4, 29.3, 29.1, 28.5, 28.1, 27.2, 25.8, 21.8, 21.0. HRMS (+ESI) calcd. for $C_{24}H_{32}N_3O_5$: 442.2347; found: 442.2339.

1-(11-Hydroxyundecyl)-2-methylimidazo[4,5-*g*]quinoline-4,9-dione (**42**, $C_{22}H_{29}N_3O_3$)

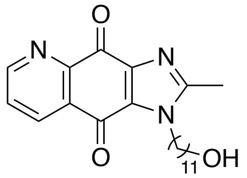

48% HBr solution in water (15 eq.) was added to a stirred solution of **41** (17.7 mg, 0.044 mmol) in 5 mL 1:1 EtOH/EtOAc mixture at RT with stirring. The resulting mixture was heated under reflux overnight. Upon completion, the reaction mixture was adsorbed onto silica gel and purified by column chromatography (2% - 10% MeOH in DCM) to afford the desired product as orange liquid (10.1 mg, 60%). ^{1}H NMR (500 MHz, $CDCl_3$): δ 9.01 (d, *J* = 3.8 Hz, 1H), 8.47 (d, *J* = 7.35 Hz, 1H), 7.65 (dd, *J* = 7.73, 4.58 Hz, 1H), 4.37 (t, *J* = 7.43 Hz, 2H), 3.63 (t, *J* = 6.63 Hz, 2H), 2.59 (s, 3H), 1.81-1.77 (m, 3H), 1.57-1.52 (m, 2H), 1.39-1.24 (m, 14H). ^{13}C NMR (125 MHz, $CDCl_3$): δ 177.0, 174.7, 154.0, 153.8, 148.6, 143.5, 134.5, 131.7, 130.0, 127.0, 63.0, 46.0, 32.7, 30.3, 30.3, 29.4, 29.3, 29.3, 29.1, 26.6, 25.7, 13.4. HRMS (+ESI) calcd. for $C_{22}H_{30}N_3O_3$: 384.2282; found: 382.2293.

11-{2-Methyl-4,9-dioxoimidazo[4,5-*g*]quinolin-1-yl}undecyl tosylate (**43**, $C_{29}H_{34}N_3O_5S$)

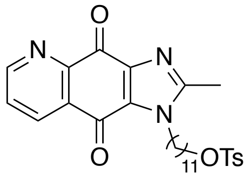

Compound **42** (17.7 mg, 0.044 mmol) and tosyl chloride (21.0 mg, 0.106 mmol) were dissolved in DCM (2 mL). Triethylamine (20 µL, 0.106 mmol) was added to the reaction mixture and allowed to stir for 3 days. Upon completion, the mixture was diluted with DCM (10 mL) and quenched with a concentrated solution of Na_2CO_3 (5 mL). The organic layer was then washed with water (10 mL), brine (10 mL) and dried over $MgSO_4$. The organic layer was concentrated and purified by column chromatography using DCM:MeOH (pure DCM – 24:1) to afford the product as yellow solid (10.1 mg, 60%). ^{1}H NMR ($CDCl_3$, 500 MHz): δ 9.01 (d, *J* = 3.55 Hz, 1H), 8.48 (d, *J* = 7.65 Hz, 1H), 7.77 (d, *J* = 8.2 Hz, 2H), 7.66 (dd, *J* = 7.5, 4.4 Hz, 1H), 7.33 (d, *J* = 8.1 Hz, 2H), 4.39 (br s, 2H), 4.00 (t, *J* = 6.5 Hz, 2H), 2.63 (s, 3H), 2.44 (s, 3H), 1.81 (br s, 2H), 1.65-1.59 (m, 2H), 1.39-1.21 (m, 14H). ^{13}C NMR ($CDCl_3$, 125 MHz): δ 174.7, 154.0, 153.8, 148.5, 144.6, 142.9, 134.7, 134.0, 133.2, 131.6, 130.0, 129.8, 127.8, 127.1, 70.6, 46.2, 30.3, 29.3, 29.2, 29.2, 29.0, 28.8, 28.8, 26.6, 25.3, 21.6, 13.4. HRMS (+ESI) calcd. for $C_{29}H_{35}N_3O_5S$: 537.23; found: 537.40.

3. Synthesis and characterization of final compounds

(3-{2-Methyl-4,9-dioxonaphtho[2,3-*d*]imidazol-1-yl}propyl)triphenylphosphonium iodide (**3**, $C_{33}H_{28}N_2O_2IP$)

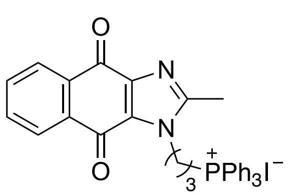

Compound **23** (29.3 mg, 0.101 mmol) was dissolved in ACN (5 mL). KI (20.3 mg, 0.122 mmol) and triphenylphosphine (28.1 mg, 0.107 mmol) were added into the solution, and the reaction mixture was refluxed for 1 day. Upon completion, purification was carried out as in Procedure B to afford the desired product as yellow solid (35.7 mg, 41%). ^{1}H NMR (500 MHz, $CDCl_3$): δ 8.06-8.04 (m, 1H), 7.99-7.97 (m, 1H), 7.84-7.80 (m, 6H), 7.77-7.74 (m, 3H), 7.67-7.64 (m, 8H), 4.83 (t, *J* = 7.28 Hz, 2H), 4.03 (m, 2H), 2.68 (s, 3H), 2.20 (m, 2H). ^{13}C NMR (125 MHz, $CDCl_3$): δ 178.6, 176.8, 154.5, 143.5, 135.2, 135.2, 133.8, 133.7, 133.6, 133.3, 132.8, 132.5, 131.6, 130.5, 130.4, 126.9, 126.1, 117.9, 117.2, 45.6, 23.5, 20.7, 20.3, 14.7. HRMS (+ESI) calculated for $C_{33}H_{28}N_2O_2P$: 515.1883; found: 515.1887.

(5-{2-Methyl-4,9-dioxonaphtho[2,3-*d*]imidazol-1-yl}pentyl)triphenylphosphonium iodide (**4**, $C_{35}H_{32}N_2O_2IP$)

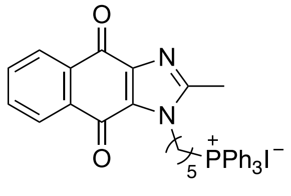

Compound **24** (40.9 mg, 0.129 mmol) was dissolved in ACN (7.5 mL). KI (25.7 mg, 0.155 mmol) and triphenylphosphine (67.7 mg, 0.155 mmol) were added into the solution, and the reaction mixture was refluxed for 4 days. Upon completion, purification was carried out as in Procedure B to afford the desired product as yellow solid (32.2 mg, 37%). ^{1}H NMR (500 MHz, $CDCl_3$): δ 8.14-8.12 (m, 1H), 7.99-7.97 (m, 1H), 7.82-7.76 (m, 9H), 7.70-7.64 (m, 8H), 4.35 (t, *J* = 6.45 Hz, 2H), 3.77-3.72 (m, 2H), 2.58 (s, 3H), 1.87-1.86 (m, 4H), 1.73-1.72 (m, 2H). ^{13}C NMR (125 MHz, $CDCl_3$): δ 178.8, 176.0, 153.7, 143.2, 135.1, 135.1, 133.6, 133.5, 133.3, 133.0, 133.6, 131.9, 130.6, 130.5, 126.8, 126.1, 118.3, 117.6, 45.4, 29.6, 27.0, 26.9, 22.8, 22.4, 22.1, 22.1, 13.9. HRMS (+ESI) calculated for $C_{35}H_{32}N_2O_2P$: 543.2196; found: 543.2194.

(7-{2-Methyl-4,9-dioxonaphtho[2,3-*d*]imidazol-1-yl}heptyl)triphenylphosphonium iodide (**5**, $C_{37}H_{36}N_2O_2IP$)

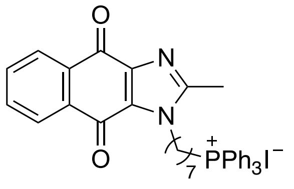

Compound **25** (44.9 mg, 0.130 mmol) and KI (43.2 mg, 0.260 mmol) were dissolved in ACN (7.5 mL) and refluxed for 1 hour. Triphenylphosphine (68.3 mg, 0.260 mmol) was then added into the solution, and the reaction mixture was heated under reflux for 4 days. Upon completion, purification was carried out as in Procedure B to afford the desired product as yellow solid (76.0 mg, 84%). ^{1}H NMR (500 MHz, $CDCl_3$): δ 8.06-8.05 (m, 1H), 7.97-7.95 (m, 1H), 7.75-7.59 (m, 17H), 4.28 (t, *J* = 7.6 Hz, 2H), 3.56-3.52 (m, 2H), 2.48 (s, 3H), 1.72-1.68 (m, 2H), 1.60 (s, 4H), 1.33-1.31 (m, 4H). ^{13}C NMR (125 MHz, $CDCl_3$): δ 178.7, 175.7, 153.1, 142.8, 134.9, 134.9, 133.4, 133.3, 133.2, 132.8, 132.4, 131.8, 130.4, 130.3, 126.5, 126.1, 118.1, 117.4, 45.5, 30.0, 29.9, 29.8, 28.2, 25.9, 22.9, 22.5, 22.2, 13.5. HRMS (+ESI) calculated for $C_{37}H_{36}N_2O_2P$: 571.2509; found: 571.2513.

Synthesis of **5** without sequential addition of reagents

Compound **25** (64 mg, 0.19 mmol), KI (93 mg, 0.56 mmol) and triphenylphosphine (146 mg, 0.56 mmol) were dissolved in ACN (5 mL) and heated under reflux for 3 days. Upon completion, the reaction mixture was filtered and purification was carried out as in Procedure B to afford the desired product as yellow solid (113.4 mg, 87%).

Synthesis of **5** under microwave-assisted condition

Compound **25** (49 mg, 0.14 mmol) and KI (70 mg, 0.42 mmol) were dissolved in ACN (3 mL) and heated at 160°C under microwave irradiation for 1 minute. Triphenylphosphine (111.1 mg, 0.42 mmol) was then added into the solution, and the reaction mixture was further heated at 160°C under microwave irradiation for 1 hour. Upon completion, the reaction mixture was filtered and purification was carried out as in Procedure B to afford the desired product as yellow solid (96.6 mg, 98%).

(9-(2-Methyl-4,9-dioxo-4,9-dihydro-1*H*-naphtho[2,3-*d*]imidazol-1-yl)nonyl)triphenylphosphonium iodide (**6**, $C_{39}H_{40}N_2O_2IP$)

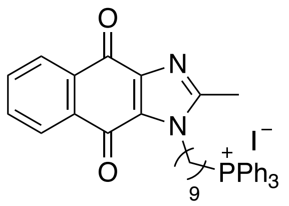

Procedure B was employed with **26** (31.1 mg, 0.08 mmol), KI (42 mg, 0.25 mmol) and triphenylphosphine (66 mg, 0.25 mmol) to afford the desired product as a yellow liquid (23.6 mg, 39%). ^{1}H NMR (500 MHz, CD_3OD): δ 8.09-8.07 (m, 1H), 8.06-8.04 (m, 1H), 7.89-7.86 (m, 3H), 7.83-7.73 (m, 14H), 4.39-4.37 (t, *J* = 7.5 Hz, 2H), 3.44-3.38 (m, 2H), 2.53 (s, 3H), 1.81-1.75 (quint, J = 7.05 Hz, 2H), 1.69-1.62 (sext, *J* = 7.8 Hz, 2H), 1.58-1.52 (quint, J = 7.05 Hz, 2H), 1.37-1.28 (m, 8H). ^{13}C NMR (125 MHz, CD_3OD): δ 178.4, 175.7, 153.5, 141.9, 134.9, 134.9, 133.6, 133.5, 133.5, 133.4, 133.0, 132.5, 131.9, 130.2, 130.1, 126.2, 126.1, 118.9, 118.3, 45.5, 30.1, 30.0, 29.6, 28.7, 28.5, 28.3, 26.1, 22.1, 22.1, 21.5, 21.1, 11.48. HRMS (+ESI) calcd. for $C_{39}H_{40}N_2O_2P$: 599.2822; found: 598.2826.

(11-{2-Methyl-4,9-dioxonaphtho[2,3-*d*]imidazol-1-yl}undecyl)triphenylphosphonium iodide (**7**, $C_{41}H_{44}N_2O_2IP$)

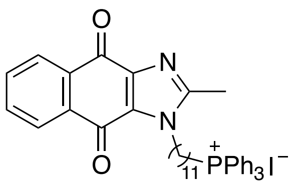

Compound **27** (33.3 mg, 0.083 mmol) and KI (41.4 mg, 0.249 mmol) were dissolved in DMF (5 mL) and refluxed for 1 hour. Triphenylphosphine (65.4 mg, 0.249 mmol) was then added into the solution, and the mixture refluxed for 4 days. Upon completion, the mixture was added to water (20 mL) and extracted with DCM (4 × 20 mL). The organic layers were combined, washed with water (4 × 20 mL), brine (20 mL) and dried over $MgSO_4$. Purification was carried out as in Procedure B to afford the desired product as yellow solid (16.6 mg, 27%). ^{1}H NMR (500 MHz, $CDCl_3$): δ 8.19-8.16 (m, 1H), 8.11-8.08 (m, 1H), 7.81-7.77 (m, 9H), 7.71-7.67 (m, 8H), 4.35 (t, *J* = 7.53 Hz, 2H), 3.67-3.61 (m, 2H), 2.55 (s, 3H), 1.76 (quint, *J* = 7.51 Hz, 2H), 1.60-1.59 (m, 4H), 1.37-1.19 (m, 12H). ^{13}C NMR (125 MHz, $CDCl_3$): δ 179.0, 176.1, 153.0, 143.1, 135.0, 135.0, 133.7, 133.6, 133.4, 133.1, 132.7, 132.1, 130.5, 130.4, 126.8, 126.4, 118.5, 117.8, 45.8, 30.4, 30.3, 30.2, 29.2, 29.1, 29.0, 29.0, 26.4, 23.1, 22.7, 22.6, 22.6, 13.4. HRMS (+ESI) calculated for $C_{41}H_{44}N_2O_2P$: 627.3135; found: 627.3148.

Triphenyl(heptyl)phosphonium iodide (**8**, $C_{25}H_{30}IP$)

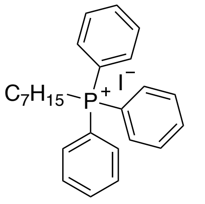

Procedure B was employed with 1-bromoheptane (40 μL, 0.2 mmol), KI (0.1 g, 0.6 mmol) and triphenylphosphine (0.157 g, 0.6 mmol) to afford the desired product as yellow liquid (94.2 mg, 96%). ^{1}H NMR (500 MHz, DMSO-*d6*): δ 7.92-7.55 (m, 15H), 3.62-3.57 (m, 2H), 1.53-1.44 (m, 4H), 1.30-1.14 (m, 14H), 0.82 (t, *J* = 7.1 Hz). ^{13}C NMR (125 MHz, DMSO-*d6*,): δ 134.8, 134.8, 133.6, 133.5, 130.2, 130.1, 118.9, 118.2, 30.9, 29.8, 29.7, 27.8, 21.9, 21.7, 21.7, 20.4, 20.0, 13.9. HRMS (+ESI) calculated for $C_{25}H_{30}P$: 361.2080; found: 361.2092.

Triphenyl(undecyl)phosphonium iodide (**9**, $C_{29}H_{38}IP$)

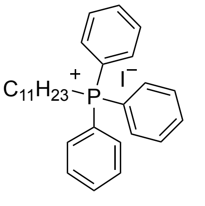

Procedure B was employed with 1-bromoundecane (70 μL, 0.2 mmol), KI (0.1 g, 0.6 mmol) and triphenylphosphine (0.157 g, 0.6 mmol) to afford the desired product as yellow liquid (0.1018 g, 93%). ^{1}H NMR (500 MHz, $CDCl_3$,): δ 7.72-7.59 (m, 15H), 3.44-3.40 (m, 2H), 1.51-1.50 (m, 4H), 1.12-1.06 (m, 14H), 0.71 (t, *J* = 7.0 Hz). ^{13}C NMR (125 MHz, $CDCl_3$): δ 134.8, 134.8, 133.2, 133.1, 130.2, 130.1, 117.9, 117.2, 31.4, 30.1, 29.9, 29.2, 29.0, 29.0, 28.8, 28.7, 28.7, 22.8, 22.4, 22.2, 22.1, 22.1, 13.7. HRMS (+ESI) calculated for $C_{29}H_{38}P$: 417.2706; found: 417.2711.

[(2-Methylbenzimidazol-1-yl)heptyl]triphenylphosphonium iodide (**10**, $C_{33}H_{36}N_2IP$)

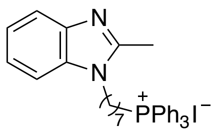

Compound **31** (33.5 mg, 0.13 mmol) and KI (0.129 g, 0.78 mmol) were dissolved in ACN (5 mL) and heated under reflux for 1 h. Triphenylphosphine (0.100 g, 0.78 mmol) was then added into the solution, and the reaction mixture was further heated under reflux for 3 days. Upon completion, purification was carried out as in Procedure B to afford the desired product as colorless liquid (48.2 mg, 62%). ^{1}H NMR (500 MHz, CD_3OD): δ 7.88-7.85 (m, 3H), 7.81-7.72 (m, 12H), 7.53-7.51 (d, *J* = 7.45 Hz, 1H), 7.44-7.43 (d, J = 7.5 Hz, 1H) 7.21-7.16 (m, 2H), 4.20-4.17 (t, *J* = 7.15 Hz, 2H), 3.42-3.36 (m, 2H), 2.57 (s, 3H), 1.81-1.75 (quint, *J* = 7.3 Hz, 2H), 1.65-1.58 (sext, *J* = 7.55 Hz, 2H), 1.56-1.49 (quint, J = 6.7 Hz, 2H), 1.39-1.28 (m, 4H). ^{13}C NMR (125 MHz, CD_3OD): δ 153.2, 142.6, 136.2, 136.2, 134.9, 134.8, 131.6, 131.5, 123.4, 123.1, 120.2, 119.6, 118.8, 111.1, 44.7, 31.4, 31.2, 30.5, 29.4, 27.4, 23.3, 23.3, 22.9, 22.5, 13.6. HRMS (+ESI) calculated for $C_{33}H_{36}N_2P$: 491.2611; found: 491.2620.

[(2-Methylbenzimidazol-1-yl)undecyl]triphenylphosphonium iodide (**11**, $C_{37}H_{44}N_2IP$)

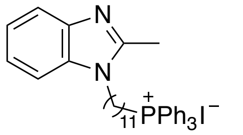

Compound **32** (47.3 mg, 0.150 mmol) and KI (73.0 mg, 0.44 mmol) were dissolved in ACN (5 mL) and heated under reflux for 1 h. Triphenylphosphine (0.116 g, 0.44 mmol) was then added into the solution, and the reaction mixture was further heated under reflux for 3 days. Upon completion, purification was carried out as in Procedure B to afford the desired product as colorless liquid (71.4 mg, 72%). ^{1}H NMR (500 MHz, CD_3OD): δ 7.88-7.79 (m, 9H), 7.76-7.73 (m, 6H), 7.53-7.51 (d, *J* = 7.95 Hz, 1H), 7.44-7.42 (d, *J* = 7.95 Hz, 1H) 7.23-7.20 (t, *J* = 7.35 Hz, 1H), 7.18-7.15 (t, *J* = 8.35 Hz, 1H), 4.19-4.17 (t, *J* = 7.25 Hz, 2H), 3.46-3.40 (m, 2H), 2.58 (s, 3H), 1.80-1.74 (quint, *J* = 7.15 Hz, 2H), 1.68-1.60 (sext, *J* = 8.55 Hz, 2H), 1.56-1.50 (quint, *J* = 7.15 Hz, 2H), 1.30-1.21 (m, 12H). ^{13}C NMR (125 MHz, CD_3OD): δ 153.1, 142.7, 136.2, 136.2, 136.1, 134.8, 134.8, 131.5, 131.4, 123.4, 123.1, 120.3, 119.6, 118.8, 111.1, 44.7, 31.5, 31.4, 30.6, 30.4, 30.3, 30.2, 29.8, 27.7, 23.5, 23.5, 22.9, 22.5, 13.6. HRMS (+ESI) calculated for $C_{37}H_{44}N_2P$: 547.3237; found: 547.3244.

(7-(4-Methyl-2,5,10-trioxo-5,10-dihydrobenzo[*g*]quinolin-1(2*H*)-yl)heptyl)triphenylphosphonium iodide (**12**, $C_{39}H_{37}NO_3IP$)

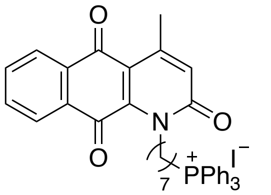

Compound **33** (25 mg, 0.07 mmol) and KI (33 mg, 0.2 mmol) were dissolved in ACN (5 mL) and heated under reflux for 1 h. Triphenylphosphine (53 mg, 0.2 mmol) was then added into the solution, and the reaction mixture was further heated under reflux for 3 days. Upon completion, purification was carried out as in Procedure B to afford the desired product as yellow semi-solid (26 mg, 53%). ^{1}H NMR (500 MHz, CD_3OD): δ 8.11-8.09 (dd, *J* = 7.6, 0.9 Hz, 2H), 8.06-8.05 (dd, *J* = 7.5, 0.8 Hz, 2H), 7.89-7.79 (m, 10H), 7.78-7.73 (m, 7H), 6.84 (s, 1H), 4.44-4.42 (t, *J* = 6.6 Hz, 2H), 3.49-3.44 (m, 2H), 2.69 (s, 3H), 1.81-1.75 (quint, *J* = 6.65 Hz, 2H), 1.75-1.68 (m, 2H), 1.65-1.59 (quint, *J* = 6.7 Hz, 2H), 1.46-1.45 (m, 4H). ^{13}C NMR (125 MHz, CD_3OD): δ 184.9, 183.6, 167.4, 155.3, 150.8, 136.4, 136.4, 135.9, 135.2, 135.0, 134.9, 133.6, 131.7, 131.6, 128.1, 127.8, 125.9, 120.5, 119.8, 119.6, 68.2, 31.6, 31.5, 30.7, 30.6, 29.7, 29.5, 26.7, 23.7, 23.6, 23.1, 22.7. HRMS (+ESI) calcd. for $C_{39}H_{37}NO_3P$: 598.2506; found: 598.2518.

(11-(4-Methyl-2,5,10-trioxo-5,10-dihydrobenzo[*g*]quinolin-1(2*H*)-yl)undecyl)triphenylphosphonium iodide (**13**, $C_{43}H_{45}NO_3IP$)

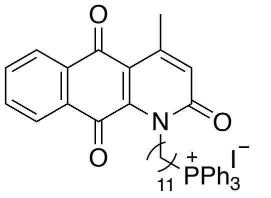

Compound **34** (20.1 mg, 0.05 mmol) and KI (30 mg, 0.16 mmol) were dissolved in ACN (5 mL) and heated under reflux for 1 h. Triphenylphosphine (42 mg, 0.16 mmol) was then added into the solution, and the reaction mixture was further heated under reflux for 3 days. Upon completion, purification was carried out as in Procedure B to afford the desired product as yellow solid (27.7 mg, 75%). ^{1}H NMR (500 MHz, CD_3OD): δ 8.13-8.09 (m, 2H), 7.89-7.86 (m, 3H), 7.82-7.72 (m, 12H), 7.65-7.61 (m, 3H), 7.56-7.52 (m, 2H), 6.87 (s, 1H), 4.45-4.42 (t, *J* = 6.5 Hz, 2H), 3.44-3.38 (m, 2H), 2.70 (s, 3H), 1.80-1.74 (quint, *J* = 6.75 Hz, 2H), 1.69-1.61 (sext, *J* = 7.75 Hz, 2H), 1.57-1.51 (quint, *J* = 6.95 Hz, 2H), 1.47-1.41 (quint, J = 6.8 Hz, 2H), 1.35-1.27 (m, 10H). ^{13}C NMR (125 MHz, CD_3OD): δ 184.9, 183.6, 167.4, 155.3, 150.8, 136.4, 136.4, 135.9, 135.2, 135.0, 134.9, 133.9, 133.9, 133.6, 133.2, 133.2, 131.7, 131.6, 130.2, 130.1, 128.0, 127.8, 125.9, 120.5, 119.8, 119.6, 68.5, 31.8, 31.6, 30.7, 30.6, 30.5, 30.5, 30.1, 29.9, 27.2, 23.7, 23.6, 23.2, 23.1, 22.7. HRMS (+ESI) calcd. for $C_{43}H_{45}NO_3P$: 654.3132; found: 654.3136.

{11-[4,9-Dioxo-2-(trifluoromethyl)naphtho[2,3-d]imidazole-1-yl]undecyl}triphenylphosphonium iodide (**14**, $C_{41}H_{41}F_3N_2O_2IP$)

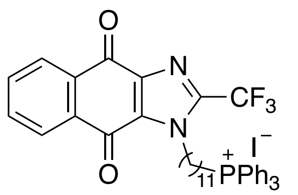

Compound **36** (33.4 mg, 0.073 mmol) was dissolved in ACN (5 mL). KI (36.6 mg, 0.220 mmol) and triphenylphosphine (57.8 mg, 0.220 mmol) were added, and the reaction mixture was heated under reflux for 5 days. Upon completion, purification was carried out as per Procedure B to afford the desired product as orange solid (29.5 mg, 60%). ^{1}H NMR (500 MHz, $CDCl_3$): δ 8.27-8.25 (m, 1H), 8.19-8.17 (m, 1H), 7.83-7.77 (m, 11H), 7.72-7.68 (m, 6H), 4.54 (t, *J* = 7.98 Hz, 2H), 3.70-3.65 (m, 2H), 1.88-1.81 (m, 2H), 1.62-1.61 (m, 4H), 1.44-1.38 (m, 2H), 1.32-1.21 (m, 10H). ^{13}C NMR (125 MHz, $CDCl_3$): δ 177.9, 176.3, 142.0, 135.0, 135.0, 134.4, 134.0, 133.5, 133.5, 133.2, 132.8, 132.5, 131.9, 131.9, 131.8, 130.5, 130.4, 128.4, 128.3, 127.2, 126.9, 118.2 (q ^3J(C,F) = 90.2 Hz), 47.5, 30.8, 30.4, 30.3, 29.1, 29.1, 29.0, 28.7, 26.3, 23.1, 22.7, 22.5, 22.5. HRMS (+ESI) calcd. for $C_{41}H_{41}F_3N_2O_2P$: 681.2852; found: 681.2862.

(11-{2-Methyl-4,9-dioxoimidazo[4,5-*g*]quinolin-1-yl}undecyl)triphenylphosphonium iodide (**15**, $C_{40}H_{43}N_3O_2IP$)

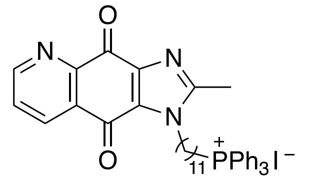

Procedure B was employed with **43** (8.5 mg, 0.016 mmol) for 2 h to afford the desired product as yellow solid (8.4 mg, 0.011 mmol, 70%). ^{1}H NMR (500 MHz, CD_3OD): δ 8.91 (dd, *J* = 9.6, 1.3 Hz, 1H), 8.55 (dd, *J* = 7.88, 1.63 Hz, 1H), 7.91-7.87 (m, 4H), 7.83-7.74 (m, 12H), 4.44 (t, *J* = 7.6 Hz, 2H), 3.43-3.37 (m, 2H), 2.58 (s, 3H), 1.83 (quint, *J* = 7.46 Hz, 2H), 1.69-1.61 (m, 2H), 1.53 (quint, *J* = 7.38 Hz, 2H), 1.42-1.26 (m, 12H). ^{13}C NMR (125 MHz, CD_3OD): δ 178.0, 175.8, 155.6, 154.5, 149.5, 143.7, 136.3, 136.3, 136.2, 134.9, 134.8, 131.6, 131.5, 129.0, 120.4, 119.7, 47.0, 31.6, 31.5, 30.9, 30.4, 30.3, 30.1, 29.9, 27.5, 23.6, 23.5, 22.9, 22.5, 13.1. HRMS (+ESI) calculated for $C_{40}H_{43}N_3O_2P$: 628.3087; found: 628.3088.

(7-{2-Methyl-4,9-dioxonaphtho[2,3-*d*]imidazol-1-yl}heptyl)pyridinium iodide (**16**, $C_{24}H_{26}N_3O_2I$)

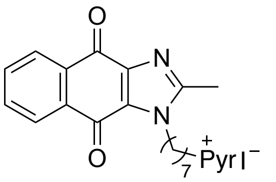

Compound **25** (19.6 mg, 0.057 mmol) and KI (28.3 mg, 0.171 mmol) were dissolved in ACN (5 mL) and heated under reflux for 1 hour. Pyridine (20 μL, 0.171 mmol) was added into the solution, and the reaction mixture was further heated under reflux for 1 day. Purification was carried out as in Procedure B with an additional recrystallisation step after column chromatography using MeOH to yield the desired product as orange crystals (52.3 mg, 64%). ^{1}H NMR (500 MHz, CD_3OD): δ 9.01 (d, *J* = 5.65 Hz, 2H), 8.58-8.55 (m, 1H), 8.10-8.05 (m, 4H), 7.75-7.73 (m, 2H), 4.63 (t, *J* = 7.58 Hz, 2H), 4.38 (t, *J* = 7.68 Hz, 2H), 2.51 (s, 3H), 2.05-2.00 (m, 2H), 1.83-1.77 (m, 2H), 1.43-1.40 (m, 6H). ^{13}C NMR (125 MHz, CD_3OD): δ 180.3, 177.0, 155.0, 146.8, 146.0, 146.0, 145.9, 135.0, 135.0, 134.4, 133.8, 133.4, 129.5, 127.5, 63.0, 46.9, 32.3, 30.8, 29.4, 27.2, 26.8, 13.5. HRMS (+ESI) calculated for $C_{24}H_{26}N_3O_2$: 388.2020; found: 388.2015.

(7-{2-Methyl-4,9-dioxonaphtho[2,3-*d*]imidazol-1-yl}heptyl)tricyclohexylphosphonium iodide (**17**, $C_{37}H_{54}N_2O_2IP$)

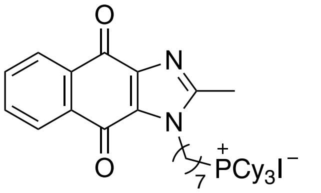

Compound **25** (17.2 mg, 0.101 mmol) and KI (24.8 mg, 0.150 mmol) were dissolved in ACN (5 mL) and heated under reflux for 1 hour. Tricyclohexylphosphine (42.0 mg, 0.150 mmol) was then added into the solution, and the reaction mixture was refluxed for 3 days. Upon completion, purification was carried out as in Procedure B to afford the desired product as yellow solid (20.1 mg, 56%). ^{1}H NMR (500 MHz, CD_3OD): δ 8.12-8.10 (m, 1H), 8.08-8.06 (m, 1H), 7.79-7.75 (m, 2H), 4.43 (t, *J* = 7.53 Hz, 2H), 2.58-2.51 (m, 2H), 2.56 (s, 3H), 2.29-2.23 (m, 2H), 1.99-1.77 (m, 17H), 1.65-1.28 (m, 24H). ^{13}C NMR (125 MHz, CD_3OD): δ 179.8, 177.1, 154.9, 143.4, 135.0, 134.8, 134.4, 133.9, 133.4, 127.6, 127.4, 46.8, 32.0, 31.9, 31.0, 30.9, 30.7, 29.3, 28.1, 28.0, 27.6, 27.5, 27.3, 26.6, 23.4, 23.3, 16.4, 16.0, 13.2. HRMS (+ESI) calculated for $C_{37}H_{54}N_2O_2P$: 589.3917; found: 589.3921.

(11-{2-Methyl-4,9-dioxonaphtho[2,3-*d*]imidazol-1-yl}undecyl)tricyclohexylphosphonium iodide (**18**, $C_{41}H_{62}N_2O_2IP$)

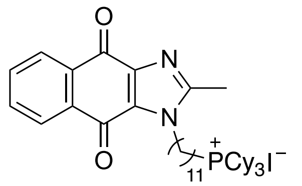

Procedure B was employed with **27** (15.8 mg, 0.040 mmol) and tricyclohexylphosphine (33.0 mg, 0.120 mmol) to afford the desired product as yellow solid (30.0 mg, 98%). ^{1}H NMR (500 MHz, $CDCl_3$): δ 8.19-8.16 (m, 1H), 8.11-8.07 (m, 1H), 7.70-7.67 (m, 2H), 4.36-4.33 (t, ^3J(H,H) = 7.5 Hz, 2H), 2.65-2.58 (m, 2H), 2.57 (s, 3H), 2.41-2.35 (m, 2H), 1.98-1.95 (m, 5H), 1.89 (m, 7H), 1.83-1.73 (m, 6H), 1.55-1.42 (m, 14H), 1.37-1.16 (m, 17H). ^{13}C NMR (125 MHz, $CDCl_3$): δ 178.9, 176.1, 152.9, 143.1, 133.6, 133.3, 133.1, 132.7, 132.0, 126.8, 126.3, 45.8, 35.4, 34.9, 31.2, 31.1, 30.2, 30.0, 29.7, 29.2, 29.2, 28.9, 28.9, 27.3, 27.2, 26.8, 26.7, 26.4, 26.4, 26.3, 26.2, 26.2, 26.0, 25.3, 22.8, 22.8, 16.1, 15.8, 13.4. HRMS (+ESI) calculated for $C_{41}H_{62}N_2O_2P$: 645.4543; found: 645.4551.

(7-{2-Methyl-4,9-dioxonaphtho[2,3-*d*]imidazol-1-yl}heptyl)trihexylphosphonium iodide (**19**, $C_{37}H_{60}N_2O_2IP$)

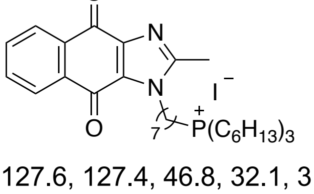

Procedure B was employed with **25** (31.0 mg, 0.090 mmol) and trihexylphosphine (77.0 mg, 0.27 mmol) to afford the desired product as blue-green solid (20.1 mg, 31%). ^{1}H NMR (500 MHz, CD_3OD): δ 8.11-8.09 (m, 1H), 8.08-8.06 (m, 1H), 7.79-7.75 (m, 2H), 4.43-4.40 (t, *J* = 7.55 Hz, 2H), 2.56 (s, 3H), 2.28-2.21 (m, 8H), 1.85-1.82 (m, 2H), 1.62-1.48 (m, 20H), 1.36-1.34 (m, 12H), 0.93-0.90 (t, *J* = 6.25 Hz, 9H). ^{13}C NMR (125 MHz, CD_3OD): δ 179.8, 177.1, 154.9, 143.4, 134.9, 134.8, 134.4, 133.9, 133.4, 127.6, 127.4, 46.8, 32.1, 31.6, 31.4, 30.9, 29.3, 27.3, 23.5, 22.4, 22.4, 22.4, 19.6, 19.3, 14.3, 13.2. HRMS (+ESI) calculated for $C_{37}H_{60}N_2O_2P$: 595.4387; found: 595.4400.

4. Synthesis of marcanine A

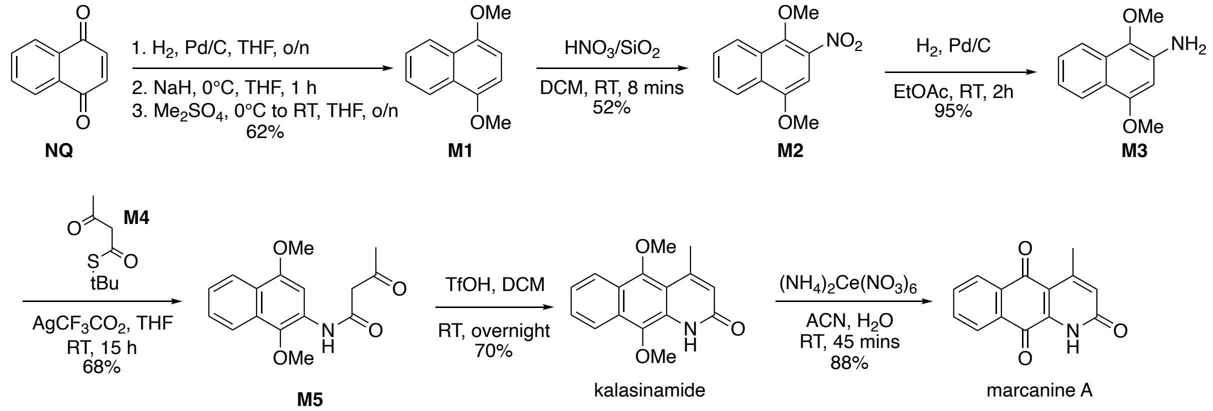

1,4-Dimethoxynaphthalene (**M1**, $C_{12}H_{12}O_2$)

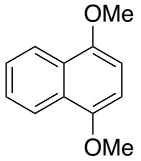

The procedure from Evans and Brandt was employed.[S11] 1,4-Naphthoquinone (**NQ**, 3.2 g, 20 mmol) was dissolved in 40 mL of EtOH/H_2O (1:1). Sodium borohydride (1.36 g, 40 mmol, 2 eq.) was added in portions over 30 minutes, and the resulting mixture was stirred for 30 mins at RT. A solution of 10 M KOH (8 mL) was added and the mixture was stirred further for 30 minutes. The reaction mixture was then heated under reflux, and dimethyl sulfate (7.6 mL, 80 mmol, 4 eq.) was added over 1 hour. The resulting mixture was heated under reflux overnight. Upon completion, the reaction mixture was extracted with DCM (3 × 20 mL) and the combined organic layer was dried over Na_2SO_4 and concentrated under reduced pressure. Purification was carried out by column chromatography (100:1 hexane/EtOAc) to afford the desired product as white solid (0.71 g, 19%). ^{1}H NMR (500 MHz, $CDCl_3$): 8.86-8.83 (m, 2H), 8.13-8.11 (m, 2H), 7.22 (s, 2H), 4.53 (s, 6H). ^{13}C NMR (125 MHz, $CDCl_3$): 149.4, 126.3, 125.8, 121.7, 103.1, 55.6. Characterization data in agreement with literature.

1,4-Dimethoxy-2-nitronaphthalene (**M2**, $C_{12}H_{11}NO_4$)

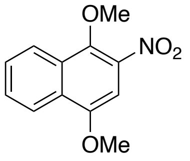

The procedure was adopted from Tapia *et al.*[S12] 1,4-dimethoxynaphthalene (**M1**, 0.7138 g, 3.8 mmol) was dissolved in 38 mL dichloromethane. 1.5 g of HNO_3-SiO_2 (prepared as described in reference) was added into the solution, and the mixture was stirred vigorously for 8 mins. Reaction mixture was filtered to remove silica, the filtrate was concentrated under reduced pressure and purified with column chromatography (hexane/EtOAc 100:1 – 40:1) to give the product as yellow solid (0.4593 g, 52%). ^{1}H NMR (500 MHz, $CDCl_3$): 8.29-8.26 (m, 2H), 7.69-7.65 (m, 2H), 7.22 (s, 1H), 4.09 (s, 3H), 4.04 (s, 3H). ^{13}C NMR (125 MHz, $CDCl_3$): 151.9, 145.7, 138.7, 128.9, 128.9, 128.8, 128.2, 124.1, 122.7. 98.5, 63.6, 56.1. HRMS (ESI) calcd. for $C_{12}H_{12}NO_4$: 234.0761; found: 234.0760. Characterization data in agreement with literature.

1,4-Dimethoxynaphthalen-2-amine (**M3**, $C_{12}H_{13}NO_2$)

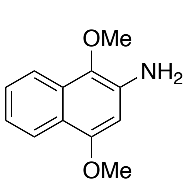

1,4-Dimethoxy-2-nitronaphthalene (**M2**, 0.4526 g, 1.94 mmol) was dissolved in 13 mL anhydrous EtOAc, and 10% Pd/C (0.09 g) was added into the solution. The mixture was bubbled with hydrogen gas and stirred vigorously at RT under H_2 for 2 h. Upon completion, the reaction mixture was purged with N_2, filtered through Celite, and the filtrate was purified with column chromatography (hexane/EtOAc 4:1 – 1:1) to yield the product as brown liquid (0.3733 g, 95%). ^{1}H NMR (400 MHz, Acetone-d$_6$): 8.01-7.99 (dt, *J* = 8.4, 0.72 Hz, 1H), 7.78-7.76 (dt, *J* = 8.44, 0.8 Hz, 1H), 7.40-7.37 (ddd, *J* = 8.2, 6.8, 1.28 Hz, 1H), 7.15-7.12 (ddd, *J* = 8.16, 6.8, 1.2 Hz, 1H), 6.58 (s, 1H), 4.74 (bs, 2H), 3.91 (s, 3H), 3.77 (s, 3H). ^{13}C NMR (100 MHz, Acetone-d$_6$): 153.3, 137.9, 137.9, 132.9, 130.2, 127.3, 122.9, 121.6, 120.6, 120.2, 98.5, 98.5. HRMS (ESI) calcd. for $C_{12}H_{14}NO_2$: 204.1019; found: 204.1018. Characterization data in agreement with literature.[S10]

1-(*tert*-Butylsulfanyl)butane-1,3-dione (**M4**, $C_8H_{14}O_2S$)

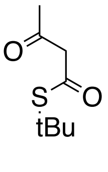

The compound was synthesized as described by López-Alvarado *et al.*[S13] 2,2,6-trimethyl-4*H*- 1,3-dioxin-4-one (2.14 g, 15 mmol) and *tert*-butylthiol (2.72 g, 30 mmol) were added into a round bottom flask with a stirrer, and the resulting mixture was refluxed at 120°C for 10 h. Upon completion, the reaction mixture was cooled to room temperature and purified with chromatography (hexane/dichloromethane 2:1) to give the product as orange oil (78%). ^{1}H NMR (500 MHz, $CDCl_3$):

12.86 (s), 5.32 (s), 3.56 (s, 2H), 2.25 (s, 3H), 1.89 (s), 1.49 (s), 1.47 (s, 9H). ^{13}C NMR (125 MHz, $CDCl_3$): 200.2, 196.1, 192.6, 172.9, 100.2, 59.1, 49.0, 48.1, 30.1, 29.6, 20.9. Characterization data in agreement with literature.

N-(1,4-Dimethoxynaphthalen-2-yl)-3-oxobutanamide (**M5**, $C_{16}H_{17}NO_4$)

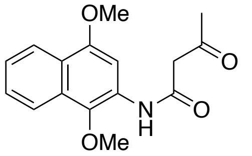

Compound **M4** (0.34 g, 1.68 mmol) was dissolved in 20 mL THF, then thioester M4 (0.322 g, 1.85 mmol, 1.1 eq.) and silver (I) trifluoroacetate (0.44 g, 2 mmol, 1.2 mmol) were added into the solution. The reaction mixture was stirred at RT overnight. The mixture was filtered through a short silica column, and the filtrate was concentrated under reduced pressure and purified with column chromatography (hexane/EtOAc 9:1 – 3:2) to yield the product as light brown solid (0.3289 g, 68%). ^{1}H NMR (500 MHz, $CDCl_3$): 9.67 (s, 1H), 8.20-8.19 (d, *J* = 8.45 Hz, 1H), 7.99-7.97 (d, *J* = 9.05 Hz, 2H), 7.53-7.50 (t, *J* = 7.9 Hz, 1H), 7.42-7.39 (t, *J* = 7.95 Hz, 1H), 5.29 (s, 1H), 4.00 (s, 3H), 3.95 (s, 3H), 2.36 (s, 3H). ^{13}C NMR (125 MHz, $CDCl_3$): 204.7, 163.5, 152.1, 137.0, 127.6, 127.6, 126.9, 124.3, 123.2, 122.5, 121.1, 98.6, 61.7, 55.8, 50.2, 31.2. HRMS (-APCI) calcd. for $C_{16}H_{16}NO_4$: 286.1085; found: 286.1087. Characterization data in agreement with literature.[S10]

5,10-Dimethoxy-4-methyl-1H,2H-benzo[g]quinoline-2-one (kalasinamide)

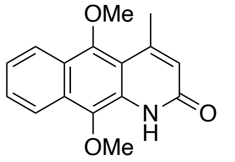

Intermediate **M4** (0.2967 g, 1.03 mmol) was dissolved in 6 mL anhydrous dichloromethane. Triflic acid (1.55 mL, 18 mmol) was slowly added via syringe with swirling. The reaction mixture was then stirred at RT overnight, then poured into 11 g of ice and diluted with dichloromethane. The organic layer was separated, and the aqueous layer was washed with 2 × 20 mL chloroform. The combined organic layers were washed with 2 × 10 mL distilled H_2O, 2 × 10 mL brine and dried over Na_2SO_4. Purification was carried out with column chromatography (8:1 to 1:4 hexane/EtOAc) to give kalasinamide as a light green solid (0.1946 g, 70%). ^{1}H NMR (500 MHz, $CDCl_3$): 9.20 (s, 1H), 8.18-8.16 (d, *J* = 8.75 Hz, 1H), 8.06-8.04 (d, *J* = 8.3 Hz, 1H), 7.58-7.55 (dd, *J* = 7.55, 6.55 Hz, 1H), 7.47-7.44 (t, *J* = 7.75 Hz, 1H), 6.46 (s, 1H), 3.98 (s, 3H), 3.96 (s, 3H), 2.78 (s, 3H). ^{13}C NMR (125 MHz, $CDCl_3$): 161.7, 151.7, 148.6, 135.9, 128.2, 128.1, 127.8, 124.6, 123.9, 123.5, 123.1, 121.1, 63.9, 61.9, 23.2. MS (ESI) calculated for $C_{16}H_{16}NO_3$: 270.11; found: 270.11. Characterization data in agreement with literature.[S10]

4-Methyl-1H,2H,5H,10H-benzo[g]quinoline-2,5,10-trione (marcanine A)

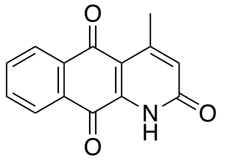

Kalasinamide (0.1571 g, 0.58 mmol) was dissolved a mixture of 10 mL acetonitrile and 5 mL distilled H_2O. Into the solution was added ceric ammonium nitrate (0.96 g, 1.75 mmol, 3 eq.) and the reaction mixture was stirred at RT for 45 minutes. The mixture was diluted with 10 mL distilled H_2O and extracted with 3 × 20 mL chloroform. The combined organic layers were washed with 2 × 20 mL distilled H_2O, dried over Na_2SO_4, and concentrated to give marcanine A as a yellow solid (0.1235 g, 88%). ^{1}H NMR (500 MHz, DMSO-d_6): 11.95 (br s, 1H), 8.08 (t, *J* = 7.15 Hz, 2H), 7.94-7.90 (td, *J* = 7.45, 1.1 Hz, 1H), 7.88-7.85 (td, *J* = 7.55, 1.1 Hz, 1H), 6.60 (s, 1H), 2.58 (s, 3H). ^{13}C NMR (125 MHz, DMSO-d_6): 181.7, 178.1, 150.8, 135.2, 133.7, 132.8, 130.5, 126.5, 125.9, 22.2. HRMS (ESI) calculated for $C_{14}H_{18}NO_3$: 238.0510; found: 238.0513. Characterization data in agreement with literature.[S10]

5. Purity determination of final compounds

Compound purity was determined by reverse phase HPLC. Chromatograms were collected on a Shimadzu Nexera SR HPLC system (Shimadzu Scientific Instruments, Columbia, MD, USA). Separations were carried out on a Zorbax Eclipse XDB-C18 (150 × 4.6 mm, 5 μm; Agilent Tech. Inc., Loveland, CO) on two mobile phases with the following compositions:

A: 85% ACN/15% H_2O + 25 mM $HCOONH_4$; flow rate 1.5 mL/min on Zorbax column; Detector channels 254/285 nm.

B: 95% MeOH/ 5% H_2O + 25 mM $HCOONH_4$; flow rate 1.5 mL/min on Zorbax column; Detector channels 254/285 nm.

The chromatogram was monitored for 15 min for the detection of the major peak of test compound, which was expressed as a percentage of total peaks detected during the run. Two wavelengths (254 nm, 280 or 285 nm) were employed for major peak detection per mobile phase. The mobile phase flow rate was 1.5 mL/min.

Table S1 Purities of final compounds based on % area of major peak.

Compound	% Major Peak					
	Mobile Phase I			Mobile Phase II		
		λ_1= 254 nm	λ_2= 280/285 nm		λ_1= 254 nm	λ_2= 280/285 nm
**3**	A	99.6	99.9	B	99.9	99.9
**4**	A	96.1	99.0	B	99.6	98.5
**5**	A	96.6	99.2	B	99.0	99.1
**6**	A	96.6	98.9	B	99.2	99.6
**7**	A	96.8	99.4	B	99.8	99.6
**8**	A	99.8	99.5	B	98.7	97.0
**9**	A	95.6	98.6	B	99.3	98.3
**10**	A	95.0	98.4	B	99.1	99.9
**11**	A	95.0	98.5	B	96.2	97.8
**12**	A	95.9	97.8	B	97.1	95.9
**13**	A	94.9	97.5	B	95.2	95.0
**14**	A	95.3	98.5	B	99.1	99.4
**15**	A	95.0	95.7	B	99.5	99.0
**16**	A	95.1	98.9	B	99.4	99.6
**17**	A	96.4	98.5	B	98.7	99.8
**18**	A	96.8	99.3	B	99.1	99.4
**19**	A	94.9	96.7	B	94.7	96.1

6. NMR spectra of final compounds and intermediates

(3-{2-Methyl-4,9-dioxonaphtho[2,3-*d*]imidazol-1-yl}propyl)triphenylphosphonium iodide (**3**, $C_{33}H_{28}N_2O_2IP$)

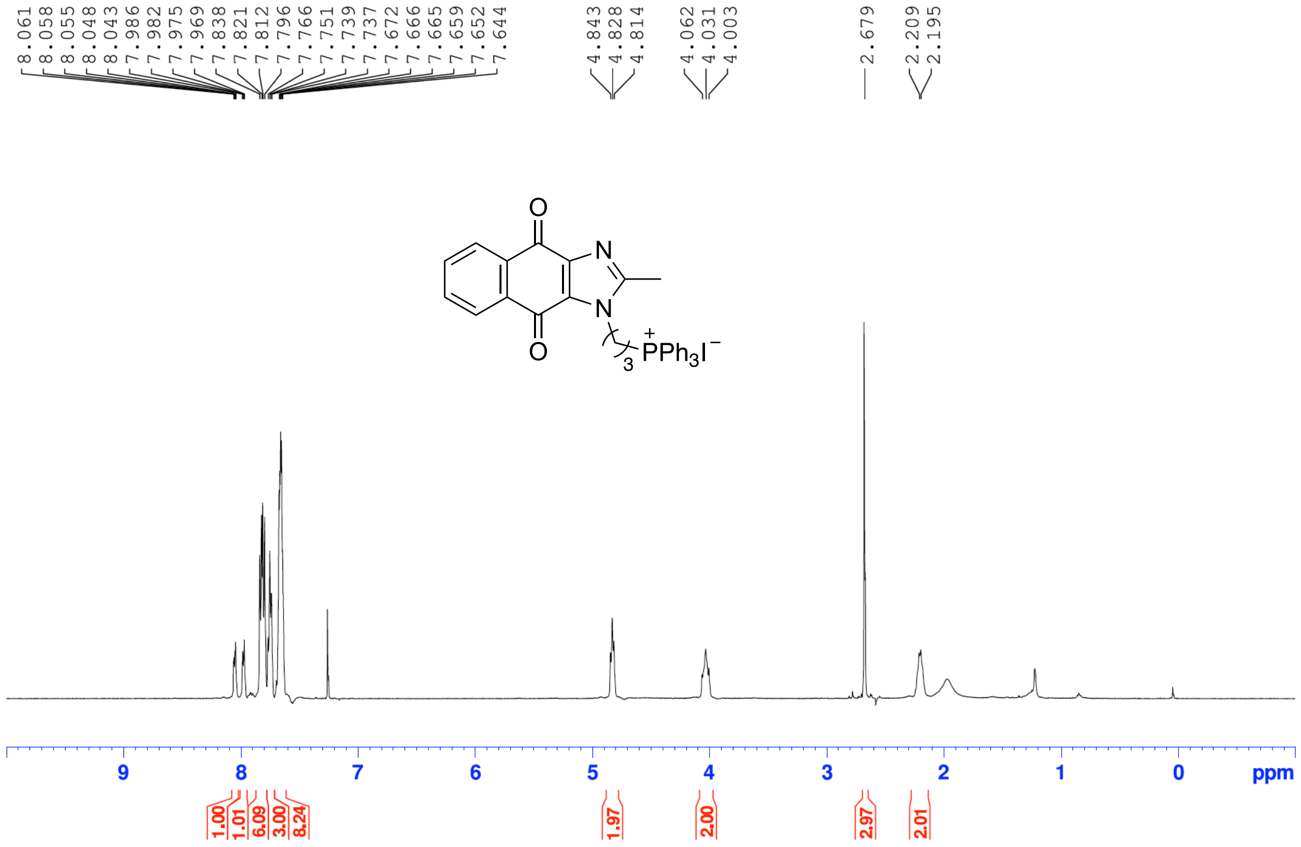

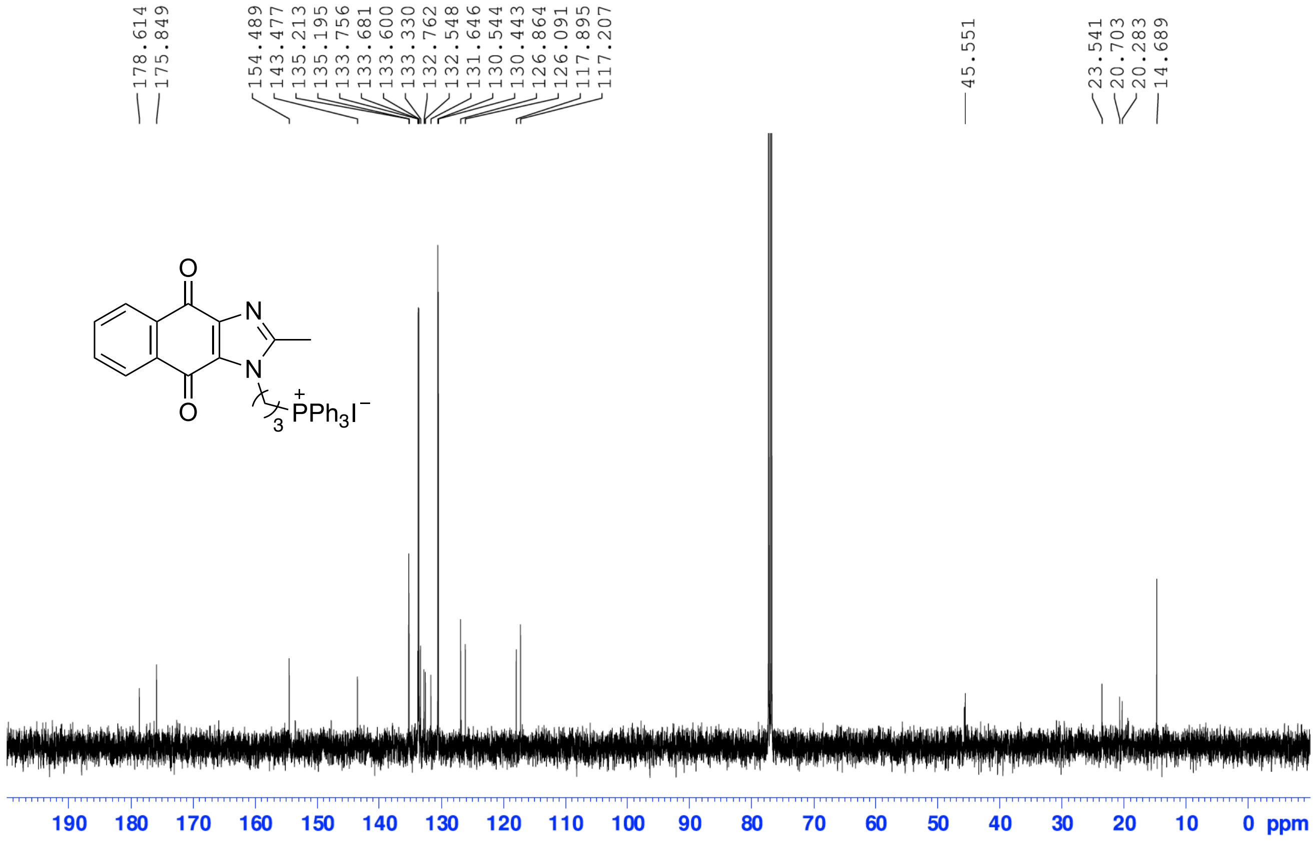

(5-{2-Methyl-4,9-dioxonaphtho[2,3-*d*]imidazol-1-yl}pentyl)triphenylphosphonium iodide (**4**, $C_{35}H_{32}N_2O_2IP$)

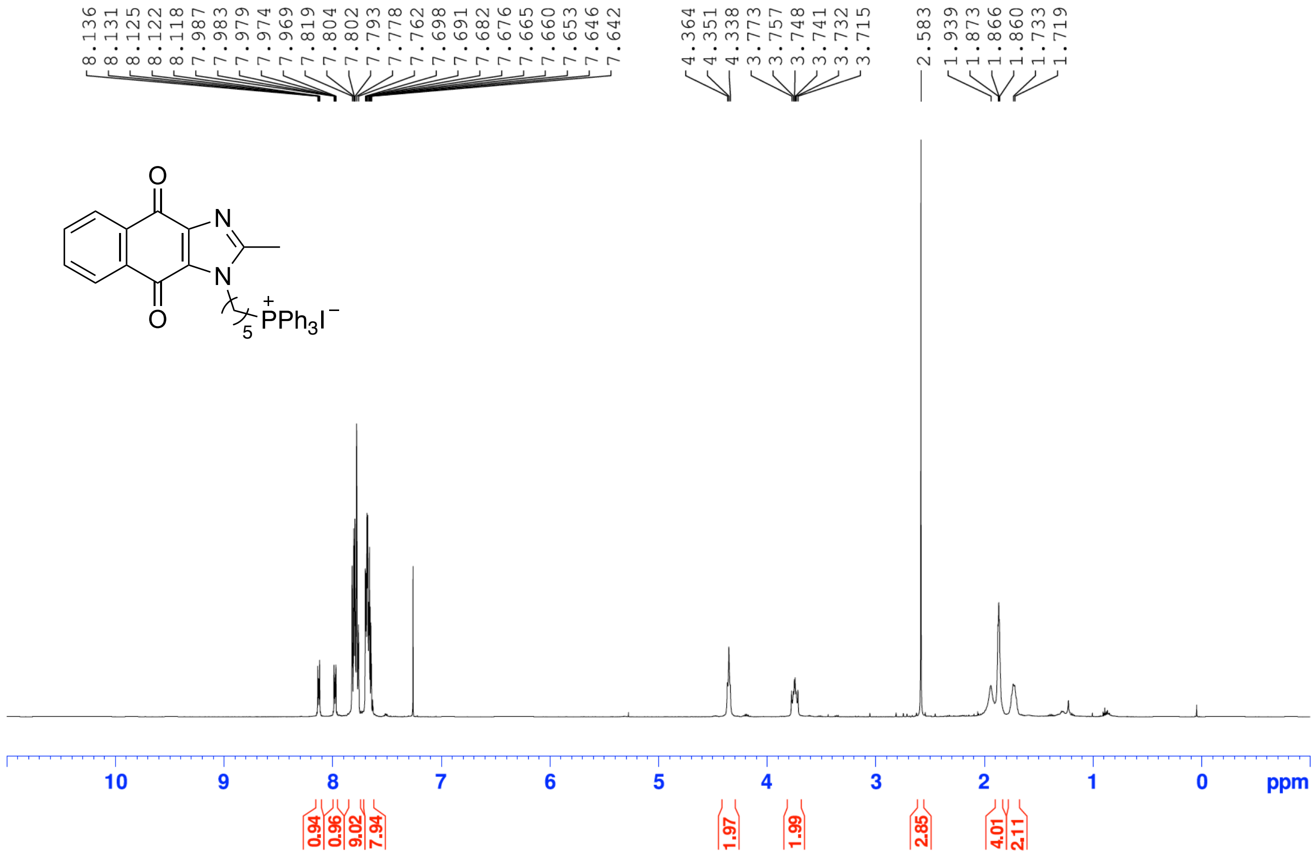

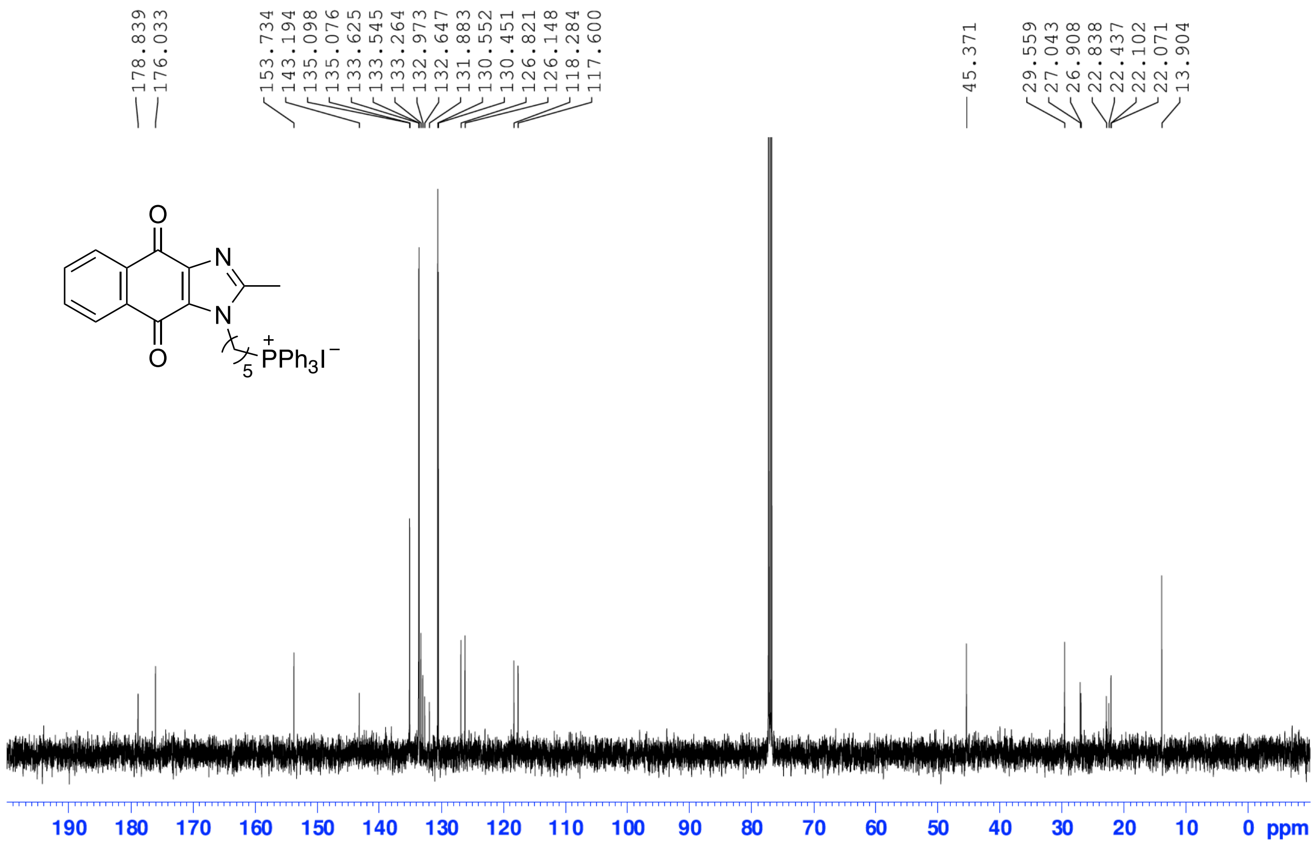

(7-{2-Methyl-4,9-dioxonaphtho[2,3-*d*]imidazol-1-yl}heptyl)triphenylphosphonium iodide (**5**, $C_{37}H_{36}N_2O_2IP$)

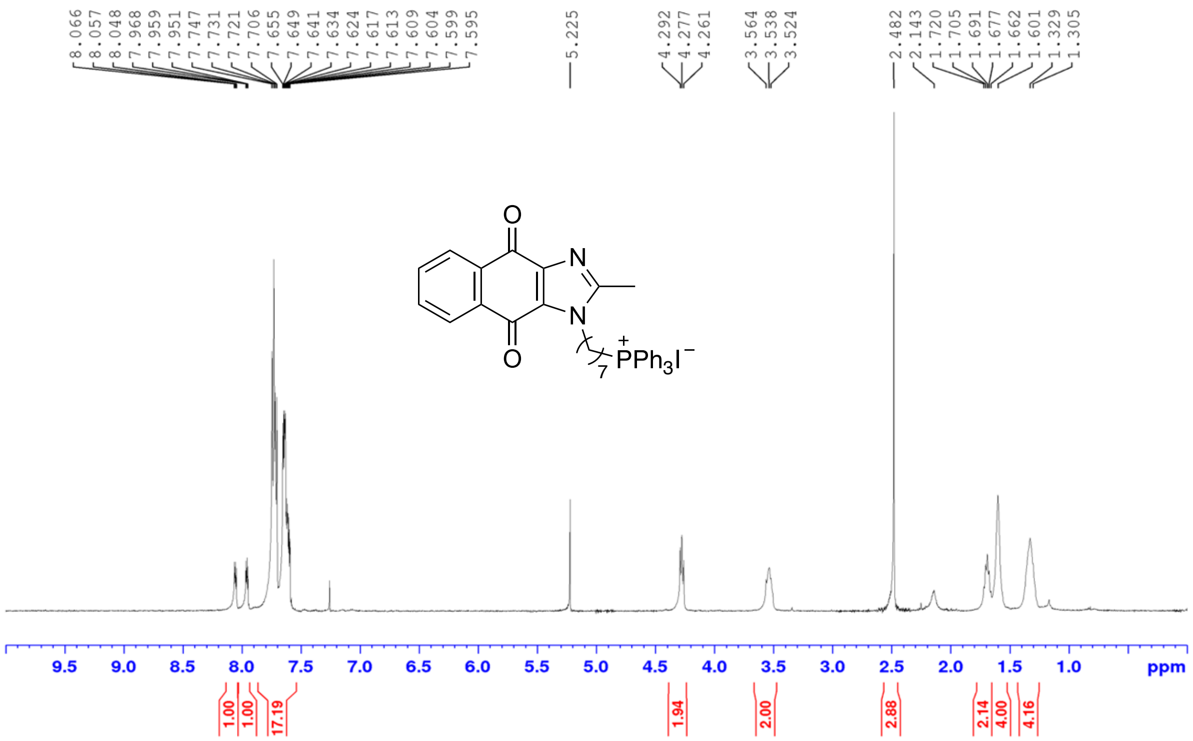

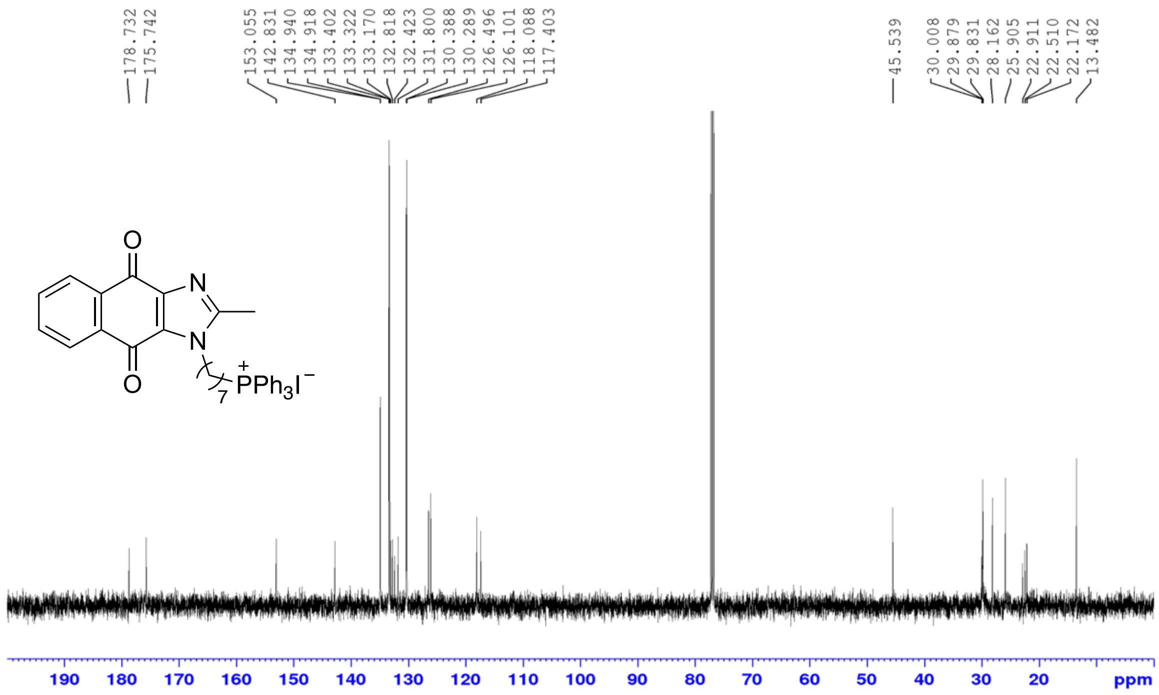

(9-(2-methyl-4,9-dioxo-4,9-dihydro-1*H*-naphtho[2,3-*d*]imidazol-1-yl)nonyl)triphenylphosphonium iodide (**6**, $C_{39}H_{40}N_2O_2IP$)

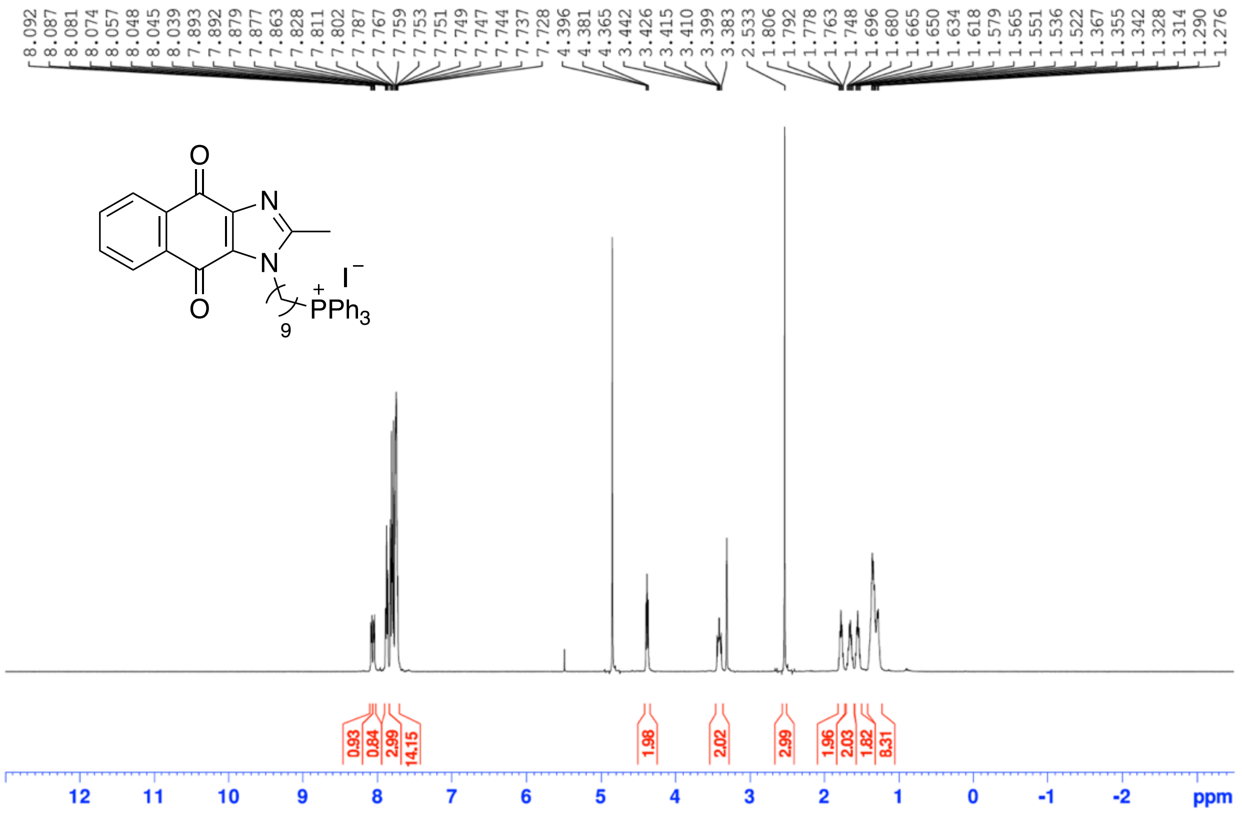

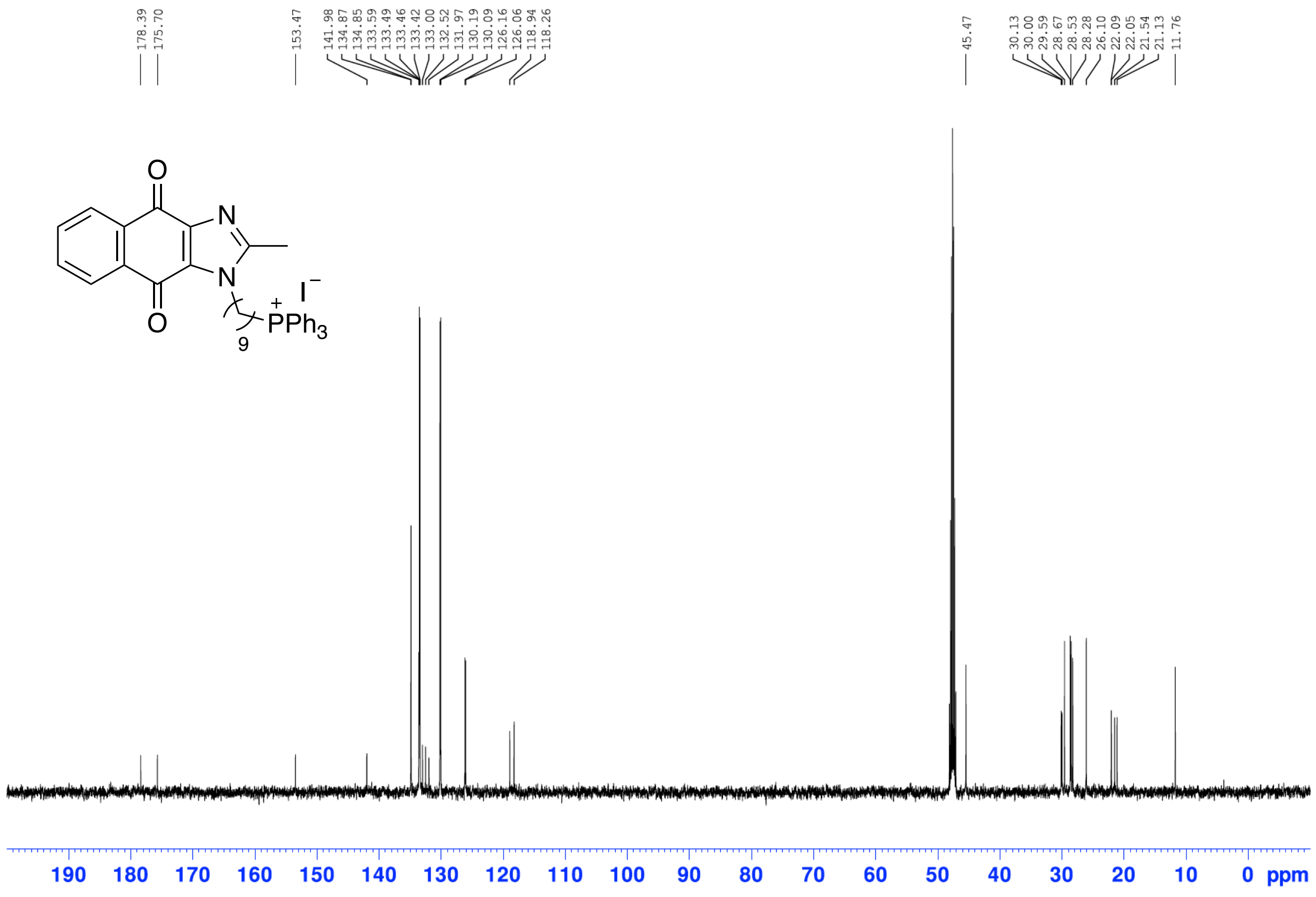

^{31}P NMR

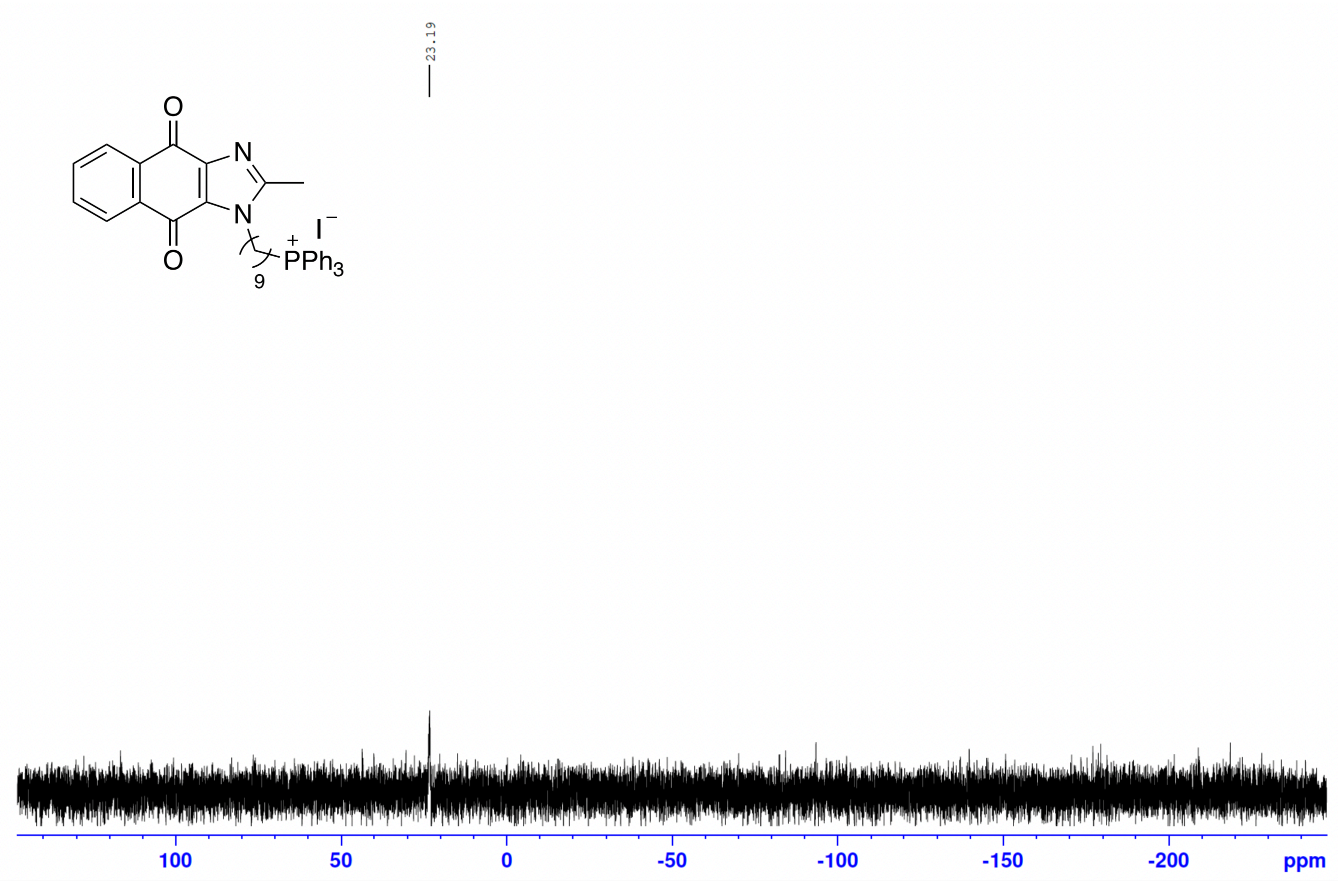

(11-{2-Methyl-4,9-dioxonaphtho[2,3-*d*]imidazol-1-yl}undecyl)triphenylphosphonium iodide (**7**, $C_{41}H_{44}N_2O_2IP$)

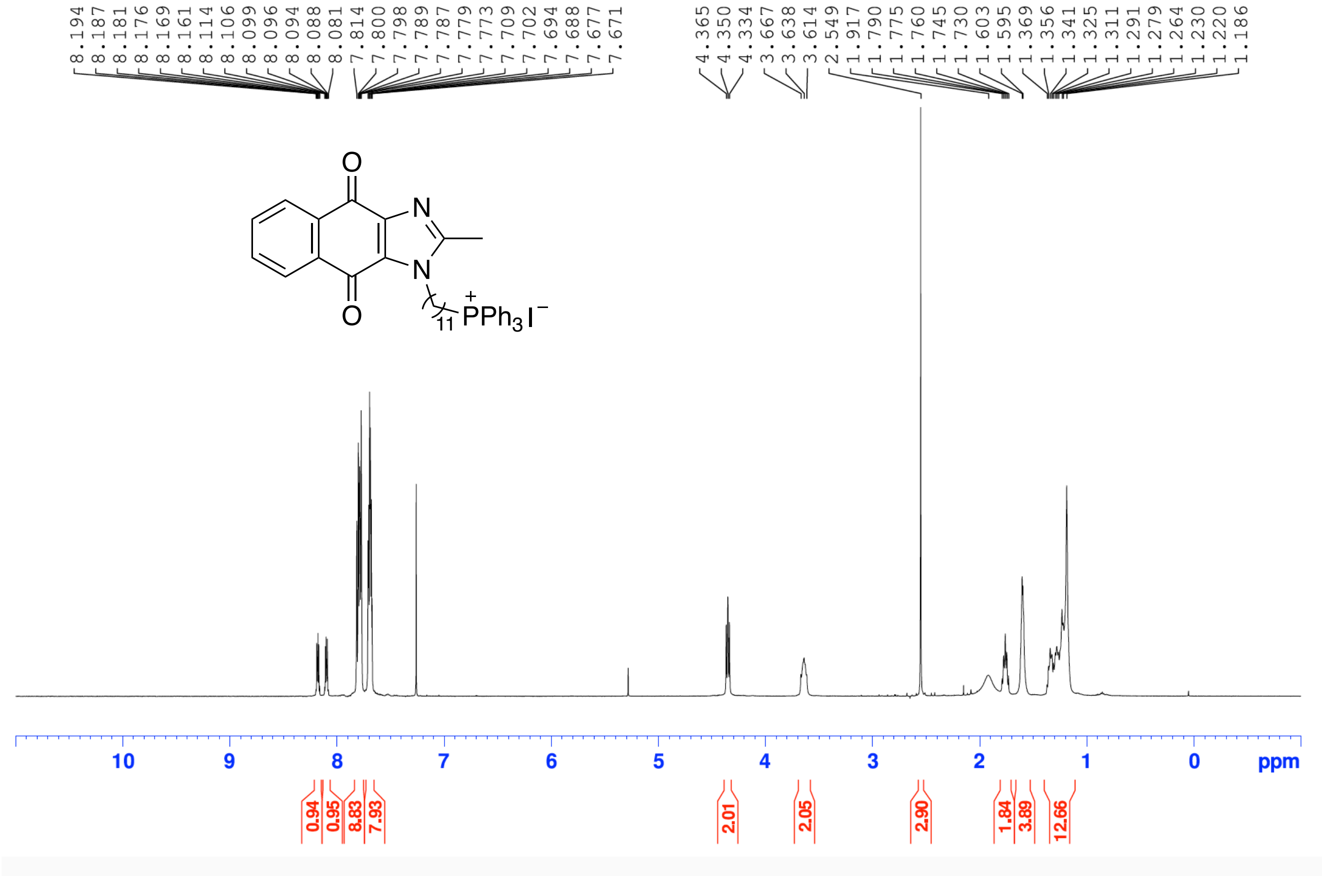

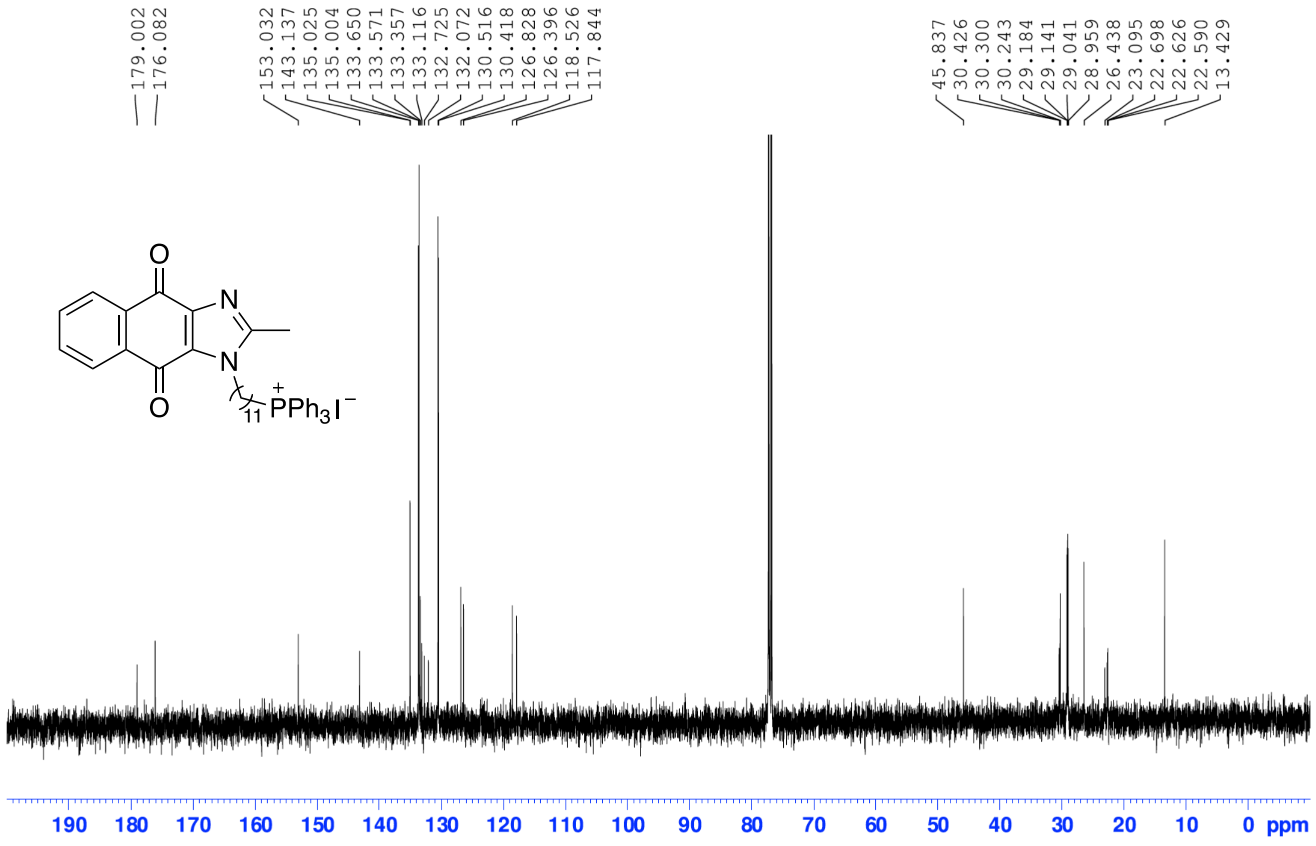
179.002
176.082
153.032
143.137
135.025
135.004
133.650
133.571
133.357
133.116
132.725
132.072
130.516
130.418
126.828
126.396
118.526
117.844
45.837
30.426
30.300
30.243
29.184
29.141
29.041
28.959
26.438
23.095
22.698
22.626
22.590
13.429
ppm

^{31}P NMR

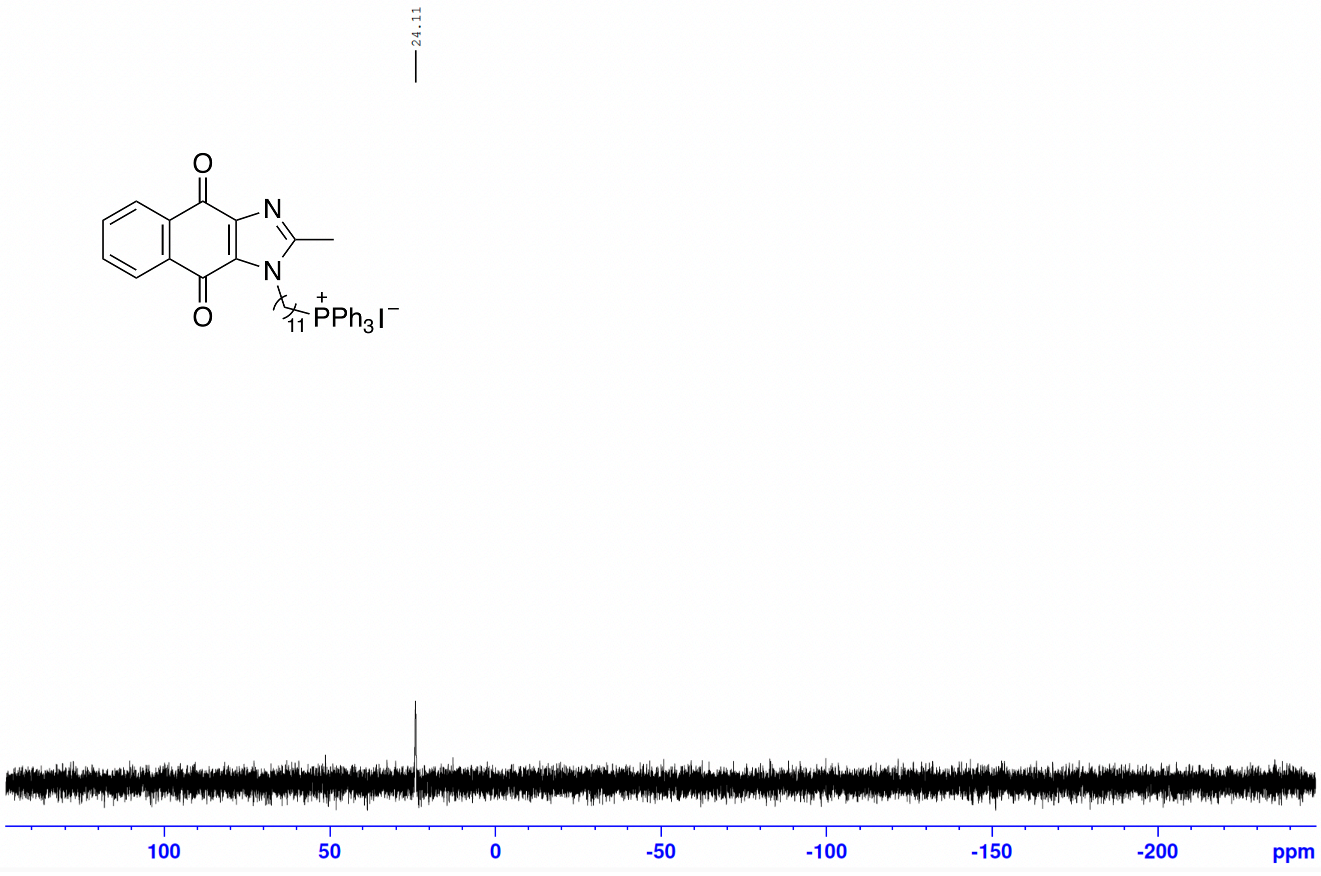
24.11
ppm

Triphenyl(heptyl)phosphonium iodide (**8**, $C_{25}H_{30}IP$)

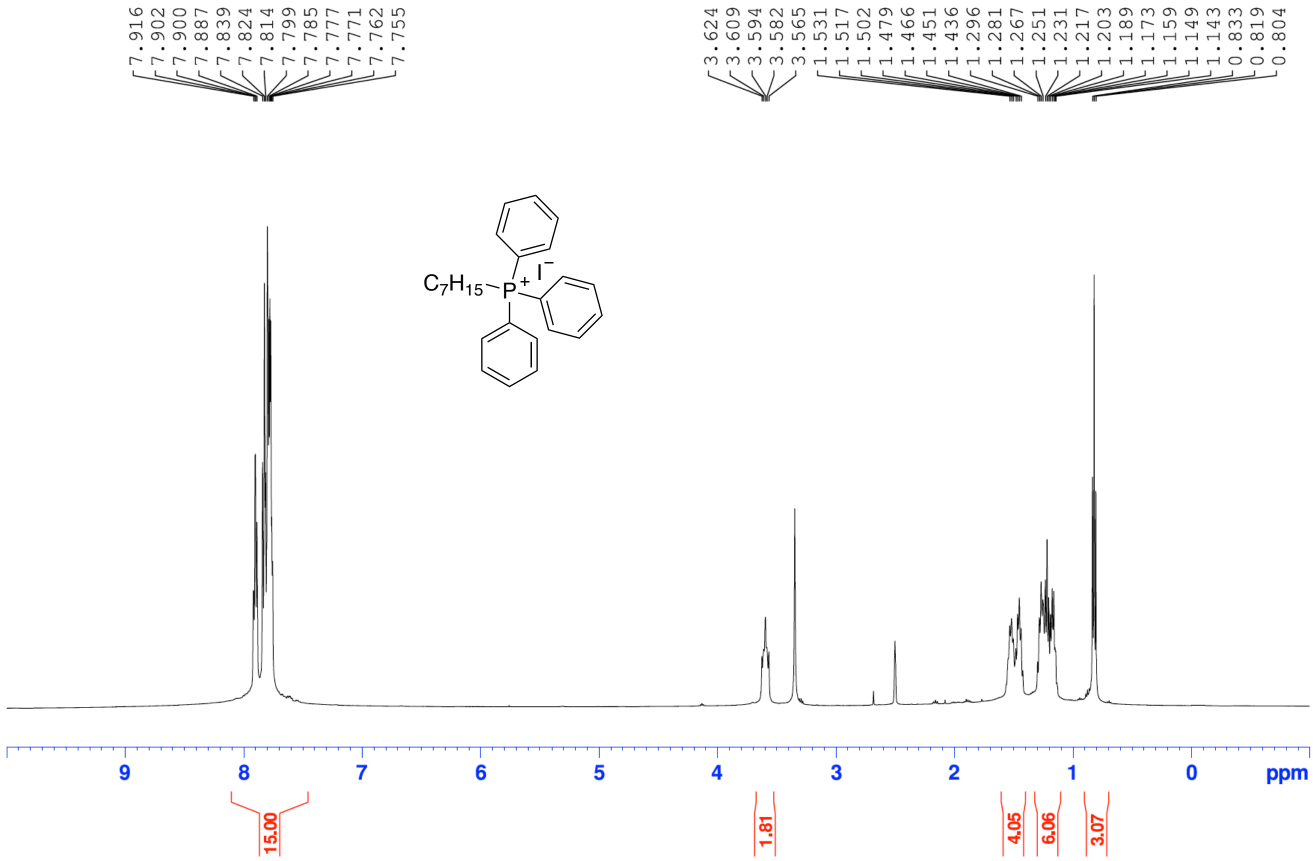

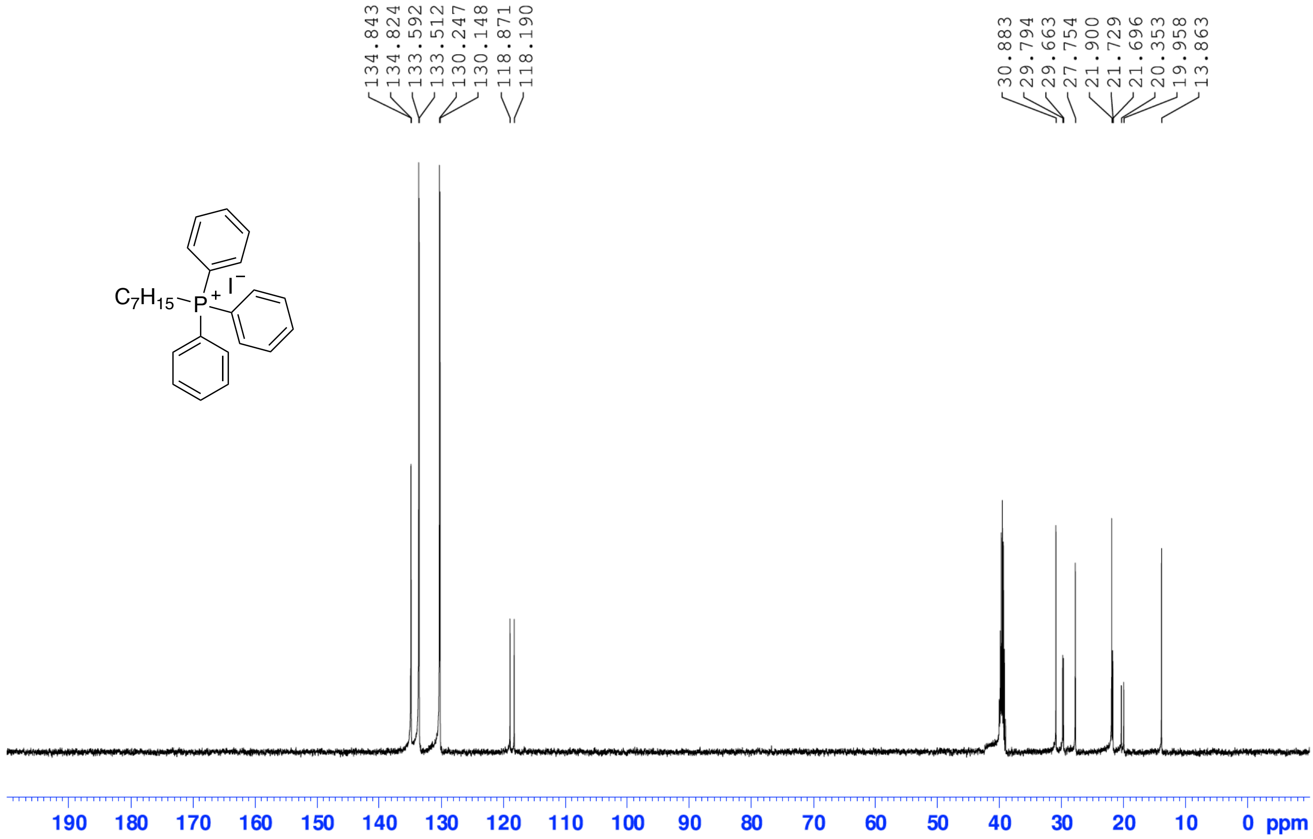

^{31}P NMR

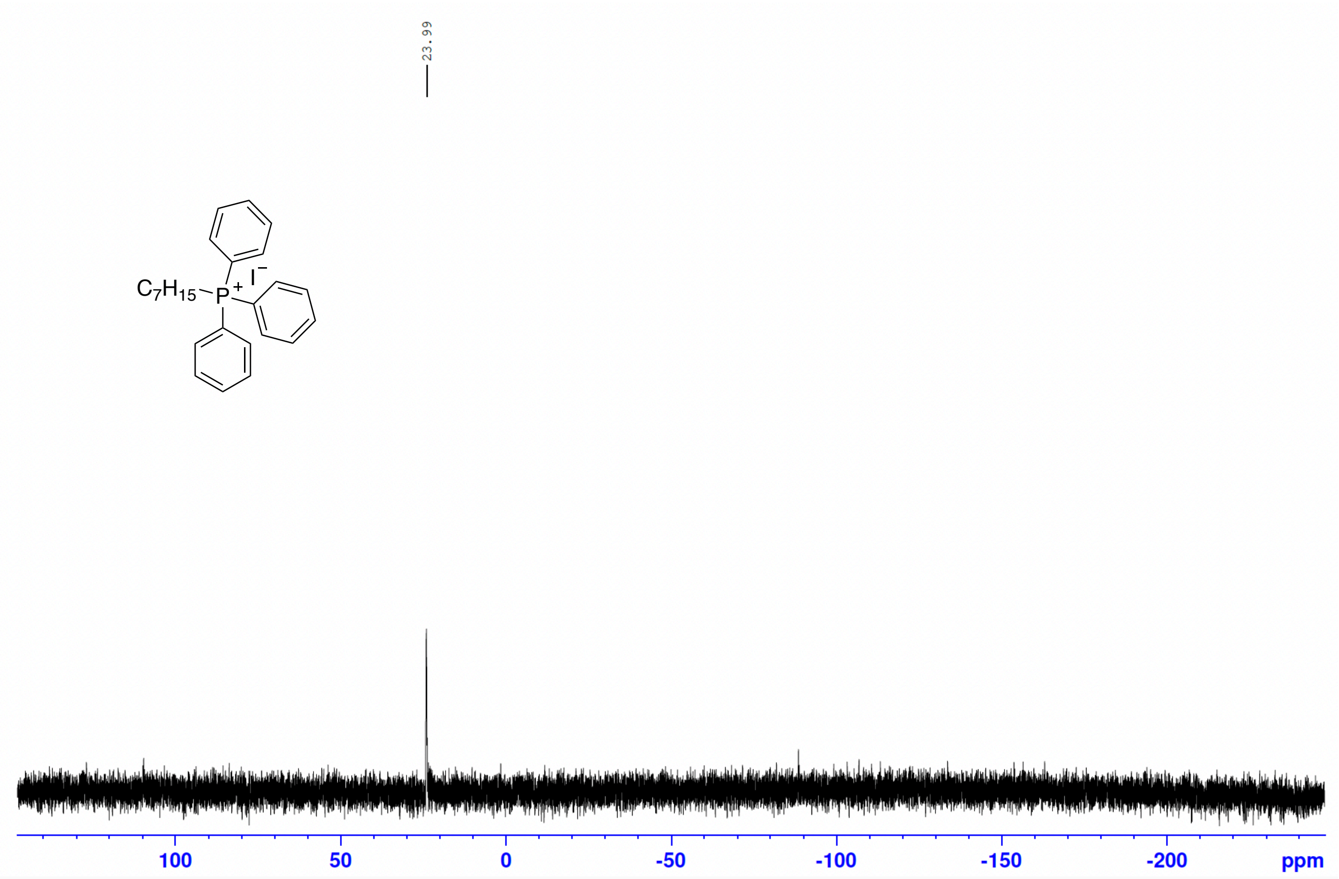

Triphenyl(undecyl)phosphonium iodide (**9**, $C_{29}H_{38}IP$)

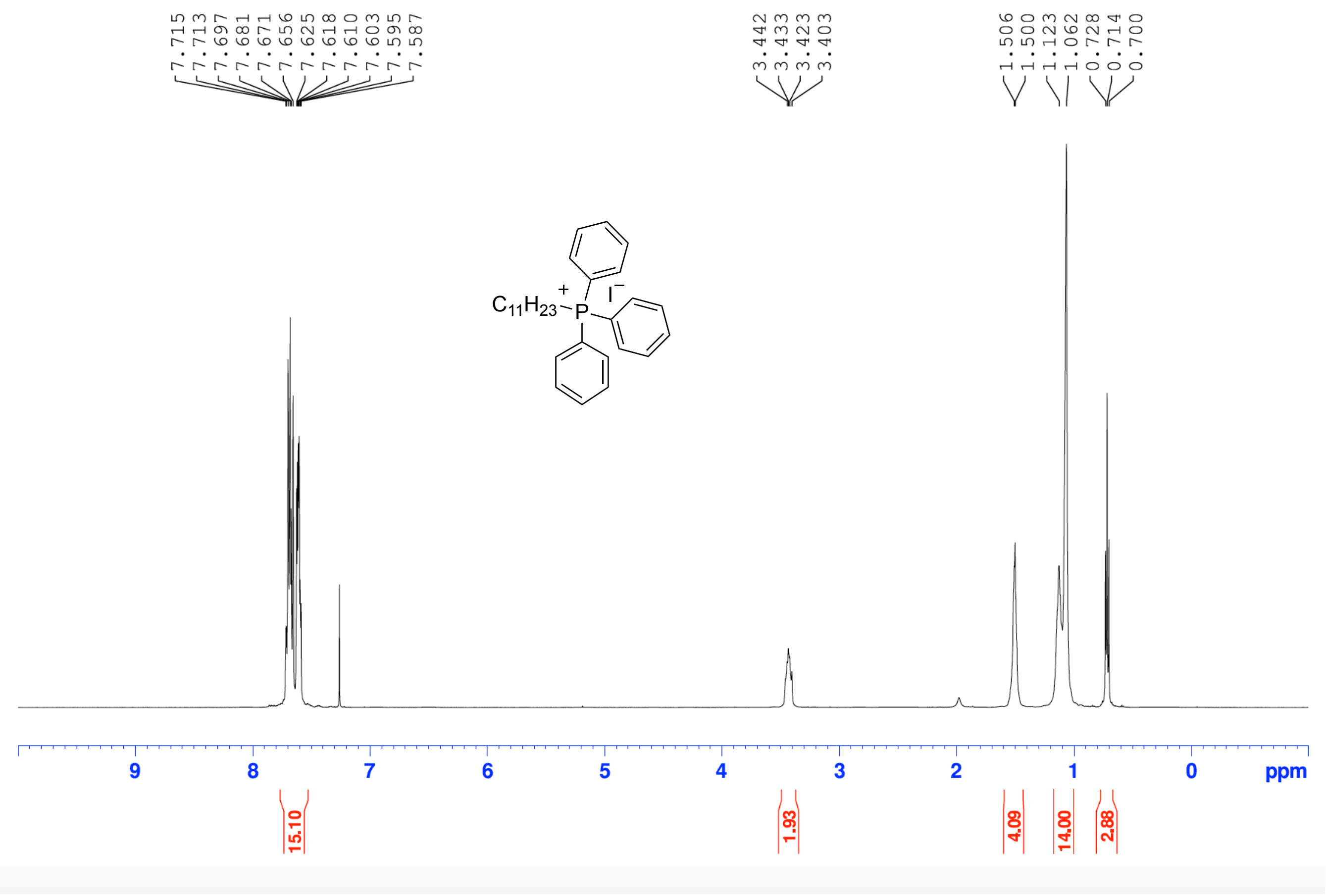

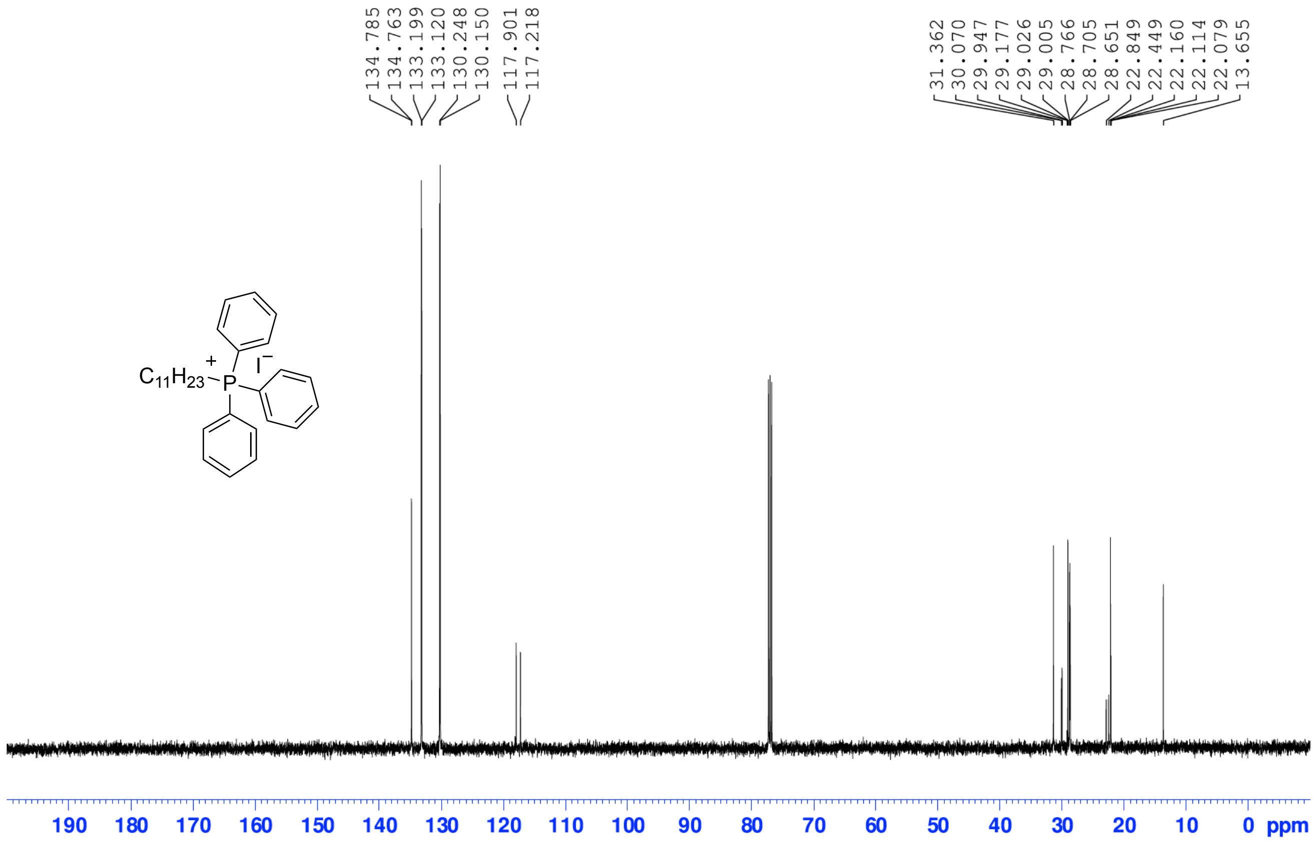
134.785
134.763
133.199
133.120
130.248
130.150
117.901
117.218
31.362
30.070
29.947
29.177
29.026
29.005
28.766
28.705
28.651
22.849
22.449
22.160
22.114
22.079
13.655
$C_{11}H_{23}$
P
I
ppm

^{31}P NMR

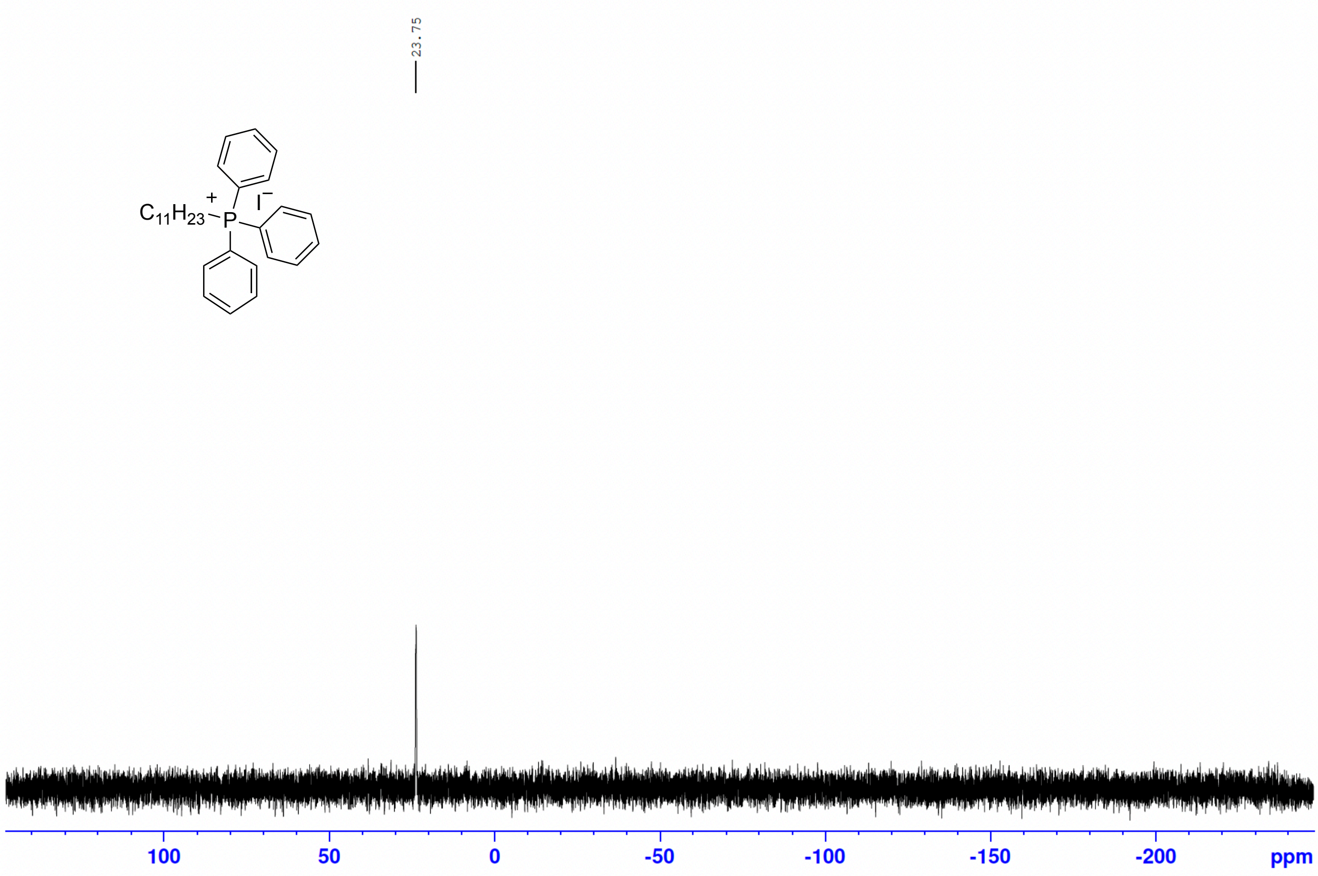
23.75
$C_{11}H_{23}$
P
I
ppm

[(2-Methylbenzimidazol-1-yl)heptyl]triphenylphosphonium iodide (**10**, $C_{33}H_{36}N_2IP$)

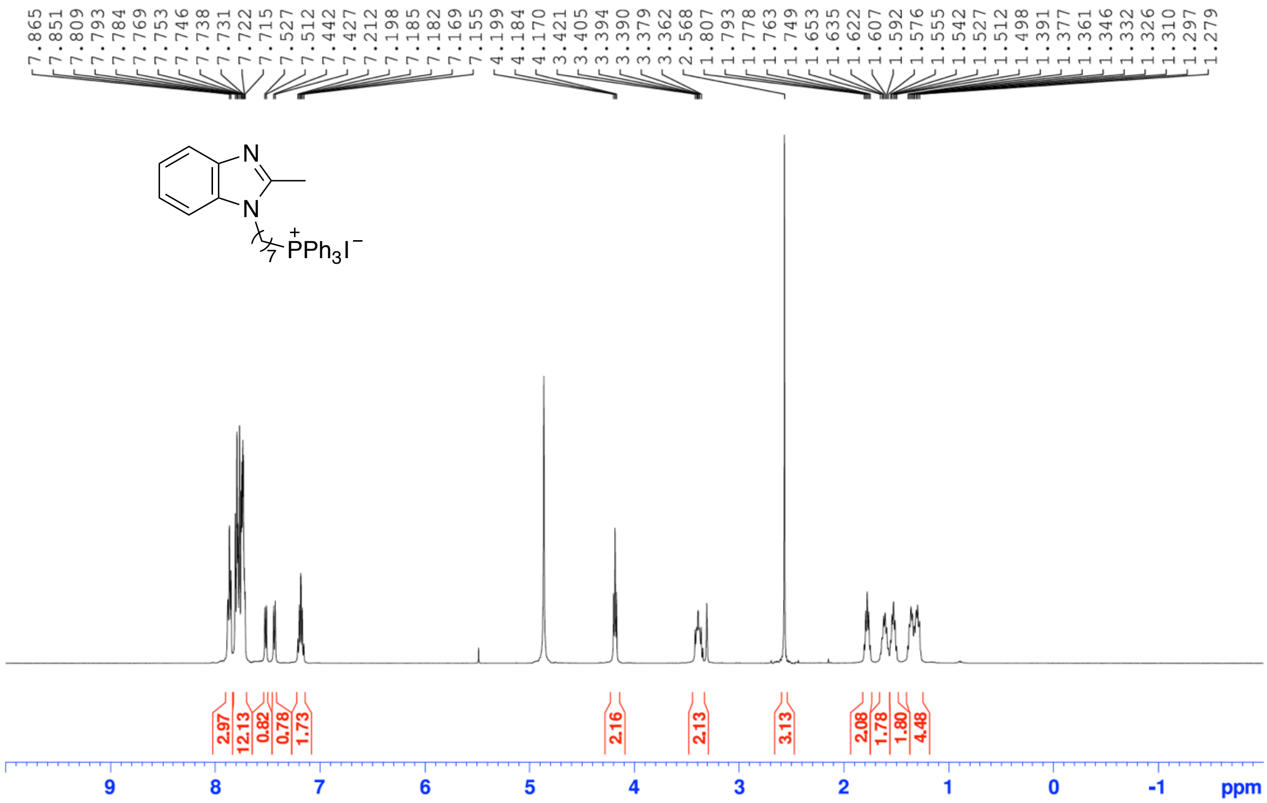

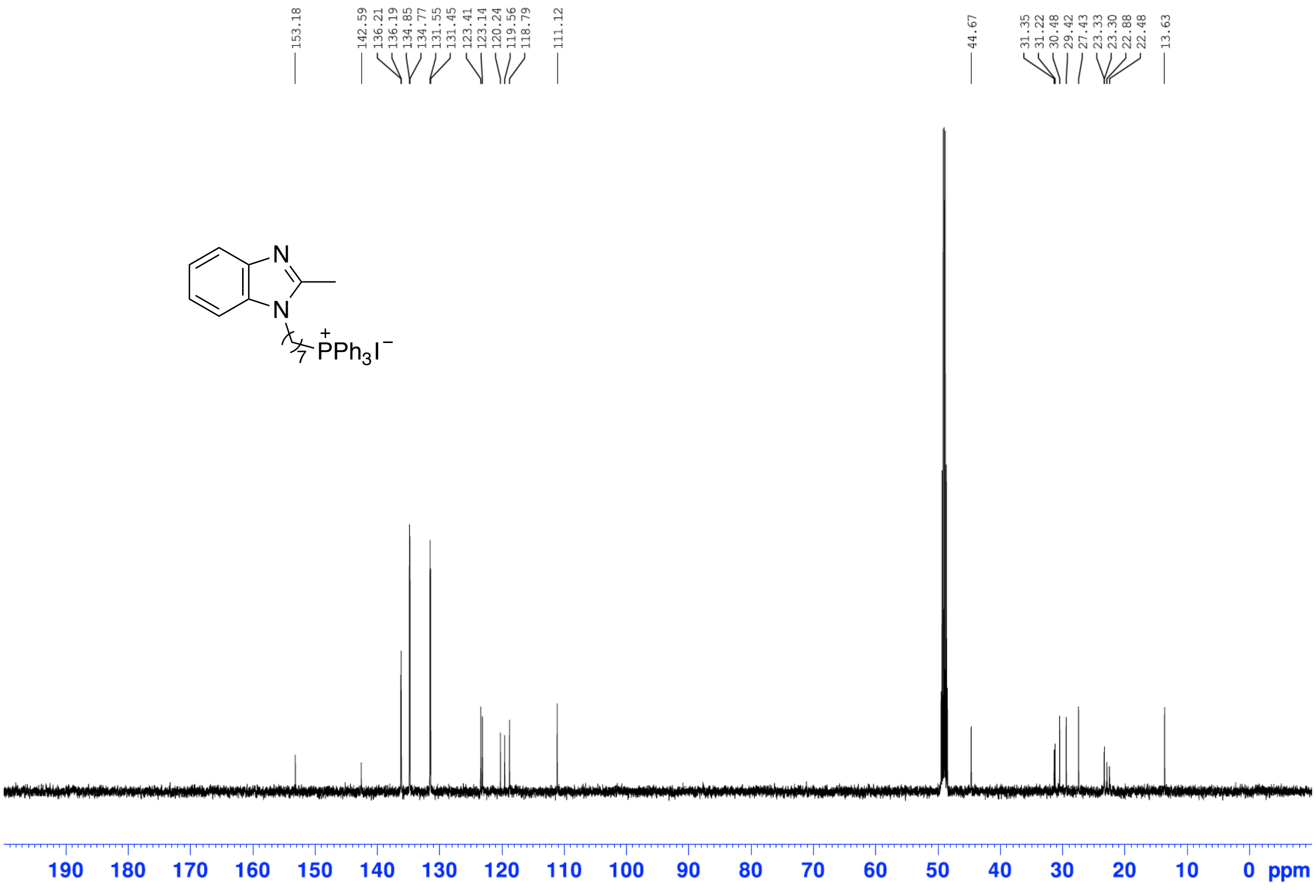

[(2-Methylbenzimidazol-1-yl)undecyl]triphenylphosphonium iodide (**11**, $C_{37}H_{44}N_2IP$)

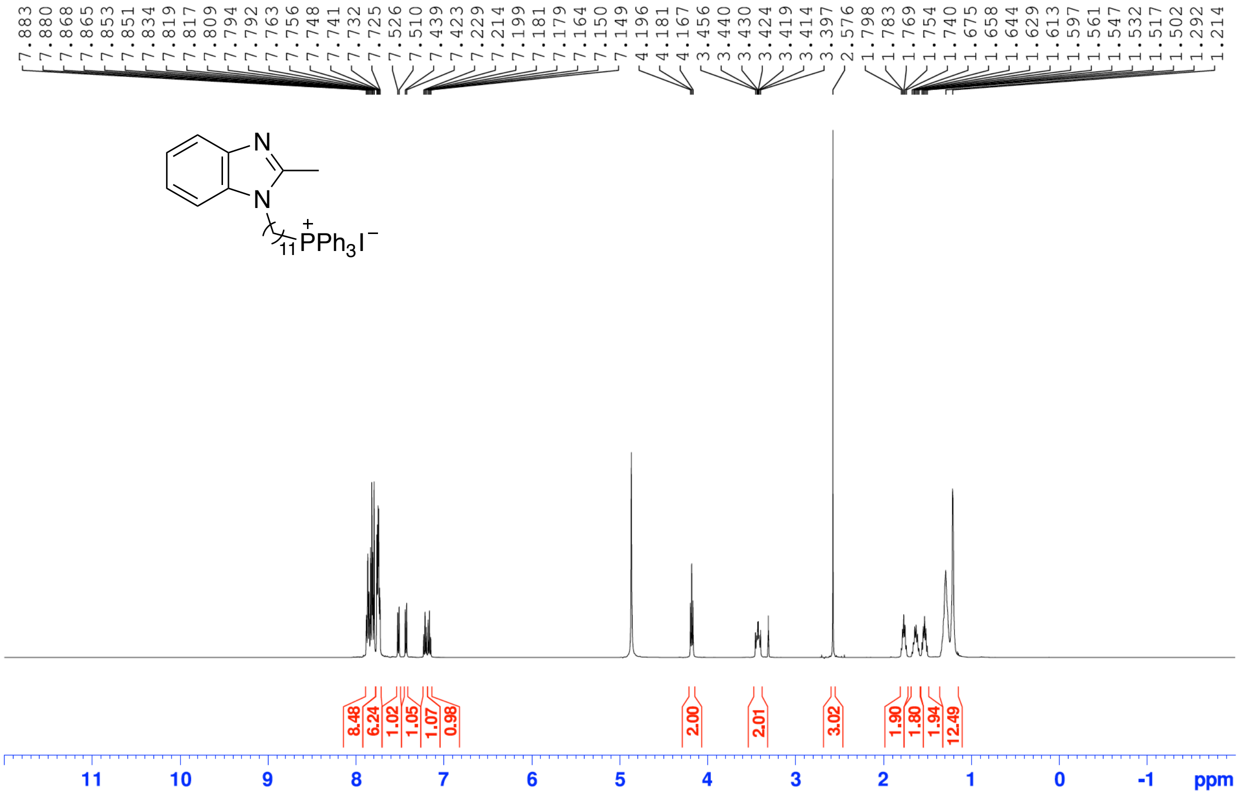

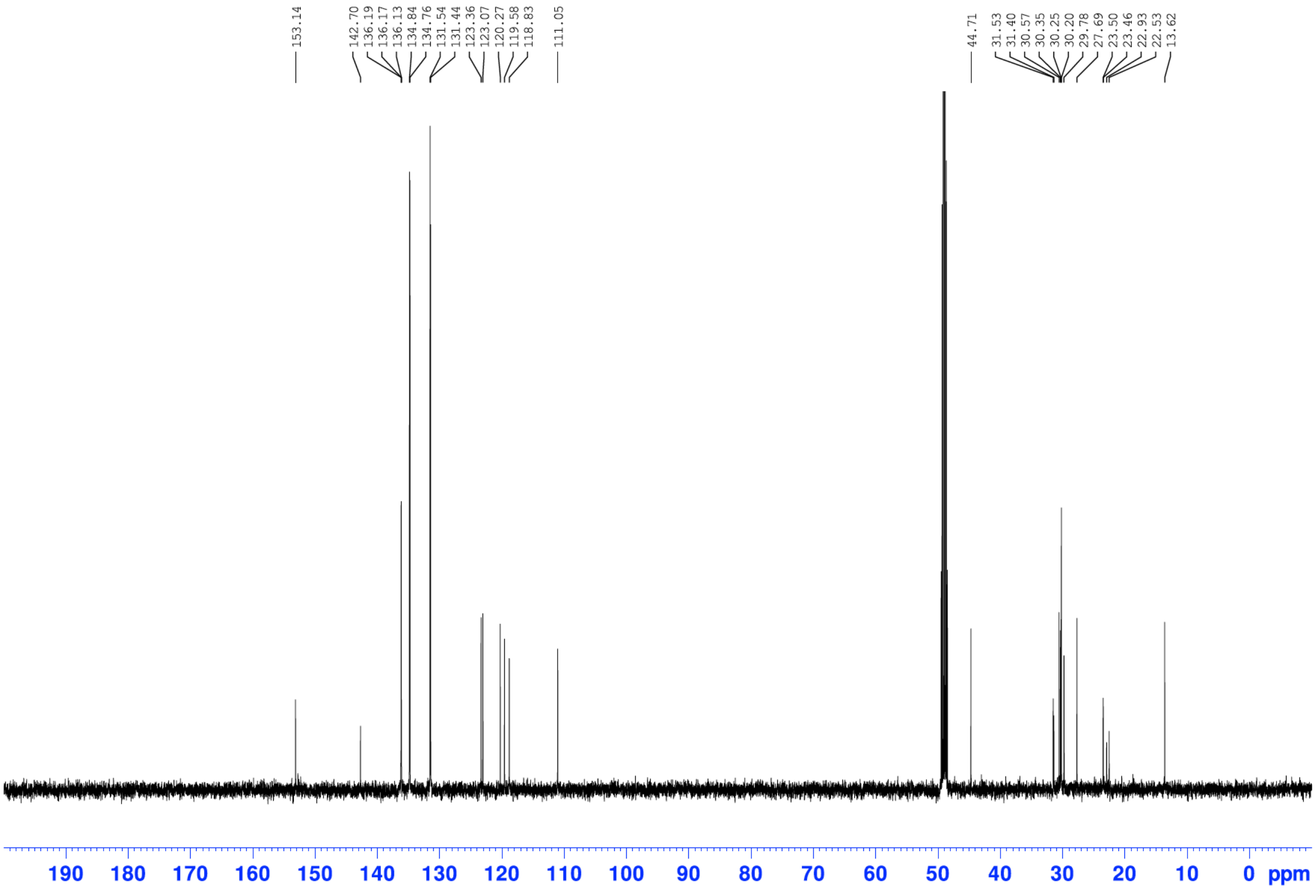

(7-(4-Methyl-2,5,10-trioxo-5,10-dihydrobenzo[*g*]quinolin-1(2*H*)-yl)heptyl)triphenylphosphonium iodide (**12**, $C_{39}H_{37}NO_3IP$)

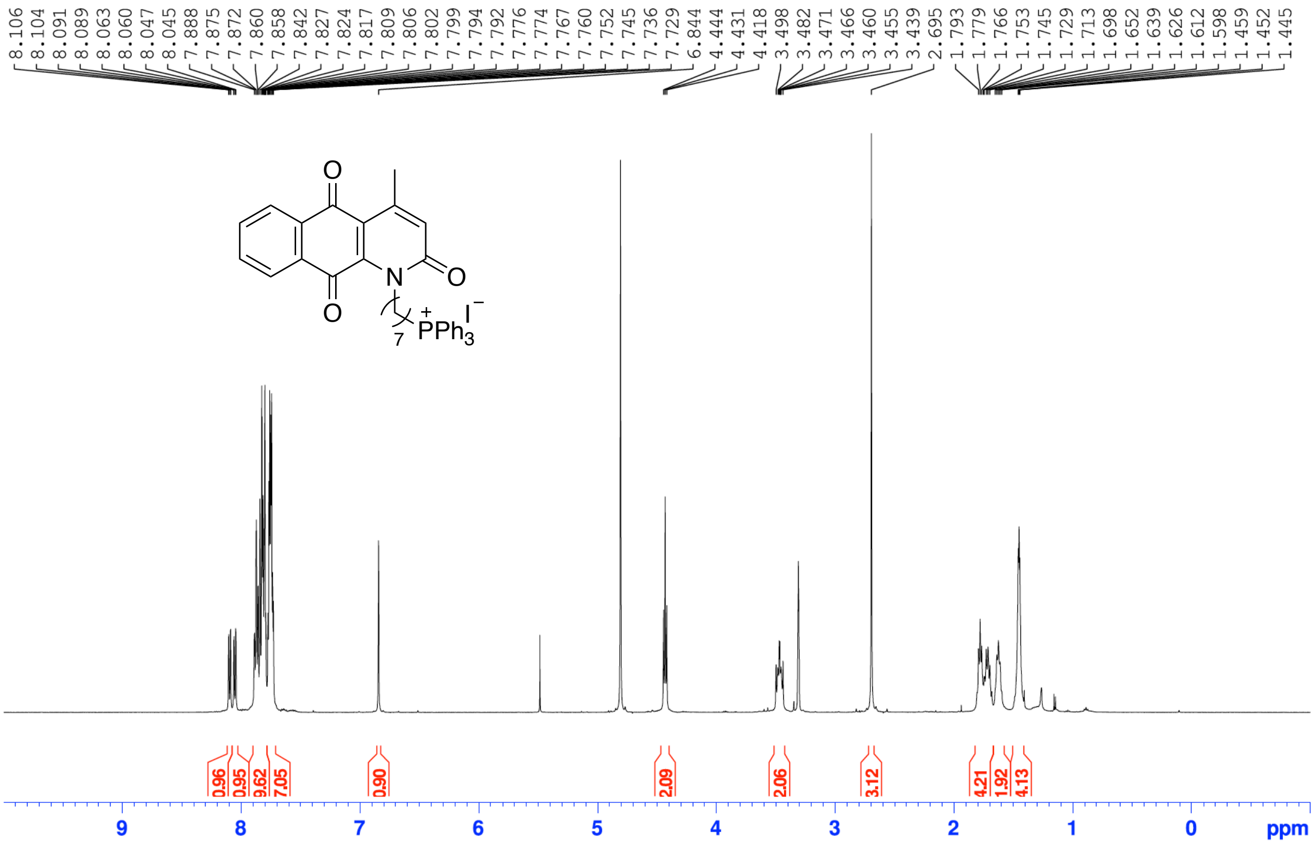

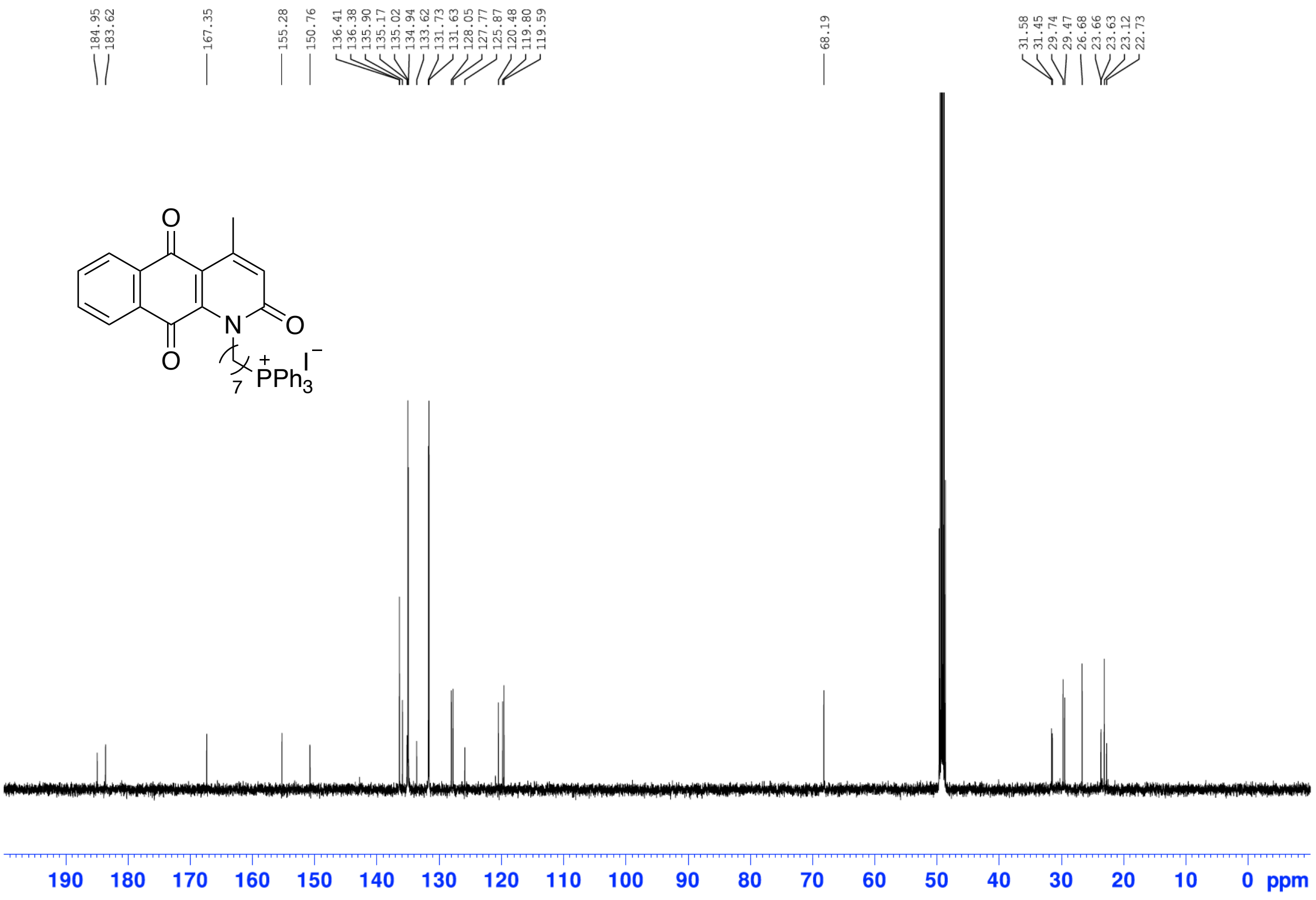

(11-(4-Methyl-2,5,10-trioxo-5,10-dihydrobenzo[*g*]quinolin-1(2*H*)-yl)undecyl)triphenylphosphonium iodide (**13**, $C_{43}H_{45}NO_3IP$)

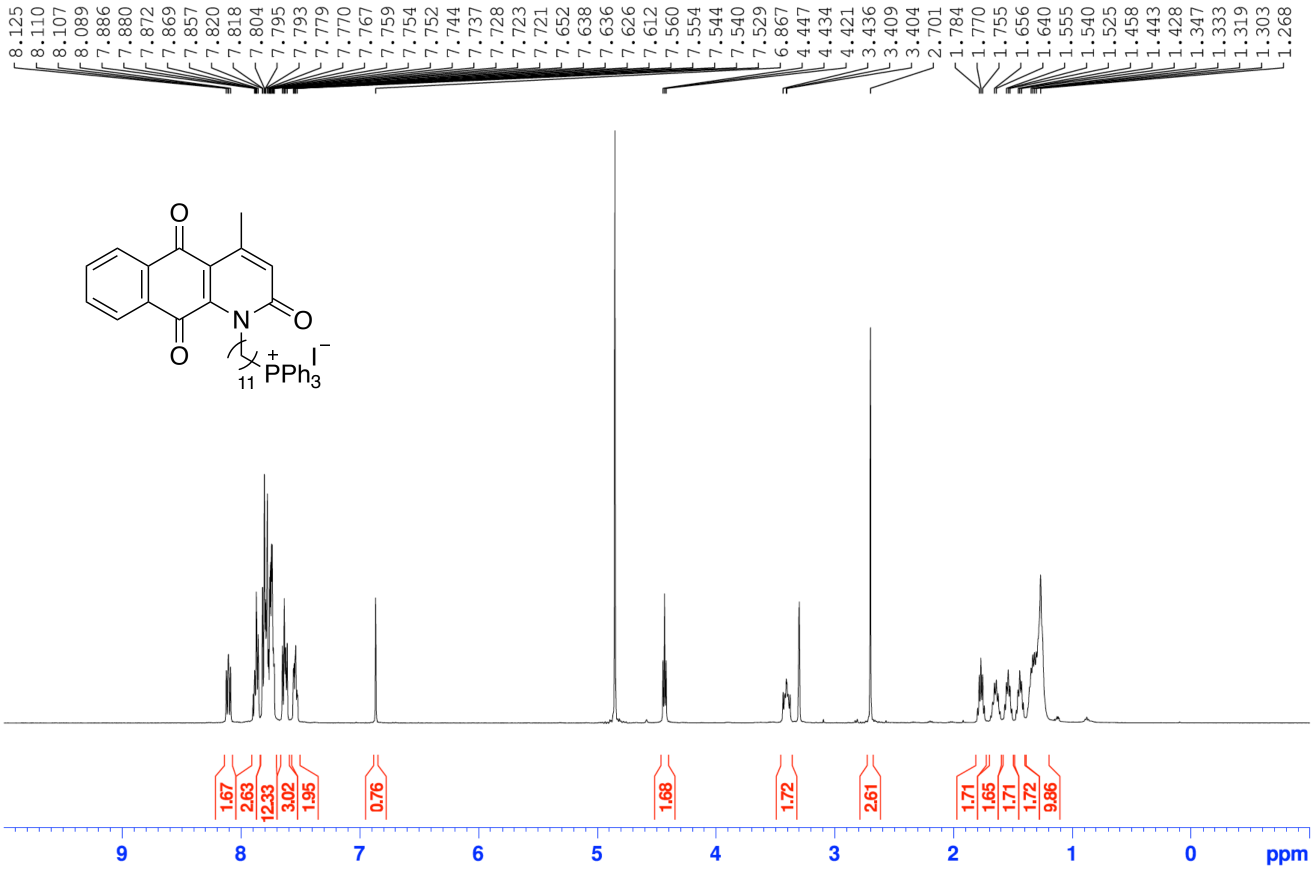

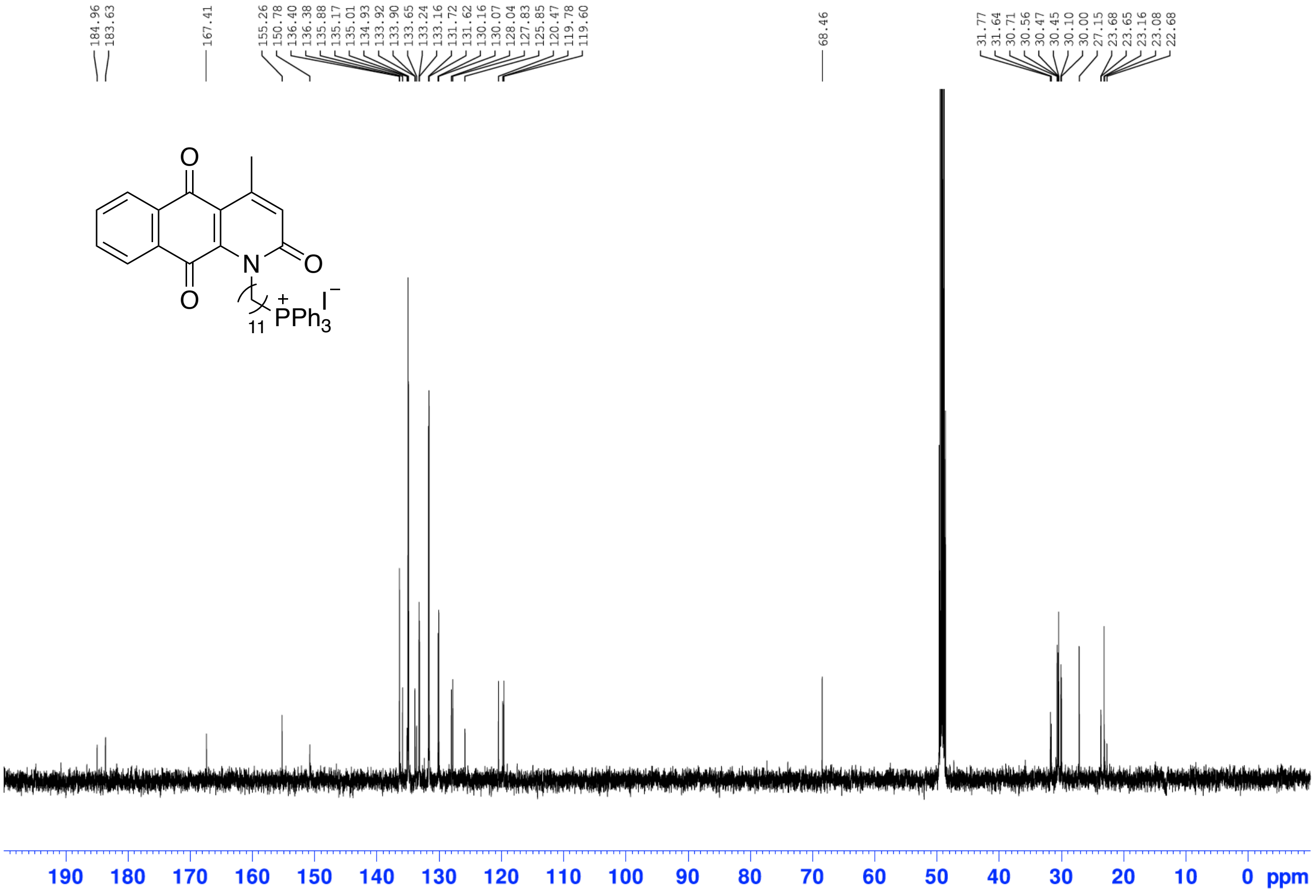

{11-[4,9-Dioxo-2-(trifluoromethyl)naphtho[2,3-d]imidazole-1-yl]undecyl}triphenylphosphonium iodide (**14**, $C_{41}H_{41}F_3N_2O_2IP$)

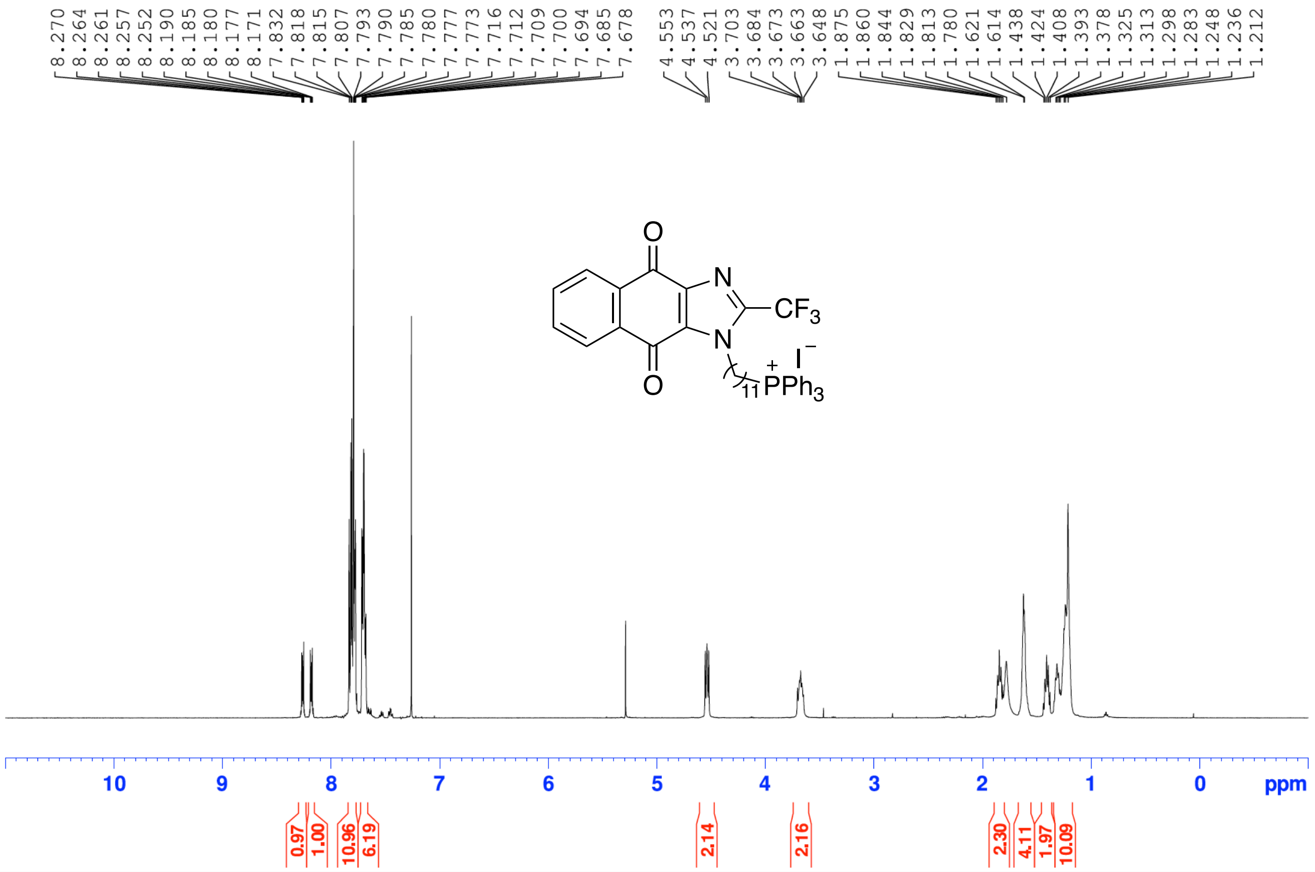

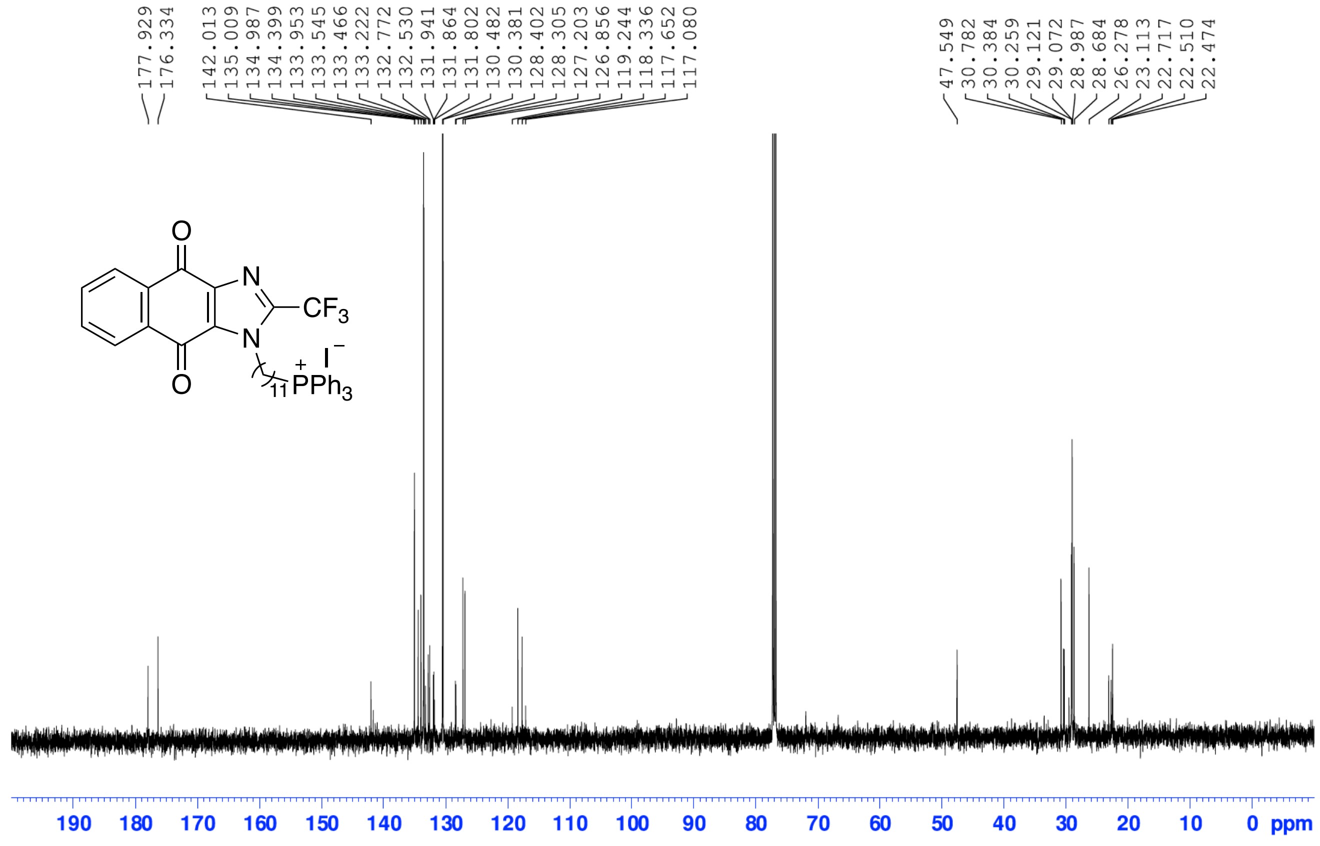

(11-{2-Methyl-4,9-dioxoimidazo[4,5-*g*]quinolin-1-yl}undecyl)triphenylphosphonium iodide (**15**, $C_{40}H_{43}N_3O_2IP$)

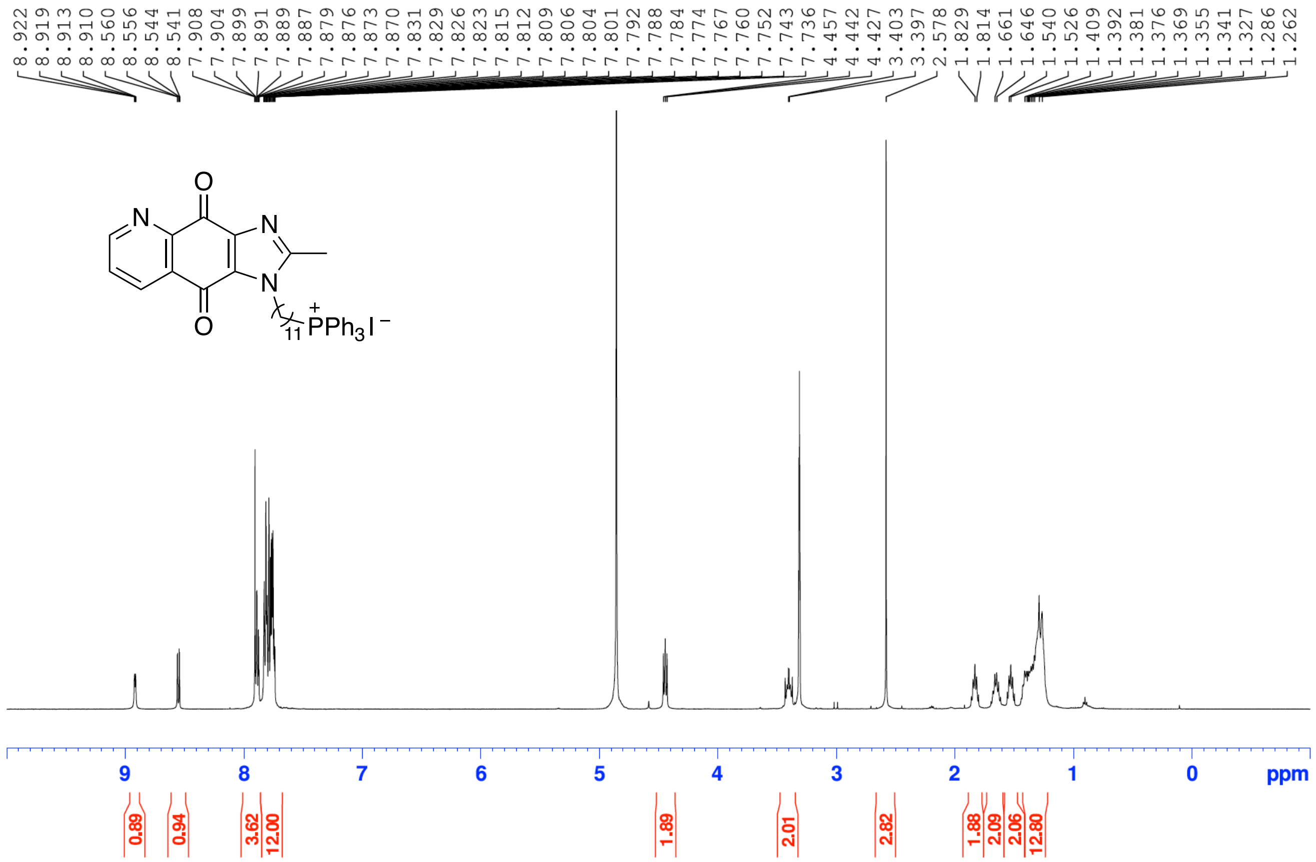

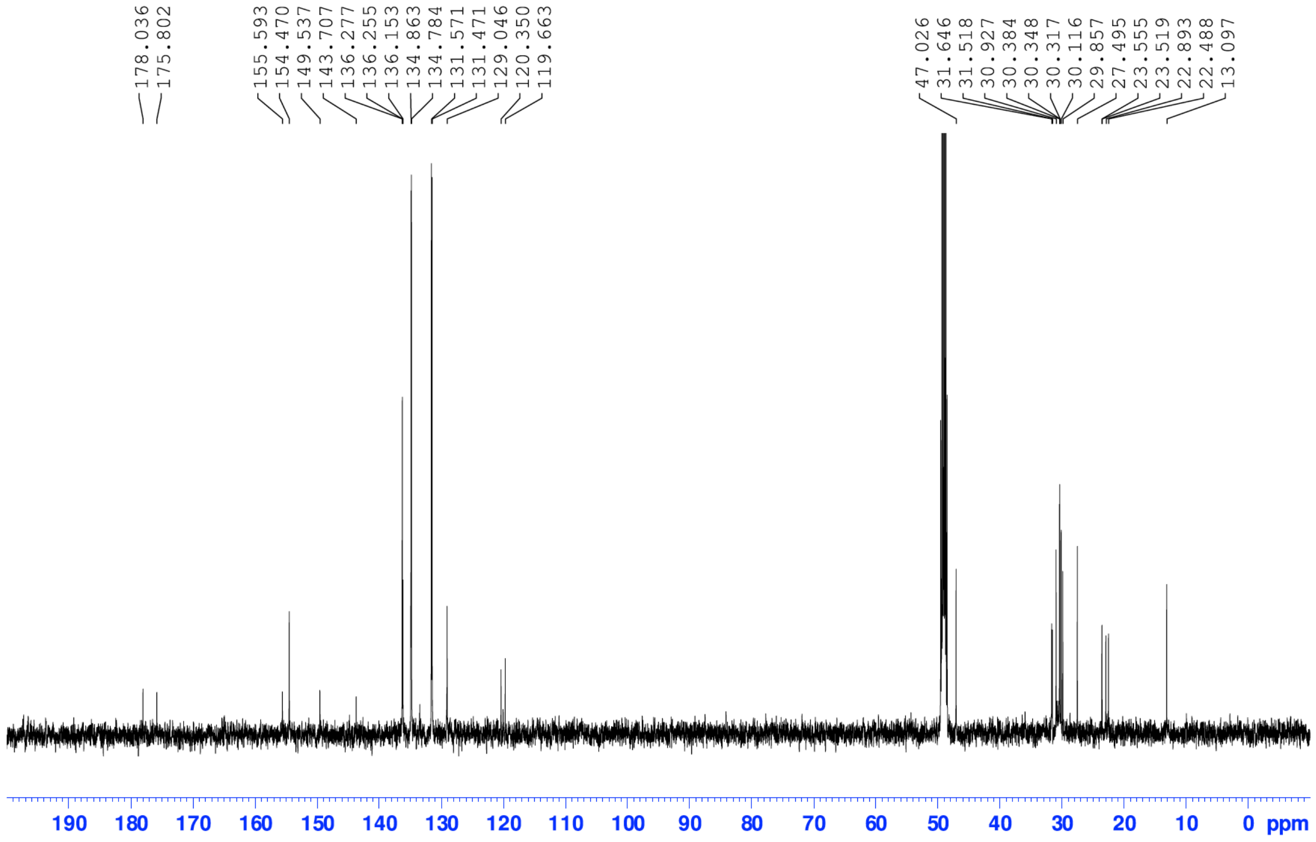

(7-{2-Methyl-4,9-dioxonaphtho[2,3-*d*]imidazol-1-yl}heptyl)pyridinium iodide (**16**, $C_{24}H_{26}N_3O_2I$)

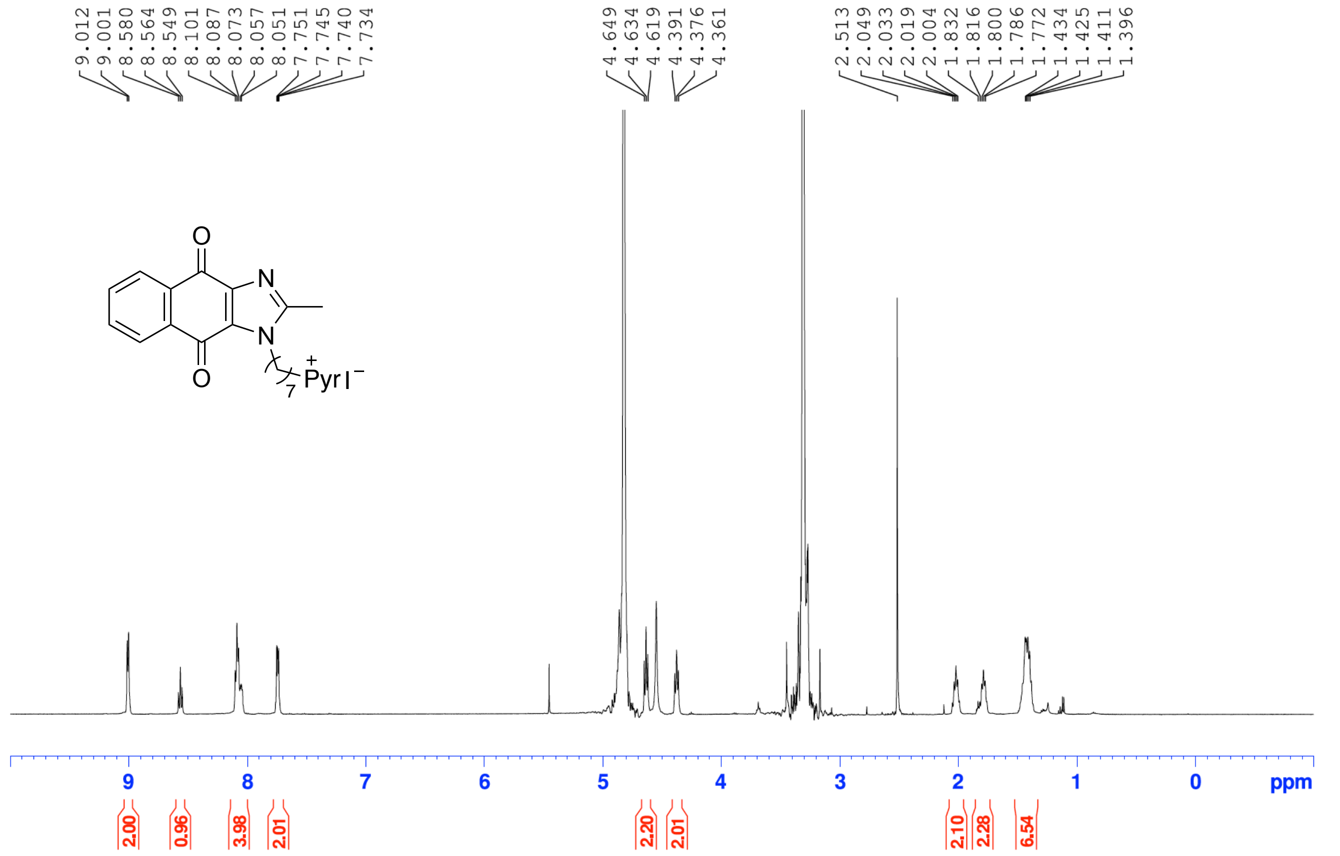

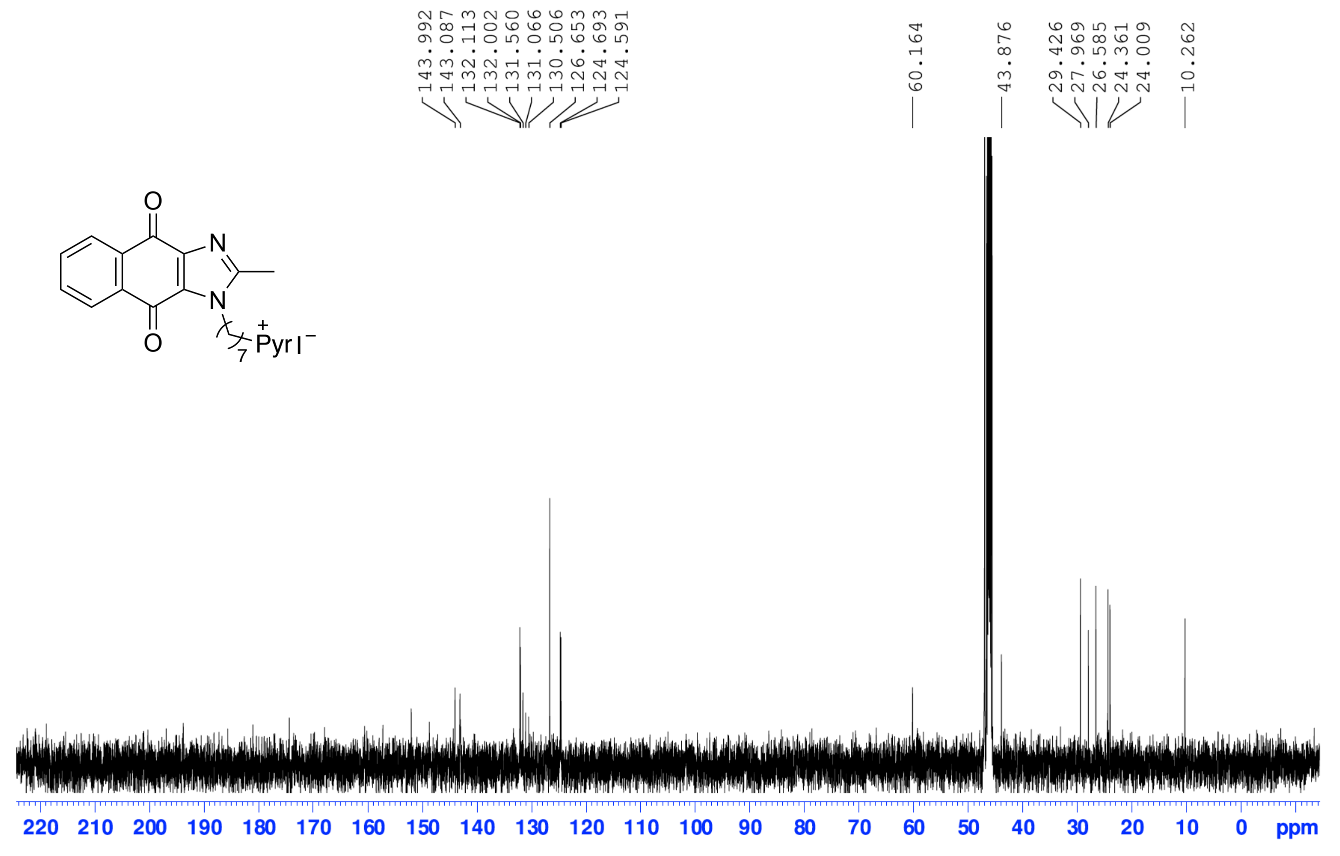

(7-{2-Methyl-4,9-dioxonaphtho[2,3-*d*]imidazol-1-yl}heptyl)tricyclohexylphosphonium iodide (**17**, $C_{37}H_{54}N_2O_2IP$)

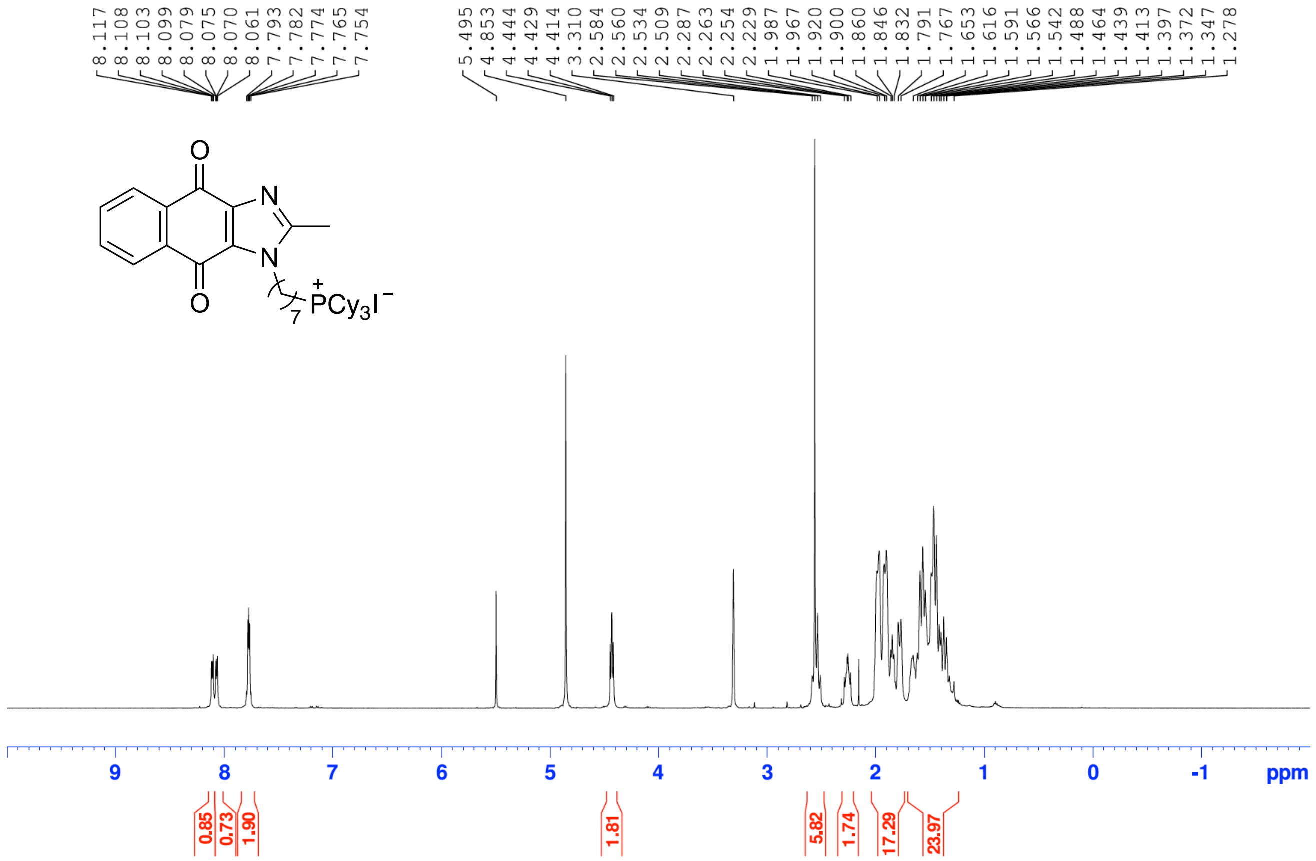

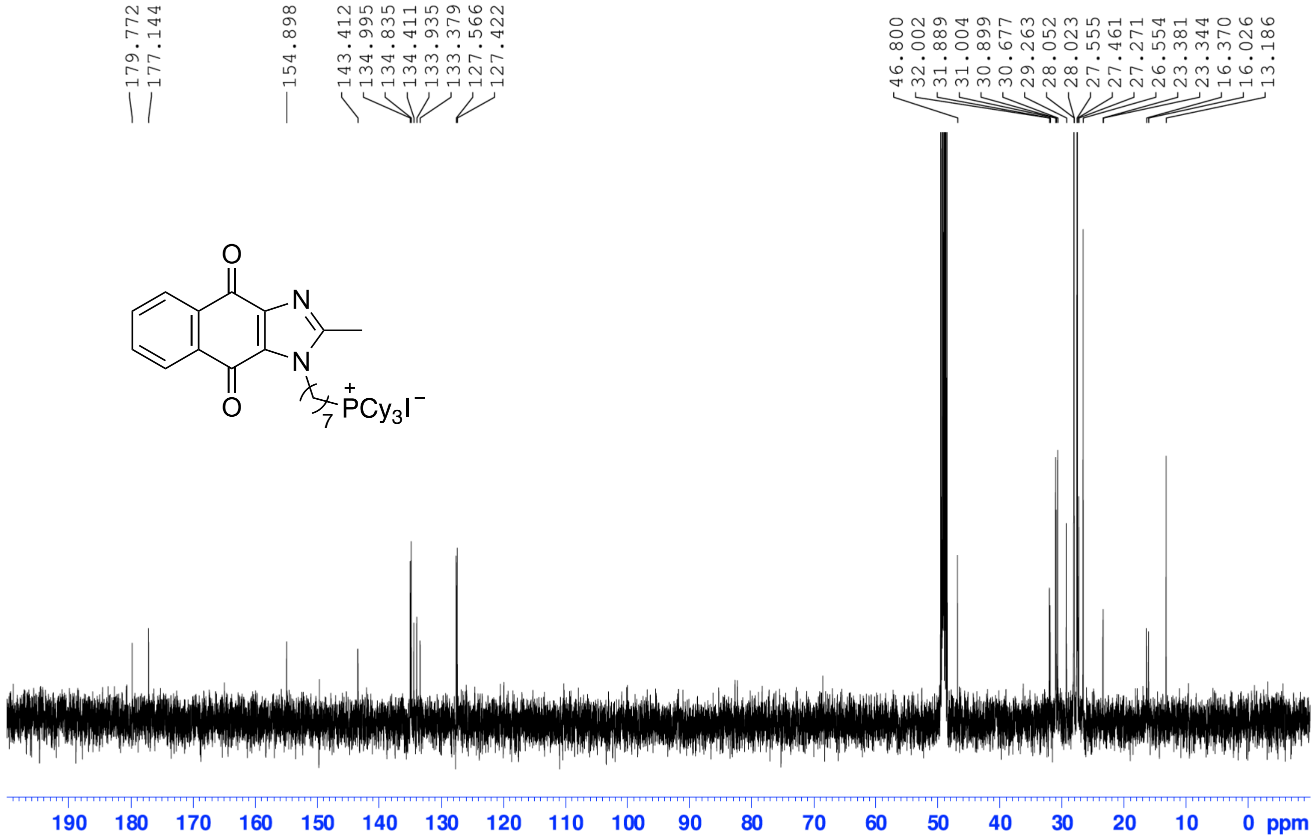

(11-{2-Methyl-4,9-dioxonaphtho[2,3-*d*]imidazol-1-yl}undecyl)tricyclohexylphosphonium iodide (**18**, $C_{41}H_{62}N_2O_2IP$)

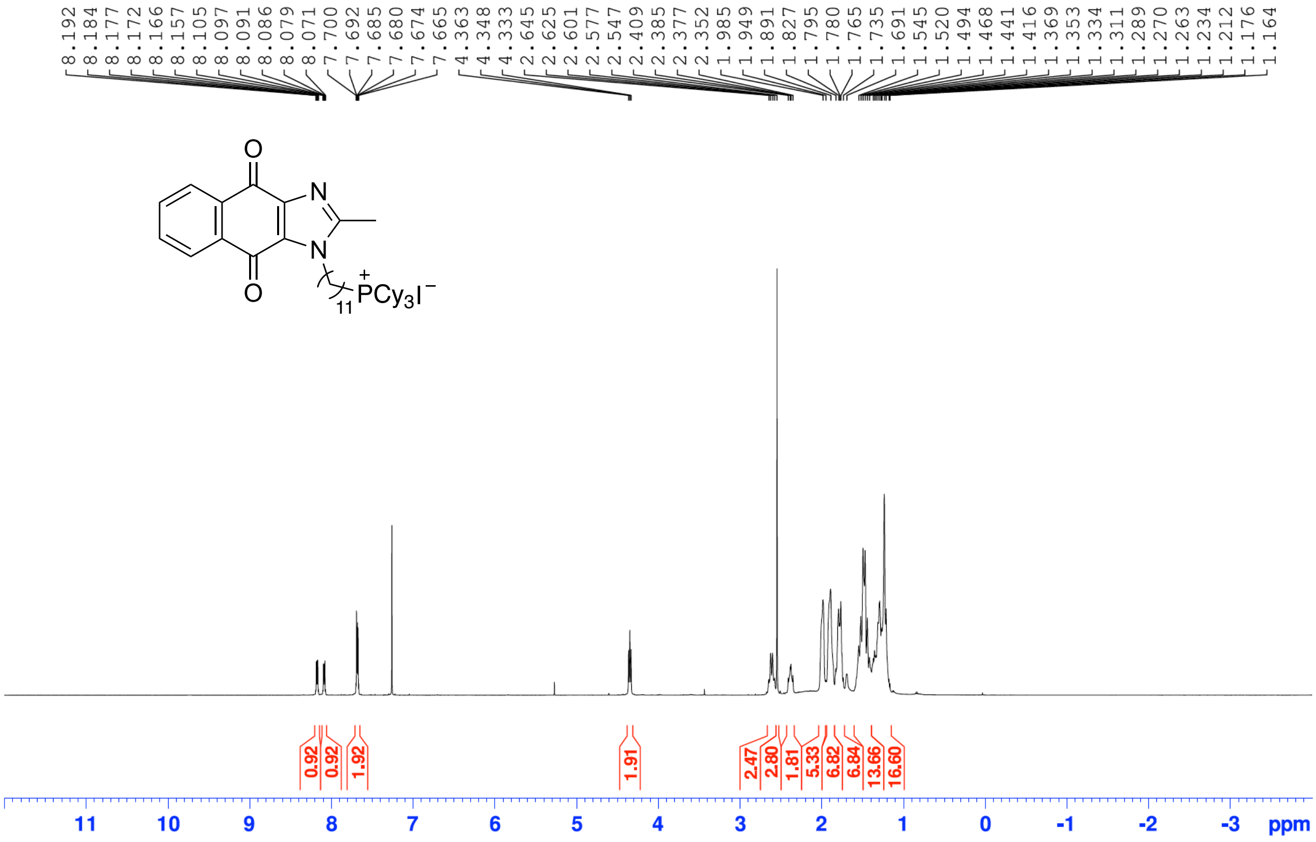

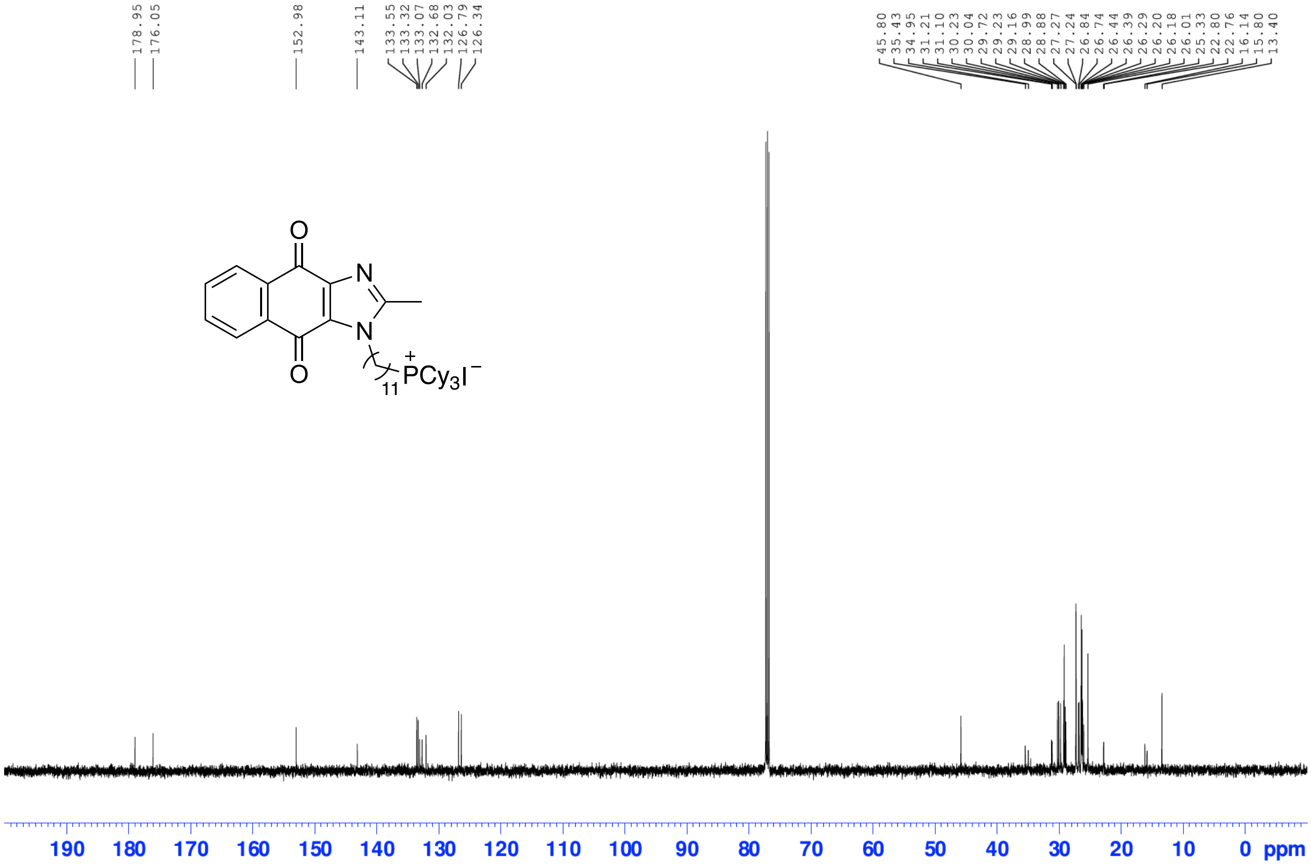

(7-{2-Methyl-4,9-dioxonaphtho[2,3-*d*]imidazol-1-yl}heptyl)trihexylphosphonium iodide (**19**, $C_{37}H_{60}N_2O_2IP$)

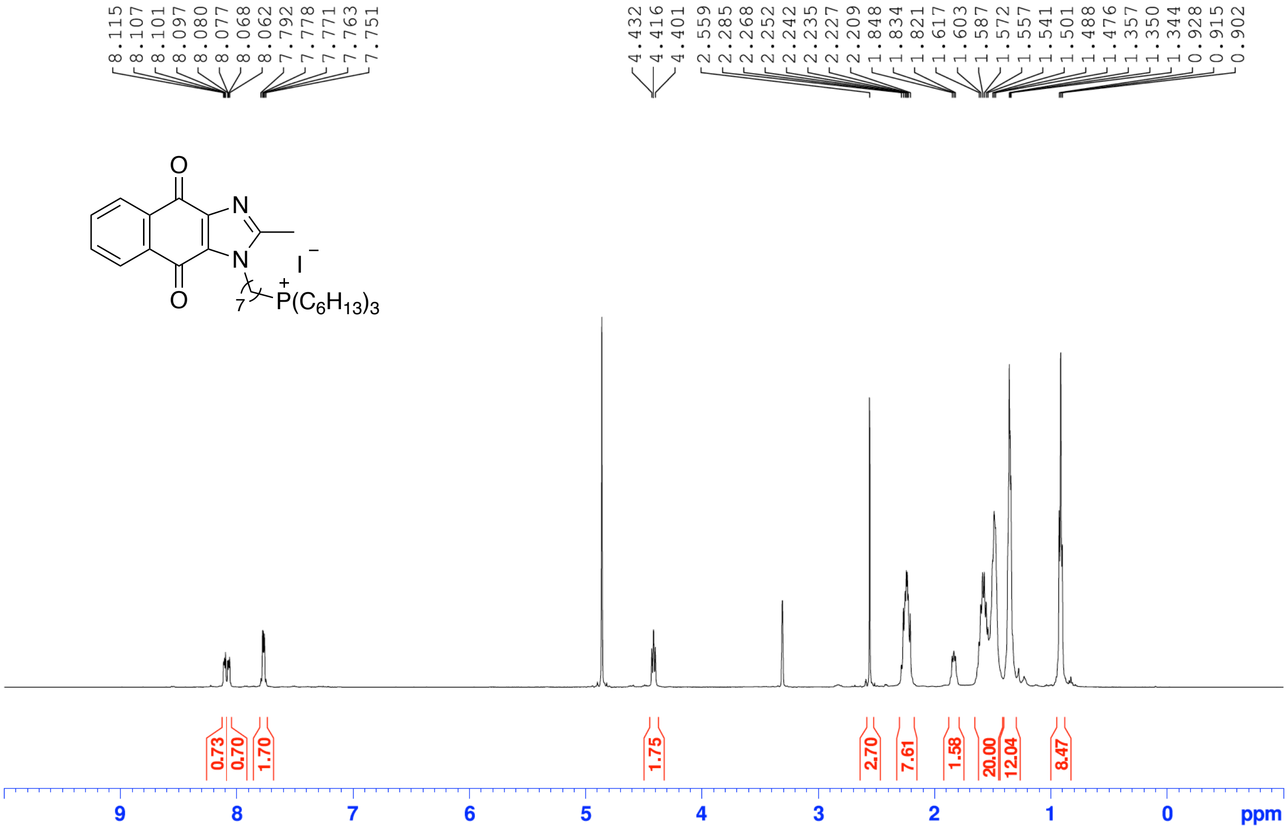

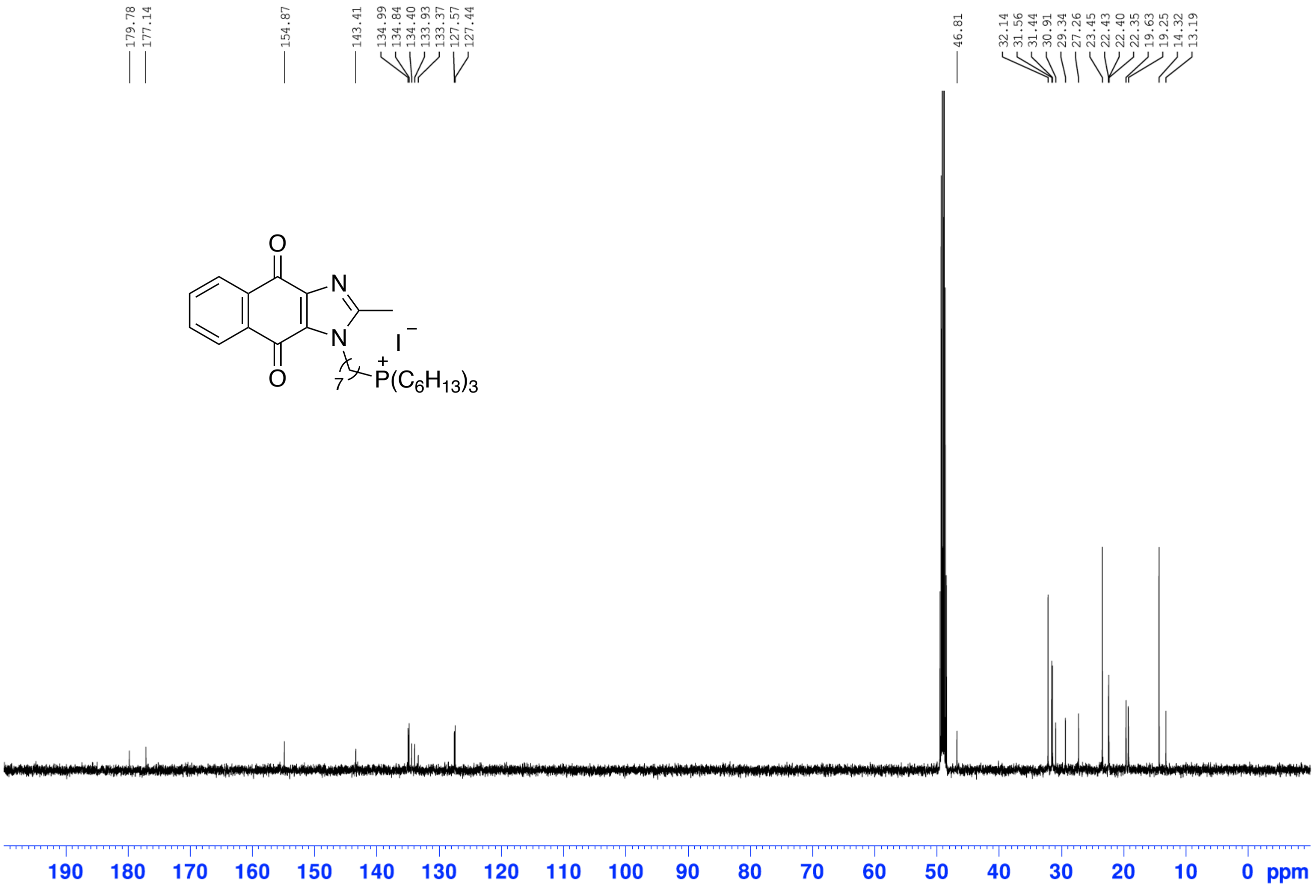

N-(3-Chloropropyl)-2-methylnaphtho[2,3-*d*]imidazole-4,9-dione (**23**, $C_{15}H_{13}N_2O_2Cl$)

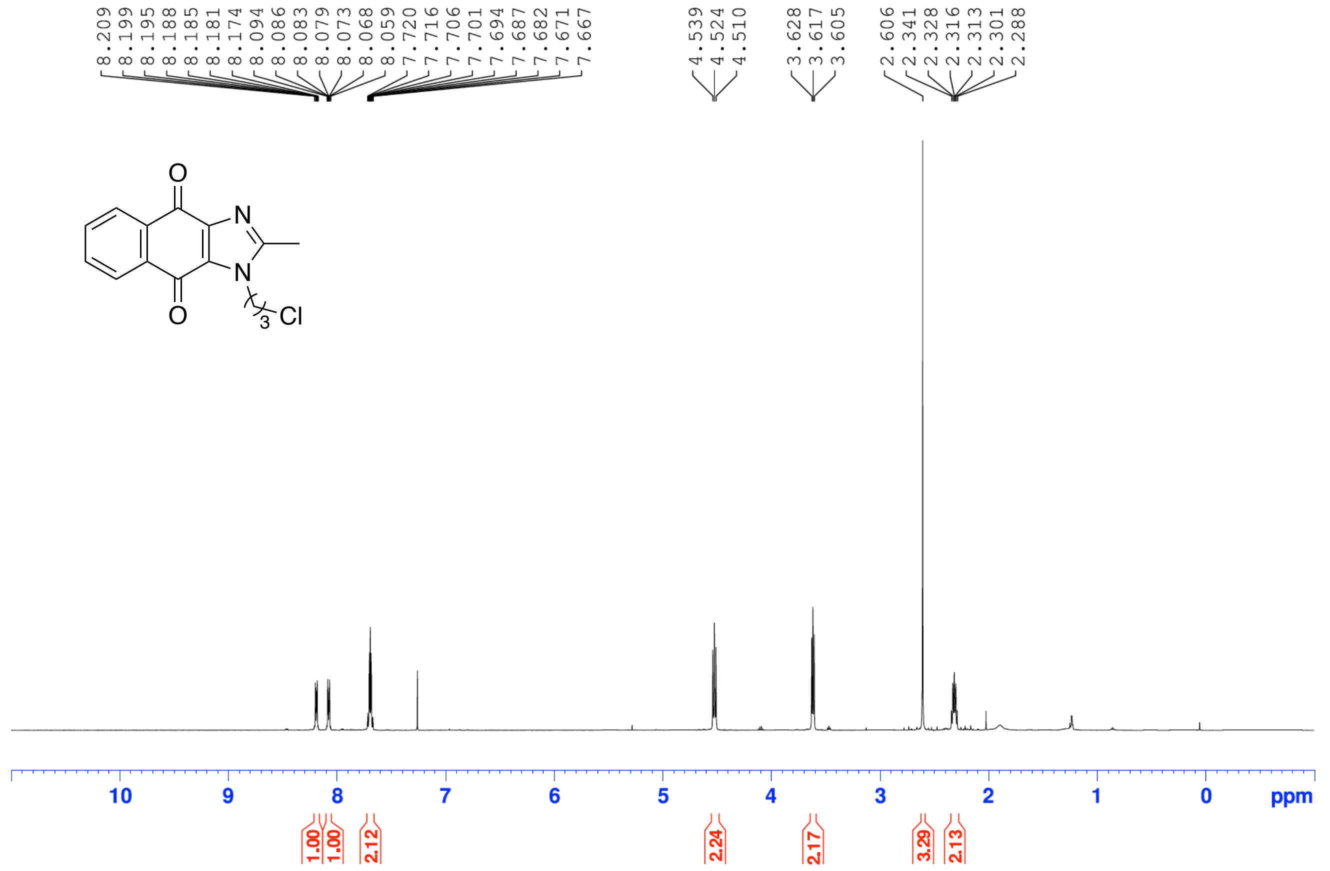

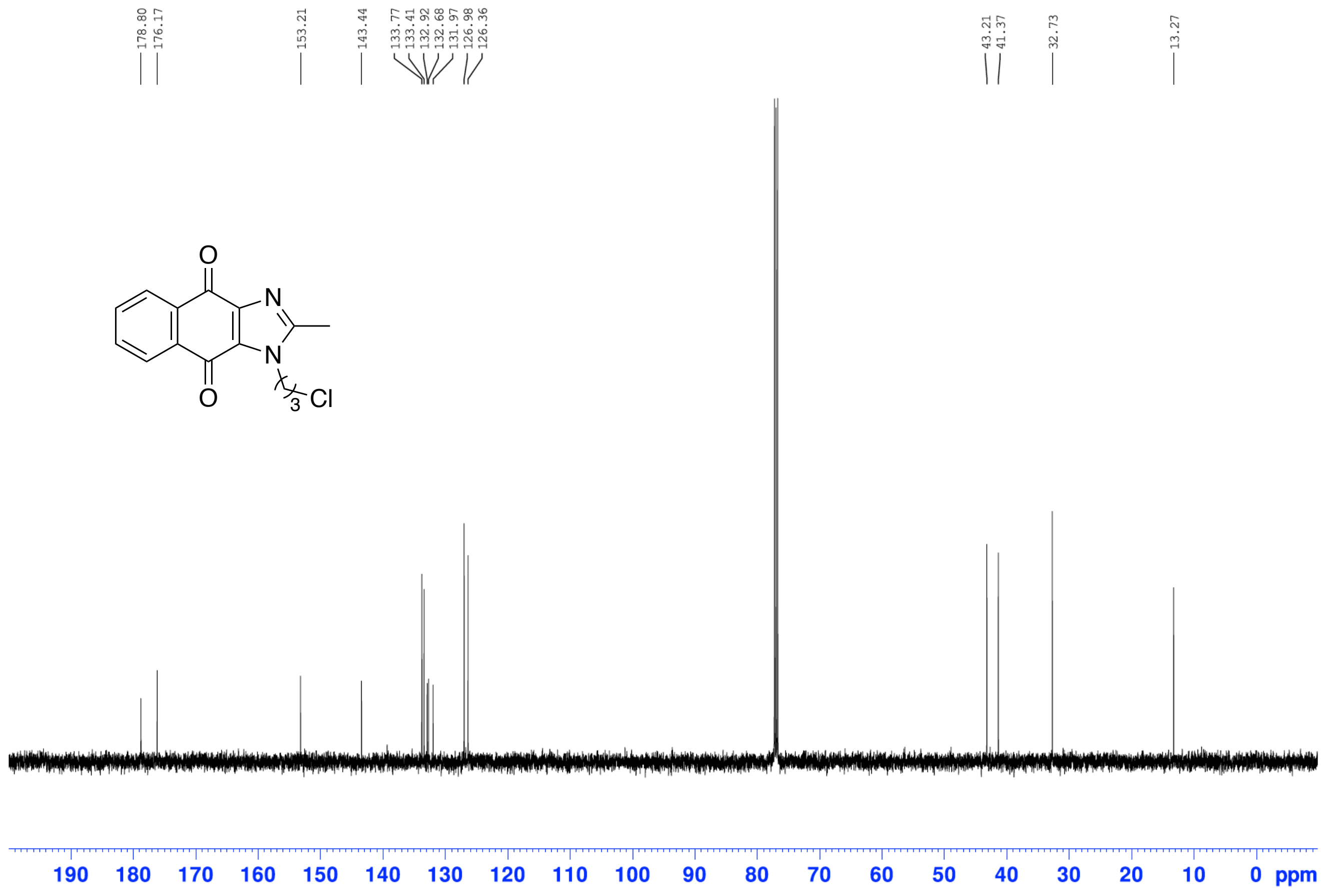

N-(5-Chloropentyl)-2-methylnaphtho[2,3-*d*]imidazole-4,9-dione (**24**, $C_{17}H_{17}N_2O_2Cl$)

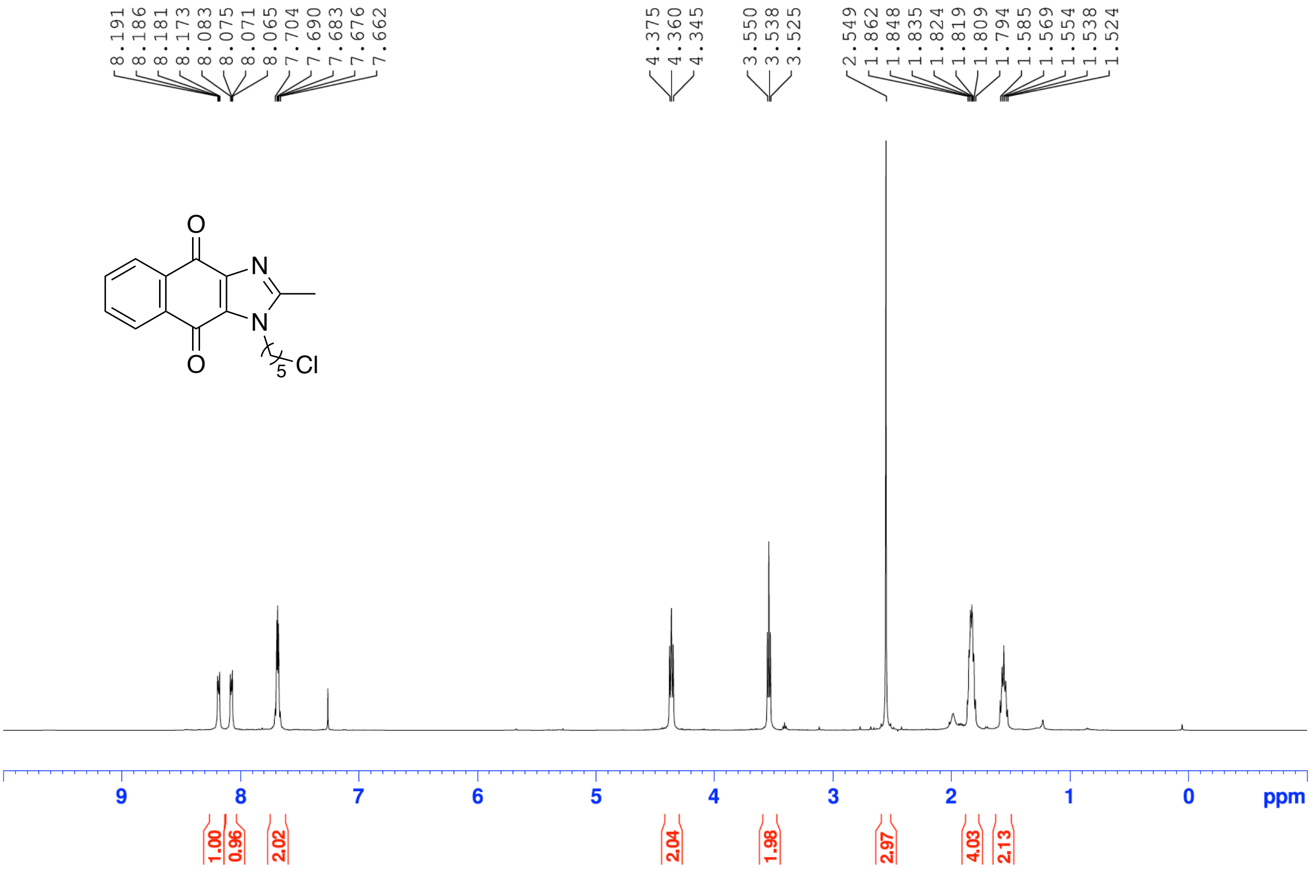

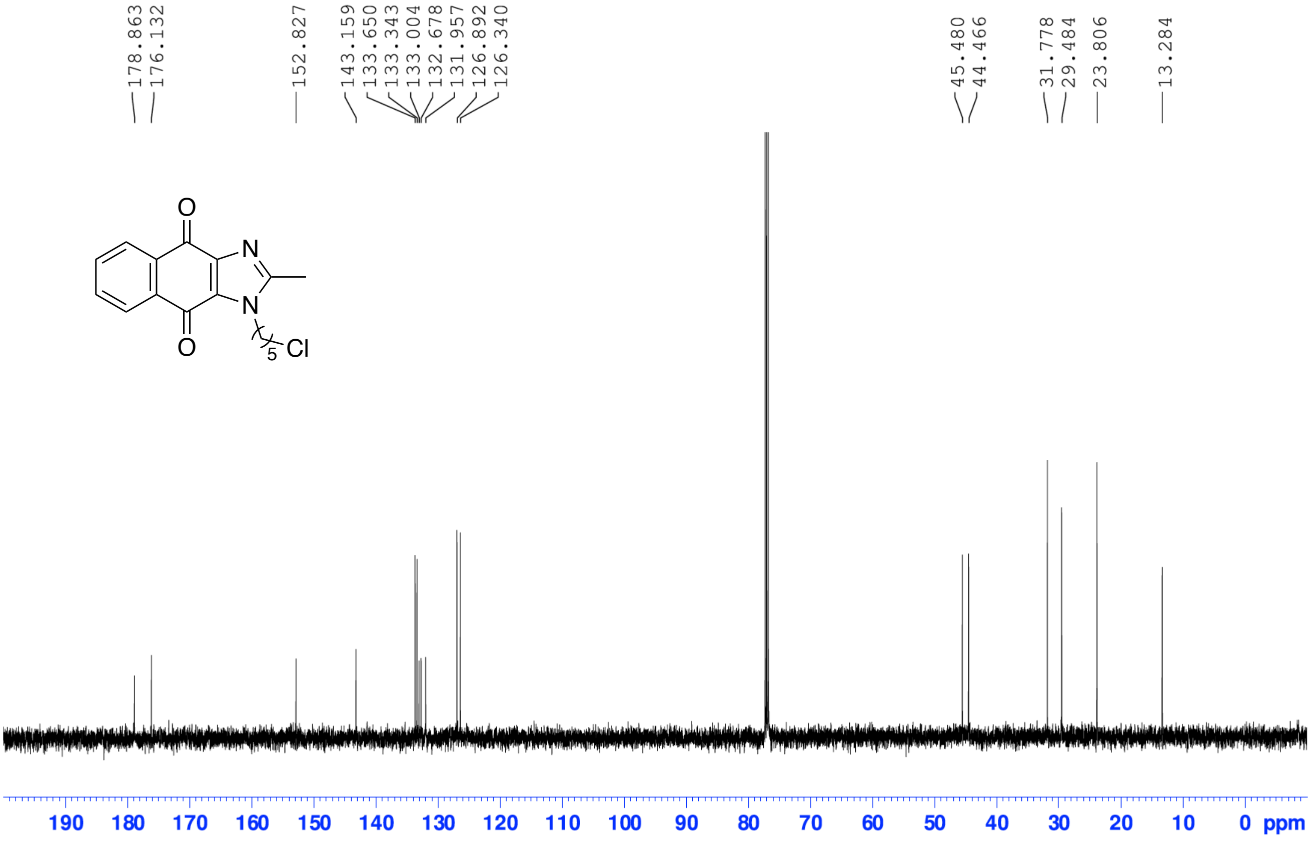

N-(7-Chloroheptyl)-2-methylnaphtho[2,3-*d*]imidazole-4,9-dione (**25**, $C_{19}H_{21}N_2O_2Cl$)

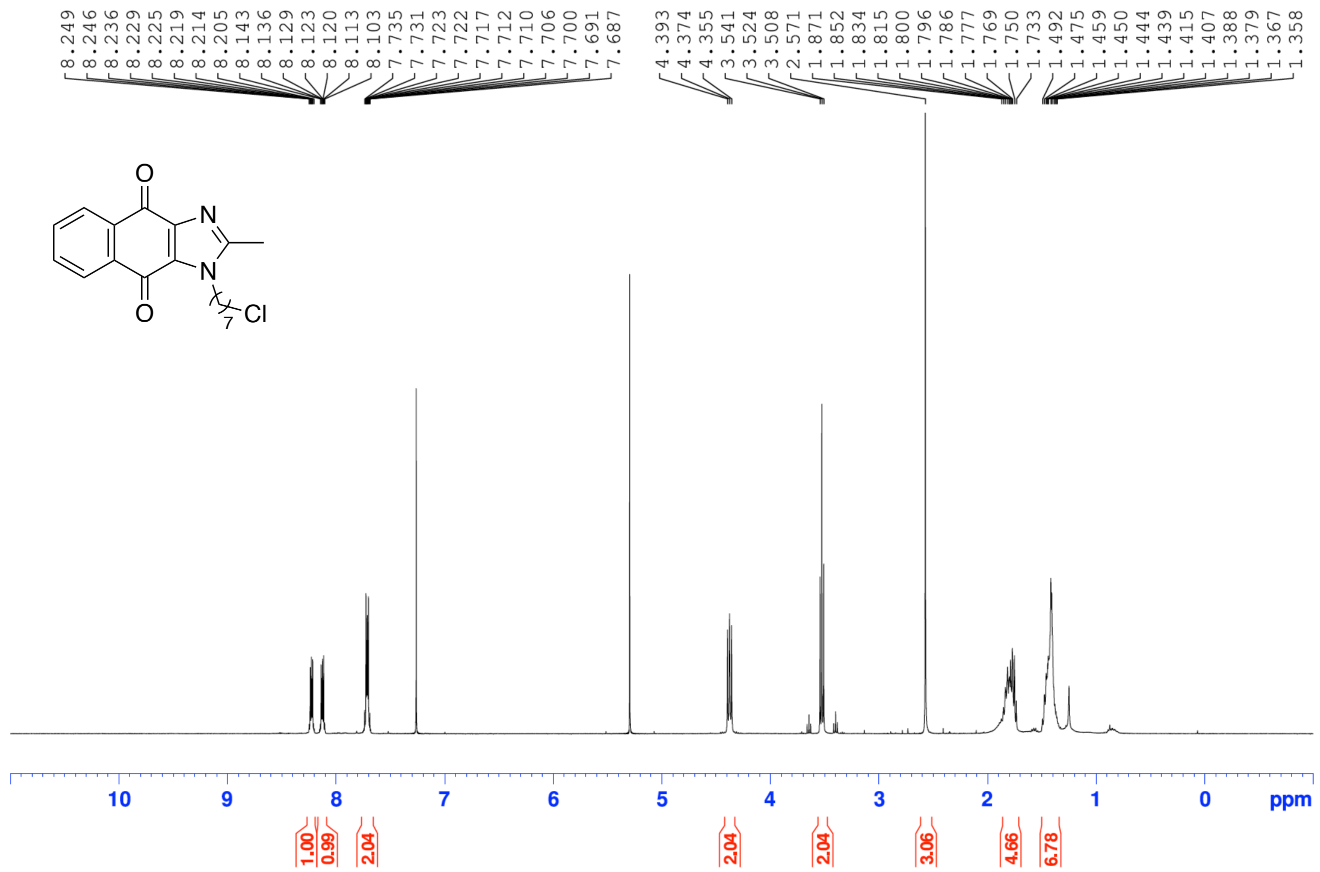

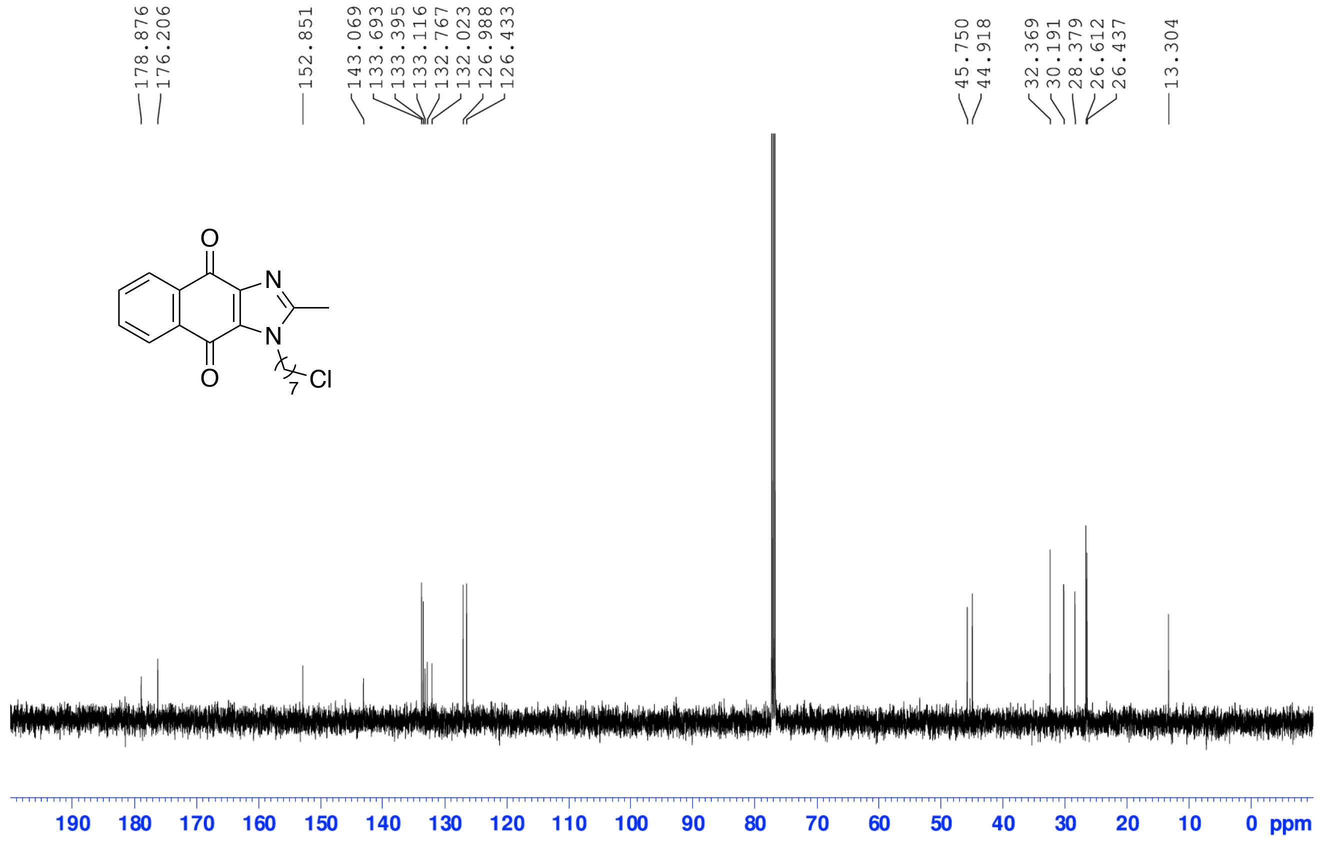

1-(9-Chlorononyl)-2-methyl-1*H*-naphtho[2,3-*d*]imidazole-4,9-dione (**26**, $C_{21}H_{25}ClN_2O_2$)

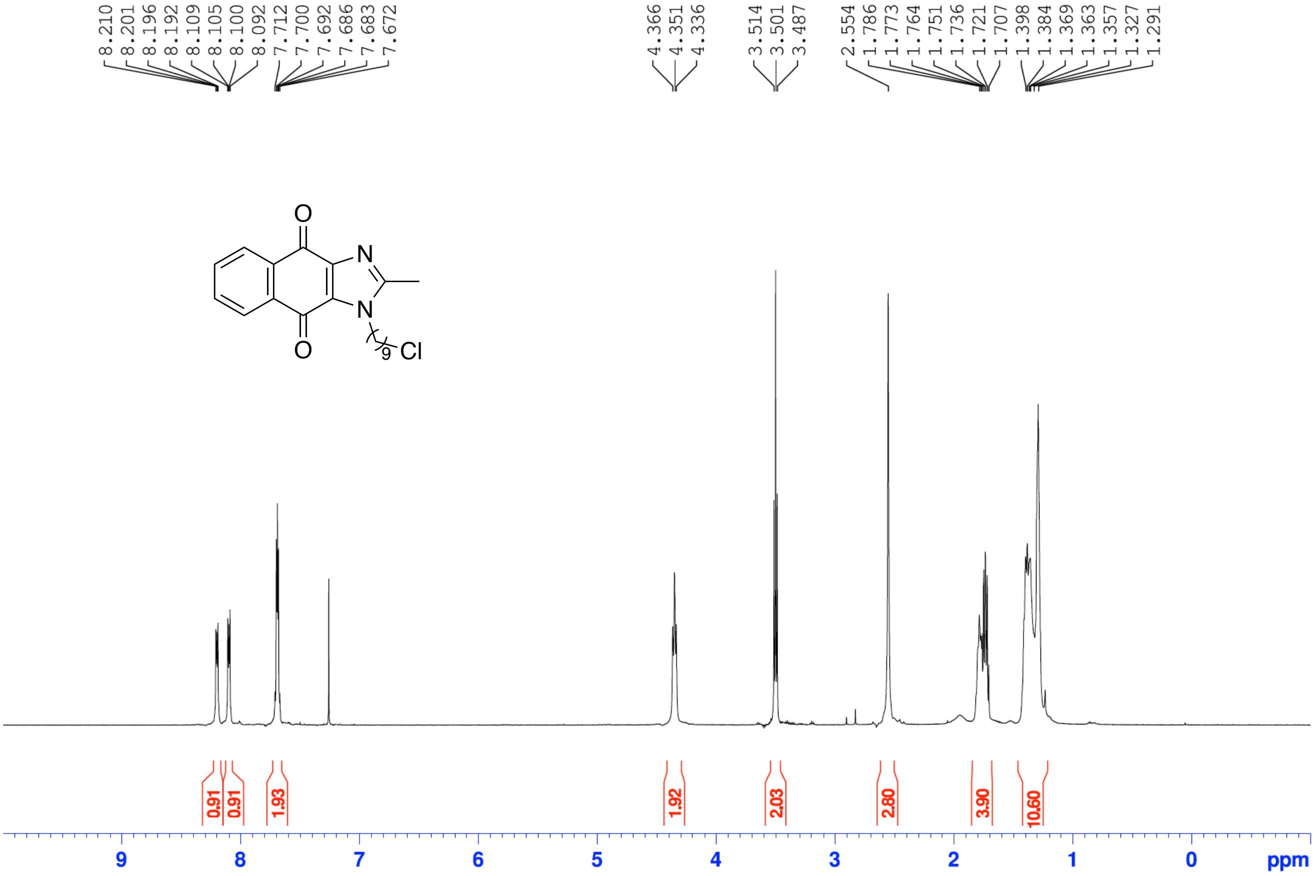

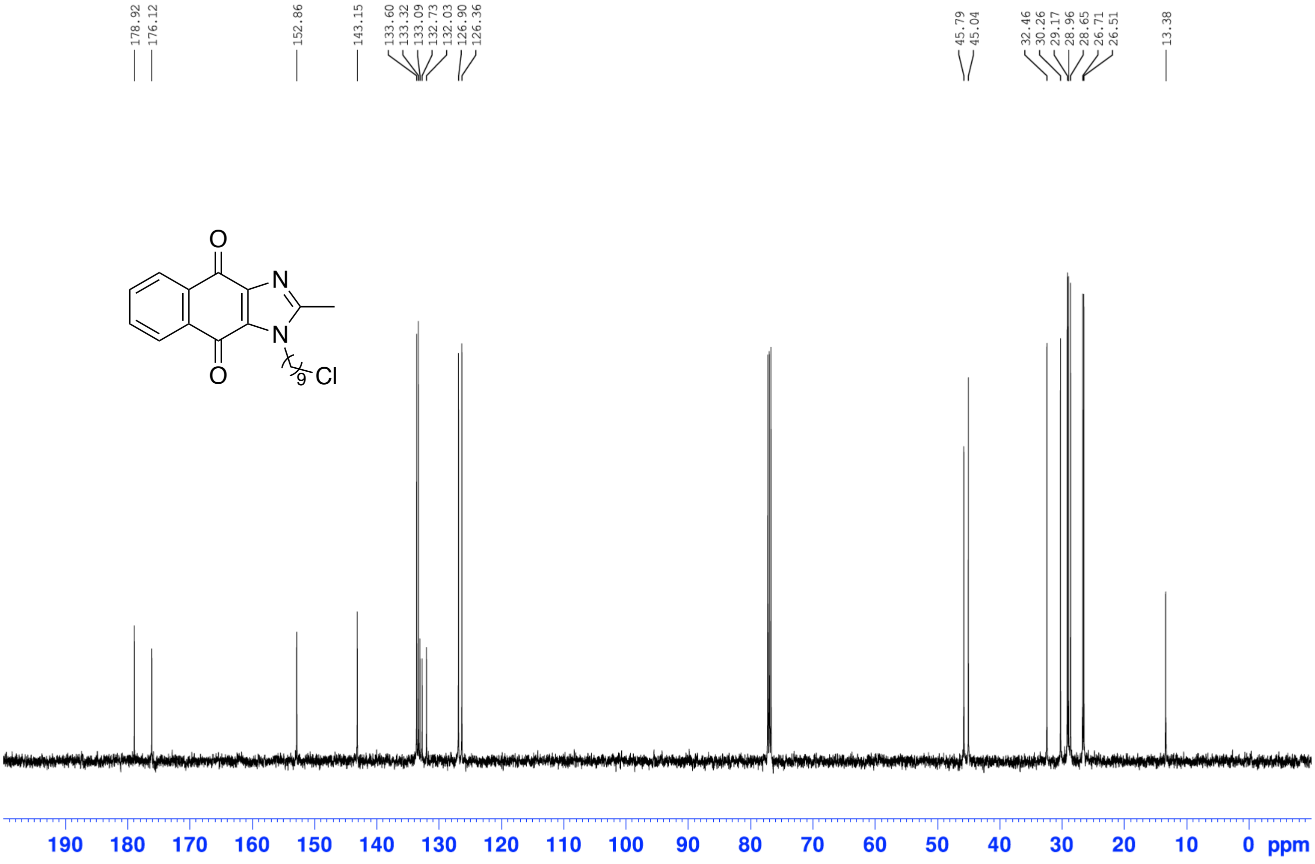

N-(11-Chloroundecyl)-2-methylnaphtho[2,3-*d*]imidazole-4,9-dione (**27**, $C_{23}H_{29}N_2O_2Cl$)

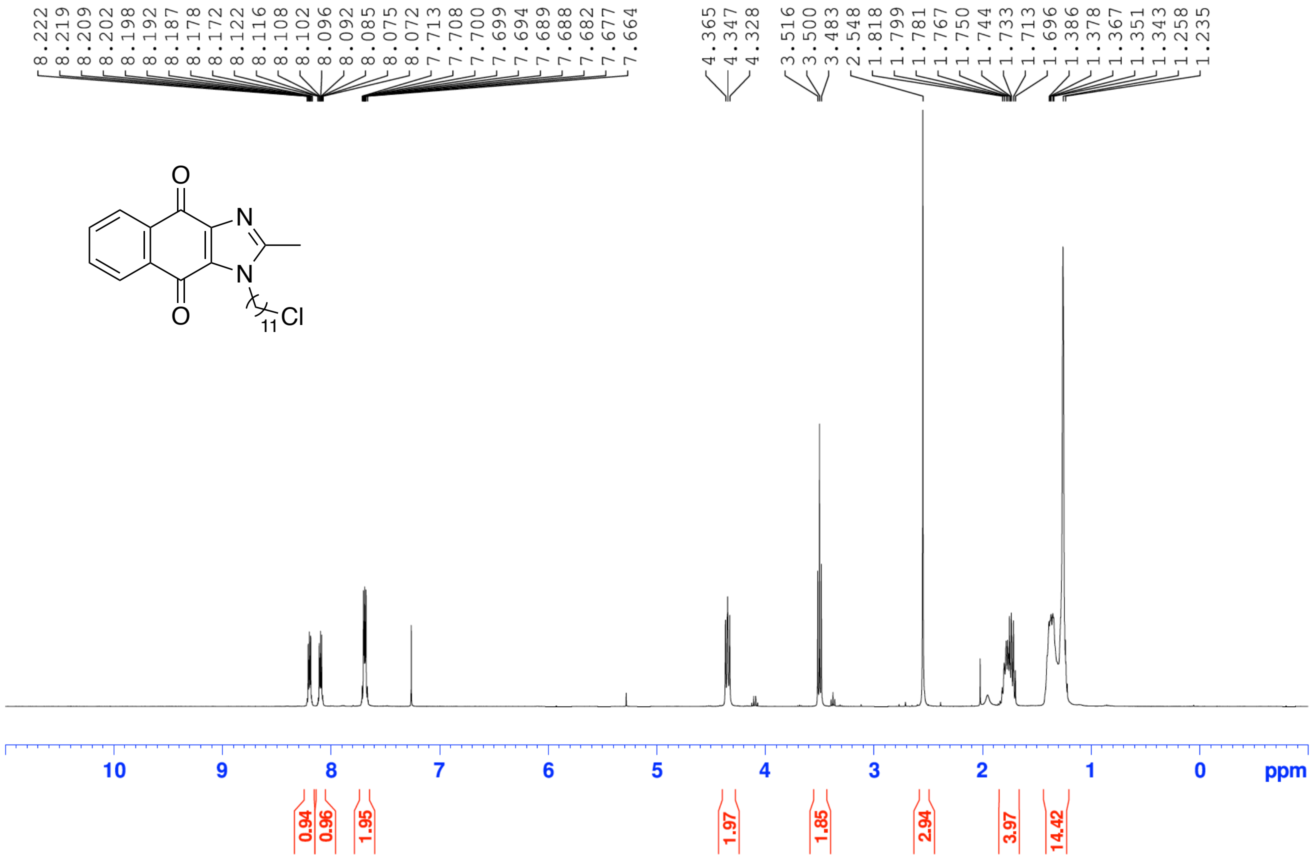

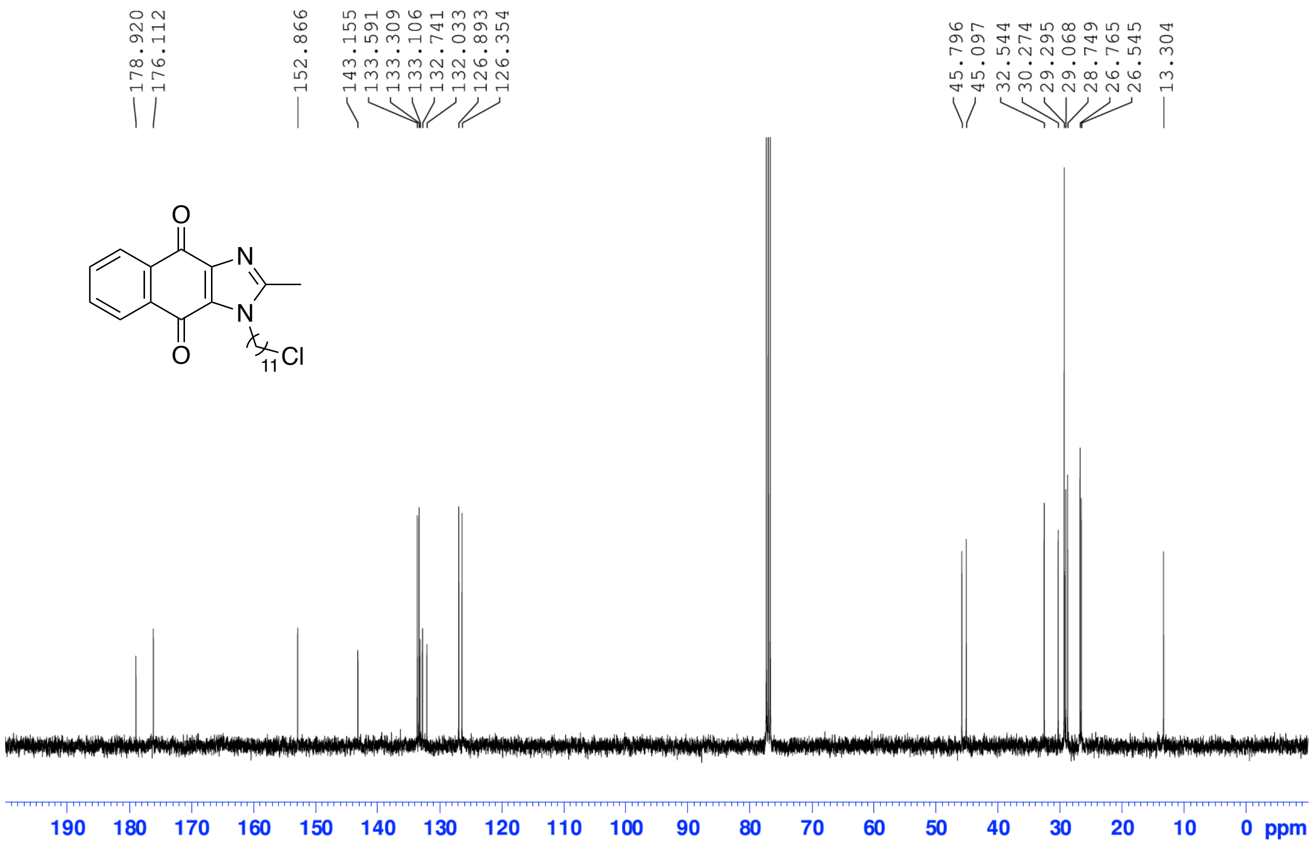

1-(Chloroheptyl)-2-methylbenzimidazole (**31**, $C_{15}H_{21}ClN_2$)

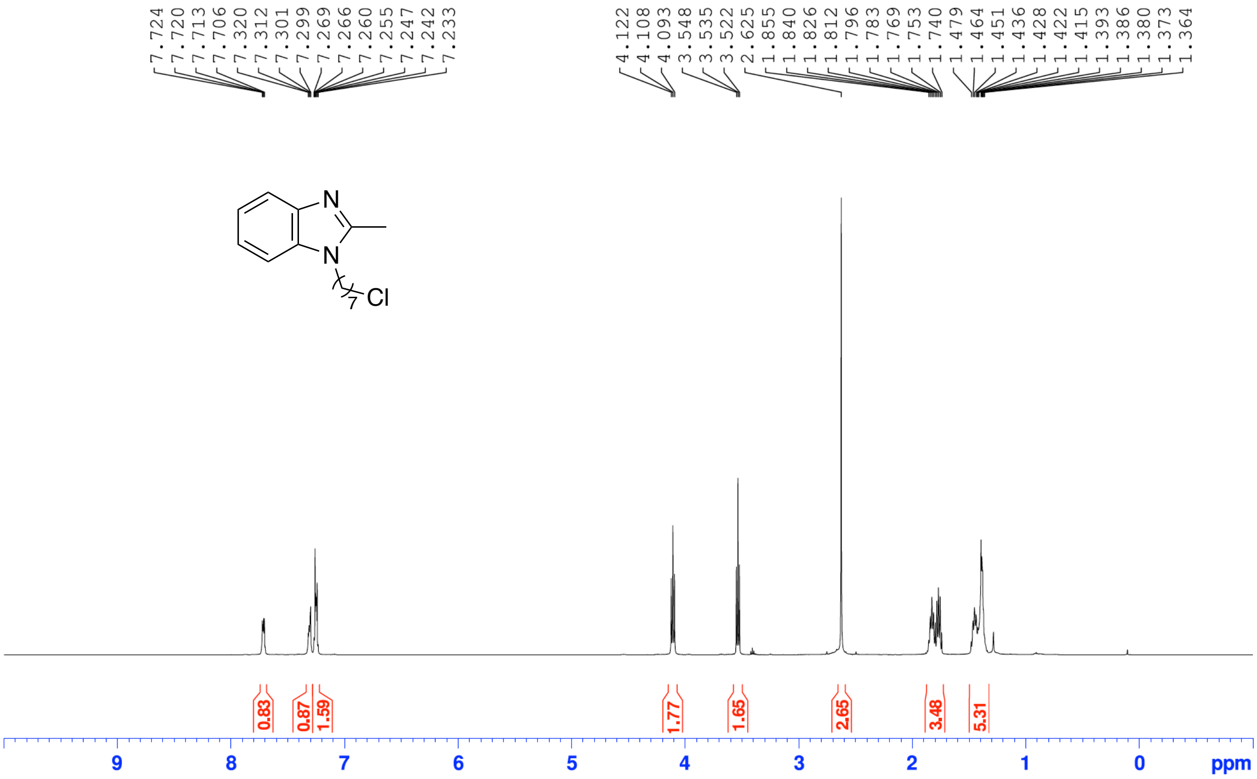

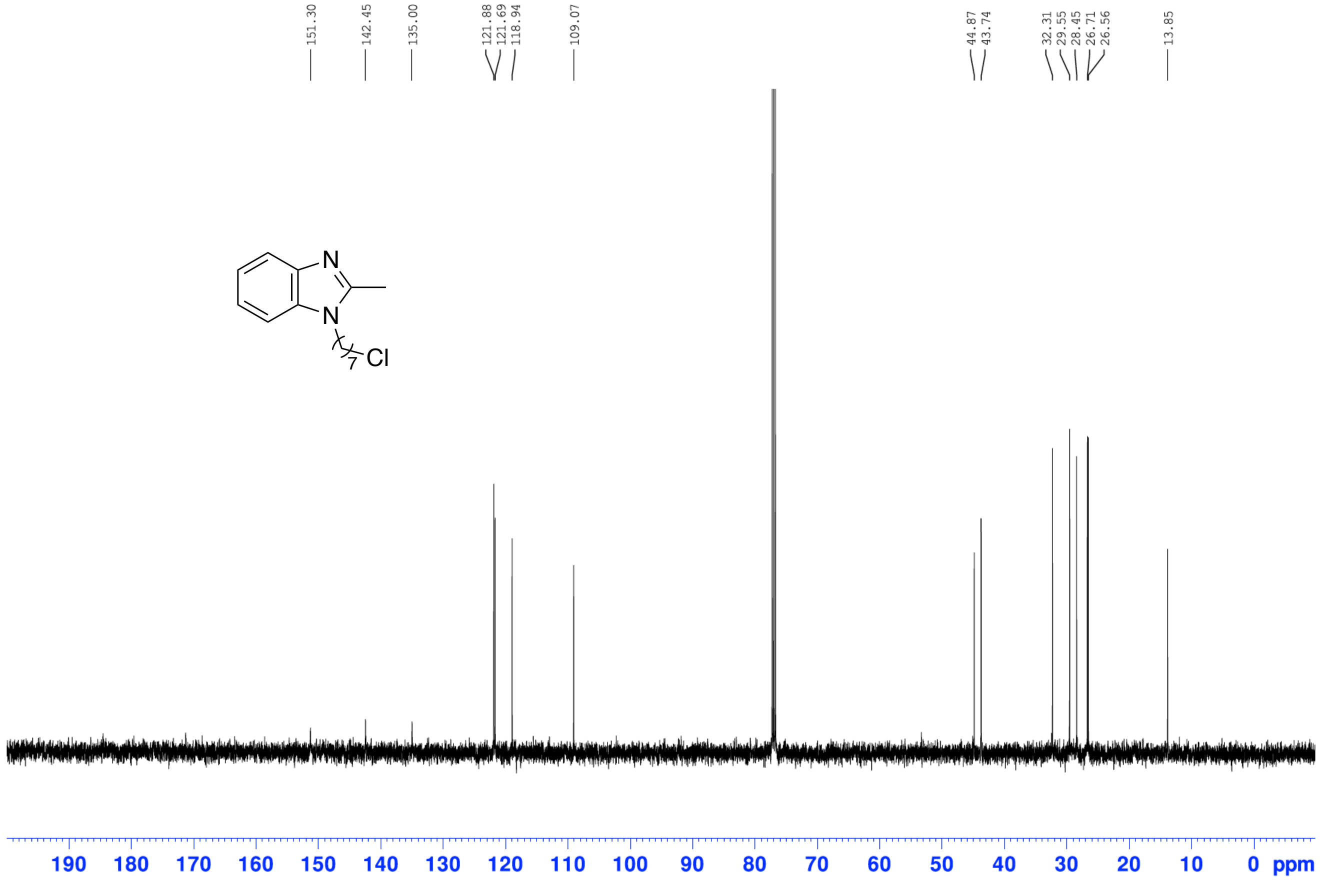

1-(Chloroundecyl)-2-methylbenzimidazole (**32**, $C_{19}H_{29}ClN_2$)

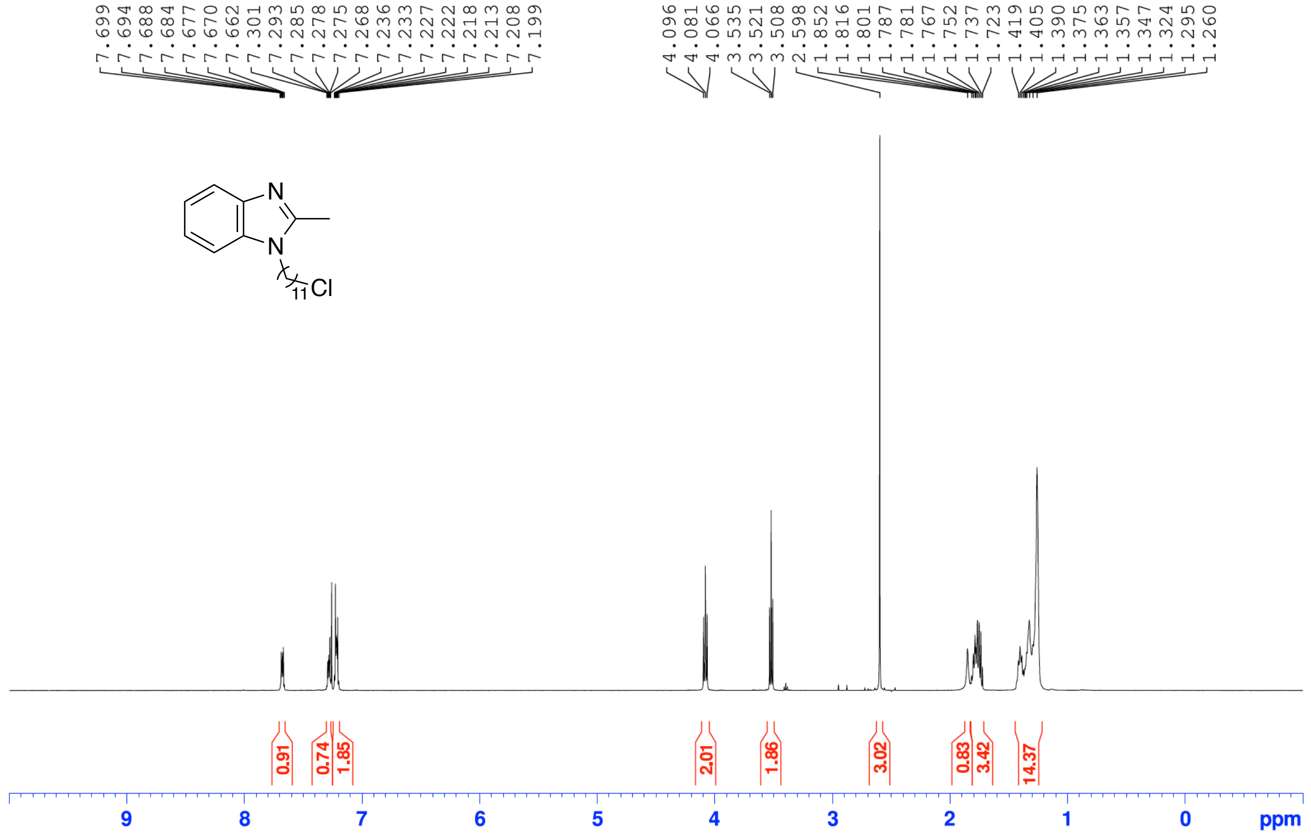

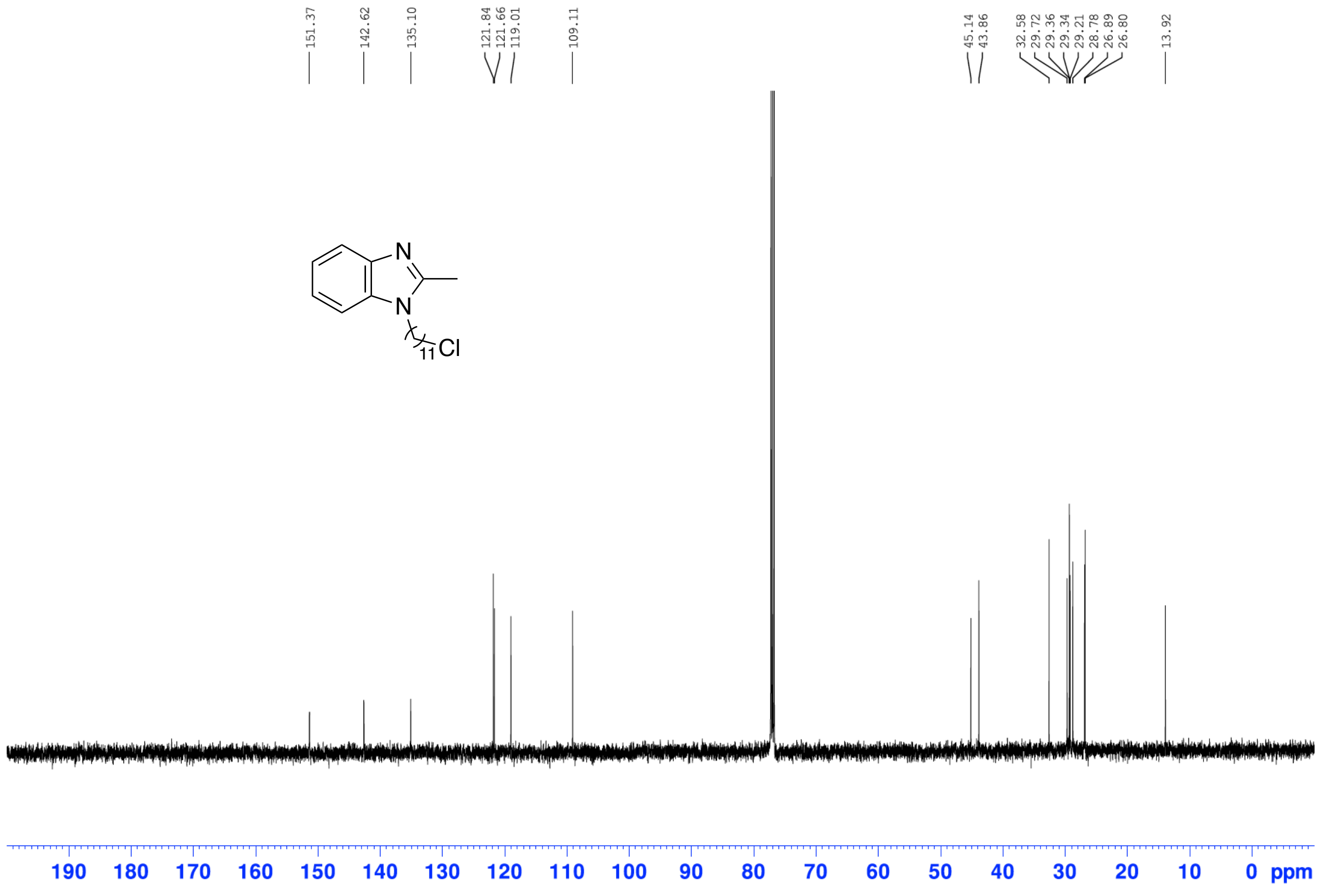

1-(7-Chloroheptyl)-4-methylbenzo[*g*]quinoline-2,5,10(1*H*)-trione (**33**, $C_{21}H_{22}ClNO_3$)

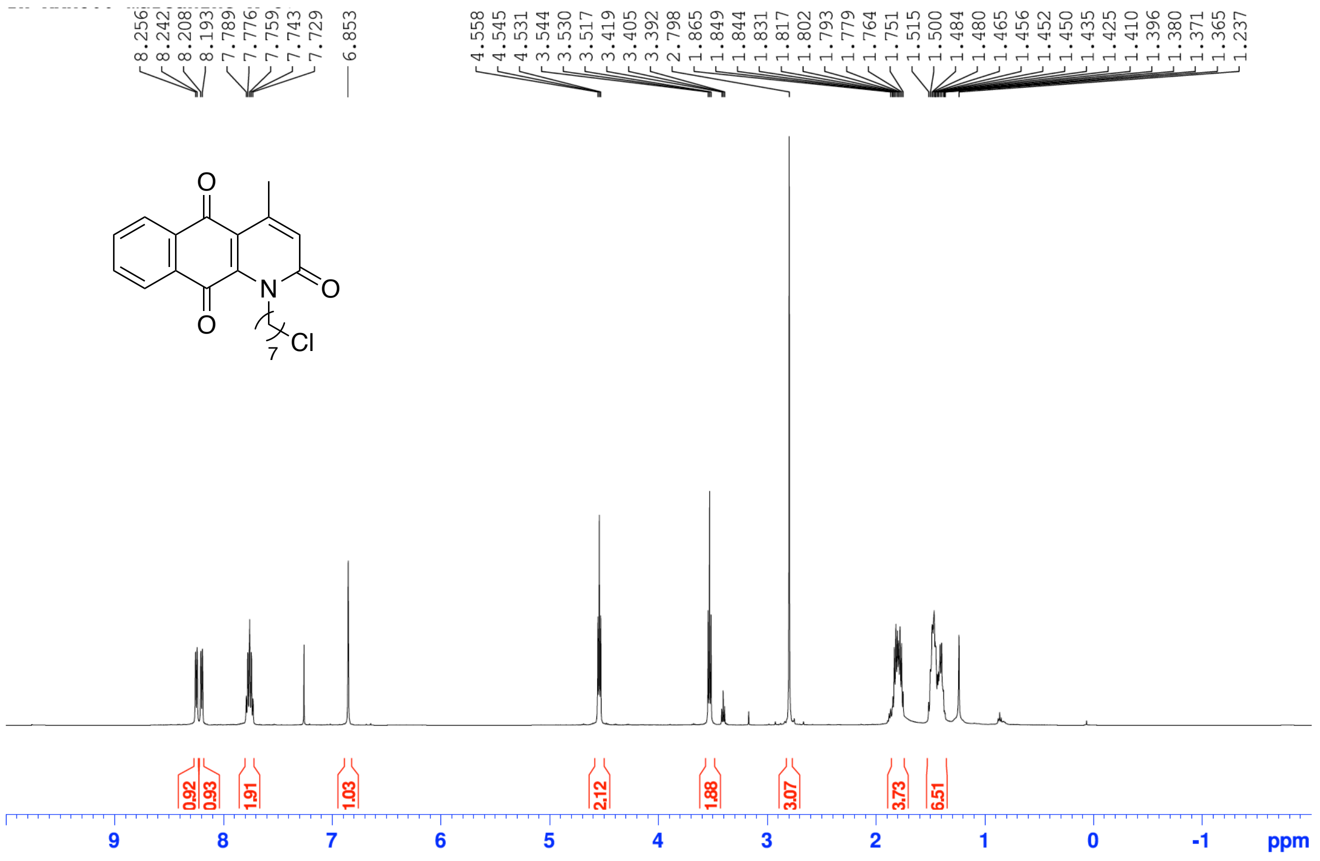

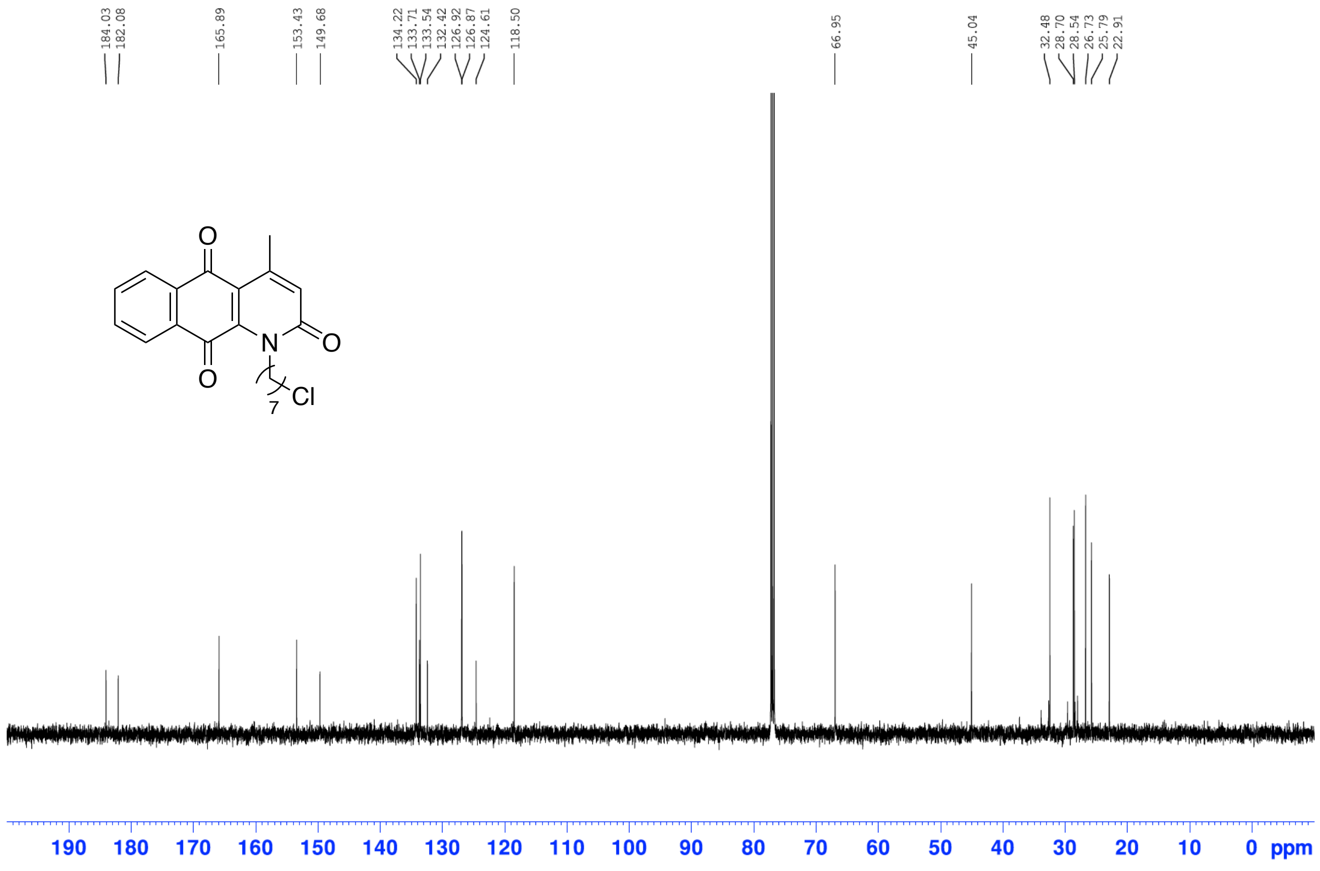

1-(11-Chloroundecyl)-4-methylbenzo[*g*]quinoline-2,5,10(1*H*)-trione (**34**, $C_{25}H_{30}ClNO_3$)

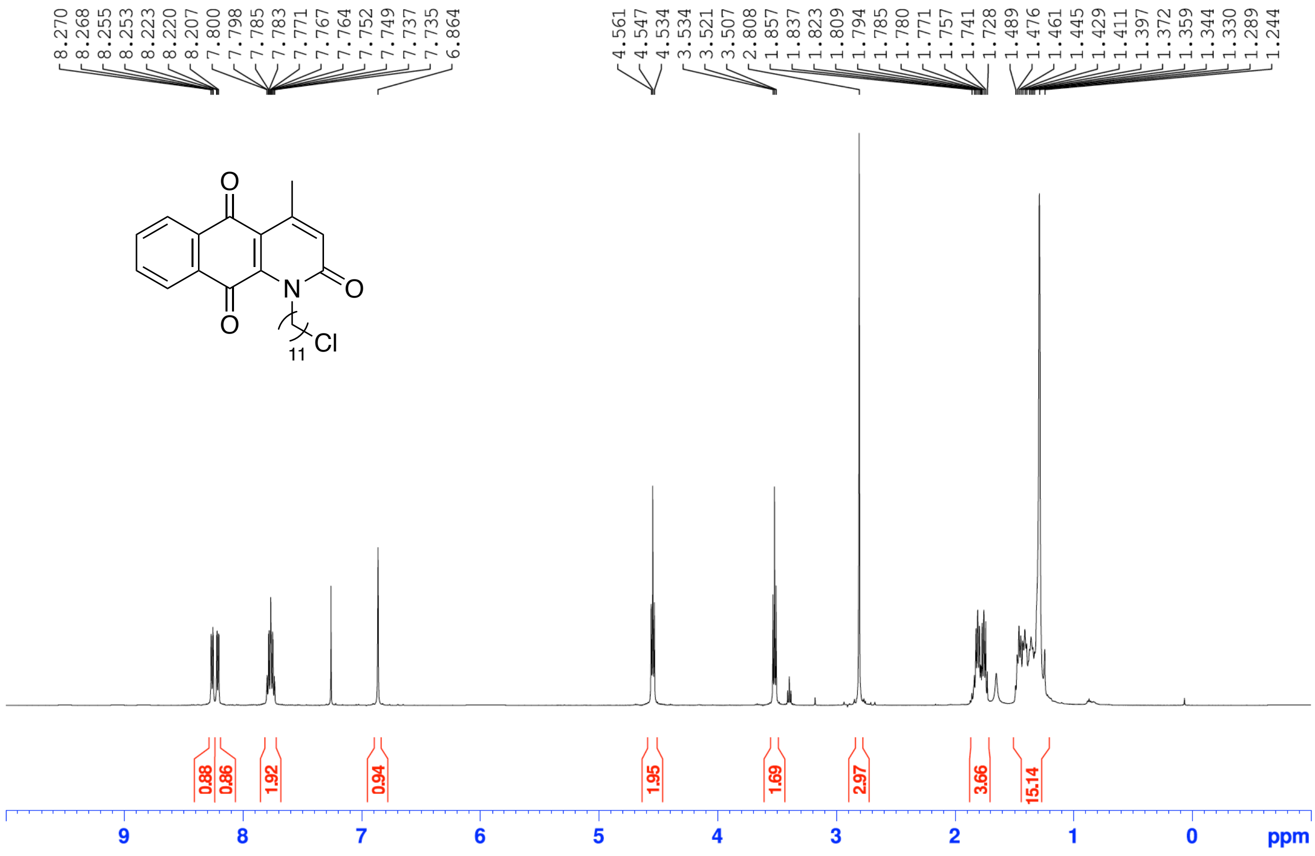

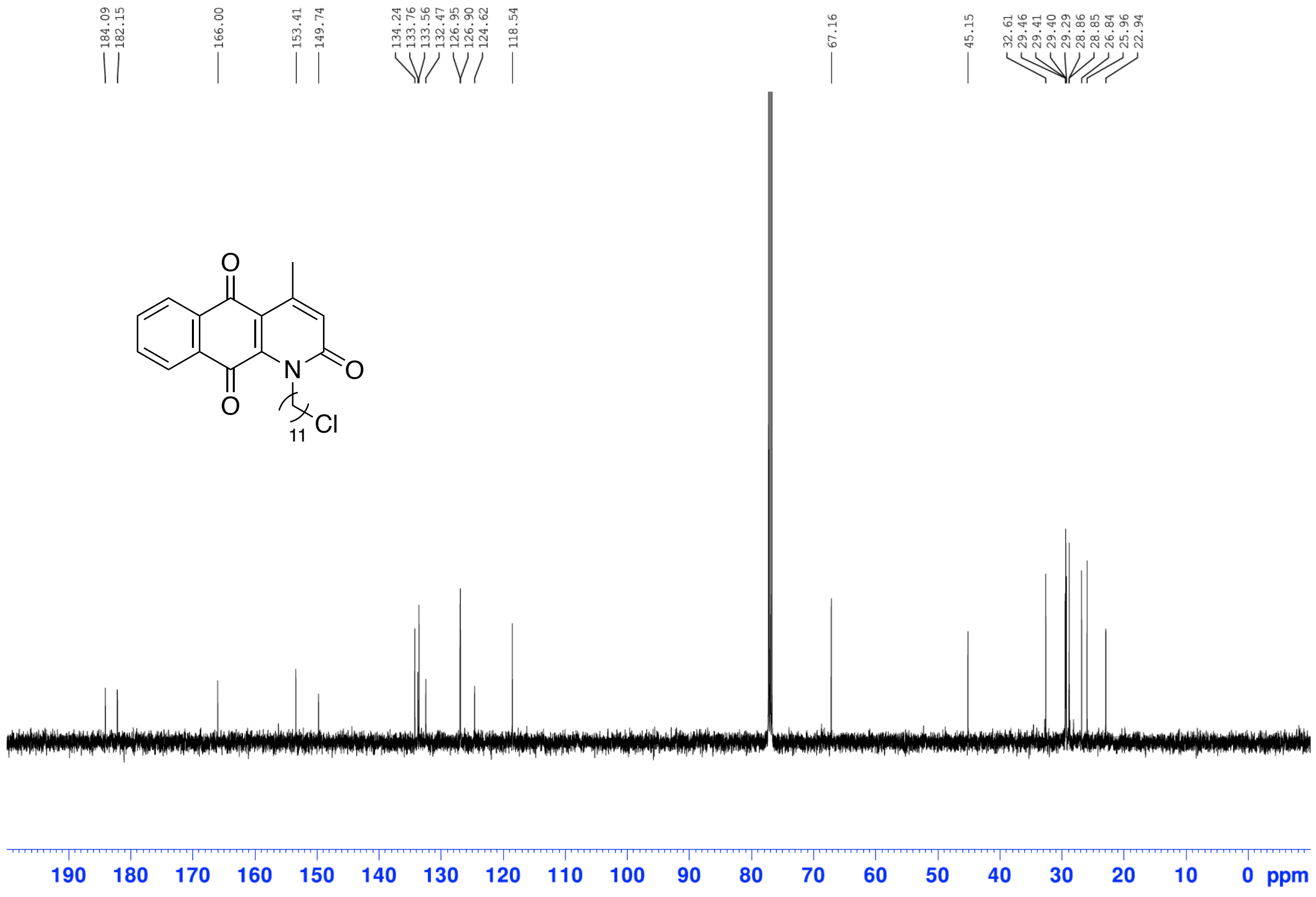

N-(11-Chloroundecyl)-2-trifluoromethylnaphtho[2,3-*d*]imidazole-4,9-dione (**36**, $C_{23}H_{26}N_2O_2ClF_3$)

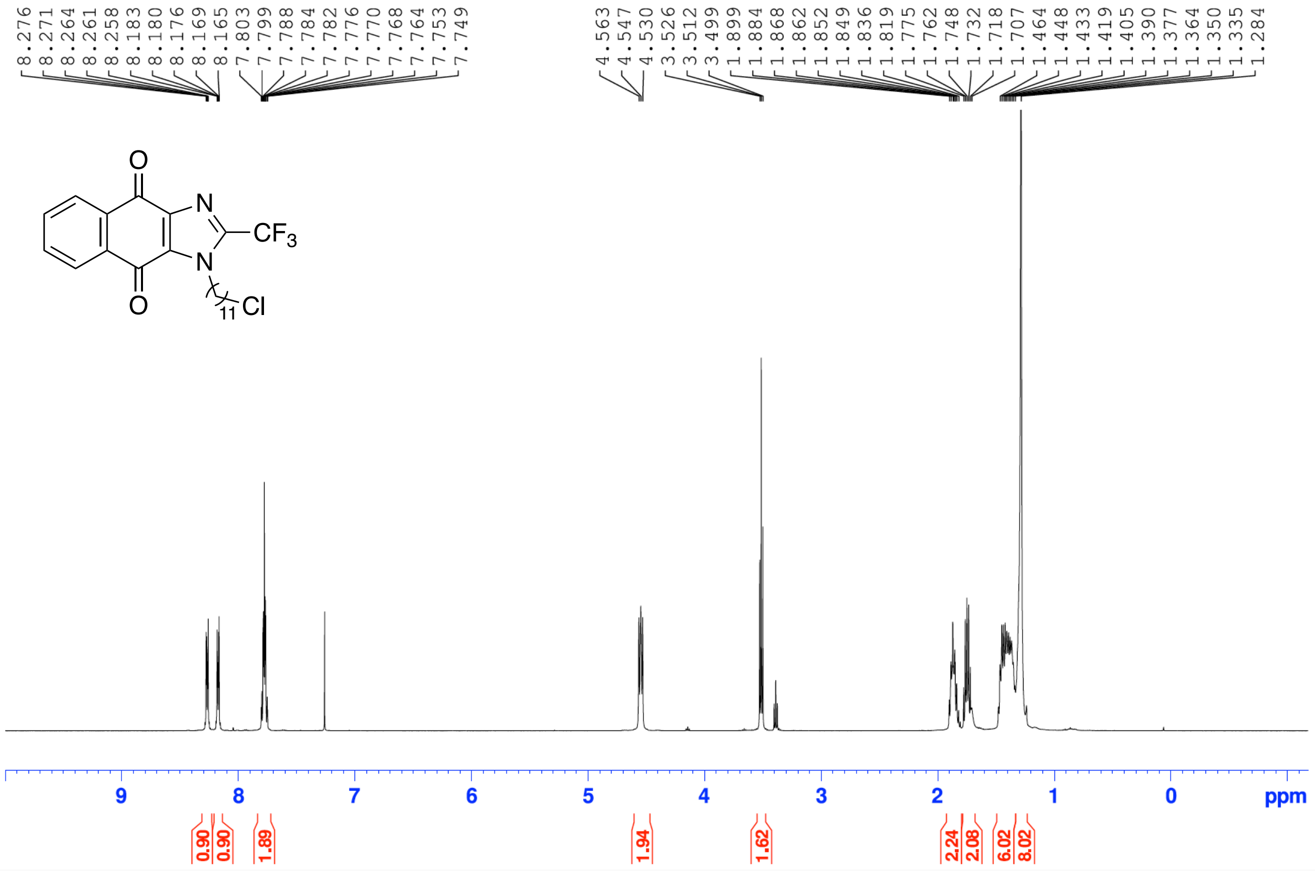

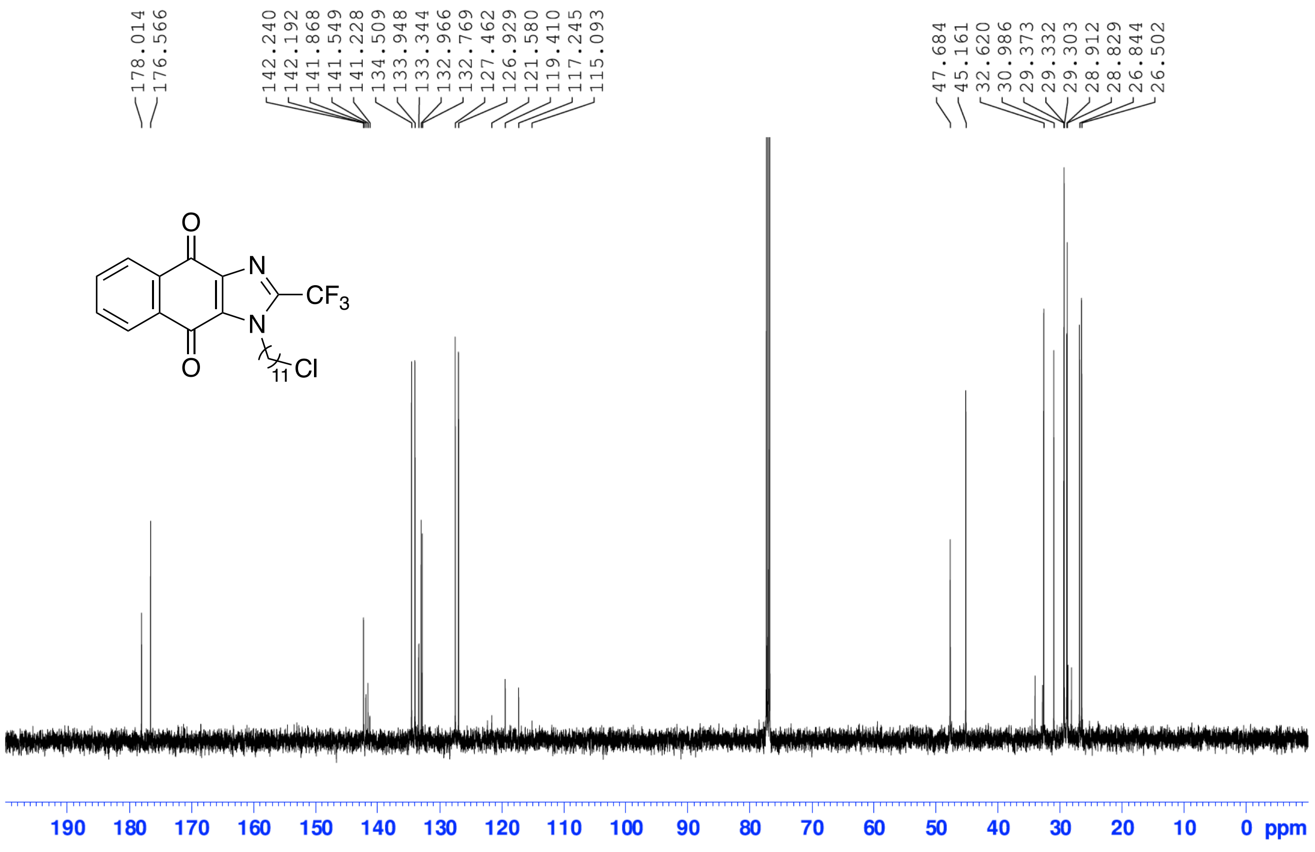

6-(11-Hydroxyundecyl)amino-7-dichloroquinoline-5,8-dione (**39**, $C_{20}H_{27}N_2O_3Cl$)

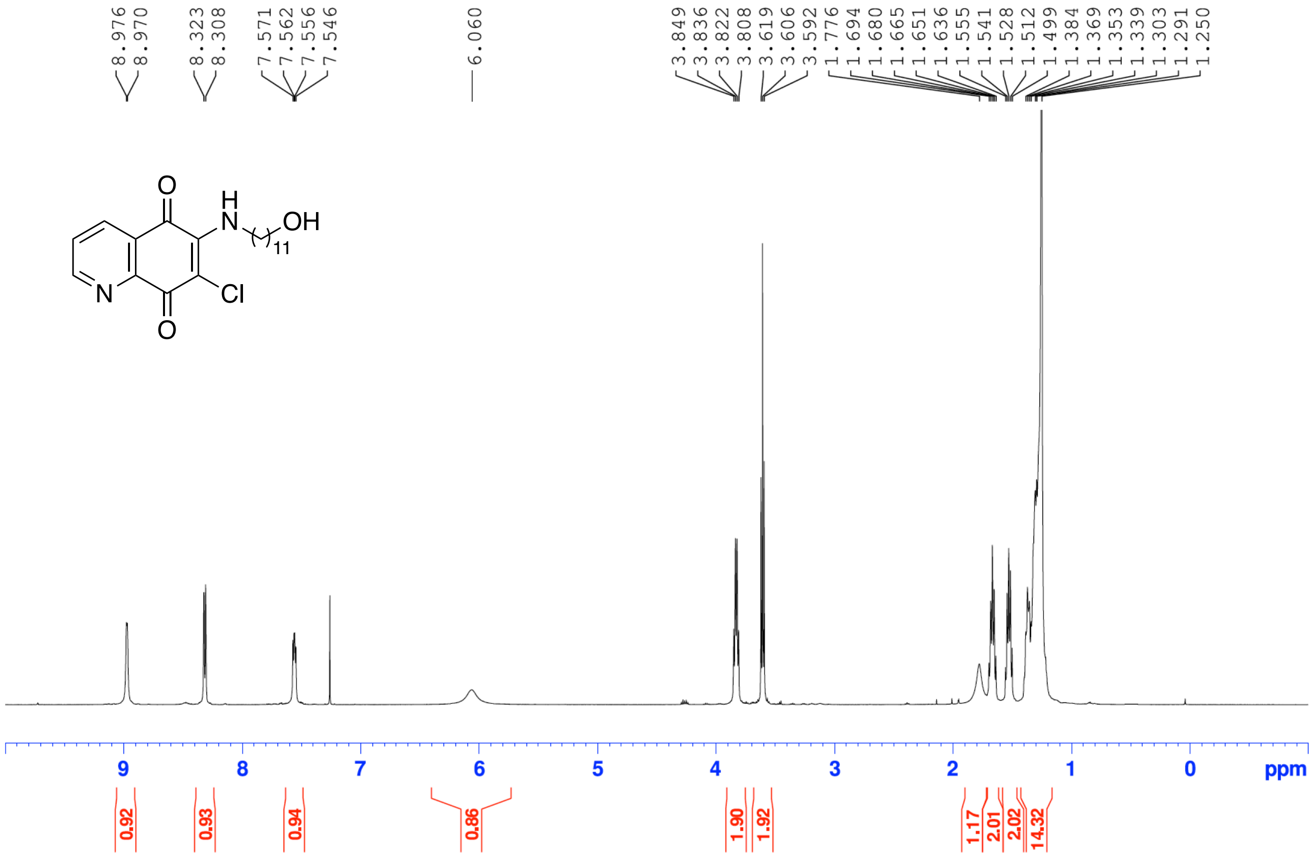

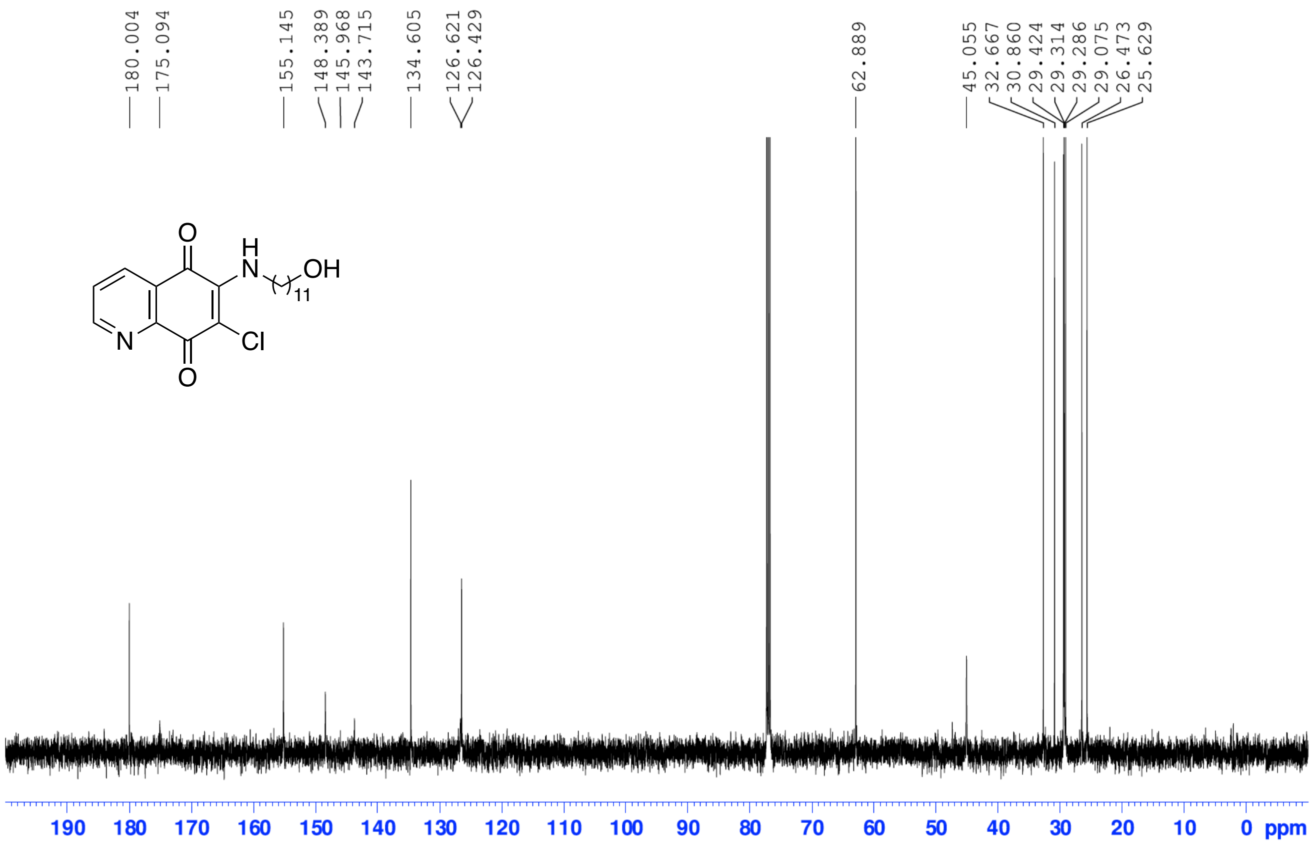

11-[*N*-(7-Chloro-5,8-dioxoquinolin-6-yl)acetamido]undecyl acetate (**40**, $C_{24}H_{31}N_2O_5$)

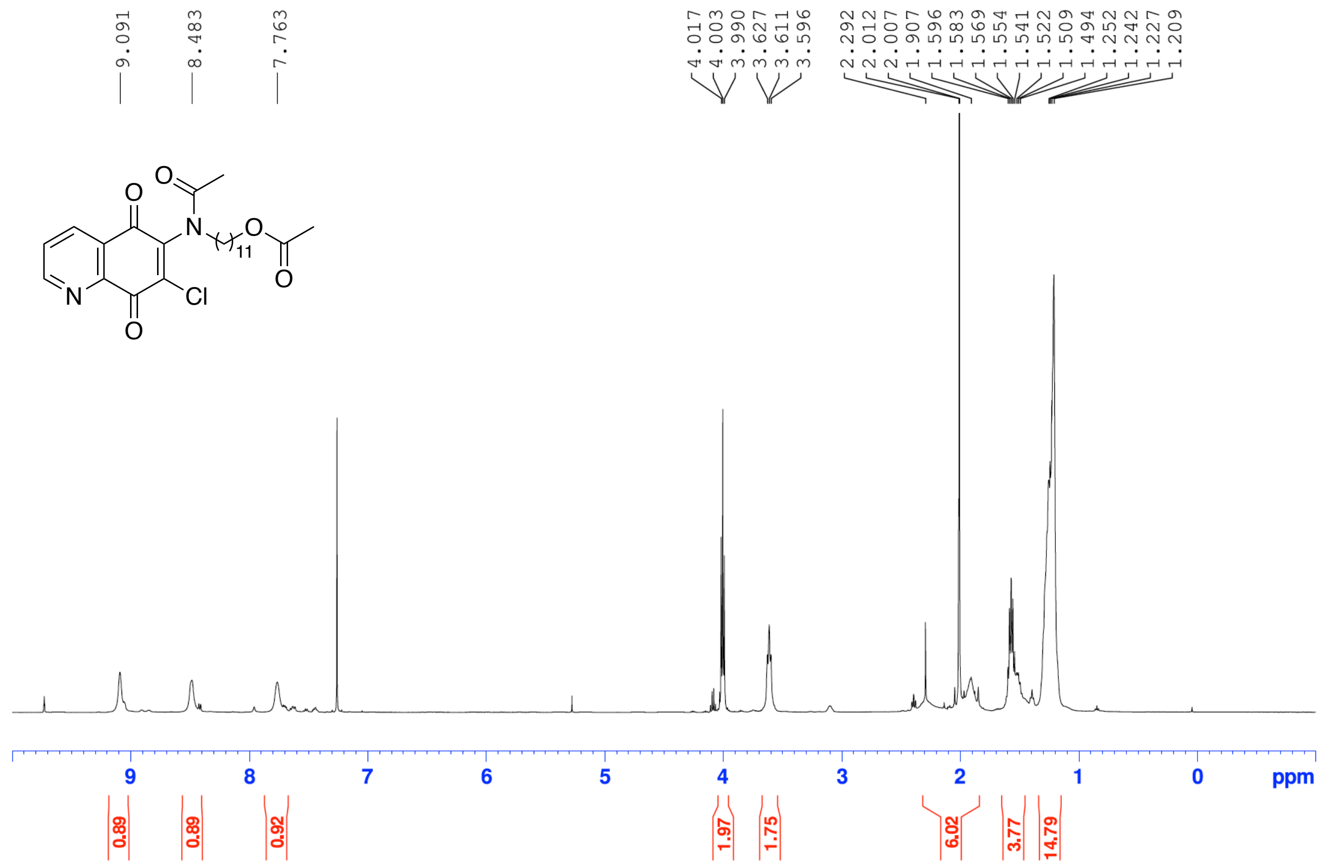

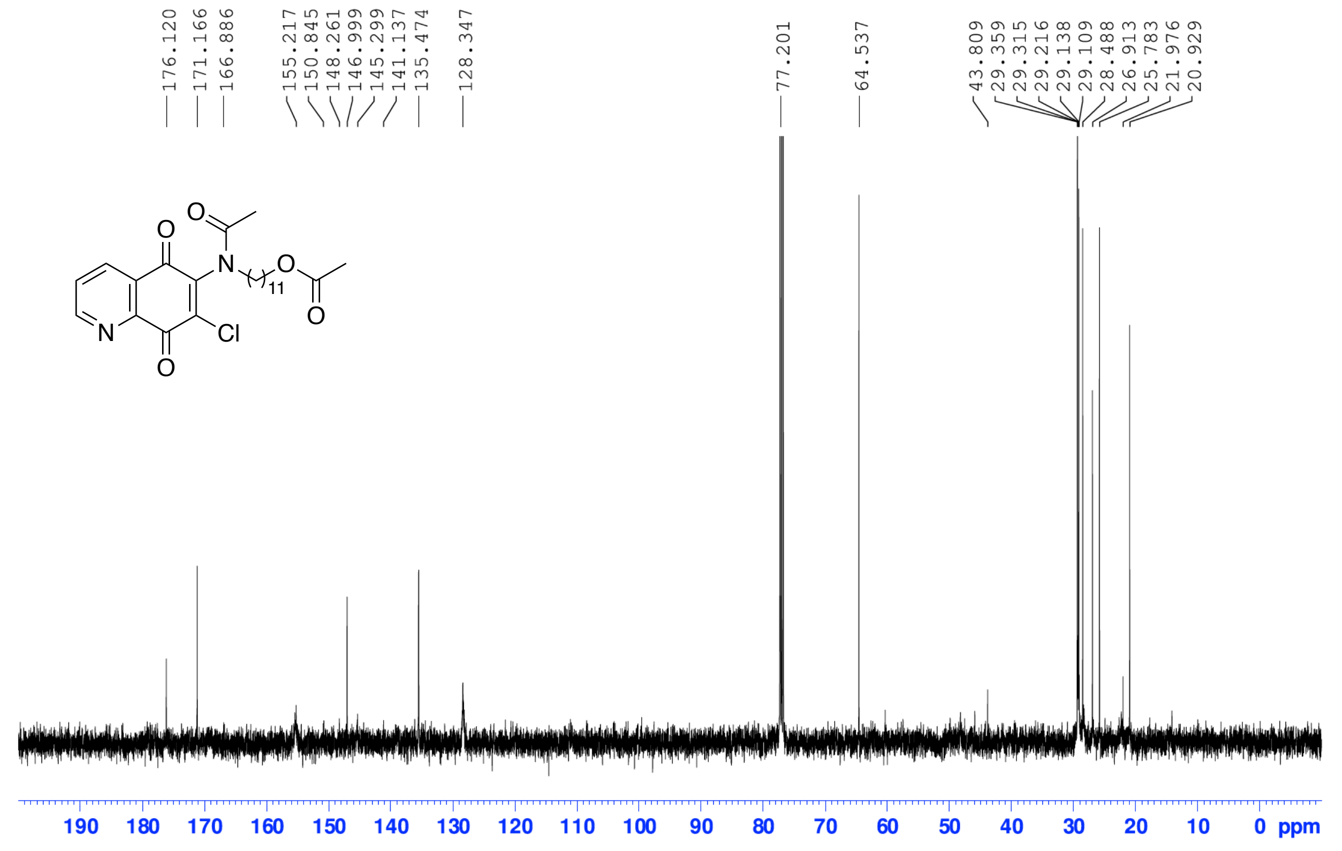

11-[*N*-(7-Amino-5,8-dioxoquinolin-6-yl)acetamido]undecyl acetate (**41**, $C_{24}H_{31}N_3O_5$)

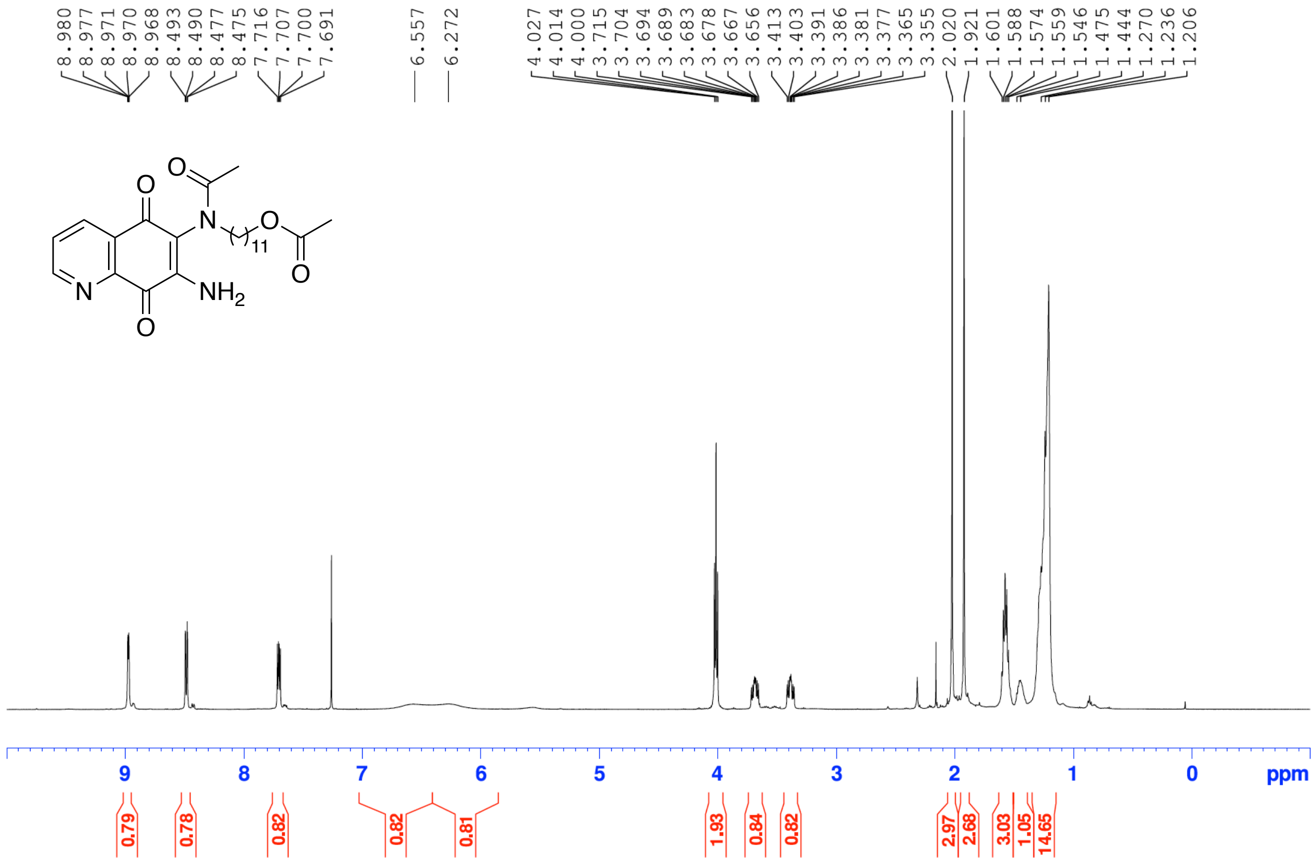

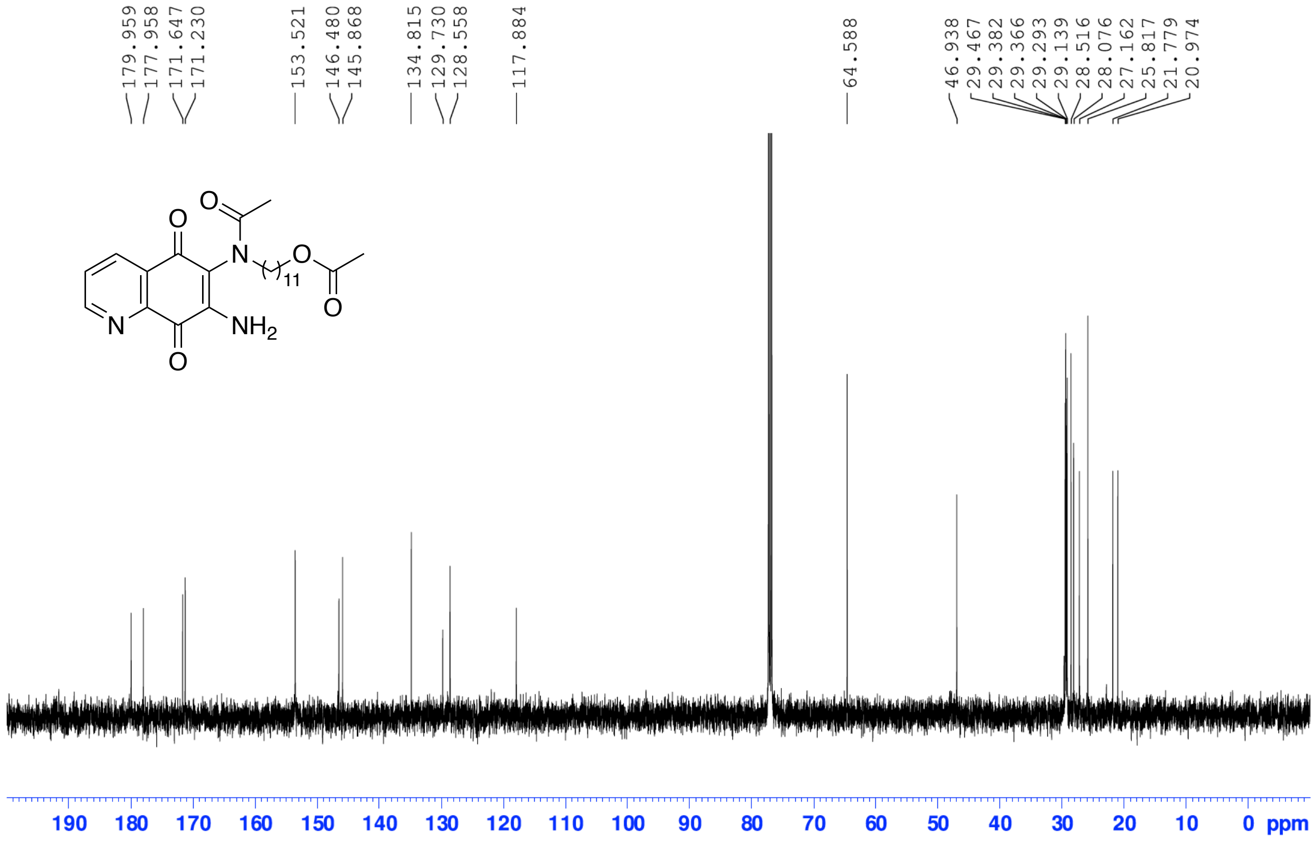

1-(11-Hydroxyundecyl)-2-methylimidazo[4,5-*g*]quinoline-4,9-dione (**42**, $C_{22}H_{29}N_3O_3$)

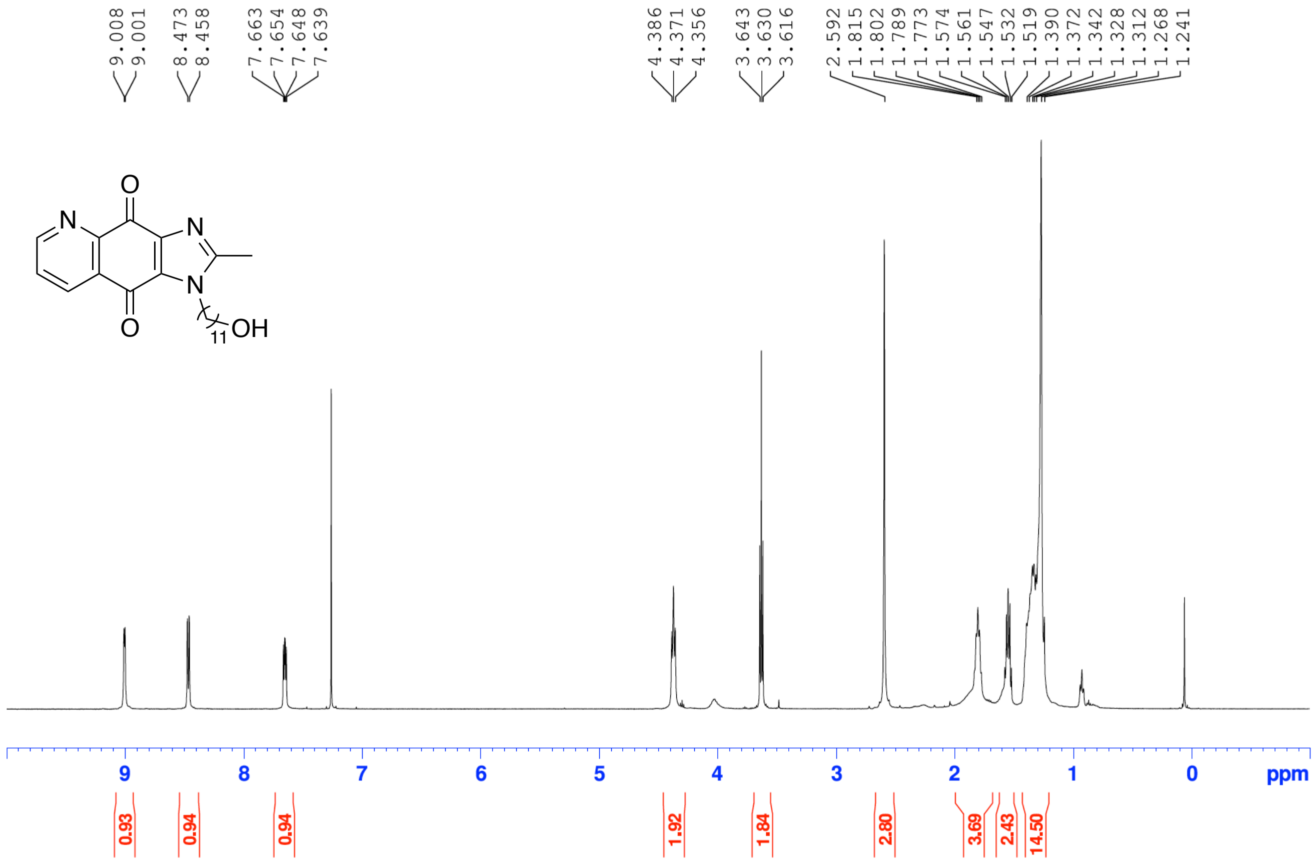

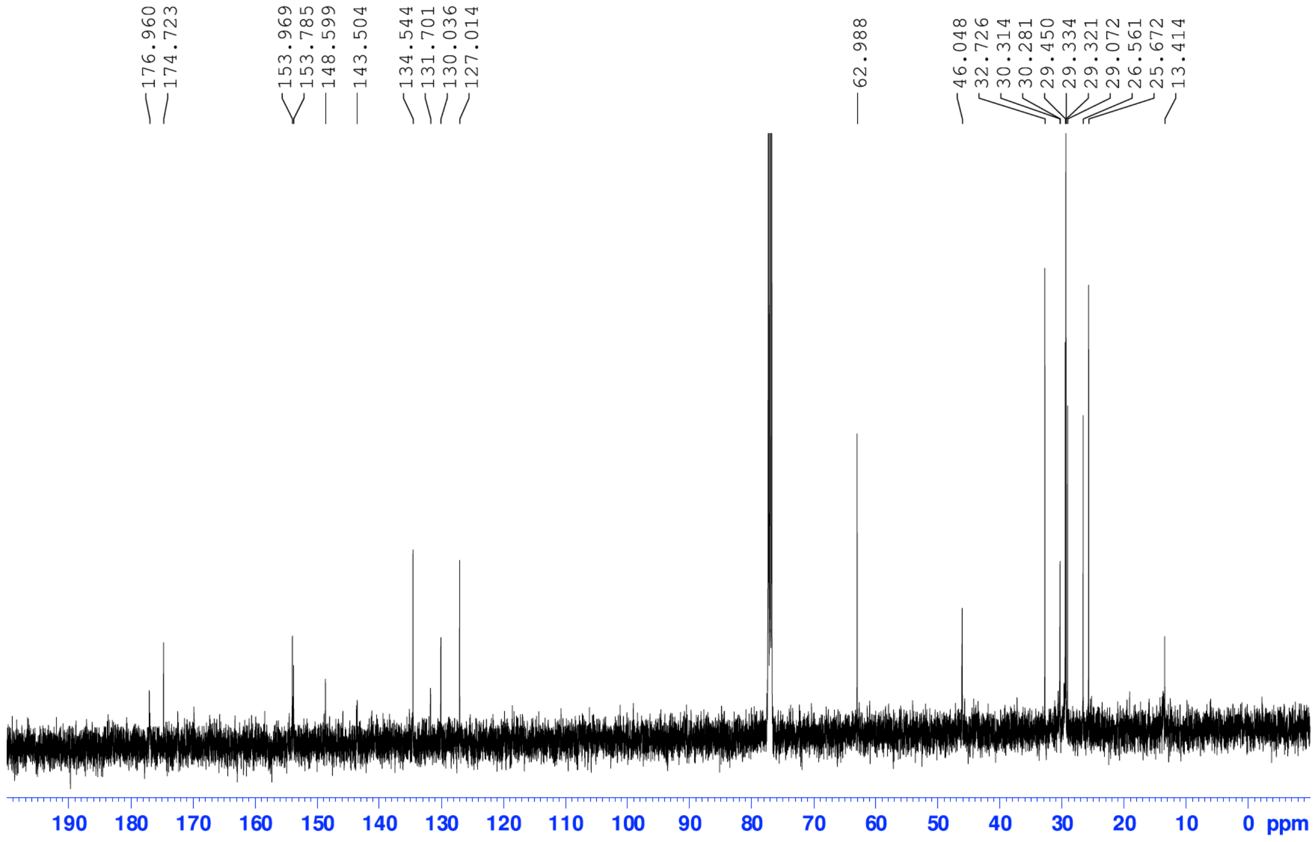

11-{2-Methyl-4,9-dioxoimidazo[4,5-*g*]quinolin-1-yl}undecyl tosylate (**43**, $C_{29}H_{34}N_3O_5S$)

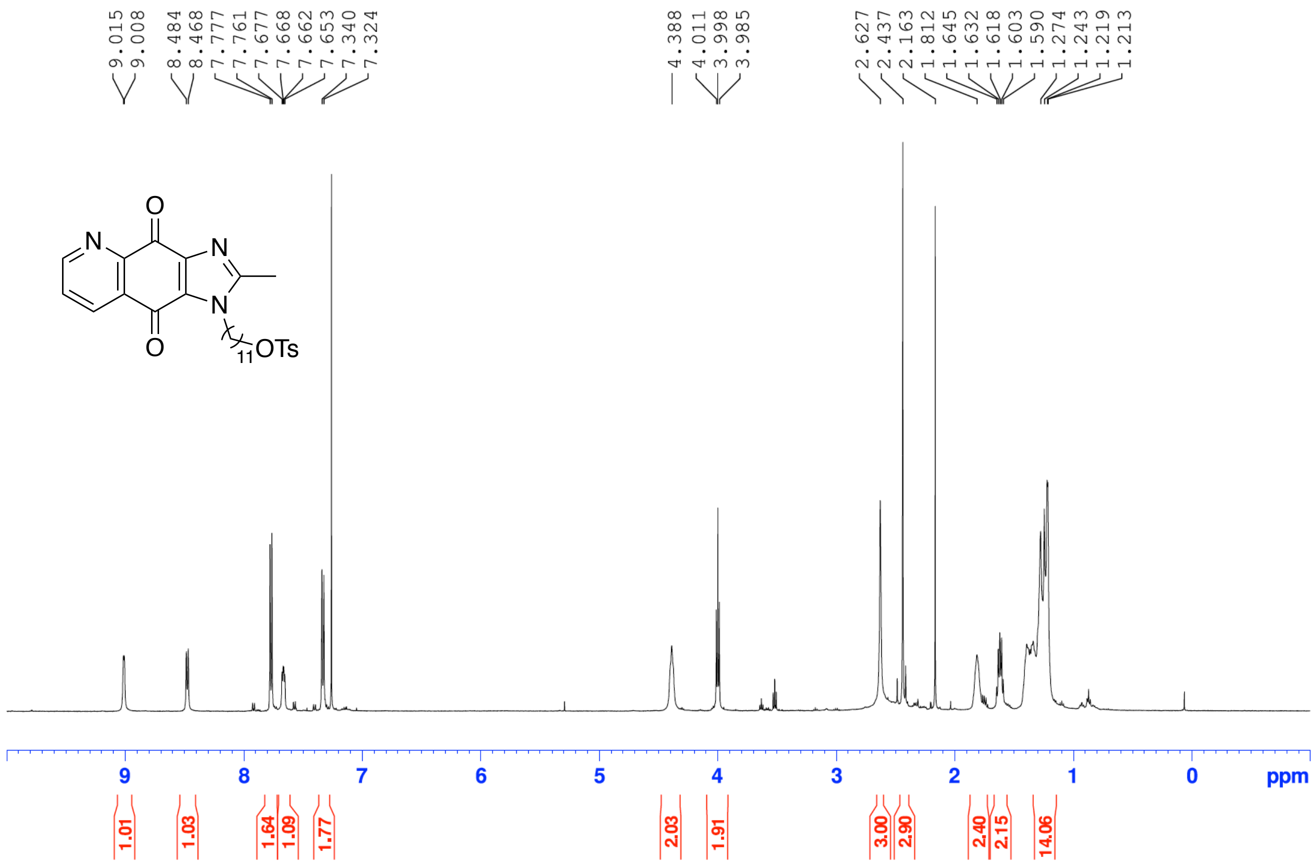

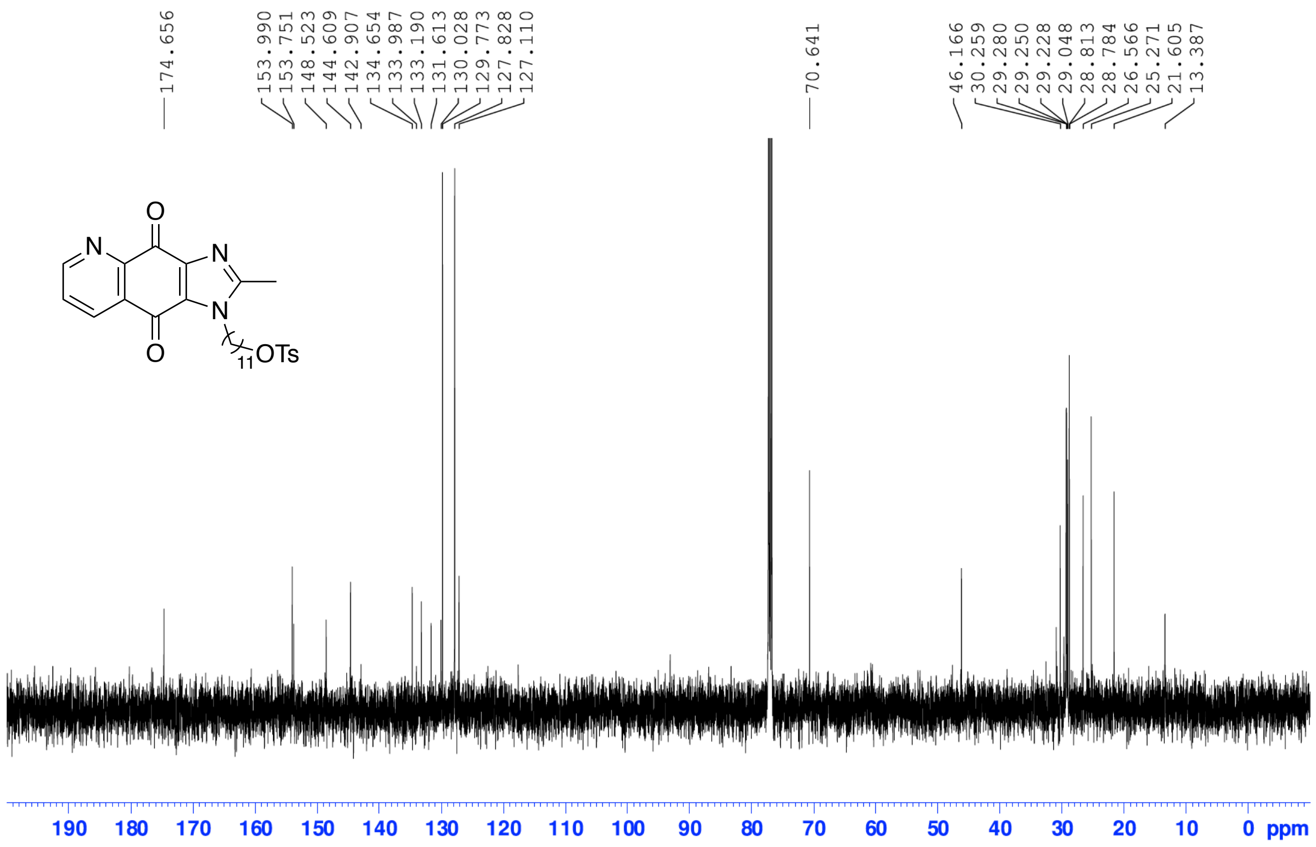

7. HRMS spectra of 6 and 7

Compound 6

Mass Spectrum SmartFormula Report

**Analysis Info**	Ion Source: **ESI**	Pority: **Positive**		Acquisition Date	1/14/2021 11:10:06 AM
Analysis Name	Z:\20210114_AcqFromChBE\SA-TPP-cg_1-E,7_01_5371.d				
Method	chemistry-1.m			Operator	NUS, Dr. Yang's Lab
Sample Name	SA-TPP-cg			Instrument / Ser#	compact
Comment	Dr Zhu Ye				8255754.20083

Meas. m/z	#	Ion Formula	m/z	err [ppm]	rdb	N-Rule
599.2826	1	C39H40N2O2P	599.2822	-0.7	21.5	ok

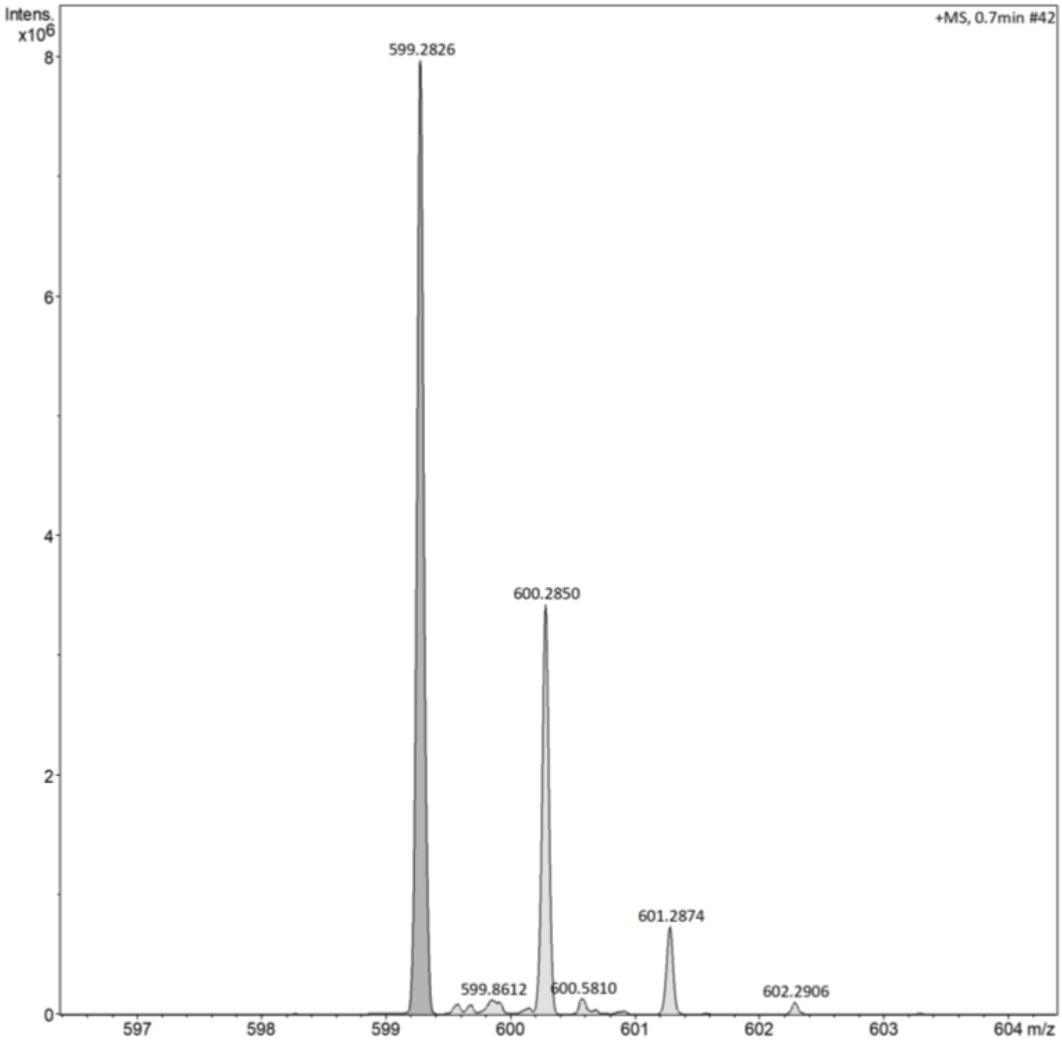

Bruker Compass DataAnalysis 4.3 printed: 1/21/2021 11:39:25 AM Page 1 of 1

Compound 7

Mass Spectrum SmartFormula Report

Analysis Info

Analysis Name	D:\Data\Chem\2020 Samples\202003\0324b\GG37.d	Acquisition Date	3/26/2020 11:23:42 AM
Method	YCH-50-500.m	Operator	default user
Sample Name	GG37	Instrument / Ser#	micrOTOF-Q II 10269
Comment	A/P Lam Yulin		

Acquisition Parameter

Source Type	ESI	Ion Polarity	Positive	Set Nebulizer	3.0 Bar
Focus	Not active	Set Capillary	4500 V	Set Dry Heater	200 °C
Scan Begin	50 m/z	Set End Plate Offset	-500 V	Set Dry Gas	6.0 l/min
Scan End	800 m/z	Set Collision Cell RF	100.0 Vpp	Set Divert Valve	Waste

Meas. m/z	#	Formula	m/z	err [ppm]	rdb	e⁻ Conf	N-Rule
627.3148	1	C 41 H 44 N 2 O 2 P	627.3135	-2.1	21.5	even	ok

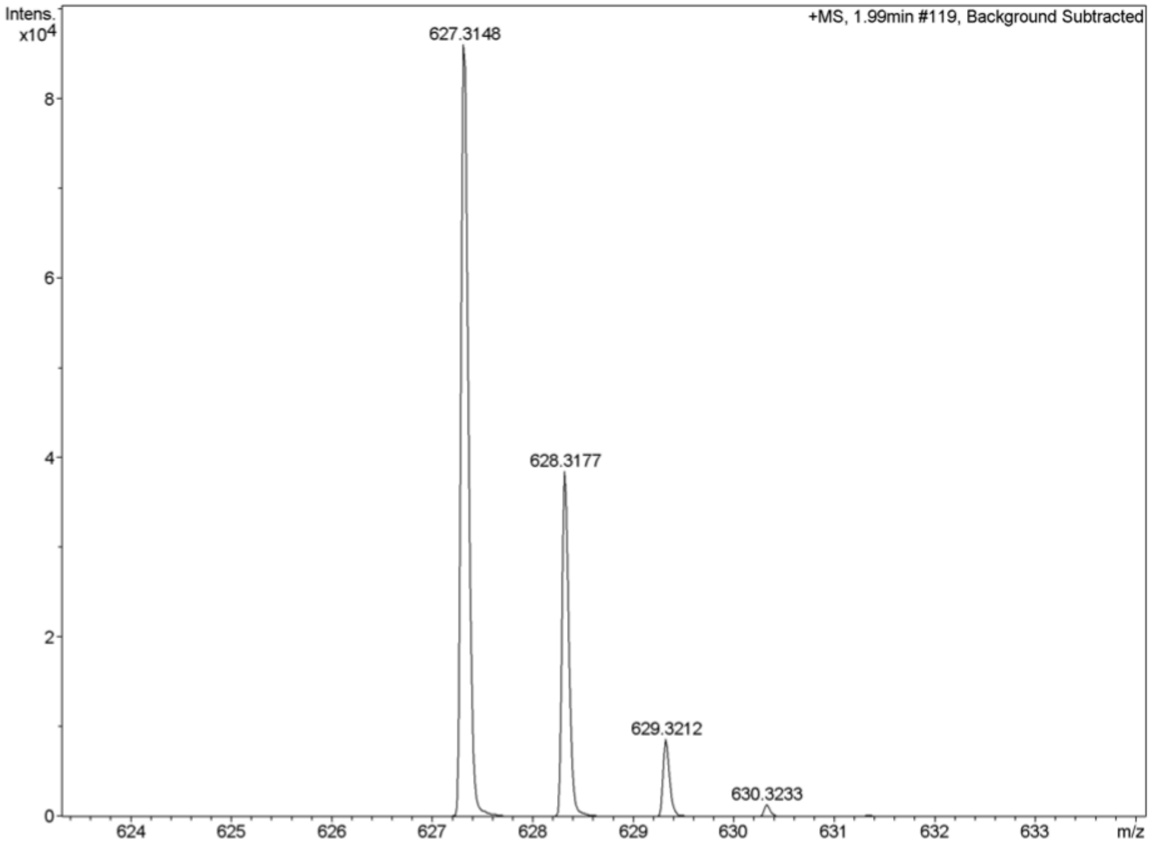

Bruker Compass DataAnalysis 4.0 printed: 3/26/2020 11:26:55 AM Page 1 of 1

8. HPLC chromatograms of 6 and 7

Compound 6

SHIMADZU LabSolutions

Analysis Report

<Sample Information>

Sample Name	: SA-C9-TPP recolumn		
Sample ID	: SA-C9-TPP recolumn		
Data Filename	: SA-C9-TPP recolumn.lcd		
Method Filename	: Kevin 85 ACN.lcm		
Batch Filename	: Kevin HPLC ACN 22.12.20.lcb		
Vial #	: 1-1	Sample Type	: Unknown
Injection Volume	: 10 uL		
Date Acquired	: 22/12/2020 11:58:37 AM	Acquired by	: System Administrator
Date Processed	: 22/12/2020 1:57:20 PM	Processed by	: System Administrator

<Chromatogram>

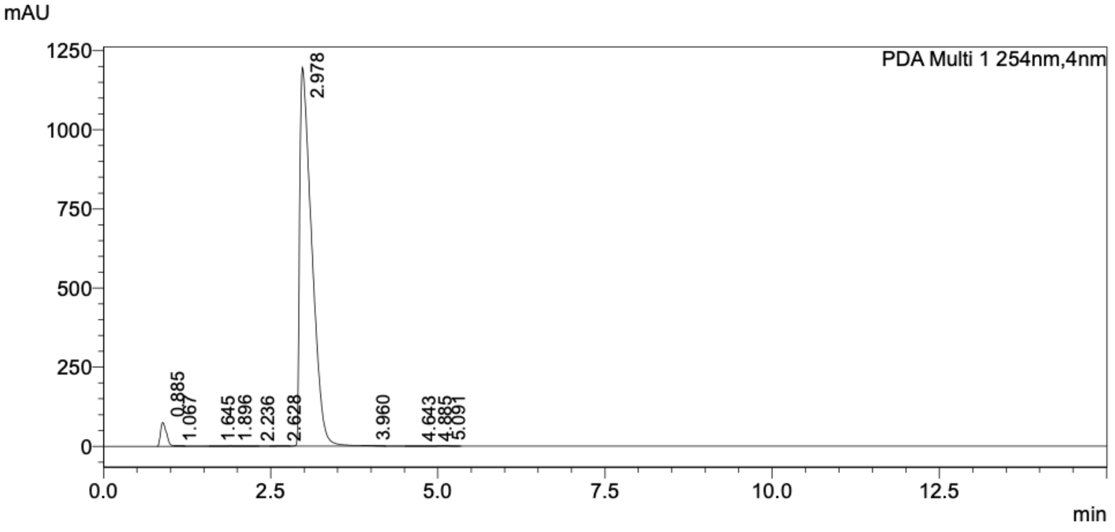

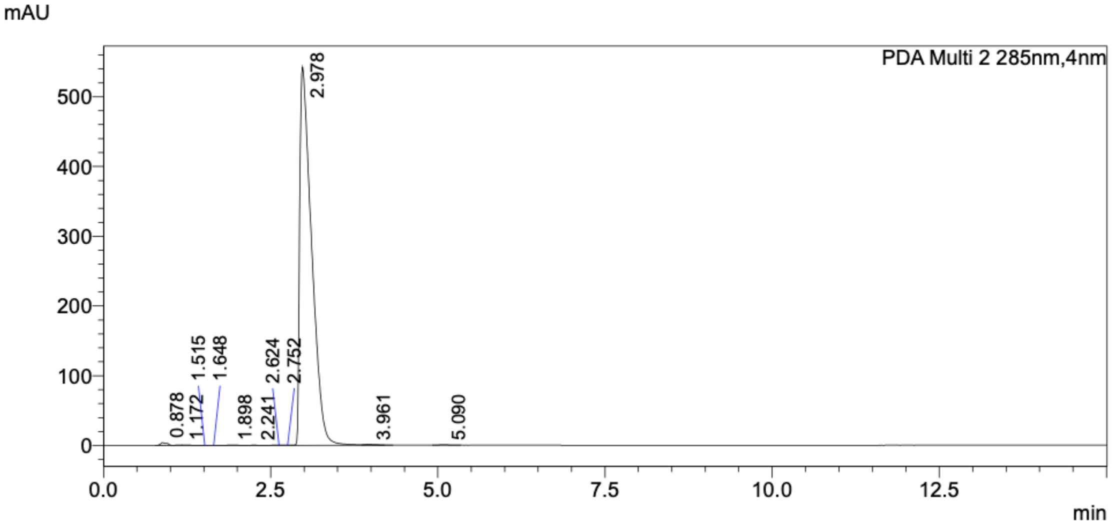

Analysis Report

<Sample Information>

Sample Name	: SA-C9-TPP recolumn MeOH		
Sample ID	: SA-C9-TPP recolumn MeOH		
Data Filename	: SA-C9-TPP recolumn MeOH.lcd		
Method Filename	: Kevin 95 MeOH.lcm		
Batch Filename	: Kevin HPLC MeOH 22.12.20.lcb		
Vial #	: 1-1	Sample Type	: Unknown
Injection Volume	: 10 uL		
Date Acquired	: 22/12/2020 2:15:19 PM	Acquired by	: System Administrator
Date Processed	: 22/12/2020 3:05:59 PM	Processed by	: System Administrator

<Chromatogram>

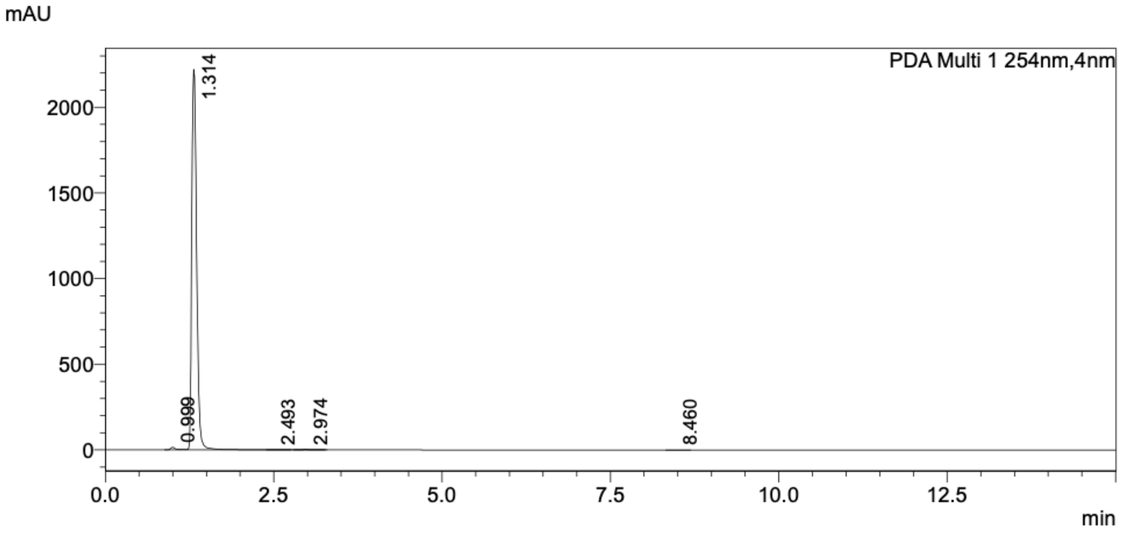

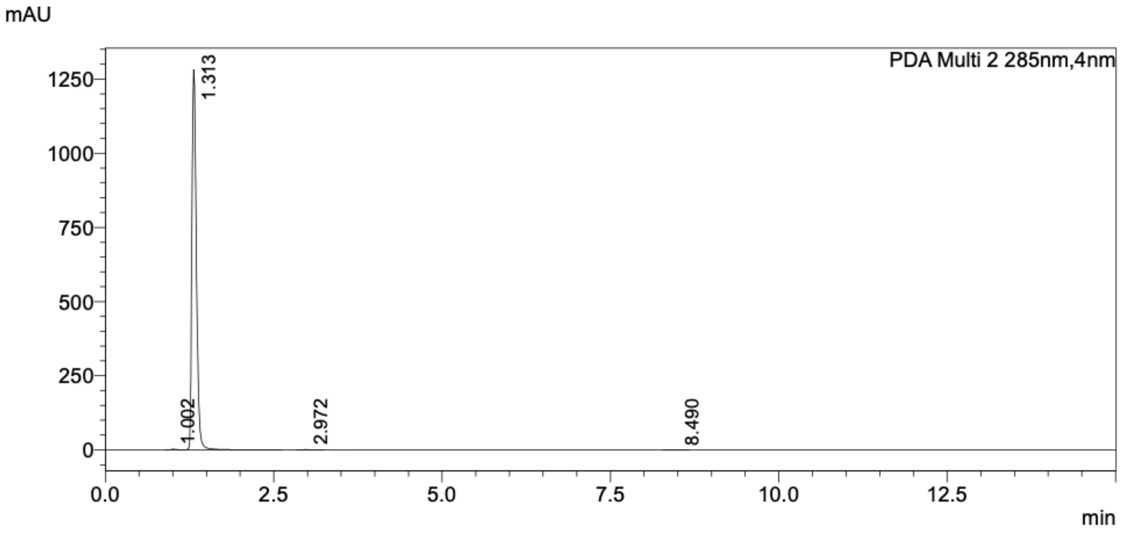

Compound 7

Analysis Report

<Sample Information>

Sample Name	: GG37		
Sample ID	: GG37		
Data Filename	: GG37.lcd		
Method Filename	: Kevin 85 ACN.lcm		
Batch Filename	: SA-TPP Test 10.10.19.lcb		
Vial #	: 1-3	Sample Type	: Unknown
Injection Volume	: 10 uL		
Date Acquired	: 10/10/2019 3:02:33 PM	Acquired by	: System Administrator
Date Processed	: 10/10/2019 3:34:32 PM	Processed by	: System Administrator

<Chromatogram>

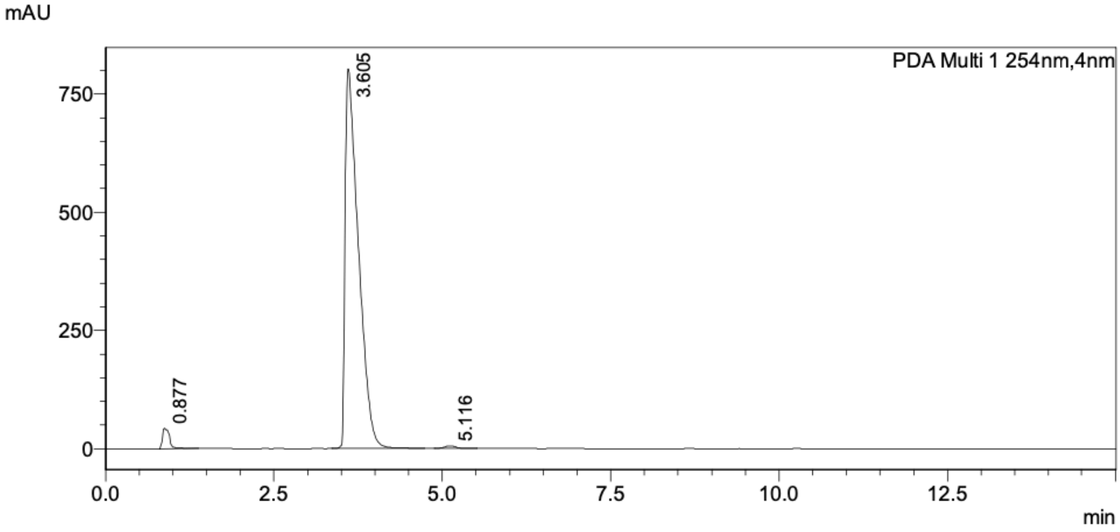

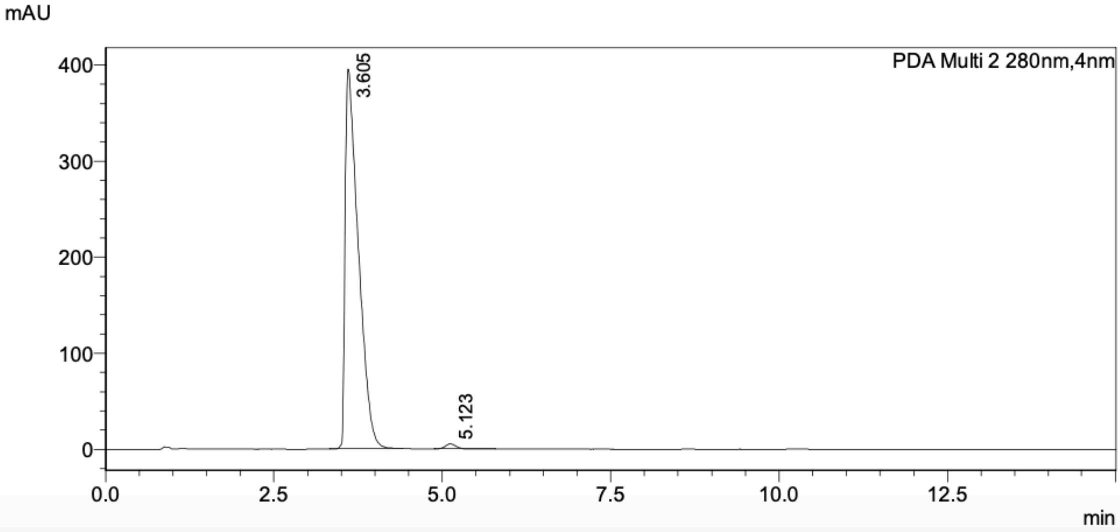

Analysis Report

<Sample Information>

Sample Name	: GG37 MeOH		
Sample ID	: GG37 MeOH		
Data Filename	: GG37 MeOH.lcd		
Method Filename	: Kevin 95 MeOH.lcm		
Batch Filename	: SA-TPP Test MeOH 10.10.19.lcb		
Vial #	: 1-3	Sample Type	: Unknown
Injection Volume	: 10 uL		
Date Acquired	: 10/10/2019 4:15:44 PM	Acquired by	: System Administrator
Date Processed	: 10/10/2019 4:57:08 PM	Processed by	: System Administrator

<Chromatogram>

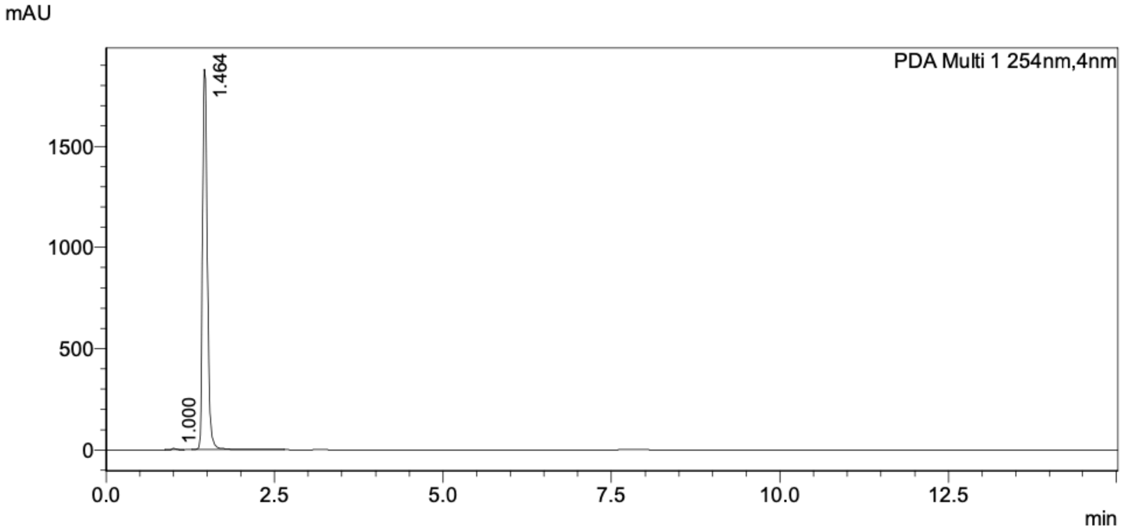

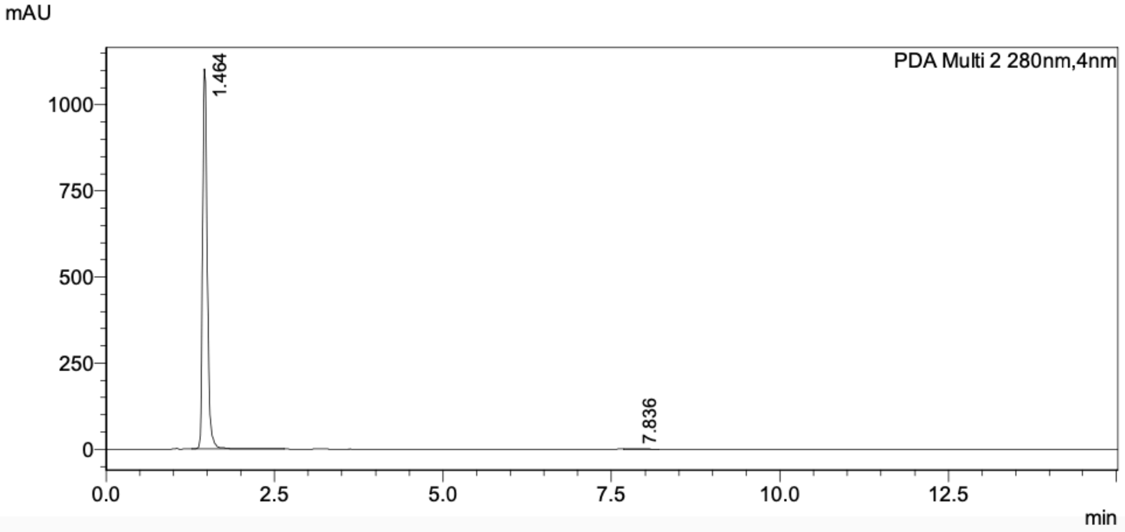

9. General Biology

Bacterial Strains and Culture Conditions
Mycobacterium tuberculosis H37Rv (ATCC 27294) and *Mycobacterium bovis* BCG (ATCC 35734) were grown at 37°C in complete Middlebrook 7H9 broth supplemented with 0.05% Tween-80, 0.5% glycerol and 10% Middlebrook albumin-dextrose-catalase or complete Middlebrook 7H10 agar supplemented with 0.5% glycerol and 10% oleic acid-albumin-dextrose-catalase. Catalase-free complete Middlebrook 7H9 broth was supplemented with 0.05% Tween-80, 0.5% glycerol, 0.5% bovine albumin, 0.2% glucose, and 0.085% NaCl. Complete Middlebrook 7H9 medium without 0.5% glycerol was prepared and used in experiments to detect glycerol dependency.

Minimum Inhibitory Concentrations (MICs) Determination
Minimum inhibitory concentrations (MICs) of test compounds were determined on *M. bovis* BCG and *Mtb* H37Rv following a previously described broth dilution method.[S1] MIC_{50} and MIC_{90} are the concentrations required to inhibit 50% and 90% of bacterial growth respectively, as compared to untreated drug free controls.

Minimum Bactericidal Concentrations (MBCs) Determination
Minimum bactericidal concentrations (MBCs) of test compounds were determined by CFU (colony forming unit) enumeration on complete Middlebrook 7H10 agar as described previously.[S14] MBC_{90}, MBC_{99}, and $MBC_{99.9}$ are the concentrations required to reduce CFUs by 10-, 100-, and 1000-fold, respectively, as compared to the untreated inoculum at time point zero.

Time-kill Kinetic Determination
The 21-day time-kill kinetic profiles of **6** was determined as follows. Briefly, pellets of mid-log-phase *M. bovis* BCG (OD_{600} 0.4 – 0.8) were spun down (3200 × *g*, 10 min) and resuspended in fresh complete Middlebrook 7H9 broth at OD_{600} 0.1. Diluted cultures were treated with test compounds at different concentrations and incubated at 37°C for 21 days with shaking. At selected time points, samples were removed for CFU determination on complete Middlebrook 7H10 agar. Isoniazid (INH) at 4× MIC_{90} (15 μM) was employed as control.

Membrane Potential and Permeability Determinations
Reported methods were followed to determine the membrane depolarizing and permeabilizing activities of test compounds on *M. bovis* BCG cultures.[S1], [S14] Briefly, *M. bovis* BCG cultures at mid-log phase were diluted at OD_{600} 0.1 in complete 7H9 broth. Diluted cultures were treated with test compounds at 4× MIC_{90} for 24 h. At selected time points, aliquots were removed from culture media and tested for changes in membrane potential using *Bac*Light^{TM} Bacterial Membrane Potential kit (Life Technologies, CA) and membrane permeability using fluorescent probes SYTO®9 and propidium iodide (PI) (Molecular Probes, Invitrogen, MA). RIF at 4× MIC_{90} (0.08 μM) was used the negative control for both assays. Carbonyl cyanide *m*-chlorophenyl hydrazone (CCCP) at 5 μM and SDS at 5% (v/v) were used as the positive controls in membrane potential and permeability tests, respectively.[S14] In the membrane depolarization assay, the dye $DiOC_2(3)$ accumulates within polarized membrane and exhibits red fluorescence; upon dissociation due to loss of membrane potential, the fluorescence emission shifts to green. Therefore, the extent of membrane depolarization is shown by the extent to which the red/green fluorescence ratio decreases. Meanwhile, in the membrane permeabilization assay, SYTO®9 exhibits green fluorescence and stains all bacteria regardless of membrane integrity, while propidium iodide (PI), which exhibits red fluorescence, only stains bacteria with damaged membrane. Therefore, a decrease in green/red fluorescence ratio indicates membrane permeabilization.

p*iniBAC* Cell Membrane Stress Reporter System
A previously reported method was followed.[S14] Briefly, test compounds and the positive control INH (0.1 − 50 μM) were incubated with the recombinant *M. bovis* BCG-p*iniBAC*-RFP strain at OD_{600} 0.2 for 24 h. Thereafter, red fluorescence (RFU, λ_{Ex} 587 nm/λ_{Em} 630 nm) and OD_{600} readings of cultures were recorded on a Tecan Infinite M200 PRO plate reader to assess induction of promoter activity. INH was employed as positive control. Normalized fluorescence readings (RFU/OD_{600}) were recorded to account for changes in cell number during the incubation period. Details of the plasmid constructs are provided in Section 11.

Reactive Oxygen Species (ROS) Detection by CellROX^{TM} Green Reagent
The generation of reactive oxygen species (ROS) in test compound-treated *M. bovis* BCG cultures was monitored using CellROX^{TM} Green Reagent (Invitrogen, MA). To monitor the dose-dependent ROS generation, *M. bovis* BCG pellets at mid-log phase were harvested by centrifugation (3200 × *g*, 10 min) and adjusted to OD_{600} 0.3 in complete 7H9 broth. Diluted cultures were treated with test compounds at 0.5×, 1×, and 2× MIC_{90} at 37°C for 1.5 h. Aliquots of CellROX^{TM} Green Reagent were added to test cultures to give a final concentration of 10 μM. Cultures were then incubated at 37°C for another 30 min in the dark. OD_{600} and green fluorescence (GFU, λ_{Ex} 485 nm/λ_{Em} 520 nm) readings were then recorded on a Tecan Infinite M200 PRO plate reader to give

normalized fluorescence readings (GFU/OD_{600}). In this assay, INH and naphthoquinone were employed as negative and positive controls, respectively.

p*furA* Oxidative Stress Reporter System
A recombinant strain *M. bovis* BCG-p*furA*-RFP was used as a reporter system to detect the oxidative stress caused by test compounds. To generate this recombinant strain, *M. bovis* BCG was transformed via electroporation with an integrative plasmid carrying a kanamycin resistance gene and the mCherry Red Fluorescent Protein (mRFP) gene under the control of the promoter p*furA*. Details of the plasmid constructs are provided in Section 12. Recombinant strains were selected using kanamycin (25 μg/mL)-containing 7H10 agar plates. The evaluation of compound induced oxidative stress was carried out as follows. Briefly, *M. bovis* BCG-p*furA*-RFP at mid-log phase were spun down (3200 × *g*, 10 min) and resuspended in fresh complete 7H9 broth at OD_{600} 0.2. Diluted cultures were treated with test compounds at 0.5×, 1×, and 2× MIC_{90} at 37°C, 110 rpm for 24 h. Thereafter, OD_{600} and red fluorescence (RFU, λ_{Ex} 587 nm/λ_{Em} 630 nm) readings were taken on a Tecan Infinite M200 PRO plate reader and expressed as normalized fluorescence readings (RFU/OD_{600}) to account for changes in cell number during the incubation period. INH was employed as the negative control.

Procedure for the horseradish peroxidase (HRP) assay
A previously reported method was followed with modifications.[S15] 2 μL of test compound (200-fold concentration in DMSO), 18 μL Hank's Balanced Salt Solution (HBSS) and 40 μL dithiothreitol (DTT) solution (2.5 mM in HBSS) were sequentially added to each well in a 96-well clear plate. The contents were shaken for 45 min, 600 rpm on a plate shaker, after which was immediately added 40 μL of horse radish peroxidase-phenol red solution (150 μg/mL horseradish peroxidase, 1 mM phenol red sodium in HBSS) and the plate shaken again for another 10 min, 600 rpm. 15 μL 1M NaOH was then added to each well, agitated (1 min) and absorbance readings were read at 610nm on a microplate reader. DMSO content per well was kept at 1% v/v. Test compound and positive control naphthoquinone were tested over a range of concentrations**.** The absorbance at 610 nm was plotted against logarithmic concentration of test compound from which EC_{50} (concentration required to increase oxidized phenol red absorbance to 50% of maximum value) was determined.

Quantification of ATP
ATP levels were measured by the BacTiter-Glo™ Microbial Cell Viability Assay Kit (Promega) following the manufacturer's instruction. Briefly, *M. bovis* BCG was cultured in complete 7H9 broth until mid-log phase, then diluted to OD_{600} = 0.1 in the same media and treated with test compounds at 4× MIC_{90}. At each time point of drug treatment, 25 μL of bacterial cultures were aliquoted and treated with an equal volume of the BacTiter-Glo™ Reagent (thawed up at room temperature prior to usage) in white flat-bottom 96-well opaque Nunc plates (Thermo Fisher Scientific) for 15 minutes at room temperature in the dark. Luminescence readings were recorded on a Tecan Infinite M200 PRO plate reader. Background readings were obtained from wells containing only media and reagent. Absolute luminescence readings of DF or drug-treated bacterial cultures were acquired by subtracting the average background readings from the raw readings of samples. To translate luminescence readings to actual ATP levels, a calibration curve was generated in the same 96-well plate by exposing 10-fold serial dilutions of pure ATP solutions (0.1 to 100 nM) in 7H9 broth to BacTiter-Glo™ Reagent followed by luminescence recording. Rifampicin was used as a negative control.

Determination of Vero IC_{50} of test compounds
African Green Monkey kidney epithelial Vero E6 cells (ATCC CRL-1586) were purchased from ATCC, Manassas, VA and grown in Dulbecco's Modified Eagle Medium (DMEM) supplemented with 1% penicillin and 10% heat-inactivated fetal bovine serum. All reagents were obtained from Hyclone Laboratories, GE Healthcare. Stock solutions of test compounds (10 mM) were prepared in DMSO. Cytotoxicity of test compounds were determined with Celltitre 96 ® Aqueous One Solution (Promega, Madison, WS), following manufacturer's instructions. To each well in a 96-well plate was seeded 30,000 Vero-E6 cells in 100 μL media. The plate was incubated at 37°C, 24 h, 5% CO_2 for cell adherence. After this time, media was removed from the well by aspiration and replaced with 100 μL aliquot of fresh media (99 μL) and test compound (1 μL from a DMSO stock solution that was 200-fold more concentrated than the final concentration in the well). DMSO content in each well was 0.5% v/v. Treated plates were incubated for 48 h (5% CO_2, 37°C) after which 10 μL of Celltitre 96 ®Aqueous One Solution was added to each well (without removal of media) and incubated for 3 h. Thereafter, absorbance readings were taken at 490 nm on a Tecan Infinite M200 Pro Microplate reader. Cell viability was determined from the expression:

$$\text{Cell viability (\%)} = \frac{Absorbance\ (cells + cpd) - Absorbance\ (cpd)}{Absorbance\ (cells + vc) - Absorbance\ (vc)} \times 100\%$$

where Absorbance (cells + cpd) = absorbance of wells containing cells and test compound in vehicle (media + 0.5% DMSO); Absorbance (cpd) = absorbance of wells containing test compound in vehicle without cells (compound control); Absorbance (cells + vc) = absorbance of wells containing cells in vehicle (vc) only (untreated

cells); Absorbance (vc) = absorbance of wells containing vehicle without cells (vehicle control).The percentage viability readings were plotted against log concentration on GraphPad Prism (Version 5.0, San Diego, CA) to give a sigmoidal curve from which IC_{50} (concentration required to reduce viability by 50% compared to control/untreated cells) was obtained. The plot was constrained to ≥ 0 and ≤ 100%. At least three separate determinations were carried out.

Determination of aqueous solubilities of compounds 6 and 7
Determination of aqueous solubility was carried out on Multiscreen ® Solubility filter plates (Millipore-MSSLBPC10) from Millipore Corporation (MA, USA). The protocol (PC2445EN00, Millipore Corporation) was followed. Briefly, various concentrations of the test compound (stock solution in DMSO) were prepared in Universal buffer (pH 7.4)/acetonitrile (80:20). DMSO content was kept at 1% in final solutions. The UV absorbance of these solutions were read at pre-determined wavelengths and used to construct calibration curves for the test compounds. Next, aliquots of the stock solution of test compound (100 µM in Universal Buffer with 1% DMSO) were dispensed into wells in the Multiscreen Solubility filter plate, and agitated for 24 h at room temperature (25°C). The suspension was filtered, the filtrate diluted with acetonitrile to give the same solvent composition used to prepare the calibration solutions. The absorbance of the diluted filtrate was read at the predetermined wavelength and the concentration of the filtrate (equivalent to the solubility of the test compound) was determined from the calibration curve. The solubility determinations were carried out on at least 3 separate occasions using different stock solutions.

10. Cytotoxicity determination on Vero E6 cells

Table S2 Summary of biological activities of test compounds.

Compound	Structure	clogP[a]	*M. bovis* BCG		Vero E6	SI[d]
			MIC_{50} (µM)[b]	MIC_{90} (µM)[b]	IC_{50} (µM)[c]	
**3**	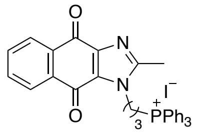	7.17	4.0 (twice)	5.6 (5.8, 5.5)	>400	>100
**4**	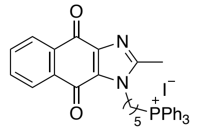	8.05	2.4 (2.6, 2.2)	4.6 (twice)	>400	>166.67
**5**	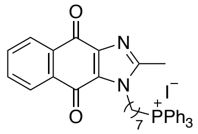	9.10	1.2 (twice)	2.3 (2.4, 2.2)	48.4	40.3
**6**	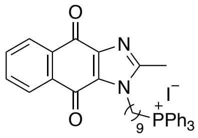	10.16	0.59 (0.6, 0.58)	1 (twice)	12.4	21.0
**7**	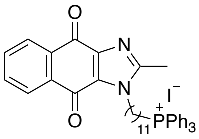	11.22	0.5 (0.6, 0.5)	0.8 (0.9, 0.7)	5.6	11.2
**8**	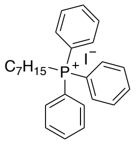	9.31	5.5 (6.7, 4.4)	10.5 (10.2, 10.8)	-	-
**9**	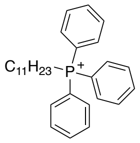	11.42	2.1 (2, 2.2)	2.8 (2.7, 2.8)	-	-
**10**	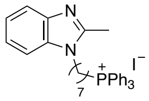	9.66	3.8 (twice)	5.6 (5.7, 5.6)	86.4	22.7

**11**	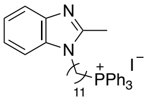	11.78	0.9 (0.9, 1.0)	1.4 (twice)	11.5	12.78
**12**	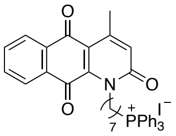	9.53	1 (1.05, 1)	1.4 (twice)	5.9	5.9
**13**	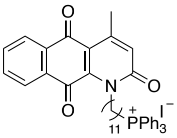	11.65	3.8 (3.6, 4.05)	5.7 (5.5, 5.9)	2.1 (2.6, 1.6)	0.6
**14**	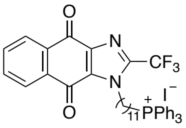	11.83	1.8 (1.7, 1.9)	2.8 (2.7, 2.8)	5.6	3.12
**15**	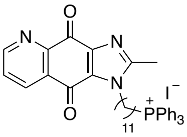	9.72	0.6 (0.6, 0.7)	1.2 (1.2, 1.3)	5.4 (7.6, 5.7, 2.9)	9.0
**16**	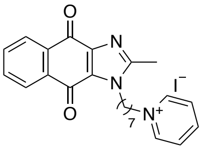	-1.23	28.7 (32.5, 35.0)	48.5 (48.0, 49.0)	-	-
**17**	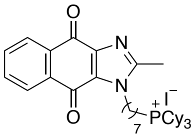	4.91	1.3 (twice)	2.5 (2.6, 2.5)	24.4 (29.8, 28.8, 14.5)	18.8
**18**	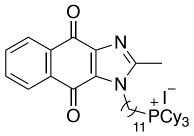	7.02	0.6 (twice)	0.9 (1.0, 0.8)	4.1	6.8
**19**	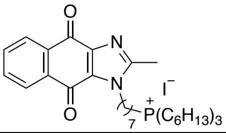	11.81	0.7 (twice)	1.4 (twice)	5.2	7.5

[a] clogP values were estimated using ChemDraw Professional 16.0. [b] Minimum inhibitory concentration required to reduce growth by 50% (MIC_{50}) or 90% (MIC_{90}) compared to untreated controls. [c] Concentration required to reduce growth of Vero E6 (African Green Monkey kidney epithelial cells) by 50% compared to untreated controls. [d] Selectivity Index = $IC_{50\ Vero}/MIC_{50\ BCG}$. MIC and IC_{50} values were the average of two or more separate determinations.

11. Plasmid and primers used in the construction of *M. bovis* BCG-p*iniBAC*-RFP reporter system

Plasmid name	Vector (Digested with)	Inserted Promoters (PCR-amplified DNA fragments)				Reporter gene (PCR-amplified DNA fragments)	
		Gene	Size (bp)	Primer Name	Primer Sequence	Primer Name	Primer Sequence
2 P-iniB-RFP	pMV306 (NotI---EcoRI)	BCG_0380	191	P-iniB-F(NotI)	gcggccgcTAAGTTCCGGACCGGCGTA	mCh-F(BamH)	ccgggatccATGGTGAGCAAGGGCGAGG
				P-iniB-R(BamH)	ccgggatccCTTCATTTCCCTTCAATCGAAGA	mCh-R(EcoRI)	ccggaattcCTACTTGTACAGCTCGTCCAT

12. Plasmid and primers used in the construction of *M. bovis* BCG-p*furA*-RFP reporter system

Plasmid name	Backbone plasmid (Digested with)	**Inserted Promoter** (PCR-amplified DNA fragments digested with NotI and BamHI)				**Inserted Reporter** (PCR product digested with BamH and EcoRI)	
		Upstream of	Size (bp)	Primer Name	Primer Sequence	Primer Name	Primer Sequence
P-furA/katG-RFP	pMV306 (NotI---EcoRI)	BCG_1948c	250	P-furA/katG-F(NotI)	gcggccgcCACACCACTACCGGTTTACCCTC	mCh-F(BamHI)	ccgggatccATGGTGAGCAAGGGCGAGG
		Rv1909c		P-furA/katG-R(BamHI)	ccgggatccACTAGACAATATGACTCCCTTTTCTG	mCh-R(EcoRI)	ccggaattcCTACTTGTACAGCTCGTCCAT

13. References

[S1] M. Li, S. A. Nyantakyi, P. Gopal, D. b. Aziz, T. Dick, M.-L. Go, *ACS Med. Chem. Lett.* **2017**, *8*, 1165-1170.

[S2] B. Chesneau, M. Hardouin-Lerouge, P. Hudhomme, *Org. Lett.* **2010,** *12* (21), 4868- 4871.

[S3] L. Pan, Q. Zheng, Y. Chen, R. Yang, Y. Yang, Z. Li, X. Meng, *Eur. J. Med. Chem.* **2018,** *157*, 423-436.

[S4] Z. Liu, Z. Zhang, W. Zhang, D. Yan, *Bioorg. Med. Chem. Lett.* **2018**, *28*, 2454-2458.

[S5] T. Schlama, V. Gouverneur, C. Mioskowski, *Tetrahedron Lett.* **1997**, *38* (20), 3517-3520.

[S6] Vogel's Textbook of Practical Organic Chemistry, 5th ed., Longman Scientific & Technical, Chelmsford, **1989**, p. 558.

[S7] H. Ihara, M. Takafuji, C. Hirayama, D. F. O'Brien, *Langmuir* **1992**, *8*, 1548.

[S8] T. Belser, M. Stöhr, A. Pfaltz, *J. Am. Chem. Soc.* **2005**, *127*, 8720-8731.

[S9] K. T. Fridianto, M. Li, K. Hards, D. A. Negatu, G. M. Cook, T. Dick, Y. Lam, M.-L. Go, *J. Med. Chem.* **2021,** *64* (21), 15991-16007.

[S10] M. N. Gandy, M. J. Piggott, *J. Nat. Prod.* **2008**, *71* (5), 866-868.

[S11] P. A. Evans, T. A. Brandt, *J. Org. Chem.* **1997,** *62* (16), 5321- 5326.

[S12] R. Tapia, G. Torres, J.A. Valderrama, *Synth. Comm.* **1986,** *16* (6), 681-687.

[S13] P. López-Alvarado, C. Avendaño, J.C. Menéndez, *Synth. Comm.* **1992,** *22* (16), 2329-2333.

[S14] S. A. Nyantakyi, M. Li, P. Gopal, M. Zimmerman, V. Dartois, M. Gengenbacher, T. Dick, M.-L. Go, *J. Med. Chem.* **2018***, 61*, 5733-5750.

[S15] S. Ahenkorah, D. Coertzen, J. X. Tong, K. Fridianto, S. Wittlin, L.-M. Birkholtz, K. S. W. Tan, Y. Lam, M.-L. Go, R. K. Haynes, *ACS Med. Chem. Lett.* **2020**, 11, 49-55.
